# Structure–Activity Relationships and Evaluation of 2-(Heteroaryl-cycloalkyl)-1*H*-indoles as Tauopathy Positron Emission Tomography Radiotracers

**DOI:** 10.1021/acs.jmedchem.4c02988

**Published:** 2025-03-11

**Authors:** Jeffrey S. Stehouwer, Guofeng Huang, Dinahlee Saturnino Guarino, Manik L. Debnath, Ashok Polu, Steven J. Geib, Brian Lopresti, Milos D. Ikonomovic, Neale Mason, Robert H. Mach, Chester A. Mathis

**Affiliations:** † Department of Radiology, University of Pittsburgh, Pittsburgh, Pennsylvania 15213, United States; ‡ Department of Radiology, University of Pennsylvania, Philadelphia, Pennsylvania 19104-6323, United States; § Department of Psychiatry, University of Pittsburgh, Pittsburgh, Pennsylvania 15213, United States; ∥ X-ray Crystallography Laboratory, Department of Chemistry, University of Pittsburgh, Pittsburgh, Pennsylvania 15213, United States; ⊥ Geriatric Research and Clinical Education, VA Pittsburgh Healthcare System, Pittsburgh, Pennsylvania 15240, United States

## Abstract

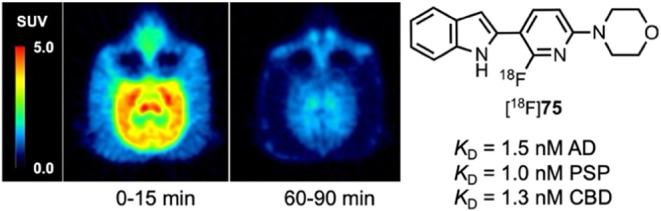

Structure–activity relationship studies were performed on a library of synthesized compounds based on previously identified tau ligands. The top 13 new compounds had *K*_i_ values in the range of 5–14 nM in Alzheimer’s disease (AD), progressive supranuclear palsy (PSP), and corticobasal degeneration (CBD) post-mortem brain tissues. One of the more promising new compounds ([^3^H]**75**) bound with high affinity in AD, PSP, and CBD tissues (*K*_D_’s = 1–1.5 nM) and Pick’s disease tissue (*K*_D_ = 3.8 nM). Autoradiography studies with [^3^H]**75** demonstrated specific binding in AD, PSP, and CBD post-mortem tissues. Nonhuman primate brain PET imaging with [^18^F]**75** demonstrated a peak standardized uptake value (SUV) of ∼5 in the cerebellum, ∼4.5 in the cortex, and ∼4 in whole brain with SUV 2-to-90 min ratios of 3.9 in whole brain, 4.9 in cortex, and 4.5 in cerebellum. Compound [^18^F]**75** is a promising candidate for translation to human brain PET imaging studies.

## Introduction

**Figure 1:**
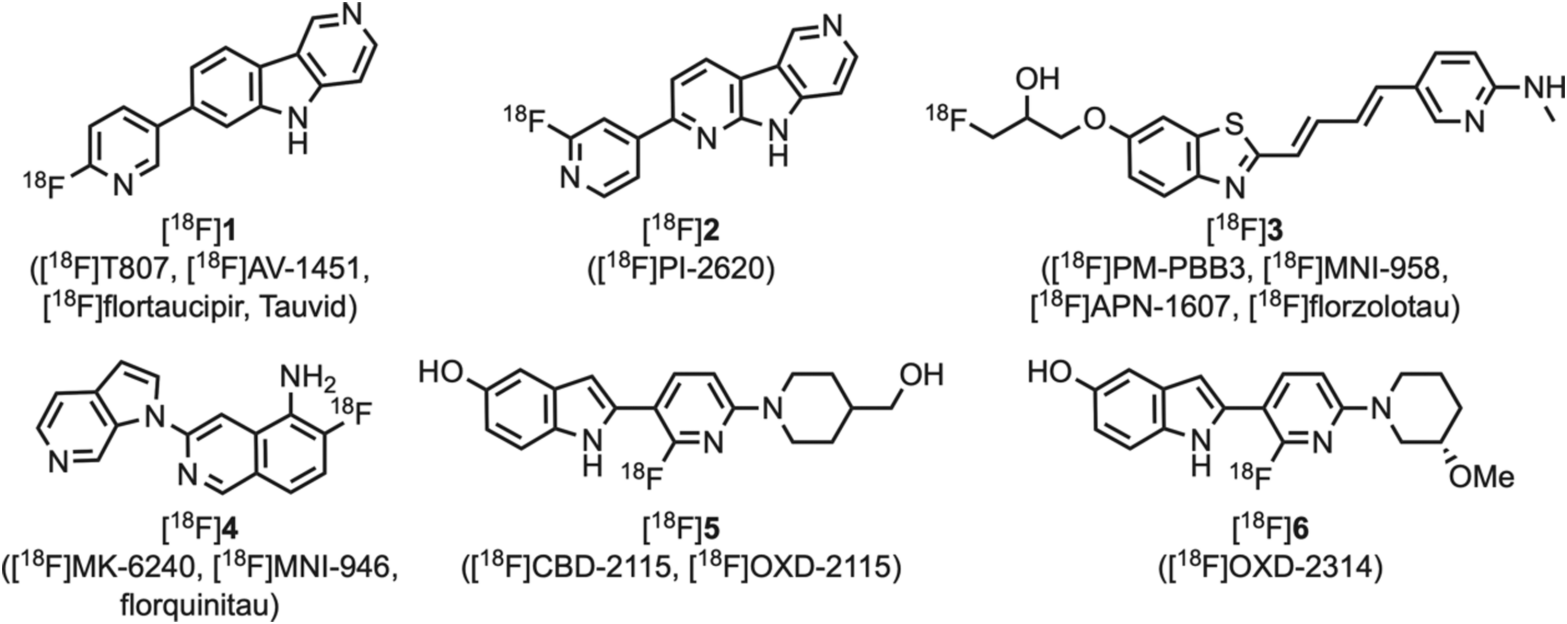
Several previously reported tau PET tracers.

Positron emission tomography (PET) imaging of aggregated microtubule-associated protein tau^1,2^ in the living human Alzheimer’s disease (AD) brain has enabled the assessment of tau burden, disease staging, and monitoring of disease progression.^3−6^ The commonly used tau PET tracers^7^ [^18^F]**1** ([^18^F]T807, [^18^F]AV-1451, [^18^F]flortaucipir, Tauvid), [^18^F]**2** ([^18^F]PI-2620), [^18^F]**3** ([^18^F]PM–PBB3, [^18^F]MNI-958, [^18^F]APN-1607, [^18^F]florzolotau), and [^18^F]**4** ([^18^F]MK-6240, [^18^F]MNI-946, florquinitau) (Figure 1) have demonstrated the ability to image AD-tau^8,9^ but can also suffer from off-target binding^10−16^ or are less effective in other tauopathies.^17−19^ Thus, more effective tau PET tracers are needed to successfully image aggregated tau in non-AD tauopathies such as chronic traumatic encephalopathy (CTE), Pick’s disease (PiD), corticobasal degeneration (CBD), and progressive supranuclear palsy (PSP).^20−25^

Tau in adult human brain consists of six isoforms resulting from 0, 1, or 2 inserts in the N-terminal domain (0N, 1N, 2N) and either three or four imperfect repeats (3R, 4R) in the microtubule-binding domain.^26−29^ Aggregates of the different isoforms constitute different tauopathies with AD and CTE filaments being composed primarily of mixed 3R/4R-tau, PiD filaments being composed mainly of 3R-tau, and CBD and PSP filaments being composed predominantly of 4R-tau.^30,31^ Recent work with cryogenic electron microscopy (cryo-EM) has demonstrated that the different tauopathies can be classified according to the structure formed by the tau filaments.^29,32−34^ Computer modeling studies of tau fibrils have predicted several potential binding sites for existing PET tracers,^35−40^ and cryo-EM structures with bound PET ligands have provided insight into possible binding modes of these compounds.^41−43^ Thus, to image non-AD tauopathies with PET, it may be necessary to develop ligands that bind with high affinity to sites on tau filaments other than those where compounds [^18^F]**1**–[^18^F]**4** bind. In an effort to develop a more potent and effective 4R-tau PET tracer, [^18^F]**5** ([^18^F]CBD-2115, [^18^F]OXD-2115) (Figure 1) was developed, but it suffered from low brain entry as shown by PET studies in mouse, rat, and nonhuman primate.^44^ Structural modifications of **5** resulted in the identification of [^18^F]**6** ([^18^F]OXD-2314), which demonstrated improved brain entry in rat and nonhuman primate PET imaging studies and high binding affinity in AD, PSP, CBD, and PiD post-mortem brain tissue homogenate assays.^45^

**Figure 2:**
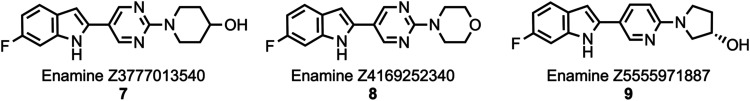
4R-tau ligands identified through a previous *in silico* chemical database fingerprint search.^46^

Compounds **7**, **8**, and **9** (Figure 2) were previously identified utilizing an *in silico* chemical database fingerprint search of the Enamine REAL collection.^46^ Evaluation of [^3^H]**7** and [^3^H]**8** in post-mortem human AD, PSP, CBD, and PiD brain tissue homogenates demonstrated that these compounds bind with high affinity to aggregated tau, yet **7** and **8** did not compete effectively against [^3^H]**2** or the structurally similar aryl-indole [^3^H]**5** in AD, PSP, and CBD brain tissue homogenate assays. Compounds **7** and **8** did compete against [^3^H]**3** in AD, PSP, and CBD brain tissue homogenate assays, thus demonstrating that **3**, **7**, and **8** apparently bind to similar locations on both aggregated mixed 3R/4R- and 4R-tau. With the goal of identifying even more potent tau ligands and improved selectivity for 4R-tau over mixed 3R/4R- and 3R-tau species, structure–activity relationship (SAR) studies were performed by synthesizing and evaluating a library of compounds based on **7**, **8**, and **9** with potential sites for F-18 radiolabeling at the 5- or 6-position of the indole ring,^47−51^ the center pyrimidine/pyridine ring,^52−54^ or the cycloalkyl ring.^55−60^

## Results and Discussion

### Chemical Synthesis

**Figure 3:**
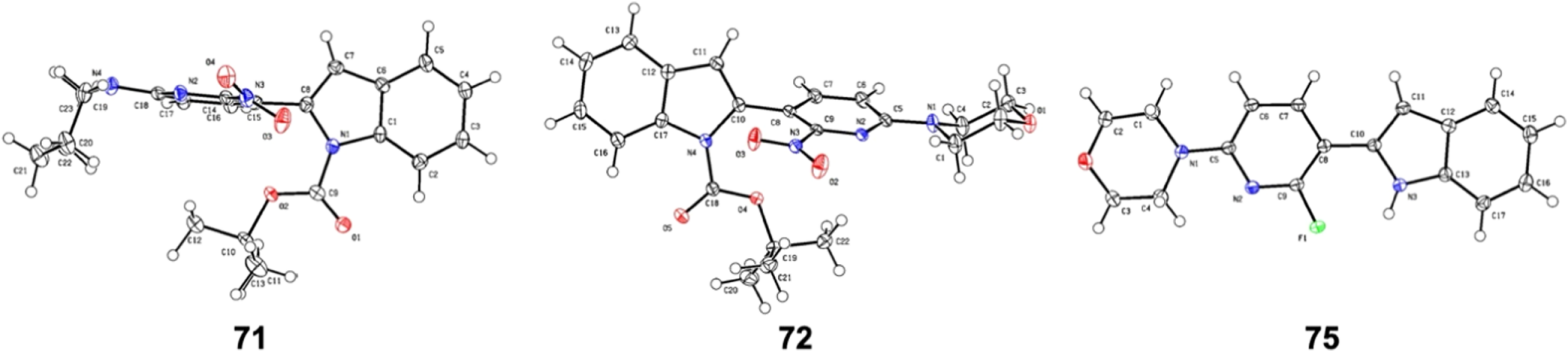
X-ray crystal structures of compounds **71**, **72**, and **75** (CCDC Deposition Numbers 2403955, 2403957, and 2403958).

**Scheme 1:**
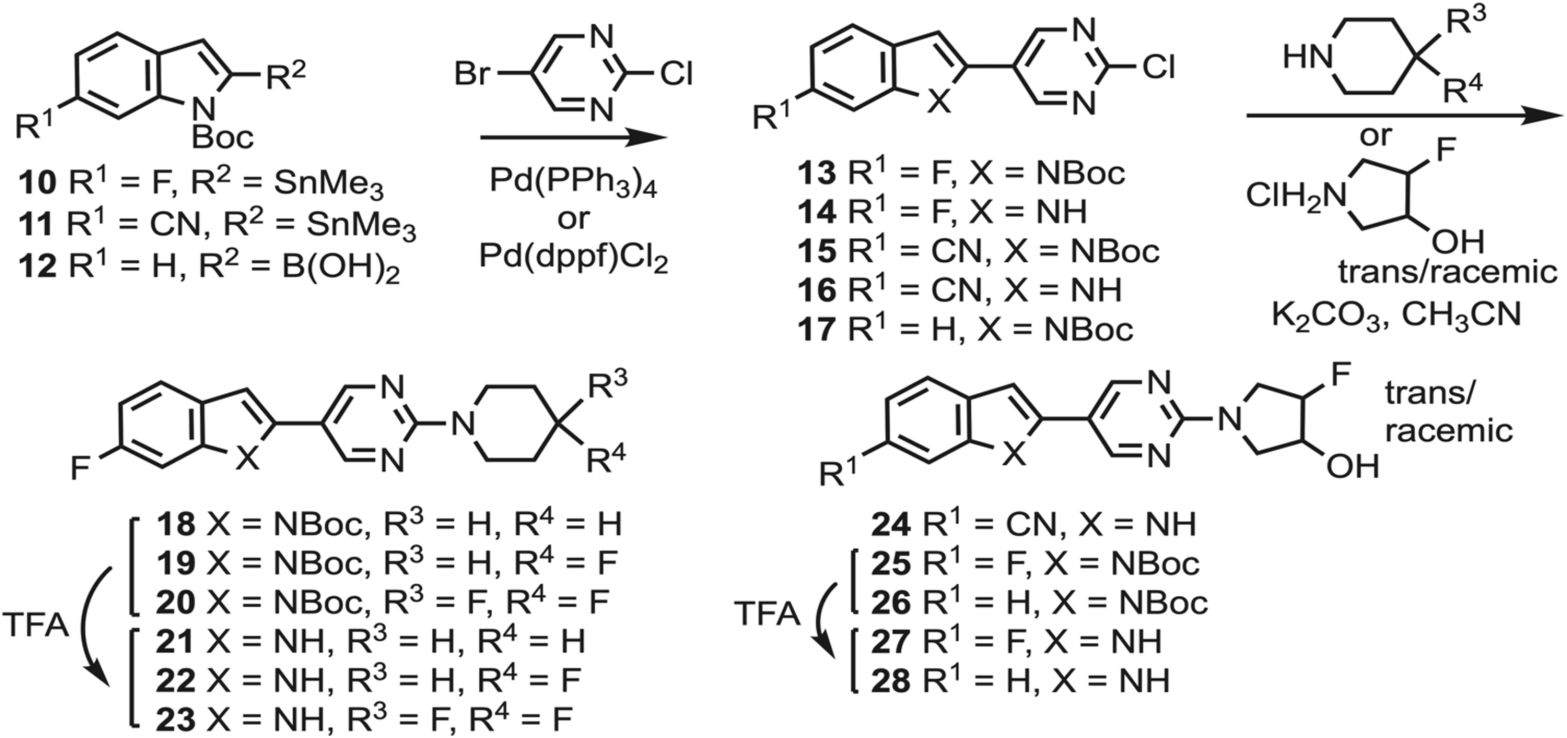
Synthesis of Compounds **21**–**23**, **24**, **27**, and **28**

**Scheme 2:**
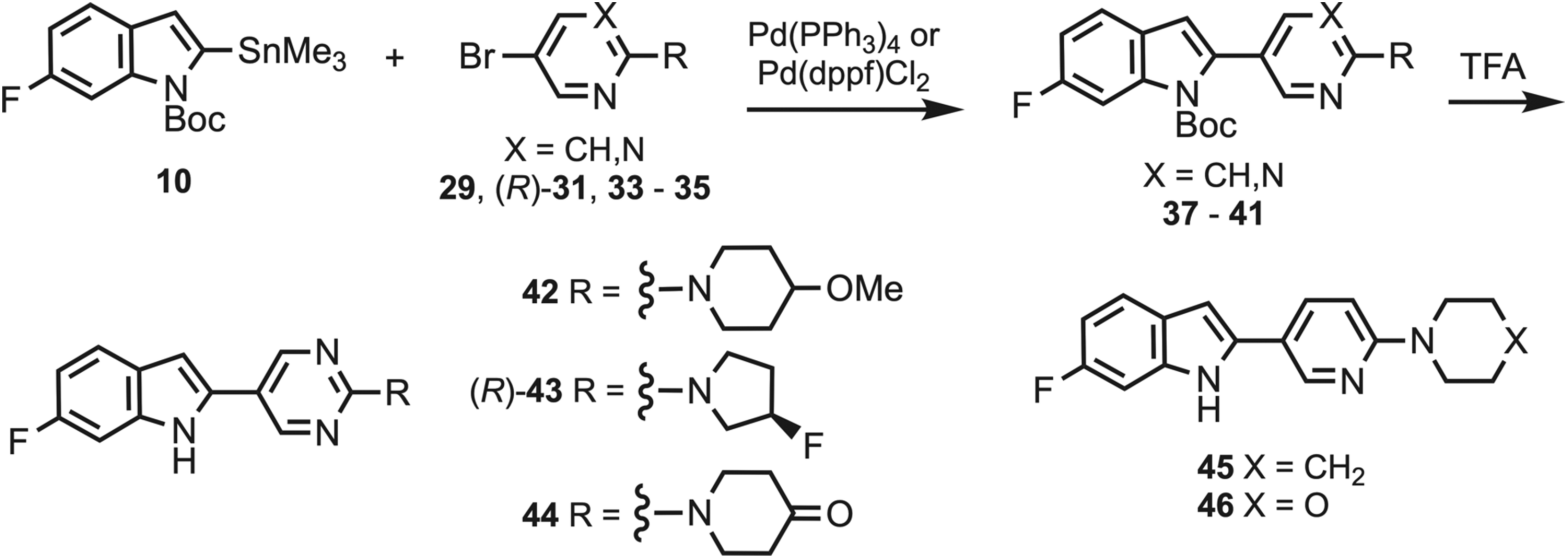
Synthesis of Compounds **42**–**46**

**Scheme 3:**
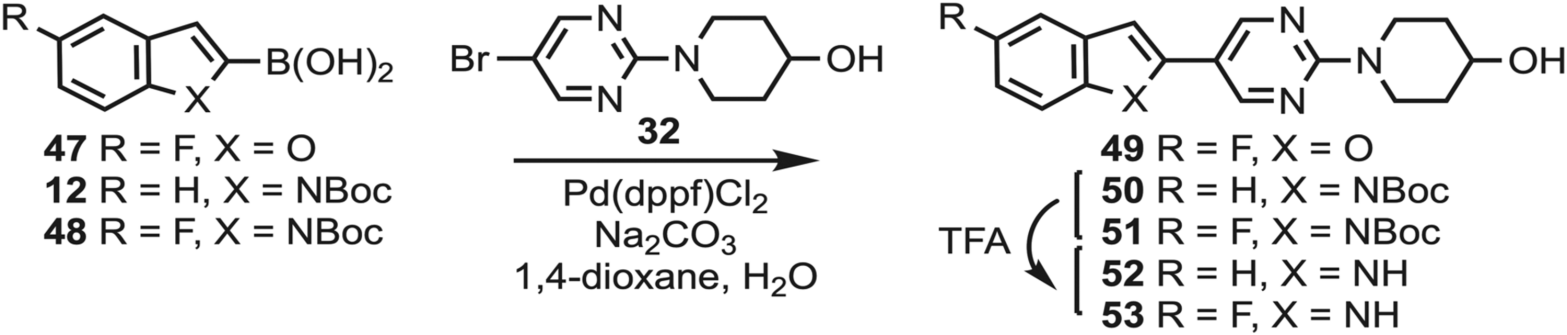
Synthesis of Compounds **49**, **52**, and **53**

**Scheme 4:**
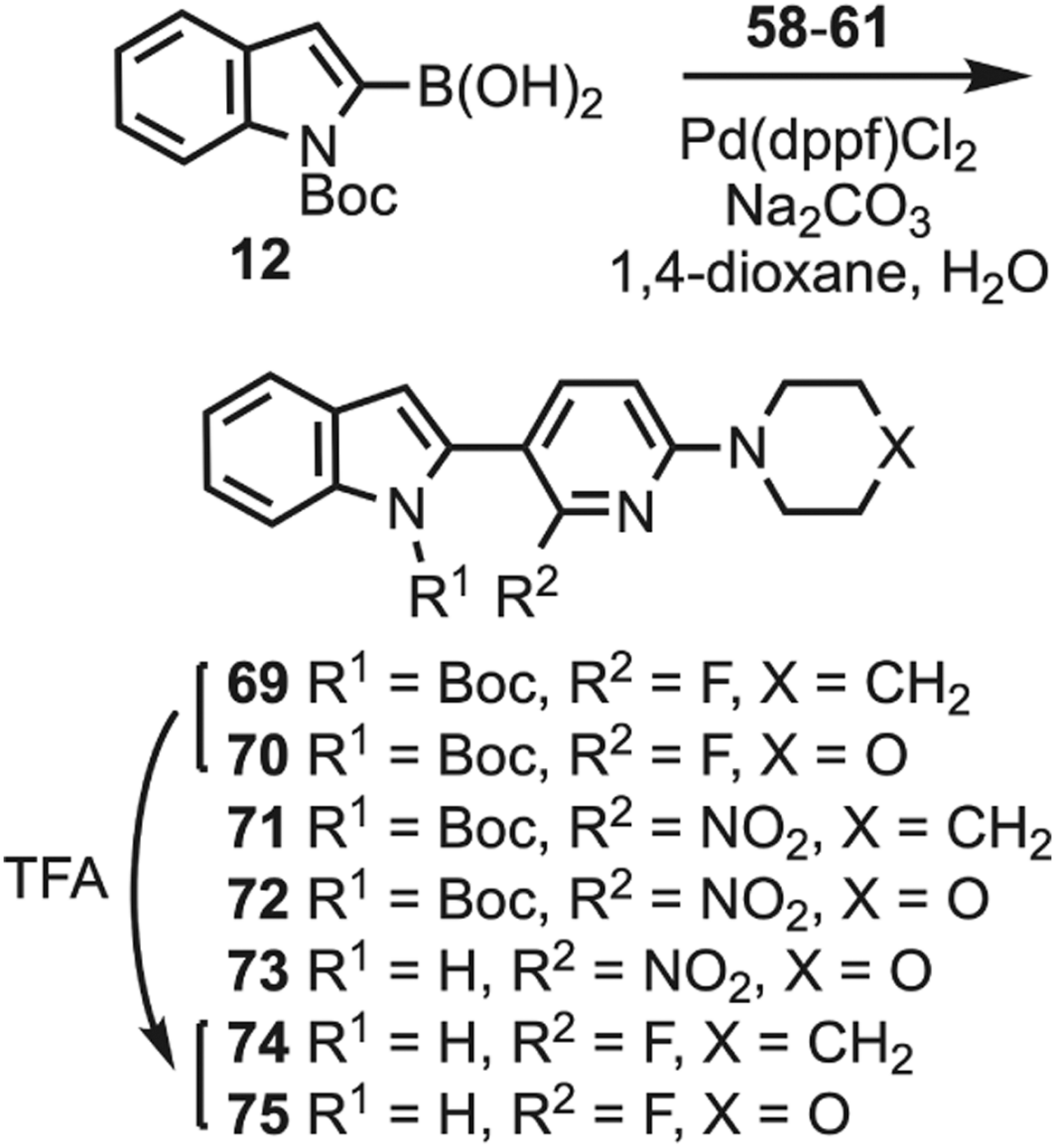
Synthesis of Compounds **71**–**75**

Compounds **10** and **11** (Scheme 1) were prepared according to a previously described procedure;^61^ compound **12** is commercially available. Compounds **10**–**12** were coupled to 5-bromo-2-chloropyrimidine to give intermediates **13**–**17** which were reacted with various cyclic amines to give compounds **18**–**20** and **24**–**26**. *N*-Boc deprotection then afforded compounds **21**–**23**, **27**, and **28**. Substituted heterocyclic intermediates **29**–**36** (Scheme S1, Supporting Information) were coupled to **10** (Scheme 2) to give intermediates **37**–**41** which were then deprotected to give compounds **42**–**46** (the structure of **44** was confirmed by X-ray crystallography, Figure S1, Supporting Information). Commercially available compounds **47**, **12**, and **48** (Scheme 3) were coupled to **32** to give compounds **49**–**51**, followed by *N*-Boc deprotection to afford compounds **52** and **53**. Substituted pyridine intermediates **54**–**57** and **62** (Scheme S2, Supporting Information) were brominated with *N*-bromosuccinimide (NBS) to give compounds **58**–**61** and **63**.^62^ Substituted pyridines **64**–**68** (Scheme S3, Supporting Information) were obtained by coupling either piperidine or morpholine^63,64^ to commercially available pyridines. Compounds **58**–**61** were coupled to compound **12** (Scheme 4) to give compounds **69**–**73**, followed by deprotection to give compounds **74** and **75**. The chemical structures of **71**, **72**, and **75** were confirmed by X-ray crystallography (Figure 3).

**Scheme 5:**
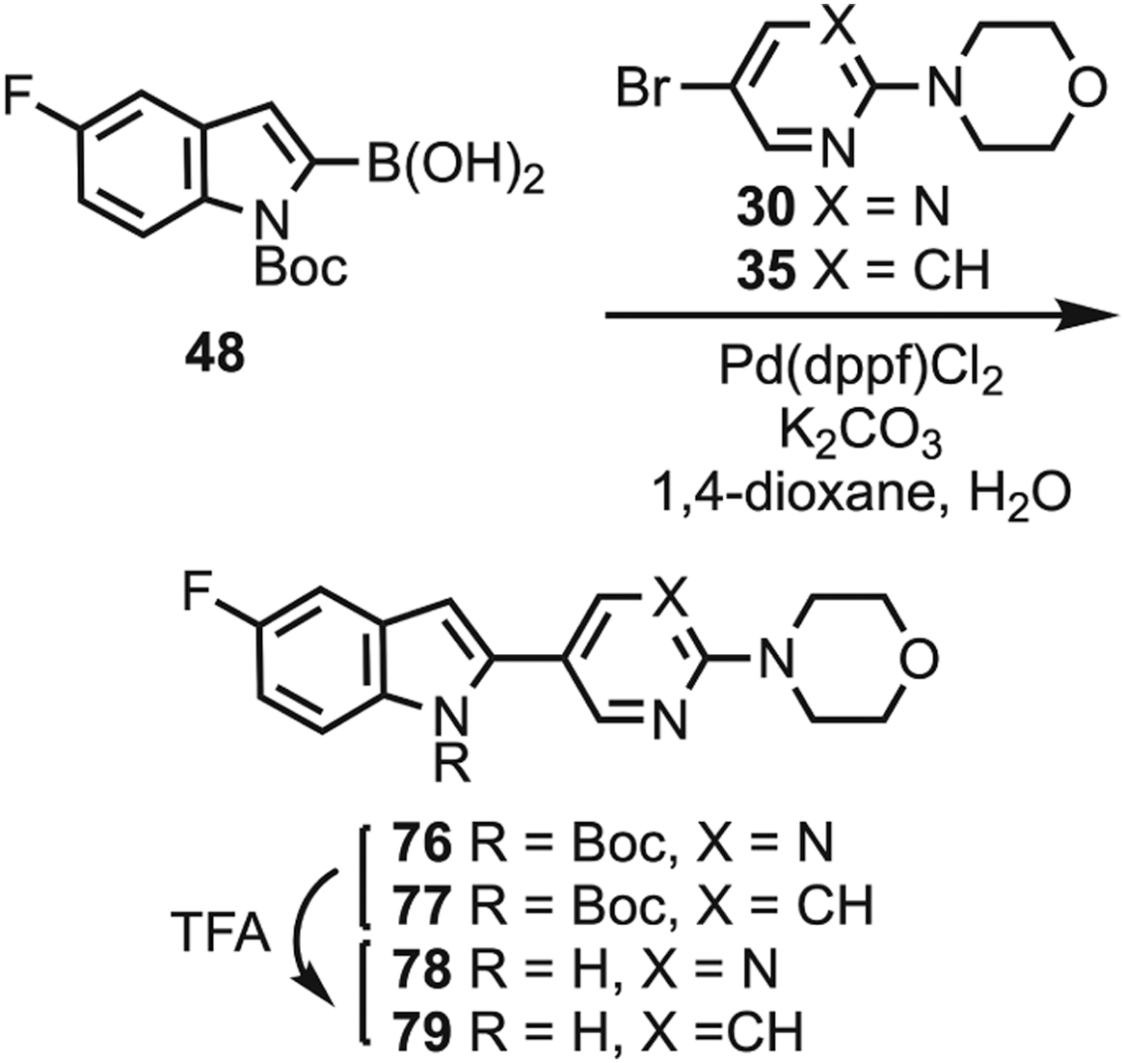
Synthesis of Compounds **78** and **79**

**Scheme 6:**
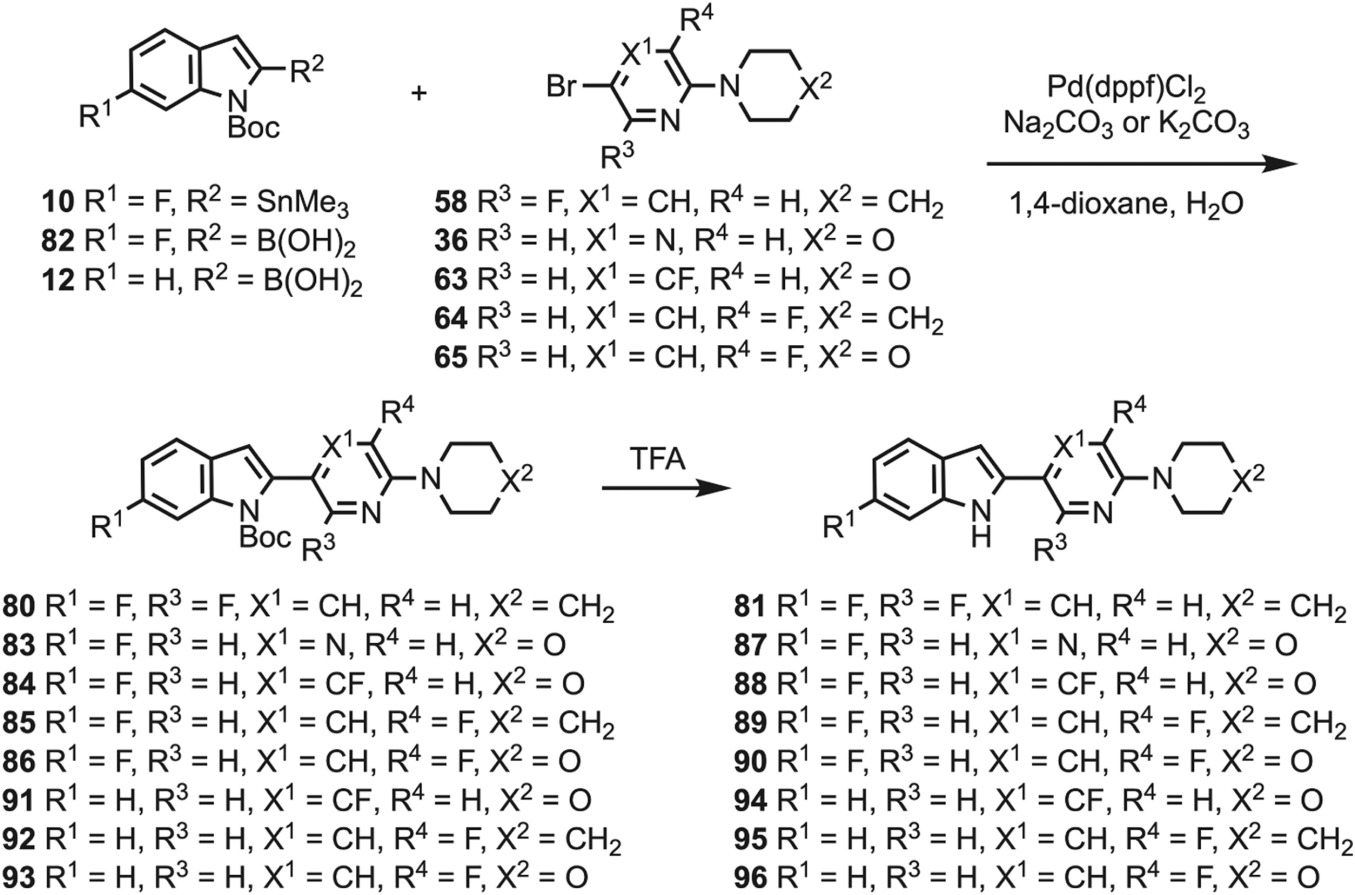
Synthesis of Compounds **81**, **87**–**90**, and **94**–**96**

**Scheme 7:**
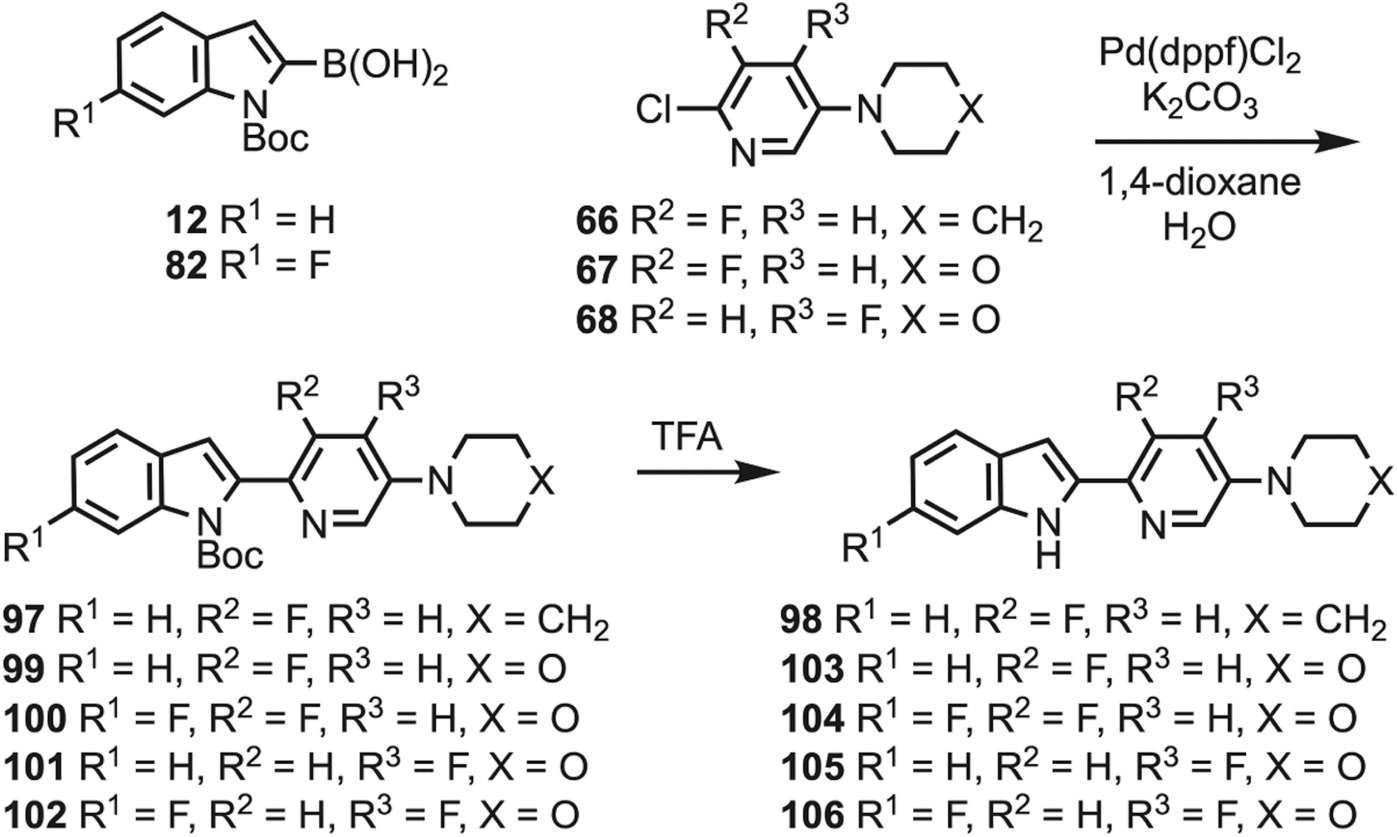
Synthesis of Compounds **98** and **103**–**106**

**Scheme 8:**
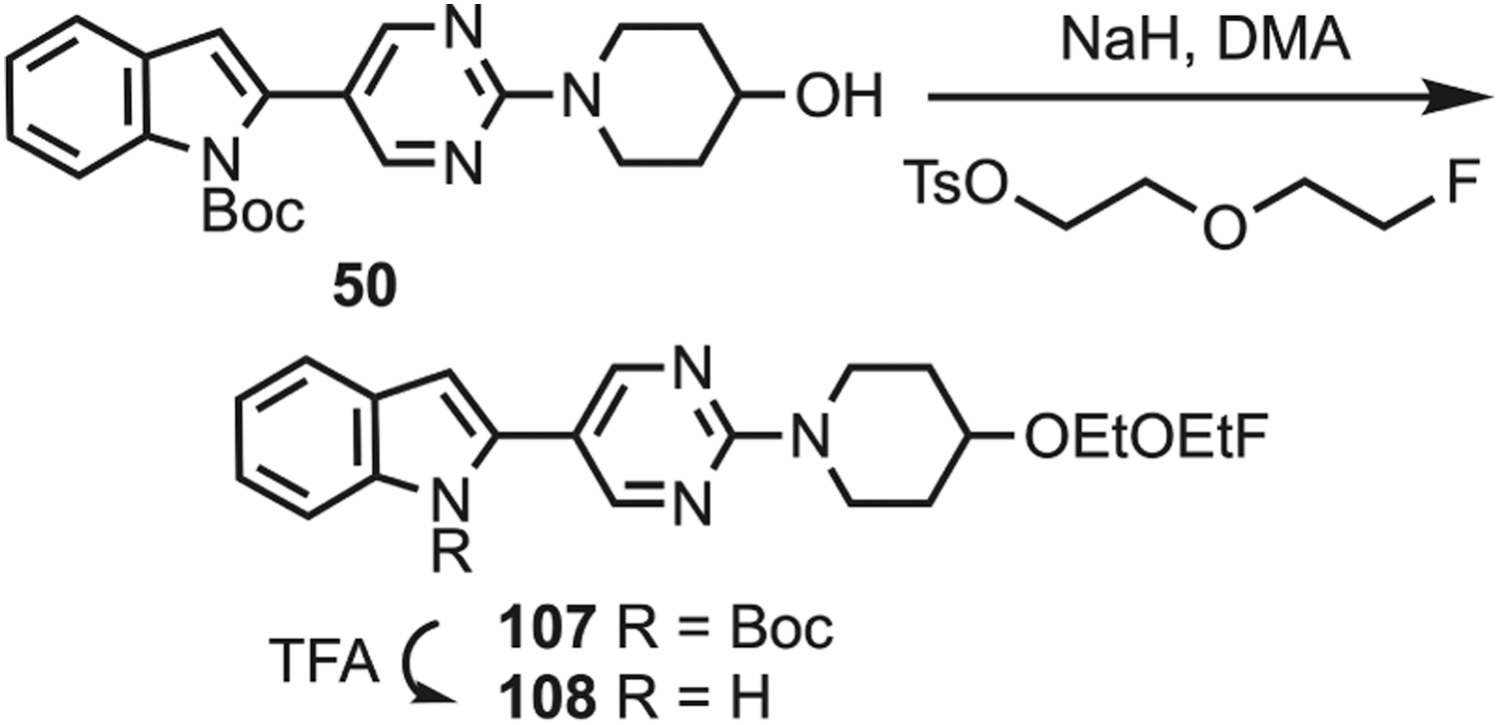
Synthesis of Compound **108**

**Scheme 9:**
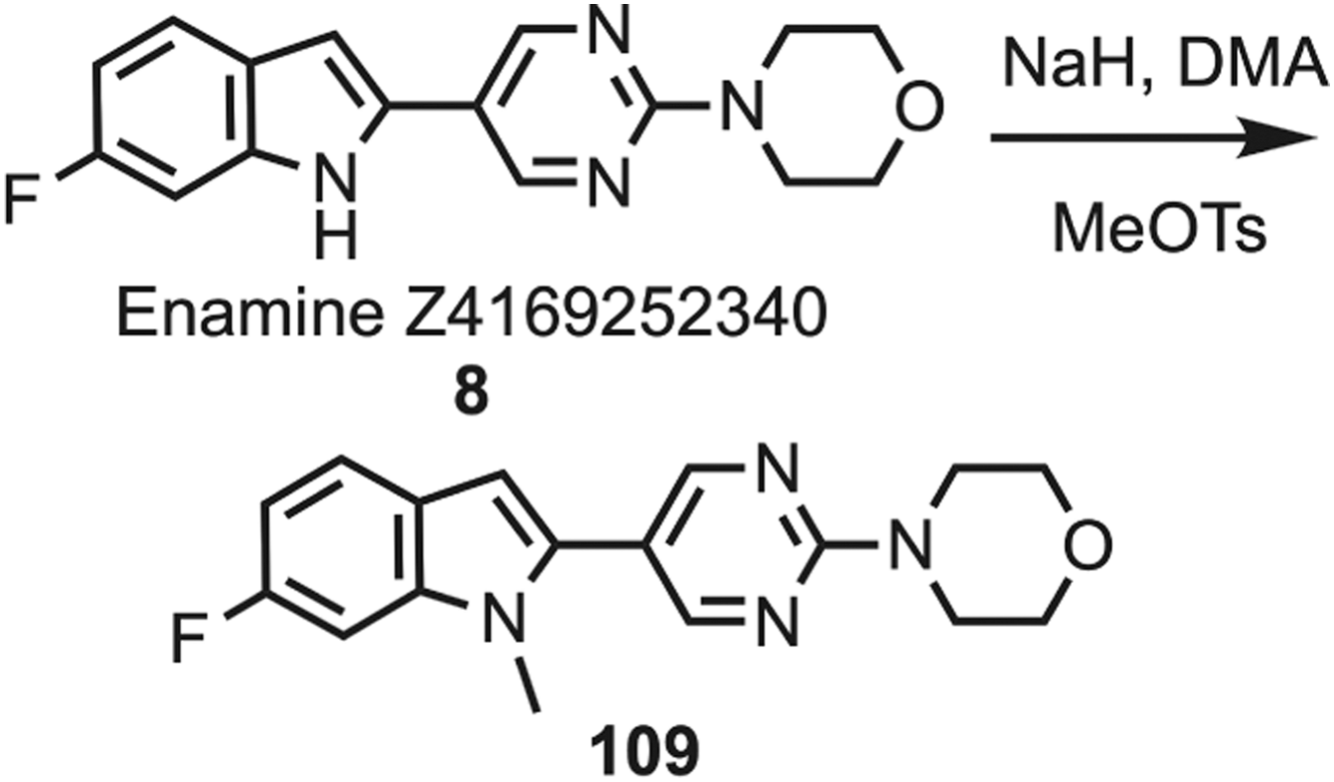
Synthesis of Compound **109**

Compound **48** was coupled to compounds **30** and **35** (Scheme 5) to give compounds **76** and **77**, followed by deprotection to give compounds **78** and **79**. Compounds **10**, **82**, and **12** were coupled to substituted heterocycles (Scheme 6) to give compounds **80**, **83**–**86**, and **91**–**93** followed by deprotection to afford compounds **81**, **87**–**90**, and **94**–**96**. Compounds **12** and **82** were coupled to substituted pyridines (Scheme 7) to give compounds **97** and **99**–**102**, followed by deprotection to afford compounds **98** and **103**–**106**. Compound **50** was *O*-alkylated (Scheme 8) to give compound **107** followed by deprotection to give compound **108**, while compound **8** was *N*-methylated (Scheme 9) to afford compound **109**. A library of compounds was thus synthesized with variable substitution patterns for SAR testing while also maintaining a fluorine atom as a potential site for radiolabeling with F-18. Additionally, compounds **42** and **109** can potentially be radiolabeled with C-11(as *O*-[^11^C]CH_3_ or *N*-[^11^C]CH_3_).

### Binding Assays and SAR Studies

All screened compounds were >95% pure as determined by analytical HPLC (Table S8 and Figures S2–S7, Supporting Information). *In vitro* binding assays in post-mortem brain tissue homogenates were performed as previously described.^46,65,66^ The inhibition constant (*K*_i_) values of all screened candidate compounds versus [^3^H]**7**, [^3^H]**8**, and [^3^H]**3** are shown in Table S1 (Supporting Information). Derivatives of **7** were prepared where the 4-hydroxypiperidine ring was held constant (compounds **49**, **52**, and **53**) and where the 4-hydroxy group was *O*-alkylated (compound **108**) (Table S2, Supporting Information). Moving the 6-fluoro group of **7** to the 5-position of the indole ring to give **53**, or removing the fluorine atom completely to give **52**, did not change the affinity in PSP tissue relative to **7** when competed against [^3^H]**7**, while *O*-alkylation of **52** to give **108** also did not change the affinity in PSP tissue relative to **7** when competed against [^3^H]**7**, but did result in a loss of affinity in AD, PSP, and CBD tissue relative to **7** when competed against [^3^H]**8**. Changing the indole ring of **53** to benzofuran to give **49** resulted in a loss of affinity in AD and PSP tissue when competed against [^3^H]**7**, suggesting that the indole NH group may be necessary as an H-bond donor.

The 6-fluoroindole and pyrimidine rings of **7** were held constant while the 4-substituent of the piperidine ring was varied to give compounds **21**, **22**, **23**, **42**, and **44** (Table S3, Supporting Information). *O*-methylation of the hydroxy group of **7** to give **42** did not significantly alter binding affinity in AD, PSP, or CBD tissue relative to **7** when competed against [^3^H]**7**, [^3^H]**8**, or [^3^H]**3**, indicating *O*-methylation as a potential strategy to increase brain uptake for PET imaging relative to what was observed for [^18^F]**7**.^46^ Conversion of the hydroxy group of **7** to a carbonyl group to give **44** did not improve affinity when competed against [^3^H]**7** or [^3^H]**8**, and resulted in a loss of affinity when competed against [^3^H]**3**. Removal of the hydroxy group of **7** to give the unsubstituted piperidine **21** improved the affinity in AD, PSP, and CBD when competed against [^3^H]**7**, maintained affinity when competed against [^3^H]**8**, and reduced affinity slightly when competed against [^3^H]**3**. Replacing the hydroxy group of **7** with fluorine to give **22** did not improve affinity when competed against [^3^H]**7** or [^3^H]**8** and resulted in a loss of affinity when competed against [^3^H]**3**, while difluorination to give **23** reduced affinity in AD and PSP tissue when competed against [^3^H]**7** relative to **22**.

The piperidine ring of **21** was held constant while the indole and pyrimidine rings were varied to give compounds **45**, **74**, **81**, **89**, **95**, and **98** (Table S4, Supporting Information). Replacement of the pyrimidine ring of **21** with pyridine to give **45**, and movement of the fluorine atom of **45** to the pyridine ring to give **74**, generally maintained affinities in AD, PSP, and CBD tissue when competed against [^3^H]**7**, [^3^H]**8**, and [^3^H]**3**. Combining **45** and **74** to give the difluorinated compound **81** reduced affinity in AD, PSP, and CBD tissue when competed against [^3^H]**8**, whereas the difluorinated compound **89** maintained affinity in AD, PSP, and CBD tissue relative to **74** when competed against [^3^H]**8**. Moving the fluorine atom of **74** across the pyridine ring to give **95** (equivalent to removing the indole fluorine of **89**) reduced affinity in AD, PSP, and CBD tissues relative to **74** and **89** when competed against [^3^H]**8**. Flipping the fluoropyridine ring of **95** to give **98** did not change the affinities in AD, PSP, and CBD tissues when competed against [^3^H]**8**.

SAR studies were performed around the structure of **8** by holding the morpholine ring constant and changing the indole and pyrimidine rings (Table S5, Supporting Information). Replacing the pyrimidine ring of **8** with pyridine to give **46**, and then moving the fluorine atom of **46** to the 2-position of the pyridine ring to give **75**, generally maintained affinities for both **46** and **75**, relative to **8**, in AD, PSP, and CBD tissue when competed against [^3^H]**7**, [^3^H]**8**, and [^3^H]**3**. Moving the indole 6-fluoro group of **8** to the indole 5-position to give **78** also maintained binding affinity when competed against [^3^H]**8**, while moving the indole 6-fluoro group of **46** to the indole 5-position to give **79** (equivalent to changing the pyrimidine ring of **78** to pyridine) resulted in a slight improvement in affinities in AD, PSP, and CBD tissue, relative to **46** and **78**, when competed against [^3^H]**8**. Replacing the pyrimidine ring of **8** with pyrazine to give **87** maintained affinities in AD, PSP, and CBD tissue when competed against [^3^H]**8**. Moving the fluorine atom of **75** to different positions on the pyridine ring to give **94** and **96** generally maintained affinities, but moving the nitrogen atom to the adjacent position of the ring to give **103** and **105** reduced affinities when competed against [^3^H]**8**. Converting **94** to difluorinated **88**, and converting **103** to difluorinated **104** both maintained affinities, while converting **96** to difluorinated **90**, and converting **105** to difluorinated **106** both resulted in reduced affinities. *N*-methylation of **8** to give **109** reduced affinities in AD and PSP tissue when competed against [^3^H]**7**, further demonstrating the potential H-bonding role of the indole NH group.

SAR studies were also performed around the structure of **9** to give compounds **24**, **27**, **28**, and (*R*)-**43** (Table S6, Supporting Information). Replacing the pyridine ring of **9** with pyrimidine, along with exchanging the OH with F and inverting the chirality to give (*R*)-**43** resulted in a loss of affinity across all tissues and tritiated ligands relative to **9**. Changing the pyrrolidine ring substitution of (*R*)-**43** to F/OH-disubstituted to give **27** retained affinities across all tissues and tritiated ligands relative to **9**. The pyrrolidine ring of **27** is a racemic mixture of *trans*-configuration, and an individual enantiomer of **27** may have improved binding affinity and/or improved 4R- over mixed 3R/4R- and 3R-tau selectivity relative to the racemic mixture. Removing the indole fluorine atom of **27** to give **28** or replacing the indole fluorine atom of **27** with cyano to give **24** both resulted in losses of affinity in AD and PSP tissues when competed against [^3^H]**7** and [^3^H]**8**.

Compounds **21**, **27**, **45**, **46**, **74**, and **75** did not compete strongly against [^3^H]**5** (Table S7, Supporting Information) in AD, PSP, or CBD tissues, similarly to what was observed with **7** and **8**,^46^ indicating that these compounds do not bind to the site on aggregated tau where **5** binds. Compounds **21**, **45**, **46**, and **75** did not compete well against [^3^H]PiB in AD tissue (Table S7, Supporting Information) indicating that these compounds do not bind strongly to the PiB binding site on amyloid-β.^65,67^

Table 1 ranks the top 13 candidate compounds by PSP *K*_i_ values (range: 5–13 nM) when competed against [^3^H]**8**. Compound **8** has *K*_i_ values (nM) 7.7 ± 0.6 (AD), 8.5 ± 1.2 (PSP), and 11 ± 1.3 (CBD) when competed against [^3^H]**8** (Table S1, Supporting Information). Thus, a series of substituted-indole compounds have been identified with similar *K*_i_ values as **8** in AD, PSP, and CBD tissues when competed against [^3^H]**8**. All compounds have an indole NH group for hydrogen bonding which was indicated as necessary by the reduction in affinity through *N*-methylation to give **109** (Table S5, Supporting Information) or conversion to a benzofuran to give **49** (Table S2, Supporting Information). Seven of the compounds in Table 1 have fluoroindole substitution (**45**, **79**, **87**, **21**, **78**, **42**, **46**), three compounds have fluoropyridine substitution (**74**, **96**, **75**), and three compounds are difluoro-substituted (**89**, **88**, **27**). Compound **27**, as stated above, may have higher affinity as a single enantiomer, and the synthesis of each enantiomer is underway. All of these compounds can potentially be F-18 radiolabeled on the indole ring,^47−51^ the pyridine ring,^52−54^ or the pyrrolidine ring^55−60^ in the case of **27**. Compounds **74** and **75**, although not the highest affinity compounds in Table 1, represent the most easily accessible F-18 radiolabeled compounds through an [^18^F]fluoro-denitration mechanism of a 2-nitropyridine ring system,^52^ as well as a fairly simple radiolabeling precursor synthesis (compounds **71** and **72**, Scheme 4). Therefore, compounds **74** and **75** were evaluated with *in silico* prediction models to gauge whether these compounds have the potential to be successful central nervous system (CNS) PET agents.

**Table 1:**
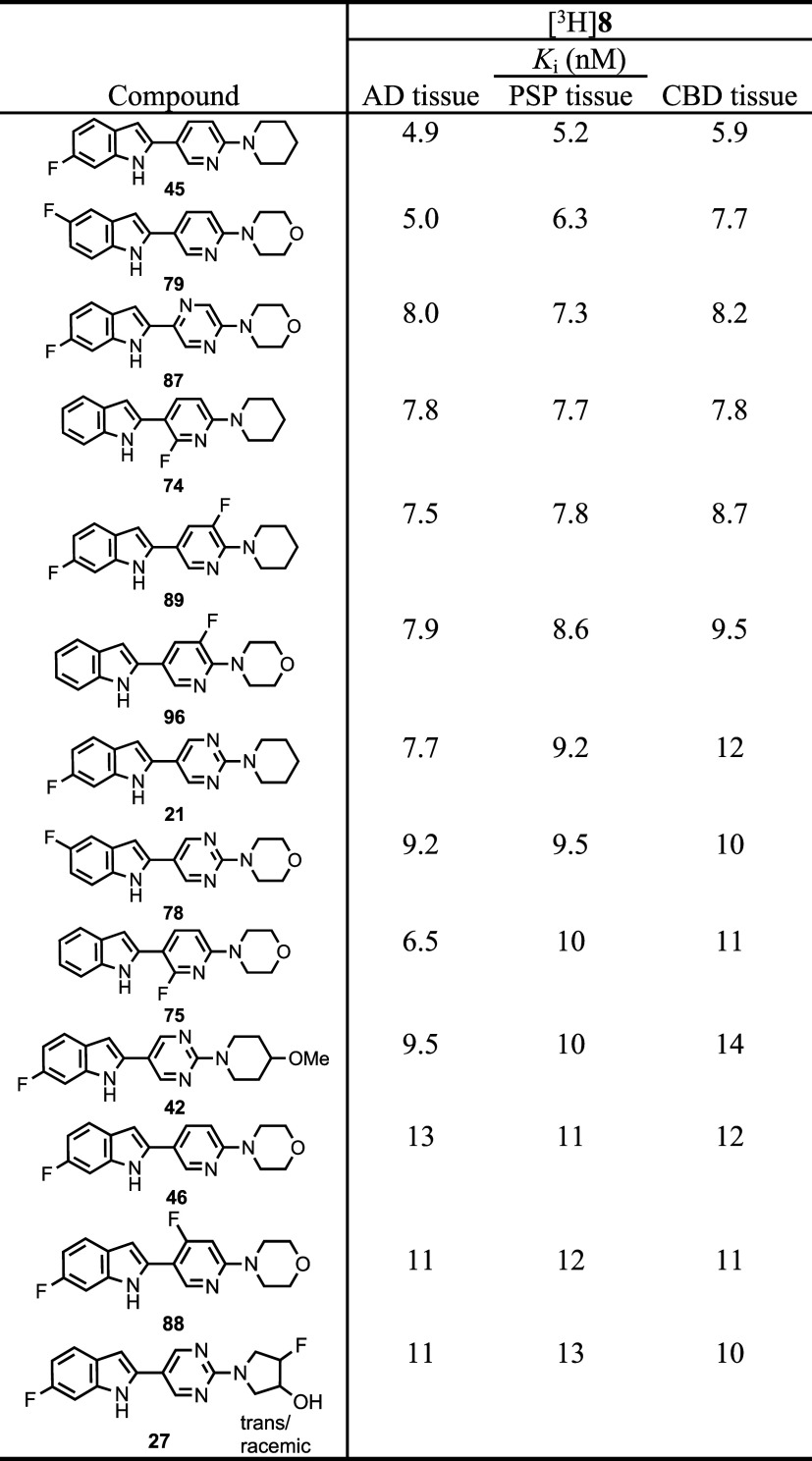
Top 13 Candidate Ligands Ranked by PSP *K*_i_ Values versus [^3^H]**8**^a^

a *n* = 1.

### In Silico CNS Exposure Predictions

Compounds **74** and **75** were evaluated with the Pfizer CNS multiparameter optimization (MPO) and PET MPO calculators,^68,69^ the blood–brain barrier (BBB) Score calculator,^70^ and the SwissADME BOILED-Egg brain penetration predictor.^71,72^ The CNS MPO and PET MPO calculators predict the likelihood of success of a CNS-targeting agent by taking into consideration physiochemical properties along with absorption, distribution, metabolism, and excretion (ADME) properties. The BBB Score estimates whether a molecule will be CNS or non-CNS active based on five physiochemical descriptors. The BOILED-Egg predictor calculates properties based on chemical structure and then creates a two-dimensional plot of WLOGP vs tPSA to predict gastrointestinal absorption (not relevant to iv-administered PET tracers) and brain permeation. These models can be used as guides for the selection of compounds with the potential to enter the CNS^73−75^ although they cannot predict other parameters that could affect the success of a candidate CNS PET tracer such as the extent of nonspecific binding and clearance rates from nontarget brain tissues.

Compound **74** (Figure S8, Supporting Information) has a CNS MPO score of 3.0 (>4 preferred, although there are several known PET tracers with CNS MPO scores <4)^73^ and a PET MPO score of 2.1 (>3 preferred) which predict a low likelihood of the compound being a successful CNS agent. But compound **74** has a BBB score of 5.01 which predicts a 90% probability that it will penetrate the BBB. Compound **75** (Figure S9, Supporting Information) has a CNS MPO score of 4.1 and a PET MPO score of 3.5 which predict that the compound will be a successful CNS agent. The BBB score for compound **75** is 4.71 which predicts that it will penetrate the BBB, but the probability is less than that of compound **74**. The BOILED-Egg plot (Figure S10, Supporting Information) predicts that both **74** and **75** will enter the brain. Thus, both compounds were advanced to radiolabeling development and PET imaging evaluation.

### Radiochemistry

**Scheme 10:**
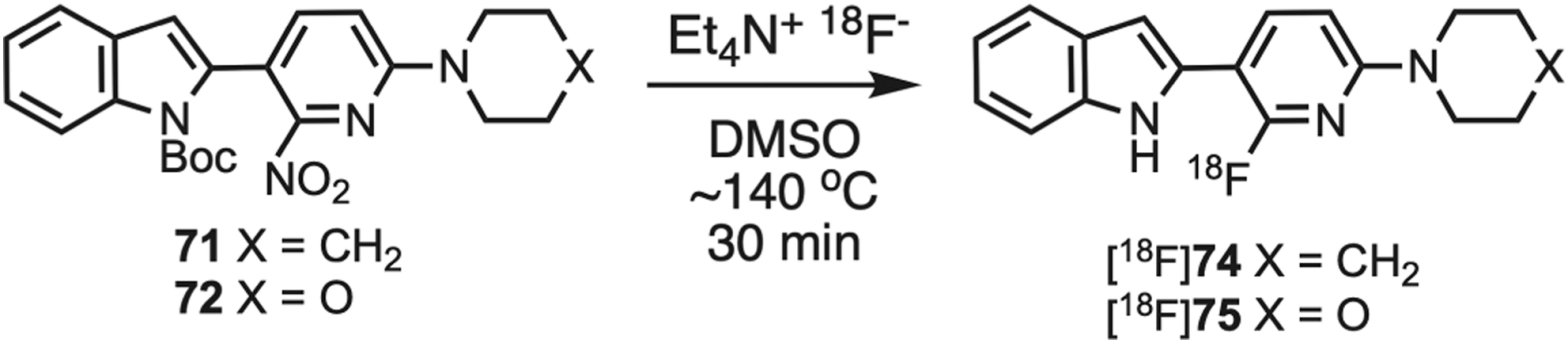
Radiolabeling of [^18^F]**74** and [^18^F]**75**

The structures of radiolabeling precursors **71** and **72** were confirmed by X-ray crystallography (Figure 3). For preliminary radiolabeling development work samples of the crude reaction mixtures were analyzed by analytical HPLC to monitor reaction progress. A sample of the crude reaction mixture of [^18^F]**74** (Figure S11, Supporting Information) demonstrated that after 45 min *N*-Boc intermediate [^18^F]**69** was not present, but *N*-deprotected [^18^F]**74** was present. Samples of the crude reaction mixture of [^18^F]**75** were taken at 15, 30, and 45 min (Figures S12–S14, Supporting Information) and showed that [^18^F]**70** was initially formed but then the Boc group was lost over time to give [^18^F]**75**, similarly to what was previously reported for [^18^F]**1**.^76,77^ For PET studies with [^18^F]**74** and [^18^F]**75**, the radiolabeling reaction (Scheme 10) was performed in dimethyl sulfoxide (DMSO) at ∼140 °C for 30 min, then the crude reaction mixture was purified by semipreparatory HPLC (Figures S15 and S16, Supporting Information). Radiochemical identity of the reformulated radiotracers was confirmed by co-injection with the nonradioactive standards (Figures S17 and S18, Supporting Information).

### Lipophilicity

The log *D*_7.4_ values of the radiolabeled compounds [^18^F]**74** and [^18^F]**75** were determined by the octanol/aqueous buffer shake-flask method using glass vials and glass pipettes, and only using plastic at the last step when a pipettor with a plastic tip was used to transfer samples to counting tubes. Initial attempts to measure the log *D*_7.4_ values by performing the study with plastic 2 mL Eppendorf tubes resulted in variable amounts of activity remaining adhered to the Eppendorf tubes. Using glass vials, the values obtained were [^18^F]**74** log *D*_7.4_ = 1.80 ± 0.01 (*n* = 4) and [^18^F]**75** log *D*_7.4_ = 1.96 ± 0.03 (*n* = 4), which are in the range (log *D* ∼ 1–5)^78^ optimal for passive diffusion across the BBB. Each of these values is less than the in silico Clog *D* predictions used for the MPO and BBB Score calculators (Figures S8 and S9, Supporting Information).

### PET Imaging Studies

**Figure 4:**
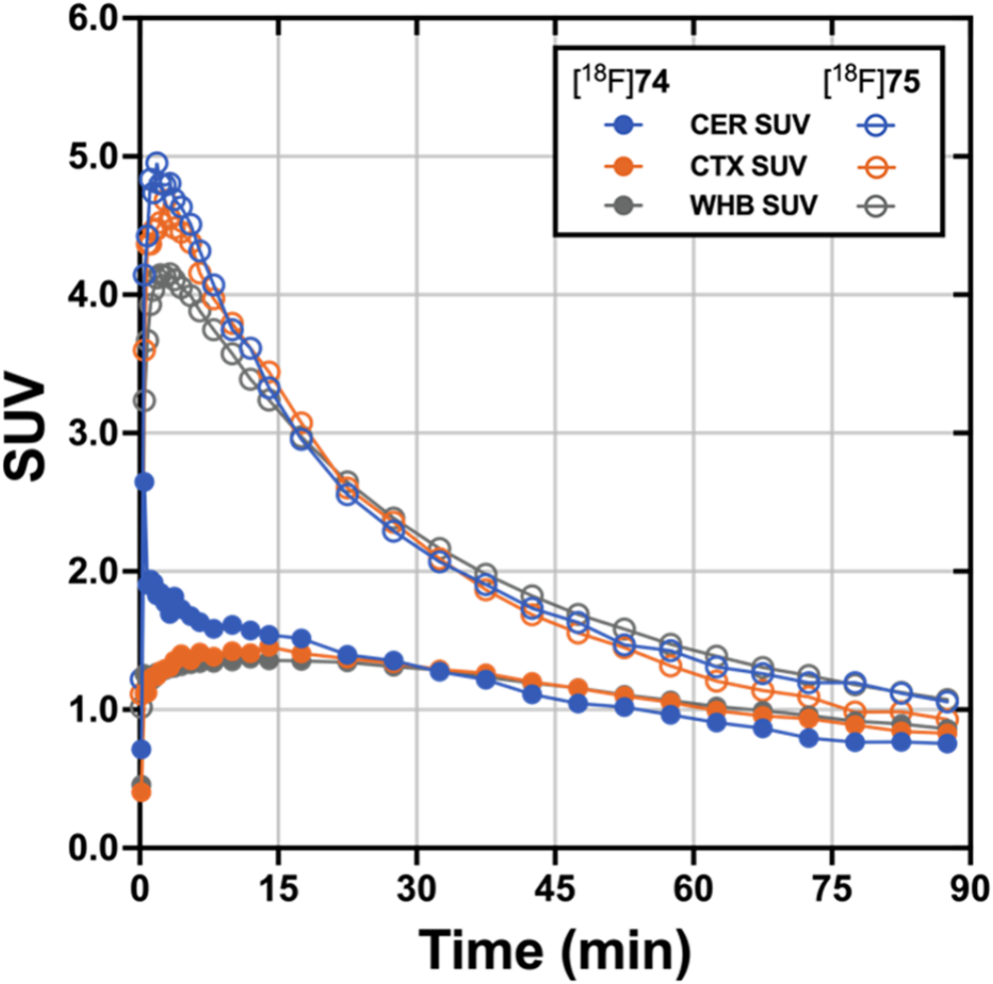
Time–activity curves of [^18^F]**74** and [^18^F]**75** in male rhesus macaques for the cerebellum, cortex, and whole brain.

**Figure 5:**
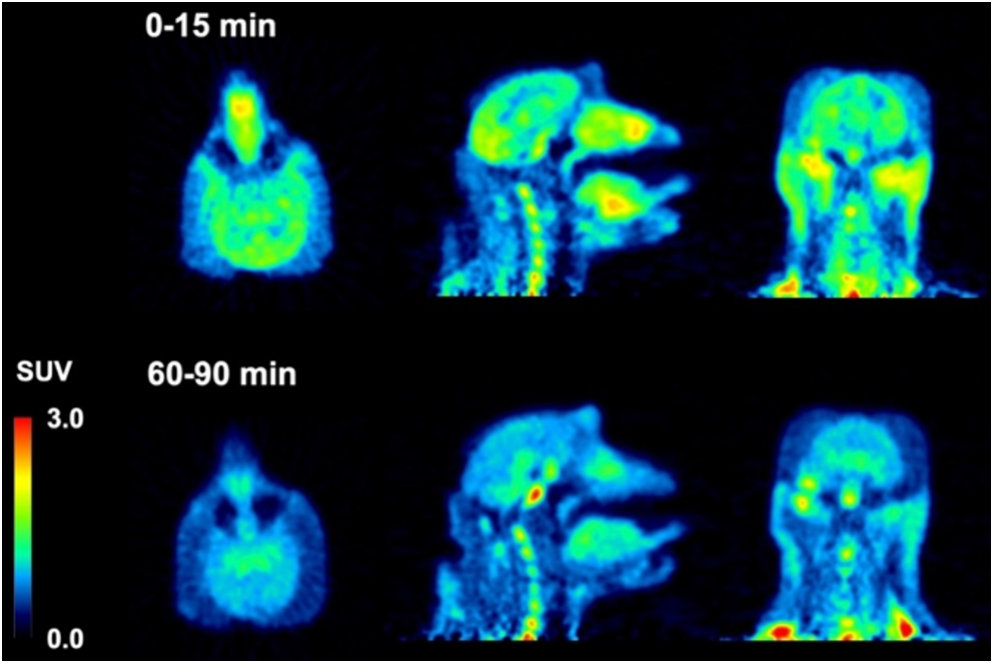
PET Images of [^18^F]**74** in a male rhesus macaque (12 kg).

Dynamic PET brain imaging studies and metabolite analysis in male rhesus macaque monkeys (*Macaca mulatta*) were performed as previously described.^45^ Evaluation of candidate tau PET tracers in nonhuman primates (NHP) that do not harbor pathological tau aggregates enables the assessment of normal brain uptake, distribution, and clearance of nonspecific binding, as well as analysis of potential radiometabolites that should more closely resemble the metabolic profile of humans compared to that obtained from rodents. Injection of [^18^F]**74** into a macaque resulted in rapid brain entry with peak standardized uptake value (SU*V*_max_) values of ∼1.5–2.5 (Figure 4 and Table 2). The earlier peak time of cerebellar radioactivity resulted in an initial period of accelerated clearance compared to cortex or whole brain, which peaked later (∼15 min postinjection). Thereafter, all regions exhibited a similar average clearance rate of ∼0.01 SUV/min. This phenomenon is common in PET studies of anesthetized primates and likely results from differential cerebral blood flow rates under isoflurane anesthesia^79^ and potentially the additive contribution of spill-in of radioactivity signal from multiple adjacent and convergent dural venous sinuses into the cerebellar region of interest. The 2-to-60 min and 2-to-90 min SUV ratios for [^18^F]**74** (Table 2) were whole brain = 1.2 and 1.5, cortex = 1.3 and 1.5, and cerebellum = 2.0 and 2.5. The summed PET images (Figure 5) indicate that from 0 to 15 min, [^18^F]**74** distributed uniformly throughout much of the brain, while the images summed from 60 to 90 min demonstrate significant clearance of brain radioactivity. The lack of skull uptake indicates that [^18^F]**74** is stable against [^18^F]defluorination and suggests that the activity in the spine evident in both early and late summed images is not the result of bone uptake of free [^18^F]fluoride. Venous metabolite analysis (Figure S19, Supporting Information) indicated that only more polar radiometabolites were formed and that ∼35% intact [^18^F]**74** remained after 10 min and ∼10% intact [^18^F]**74** remained after 30 min.

**Table 2:** Comparison of [^18^F]**74** and [^18^F]**75** SU*V*_max_, 2-to-60 min and 2-to-90 min SUV Ratios In Macaque Brain

	whole Brain			cortex			cerebellum		
	SU*V*_max_	2′:60′	2′:90′	SU*V*_max_	2′:60′	2′:90′	SU*V*_max_	2′:60′	2′:90′
[^18^F]**74**	1.4	1.2	1.5	1.5	1.3	1.5	2.7	2.0	2.5
[^18^F]**75**	4.2	3.0	3.9	4.7	3.7	4.9	5.0	3.8	4.5

**Figure 6:**
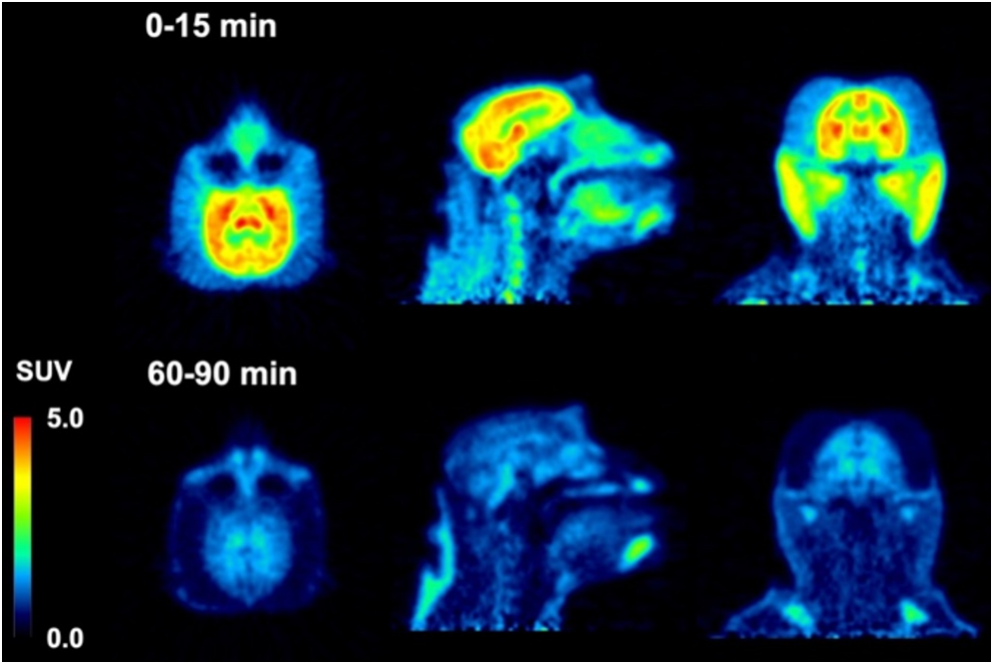
PET Images of [^18^F]**75** in a male rhesus macaque (13 kg).

Injection of [^18^F]**75** into a male macaque resulted in rapid brain entry with SU*V*_max_ values of ∼4–5 (Figure 4 and Table 2). Brain radioactivity clearance of [^18^F]**75** was more rapid on average (∼0.04 SUV/min) than we observed for [^18^F]**74**, yielding 2-to-60 min and 2-to-90 min SUV ratios (Table 2) of whole brain = 3.0 and 3.9, cortex = 3.7 and 4.9, and cerebellum = 3.8 and 4.5. The summed PET images (Figure 6) indicate that from 0 to 15 min, [^18^F]**75** distributed uniformly throughout the cortex, cerebellum, and midbrain, and then largely cleared from the brain by the 60-to-90 min image interval. Primate studies showing high brain uptake, uniform brain distribution, and rapid clearance of nonspecifically bound [^18^F]**75** suggest favorable properties for translational human studies for the visualization of pathologic tau aggregates. The lack of skull uptake indicates that [^18^F]**75** is stable against [^18^F]defluorination. Venous metabolite analysis (Figure S19, Supporting Information) indicated that only more polar radiometabolites were formed and that ∼35% intact [^18^F]**75** remained after 10 min, ∼20% intact [^18^F]**75** remained after 30 min, and ∼10% intact [^18^F]**75** remained after 90 min.

Compounds **74** and **75** differ structurally (Scheme 10) only by the replacement of a CH_2_ group with an O (piperidine changed to morpholine), and they have similar *K*_i_ values in AD, PSP, and CBD tissue samples (Table 1). Both [^18^F]**74** and [^18^F]**75** rapidly entered the brain during a nonhuman primate PET study, but [^18^F]**75** had superior uptake and clearance properties (Figure 4) as evidenced by greater 2-to-60 min and 2-to-90 min SUV ratios (Table 2). Thus, **74** was dropped from further consideration and additional studies were performed with **75**.

### Additional Evaluations of **75** and [^3^H]**75**

Compound **75** was screened at a 10-μM concentration against 98 CNS protein targets in the Eurofins CNS SafetyScreen panel (Supporting Information). Compound **75** displayed ≤25.3% inhibition at 96 targets, 44.6% inhibition at human 5-HT_2B_ (vs ± DOI), and 61% inhibition at human monoamine oxidase-B (MAO-B) (vs deprenyl). Off-target binding of some tau PET tracers to MAOs has been previously observed and is a potential concern.^12,14,80^ The binding of **75** to human recombinant MAO-B was determined by Eurofins (Figure S20, Supporting Information) and was found to be IC_50_ = 3.0 μM, which is expected to be too weak to result in off-target binding during PET imaging studies. The NHP brain PET study with [^18^F]**75** (Figures 4 and 6) demonstrated clearance of activity with no apparent retention pattern that would indicate significant off-target binding to any constitutive binding site in normal brain tissue.

Compound [^3^H]**75** was tritiated by Novandi Chemistry AB (www.novandi.se) via direct H/T exchange (37 MBq/mL (1.0 mCi/mL), *A*_m_ = 1.74 TBq/mmol (47 Ci/mmol), radiochemical purity >99%, chemical purity 99%, and stored at a reduced temperature in an EtOH + 0.01% ascorbic acid solution). The *K*_D_ and *B*_max_ values of [^3^H]**75** were determined by homologous binding assays^46,65,66,81^ with post-mortem brain tissue samples from AD, PSP, CBD, PiD, young control (CT), elderly CT, Parkinson’s disease (PD), and transactive response DNA-binding protein 43 (TDP-43)^82,83^ cases with neuropathological confirmation, as well as P301L transgenic mouse brain^84,85^ (Table 3). Compound [^3^H]**75** binds with high affinity in AD, PSP, CBD, PiD, and P301L brain tissues with *K*_D_ values of ∼1 nM and *B*_max_ values ranging from 800 to 1800 nM, while only weak binding was observed in young CT, elderly CT, PD, and TDP-43 tissues. Thus, [^3^H]**75** binds avidly to aggregated tau, but is not selective for 4R-tau (PSP, CBD, P301L) over 3R-tau (PiD) or mixed 3R/4R-tau (AD). The weak binding of [^3^H]**75** in PD tissue suggests that off-target binding to α-synuclein copathologies^86−88^ is not likely to confound tau PET imaging studies of [^18^F]**75** in human subjects, and further suggests the possibility of imaging tau in synucleinopathies.^89−92^ Additionally, the weak binding in TDP-43 tissue is important due to the possible presence of TDP-43 aggregates in PSP and CBD^93^ and a recently characterized age-related TDP-43 proteinopathy, termed limbic-predominant age-related TDP-43 encephalopathy (LATE) in subjects of advanced age (>80 y).^94^

**Table 3:** Equilibrium Dissociation Constant (*K*_D_) and Maximum Binding Density (*B*_max_) Values of [^3^H]**75** in Post-Mortem Brain Tissue Homogenates^a^

	AD	PSP	CBD	P301L	PiD
*K*_D_ (nM)	1.5 ± 0.0	1.0 ± 0.1	1.3 ± 0.2	2.6 ± 0.2	3.8 ± 0.1
*B*_max_ (nM)	1086 ± 102	766 ± 64	801 ± 58	1782 ± 64	1049 ± 55

	Young CT	Elderly CT	PD	TDP-43
*K*_D_ (nM)	50 ± 5	103 ± 11	72 ± 11	114 ± 5
*B*_max_ (nM)	3458 ± 323	6814 ± 969	9254 ± 1518	15401 ± 1023

a *n* = 3, mean ± standard deviation (SD).

The inhibition constants (*K*_i_) of the established tau tracers **1**–**4**, and the experimental tau tracers **5** and **8**, were determined versus [^3^H]**75** in post-mortem brain tissue samples from AD, PSP, CBD, and PiD (Table 4). Compounds **1**, **2**, **4**, and **5** competed poorly against [^3^H]**75** in all four tissue types. Computational studies have predicted several potential binding sites on tau fibrils^35−40^ and the inability of compounds **1**, **2**, **4**, and **5** to potently displace [^3^H]**75** demonstrates that these compounds do not bind to the same location on aggregated tau where [^3^H]**75** binds. Compound **3** had *K*_i_ values of ∼100 nM in all four tissue types demonstrating that there may be an overlap of binding sites between **3** and [^3^H]**75** on aggregated tau. Cryo-EM studies^41^ identified multiple binding sites for **3** on the Alzheimer’s tau fold, and the weak competition (*K*_i_ ∼ 86 nM) of **3** against [^3^H]**75** in AD tissue suggests that [^3^H]**75** may bind in proximity to one of the identified sites. Compound **8** had *K*_i_ values versus [^3^H]**75** of ∼15 nM in AD, PSP, and CBD tissues, and a *K*_i_ value of ∼24 nM in PiD tissue, indicating overlap of binding sites for the two structurally similar compounds in these tissues.

**Table 4:** Inhibition Constant (*K*_i_) Values of Tau Ligands versus [^3^H]**75** in AD, PSP, CBD, and PiD Brain Tissue Homogenates^a^

	AD tissue	PSP tissue	CBD tissue	PiD tissue
competitor	*K*_i_ (nM)	*K*_i_ (nM)	*K*_i_ (nM)	*K*_i_ (nM)
**1**^b^	1485 ± 53	1860 ± 39	2131 ± 58	1494 ± 26
**2**	3023 ± 283	10,000	10,000	1967 ± 96
**3**	86 ± 5	123 ± 7	106 ± 6	124 ± 3
**4**	240 ± 6	780 ± 32	888 ± 32	656 ± 36
**5**	1209 ± 47	566 ± 42	572 ± 32	575 ± 36
**8**	15 ± 1	15 ± 2	16 ± 0	24 ± 2

a *n* = 3, mean ± SD.

b +10 μM Ro-41-1040 (MAO-A inhibitor) and 10 μM deprenyl (MAO-B inhibitor).

**Figure 7:**
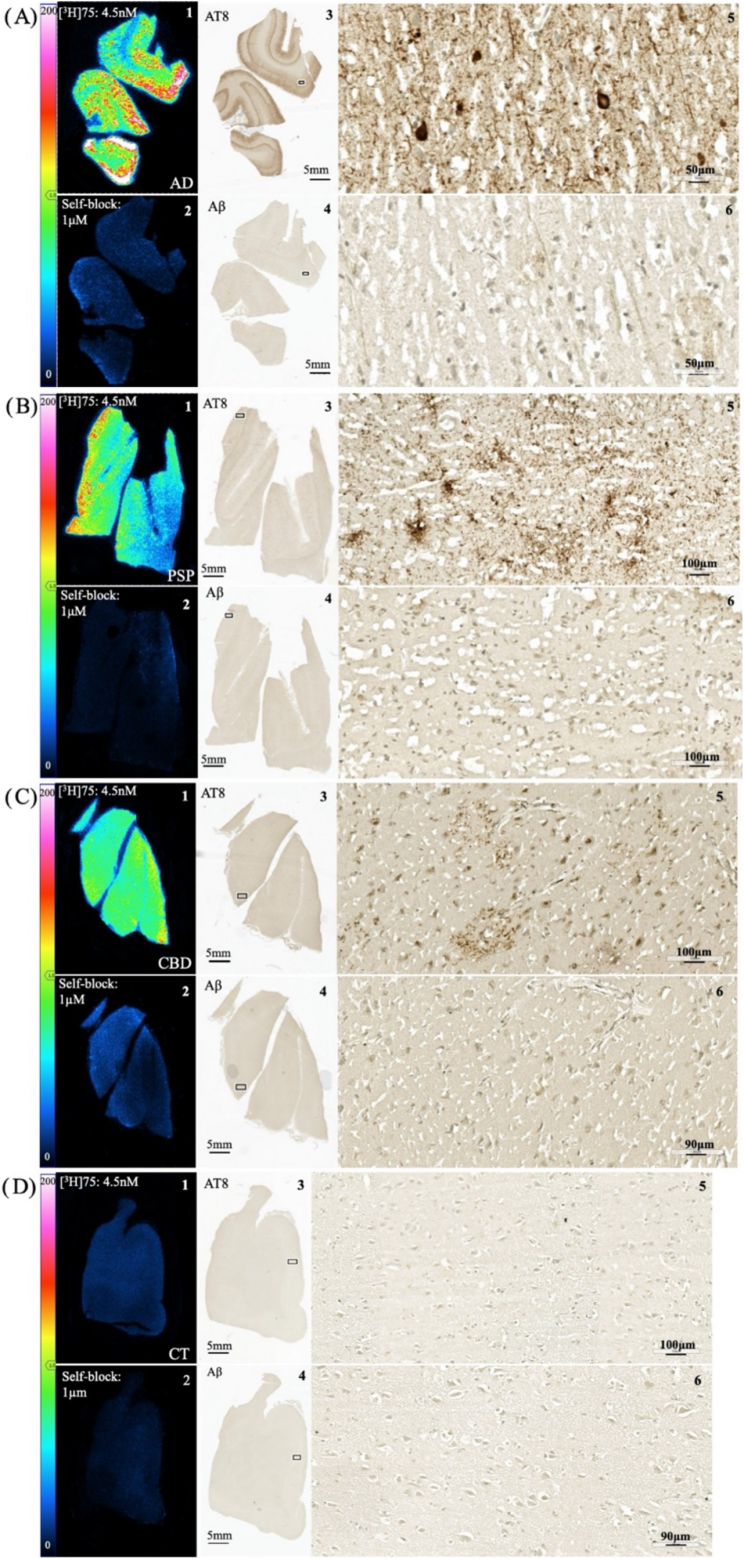
Autoradiographs showing [^3^H]**75** total binding (left upper panel; determined with 4.5 nM [^3^H]**75**) and nonspecific binding (left lower panel; determined by blocking with 1 μM **75**) of FFPE post-mortem brain sections: AD parietal cortex (A), PSP parietal cortex (B), CBD frontal cortex (C), and healthy control cingulate gyrus (D). Autoradiography color/brightness threshold levels are expressed in counts (0–200). For each case, phospho-tau (AT8 antibody) and Aβ (moC23 antibody) IHC were performed on adjacent sections as pathology reference. Magnification of AT8 and Aβ IHC are shown from areas indicated by squares. In AD brain, (A) the [^3^H]**75** spatial distribution was consistent with the laminar pattern of neurofibrillary tangles (NFTs) and neuropil threads (NTs) (A 3,5); [^3^H]**75** and AT8 IHC concordance was observed in the parietal cortex from PSP (B) and the frontal cortex from CBD (C) brain sections in areas where tufted astrocytes (PSP)(B5) and astrocytic plaques (CBD)(C5) were found. No colocalization was observed with Aβ plaques in AD, PSP, and CBD (A 4,6; B 4,6; C 4,6). Binding of [^3^H]**75** was absent in the cingulate cortex of a healthy control (D).

Autoradiography studies with [^3^H]**75** were performed using formalin-fixed, paraffin-embedded (FFPE) post-mortem brain sections from the parietal cortex of AD and PSP subjects, the frontal cortex of a CBD subject, and the cingulate gyrus of an elderly CT subject (Figure 7). A clear autoradiographic signal could be observed in AD, PSP, and CBD tissue that corresponded to phospho-tau (AT8) antibody immunohistochemistry (IHC) on adjacent tissue sections. No colocalization was observed with Aβ plaques in AD, PSP, and CBD tissue sections. Specific binding in AD, PSP, and CBD tissue was demonstrated by blocking with **75** (1 μM), while no binding was detected in elderly CT tissue.

## Summary and Conclusions

Compound library synthesis and SAR studies based upon the previously identified tau ligands **7**, **8**, and **9** (Figure 2) were performed with the goal of identifying higher affinity tau ligands that also have the potential for radiolabeling with F-18. Thirteen candidate compounds were identified with *K*_i_ values vs [^3^H]**8** in the ranges of 5–13 nM in AD and PSP tissues, and 6–14 nM in CBD tissue (Table 1), all 13 of which can potentially be radiolabeled with F-18. The synthesis of precursors for radiolabeling as a 5- or 6-[^18^F]fluoroindole is more complex due to the need to couple the center aromatic ring to the 2-position of the indole ring without affecting the 5- or 6-position of the indole ring. These syntheses are underway and will be reported in due course. Likewise, the synthesis of individual enantiomers of **27** is underway. Compounds **74** and **75** were thus chosen to be evaluated first due to the ease of radiolabeling precursor synthesis (Scheme 4) and the ease of radiolabeling a 2-[^18^F]fluoropyridine ring system (Scheme 10). Compounds [^18^F]**74** and [^18^F]**75** were each radiolabeled and evaluated in NHP brain with PET imaging to assess normal brain uptake, clearance, and retention. Both compounds rapidly entered the brain, but [^18^F]**75** showed higher brain uptake and more rapid nonspecific binding clearance (Figure 4 and Table 2). Compound **75** was determined to have low affinity for MAO-B (IC_50_ = 3.0 μM, Figure S20, Supporting Information), and no other appreciable off-target binding in the Eurofins CNS SafetyScreen panel (Supporting Information). Compound **75** also competed weakly against [^3^H]PiB in AD tissue (*K*_i_ = 61 ± 3 nM, Table S7, Supporting Information) indicating that it does not bind strongly to the PiB binding site on amyloid-β. Homologous binding assays in human post-mortem brain tissue (Table 3) demonstrated that [^3^H]**75** binds with high affinity (*K*_D_ = 1–1.5 nM) in AD (mixed 3R/4R-tau), PSP (4R-tau), and CBD (4R-tau), and with slightly reduced affinity (*K*_D_ ∼ 3.8 nM) in PiD (3R-tau). Weak binding was observed in young CT, elderly CT, PD, and TDP-43 cases. Competition binding of existing tau tracers **1**–**5** against [^3^H]**75** (Table 4) demonstrated that **1**, **2**, **4**, and **5** bind to different locations on tau aggregates than where [^3^H]**75** binds, and that the binding site of **3** has some overlap with the binding site of [^3^H]**75**. Autoradiography studies in post-mortem human brain tissue samples (Figure 7) demonstrated specific binding of [^3^H]**75** in AD, PSP, and CBD with no significant specific binding in elderly CT tissue. Therefore, [^18^F]**75** is a promising candidate for translation to human brain PET imaging studies with the potential to image tau aggregates not only in 4R-tauopathies, but also in 3R-tauopathies and mixed 3R/4R-tauopathies without the confound of differential sources of off-target binding that are observed with some of the current AD-tau PET radiopharmaceuticals.

## Methods

### General

Solvents and reagents were used as received. Tetrahydrofuran (THF) was distilled from sodium benzophenone ketyl radical. NMR spectra were obtained on Bruker Avance III spectrometers at the specified frequencies. ^1^H NMR spectra in CDCl_3_ are referenced to internal tetramethylsilane (TMS), whereas spectra in DMSO-*d*_6_, CD_3_OD, or acetone-*d*_6_ are referenced to solvent residual protons. ^13^C NMR spectra are referenced to solvent resonances. ^19^F NMR spectra are unreferenced. Radial chromatography was performed on a Harrison Research Chromatotron using silica rotors from Miles Scientific. Dry silica gel purifications were performed by placing silica gel in a medium-fritted glass filter funnel attached to a vacuum-takeoff adapter (24/40 joint), eluting under vacuum, and collecting fractions in flat-bottom boiling flasks (24/40 joint). Silica gel used was Silicycle SiliFlash P60 40–63 μm (230–400 mesh). Solvent removal was performed on a Buchi Rotary Evaporator. Nonhuman primate PET studies were conducted in accordance with the guidelines set forth by the University of Pittsburgh Institutional Animal Care and Use Committee. *In vitro* binding assays were performed as previously described.^46,65,66^ All screened compounds were >95% pure as determined by analytical HPLC (Table S8 and Figures S2–S7, Supporting Information).

### Chemistry

#### *tert*-Butyl 6-Fluoro-2-(trimethylstannyl)-1*H*-indole-1-carboxylate (**10**)

Compound **110** (2.23 g, 9.48 mmol) and Me_3_SnCl (2.09 g, 10.5 mmol, 1.1 equiv) were flushed with N_2(g)_ for 40 min, then dissolved in freshly distilled THF (100 mL) and cooled in a CH_3_CN/dry ice bath. LDA solution (2.0 M THF/heptane/ethylbenzene, 6 mL, 12 mmol, 1.3 equiv) was added dropwise over a period of 3 min, the reaction mixture was stirred at CH_3_CN/dry ice temperature for 5 min, then warmed to ambient temperature and stirred for 4 h. H_2_O (0.5 mL) was added, the mixture was stirred for 5 min, then concentrated to a dark brown oil. CH_2_Cl_2_ and hexane were added and removed to give a dark green/brown syrup/residue that was dissolved in CH_2_Cl_2_ and purified by vacuum flash chromatography on silica (15 cm h × 4 cm i.d.): % CH_2_Cl_2_/hexane–25% (200 mL), 50% (100 mL) to give a colorless syrup that slowly solidified (3.64 g). The crude product was purified twice by radial chromatography (4 mm silica): hexane (100 mL) to afford **10** (3.52 g, 93%) as a white crystalline solid: ^1^H NMR (500 MHz, CDCl_3_) δ 7.64 (dd, 1H, *J* = 10.5 Hz, *J* = 2.0 Hz), 7.41 (dd, 1H, *J* = 8.5 Hz, *J* = 5.5 Hz), 6.94 (td, 1H, *J* = 9.0 Hz, *J* = 2.0 Hz), 6.68 (t, 1H, ^3^*J*_SnH_ = 9.0 Hz), 1.70 (s, 9H), 0.30 (t, 9 H, ^2^*J*_SnH_ = 28.0 Hz); ^13^C NMR (100 MHz, CDCl_3_) δ 160.70 (d, ^1^*J*_FC_ = 237.2 Hz), 151.99, 143.73 (d, *J*_FC_ = 3.9 Hz), 137.71 (d, *J*_FC_ = 12.4 Hz), 128.72 (t, *J*_SnC_ = 21.5 Hz), 120.61 (d, *J*_FC_ = 10.0 Hz), 117.98 (t, *J*_SnC_ = 21.5 Hz), 110.66 (d, *J*_FC_ = 24.0 Hz), 102.85 (d, *J*_FC_ = 28.0 Hz), 84.71, 28.35, −7.01 (t, ^1^*J*_117SnC_ = 187.0 Hz, ^1^*J*_119SnC_ = 195.5 Hz); HRMS (ESI) [M + H]^+^ Calcd for C_16_H_23_O_2_NFSn: 400.0729, found: 400.0734.

#### *tert*-Butyl 2-(2-Chloropyrimidin-5-yl)-6-fluoro-1*H*-indole-1-carboxylate (**13**) and 2-(2-Chloropyrimidin-5-yl)-6-fluoro-1*H*-indole (**14**)

Compound **10** (0.380 g, 0.955 mmol), 5-bromo-2-chloropyrimidine (0.250 g, 1.29 mmol, 1.4 equiv), Pd(PPh_3_)_4_ (0.080 g, 0.069 mmol, 0.07 equiv), and toluene (25 mL) were stirred at reflux under N_2(g)_ for 15 h, then cooled to ambient temperature and stirred for 3 h. The reaction mixture was poured onto dry silica (55 mm h × 45 mm i.d.) and eluted under vacuum: hexane (50 mL), CH_2_Cl_2_ (100 mL), %MeOH/CH_2_Cl_2_–1% (100 mL), 2.5% (200 mL), 5% (100 mL), 10% (50 mL) to give an orange/brown residue (0.33 g). Purification by radial chromatography (2 mm silica): hexane/EtOAc/NEt_3_ v/v/v 90:8:2 (100 mL), 75:20:5 (100 mL), 50:45:5 (50 mL) afforded **13** (0.057 g, 17%) as an off-white solid, and **14** (0.030 g, 13%) as a light tan solid.

##### Compound **13**

^1^H NMR (300 MHz, CDCl_3_) δ 8.68 (s, 2H), 7.94 (dd, 1H, *J* = 10.5 Hz, *J* = 2.4 Hz), 7.53 (dd, 1H, *J* = 8.4 Hz, *J* = 5.4 Hz), 7.06 (td, 1H, *J* = 8.7 Hz, *J* = 2.4 Hz), 6.68 (d, 1H, *J* = 0.6 Hz), 1.48 (s, 9H); HRMS (ESI) [M + H]^+^ Calcd for C_17_H_16_ClFN_3_O_2_: 348.0910, found: 348.0902.

##### Compound **14**

^1^H NMR (300 MHz, acetone-*d*_6_) δ 11.10 (br s, 1H), 9.15 (s, 2H), 7.64 (dd, 1H, *J* = 8.7 Hz, *J* = 5.4 Hz), 7.19 (m–overlapping resonances, 2H), 6.91 (ddd, 1H, *J* = 9.9 Hz, *J* = 8.7 Hz, *J* = 2.1 Hz); HRMS (ESI) [M + H]^+^ Calcd for C_12_H_8_ClFN_3_: 248.0385, found: 248.0382.

#### *tert*-Butyl 2-(2-Chloropyrimidin-5-yl)-6-cyano-1*H*-indole-1-carboxylate (**15**) and 2-(2-Chloropyrimidin-5-yl)-1*H*-indole-6-carbonitrile (**16**)

1,4-Dioxane was purged with N_2(g)_ for 40 min. Compound **11** (0.460 g, 1.14 mmol), 5-bromo-2-chloropyrimidine (0.230 g, 1.19 mmol), Pd(dppf)Cl_2_ (0.078 g, 0.11 mmol, 0.09 equiv), and Na_2_CO_3_ (0.140 g, 1.32 mmol, 1.2 equiv) were flushed with N_2(g)_ for 15 min, then 1,4-dioxane (20 mL) was added. The reaction mixture was stirred at reflux under N_2(g)_ for 4 h, cooled, and the solvent was removed to give a brown residue that was dissolved in CH_2_Cl_2_, poured onto dry silica (55 mm h × 45 mm i.d.), and eluted under vacuum: CH_2_Cl_2_ (100 mL), %MeOH/CH_2_Cl_2_–1% (100 mL), 2% (100 mL), 3% (200 mL), 4% (100 mL) to give an orange/brown residue (0.160 g, crude **15**) and an orange/brown solid (0.062 g, crude **16**).

##### Compound **15**

The residue was dissolved in CH_2_Cl_2_/MeOH, poured onto dry silica (33 mm h × 33 mm i.d.), and eluted under vacuum: %MeOH/CH_2_Cl_2_–1% (100 mL), 2% (100 mL) to give a dark orange residue that was dried under vacuum briefly. The residue was dissolved/suspended in CHCl_3_ (1 mL), filtered, and the precipitate was rinsed with CHCl_3_ (1 mL × 2). The filtrate was purified by radial chromatography (1 mm silica): 75:20:5 v/v/v hexane/EtOAc/NEt_3_ (100 mL) to afford **15** (0.038 g, 9%) as a white solid: ^1^H NMR (300 MHz, CDCl_3_) δ 8.71 (s, 2H), 8.58 (d, 1H, *J* = 0.6 Hz), 7.69 (d, 1H, *J* = 8.1 Hz), 7.56 (dd, 1H, *J* = 8.1 Hz, *J* = 1.5 Hz), 6.77 (s, 1H), 1.49 (s, 9H); HRMS (ESI) [M + H]^+^ Calcd for C_18_H_16_O_2_N_4_Cl: 355.0956, found: 355.0958.

##### Compound **16**

The solid was dissolved/suspended in CHCl_3_ (2.5 mL), filtered, and the precipitate was rinsed with CHCl_3_ (2.5 mL × 3), then dried under vacuum to afford **16** (0.029 g, 10%) as a yellow/orange solid: ^1^H NMR (400 MHz, DMSO-*d*_6_) δ 12.44 (s, 1H), 9.31 (s, 2H), 7.96 (s, 1H), 7.79 (d, 1H, *J* = 8.0 Hz), 7.40 (d, 1H, *J* = 8.0 Hz), 7.36 (s, 1H); ^13^C NMR (125 MHz, DMSO-*d*_6_) δ 158.62, 156.62, 136.80, 134.87, 131.37, 125.01, 122.21, 121.53, 120.45, 116.71, 103.61, 101.88; HRMS (ESI) [M + H]^+^ Calcd for C_13_H_8_N_4_Cl: 255.0432, found: 255.0430.

#### *tert*-Butyl 2-(2-Chloropyrimidin-5-yl)-1*H*-indole-1-carboxylate (**17**)

1-Boc-indole-2-boronic acid (**12**) (0.250 g, 0.958 mmol), 5-bromo-2-chloropyrimidine (0.180 g, 0.931 mmol), and Pd(dppf)Cl_2_ (0.052 g, 0.071 mmol, 0.08 equiv) were flushed with N_2(g)_ for 10 min, then 1,4-dioxane (10 mL) was added followed by a solution of K_2_CO_3_ in H_2_O (1.5 mL, 2 M, 3.0 mmol, 3.2 equiv). The reaction mixture was stirred at reflux under N_2(g)_ for 7 h, cooled to ambient temperature, stirred overnight, then filtered through Celite, and the Celite was rinsed with 1,4-dioxane. The filtrate was concentrated to a brown oil, then CHCl_3_ and hexane were added and removed to give a brown syrup that was dissolved in CH_2_Cl_2_, poured onto dry silica (45 mm h × 45 mm i.d.) and eluted under vacuum: CH_2_Cl_2_ (200 mL), %MeOH/CH_2_Cl_2_–1% (100 mL), 2% (50 mL), 3% (50 mL) to give a tan solid (0.19 g). Purification by radial chromatography (2 mm silica): CH_2_Cl_2_ (100 mL) gave an off-white solid (0.11 g) that was again purified by radial chromatography (2 mm silica): hexane/EtOAc/NEt_3_ v/v/v 90:8:2 (35 mL), 85:12:3 (100 mL), 75:20:5 (50 mL) to afford **17** (0.082 g, 27%) as a white solid: ^1^H NMR (400 MHz, CDCl_3_) δ 8.69 (s, 2H), 8.20 (d, 1H, *J* = 8.4 Hz), 7.60 (d, 1H, *J* = 7.6 Hz), 7.40 (ddd, 1H, *J* = 8.4 Hz, *J* = 7.2 Hz, *J* = 1.2 Hz), 7.30 (ddd, 1H, *J* = 7.8 Hz, *J* = 7.2 Hz, *J* = 0.8 Hz), 6.71 (s, 1H), 1.49 (s, 9H); ^13^C NMR (125 MHz, CDCl_3_) δ 160.12, 158.61, 149.85, 137.70, 131.83, 128.95, 128.04, 125.88, 123.79, 121.23, 116.08, 113.04, 85.33, 28.11; HRMS (ESI) [M + H]^+^ Calcd for C_17_H_17_O_2_N_3_Cl: 330.1004, found: 330.1018.

#### *tert*-Butyl 6-Fluoro-2-(2-(piperidin-1-yl)pyrimidin-5-yl)-1*H*-indole-1-carboxylate (**18**)

Compound **13** (0.042 g, 0.12 mmol), piperidine (0.05 mL, 0.51 mmol, 4.2 equiv), K_2_CO_3_ (0.024 g, 0.17 mmol, 1.4 equiv), and CH_3_CN (10 mL) were stirred at reflux under N_2(g)_ for 75 min, then cooled to ambient temperature. The CH_3_CN was removed to give a residue that was dissolved in CH_2_Cl_2_, poured onto dry silica (33 mm h × 33 mm i.d.), and eluted under vacuum: CH_2_Cl_2_ (25 mL), %MeOH/CH_2_Cl_2_–1% (50 mL), 2% (75 mL), 3% (50 mL) to afford **18** (0.047 g, 98%) as a light tan solid: ^1^H NMR (500 MHz, CDCl_3_) δ 8.33 (s, 2H), 7.93 (dd, 1H, *J* = 10.5 Hz, *J* = 2.5 Hz), 7.45 (dd, 1H, *J* = 8.5 Hz, *J* = 5.5 Hz), 7.01 (td, 1H, *J* = 8.5 Hz, *J* = 2.5 Hz), 6.48 (s, 1H), 3.84 (t, 4H, *J* = 5.0 Hz), 1.71 (m, 2H), 1.62 (m, 4H), 1.48 (s, 9H); ^13^C NMR (125 MHz, CDCl_3_) δ 161.06 (d, ^1^*J*_FC_ = 238.8 Hz), 160.86, 157.36, 150.08, 137.63 (d, *J*_FC_ = 13.8 Hz), 135.95 (d, *J*_FC_ = 3.8 Hz), 125.64, 121.04 (d, *J*_FC_ = 10.0 Hz), 116.48, 111.50 (d, *J*_FC_ = 23.8 Hz), 109.95, 103.42 (d, *J*_FC_ = 28.8 Hz), 84.63, 45.24, 28.11, 25.96, 25.09; HRMS (ESI) [M + H]^+^ Calcd for C_22_H_26_O_2_N_4_F: 397.2034, found: 397.2040.

#### *tert*-Butyl 6-Fluoro-2-(2-(4-fluoropiperidin-1-yl)pyrimidin-5-yl)-1*H*-indole-1-carboxylate (**19**)

Compound **13** (0.060 g, 0.17 mmol), 4-fluoropiperidine·HCl (0.034 g, 0.24 mmol, 1.4 equiv), K_2_CO_3_ (0.088 g, 0.64 mmol, 3.7 equiv), and CH_3_CN (5 mL) were stirred at reflux under N_2(g)_ for 5 h, then cooled. The solvent was removed to give a residue that was dissolved in CH_2_Cl_2_, poured onto dry silica (33 mm h × 33 mm i.d.), and eluted under vacuum with CH_2_Cl_2_ (125 mL) to afford **19** (0.064 g, 89%) as a white foam: ^1^H NMR (300 MHz, CDCl_3_) δ 8.35 (s, 2H), 7.93 (dd, 1H, *J* = 10.8 Hz, *J* = 2.4 Hz), 7.46 (dd, 1H, *J* = 8.7 Hz, *J* = 5.7 Hz), 7.01 (td, 1H, *J* = 8.7 Hz, *J* = 2.4 Hz), 6.50 (s, 1H), 4.99 (tt, 0.5 H, *J* = 6.3 Hz, *J* = 3.3 Hz) and 4.83 (dt, 0.5 H, *J* = 9.3 Hz, *J* = 4.8 Hz) (^2^*J*_HF_ = 48.3 Hz), 3.97 (t, 4H, *J* = 5.7 Hz), 1.93 (m, 4H), 1.48 (s, 9H); HRMS (ESI) [M + H]^+^ Calcd for C_22_H_25_F_2_N_4_O_2_: 415.1940, found: 415.1944.

#### *tert*-Butyl 2-(2-(4,4-Difluoropiperidin-1-yl)pyrimidin-5-yl)-6-fluoro-1*H*-indole-1-carboxylate (**20**)

Compound **13** (0.095 g, 0.27 mmol), 4,4-difluoropiperidine·HCl (0.060 g, 0.38 mmol, 1.4 equiv), K_2_CO_3_ (0.186 g, 1.35 mmol, 5 equiv), and CH_3_CN (20 mL) were stirred at reflux under N_2(g)_ for 21 h, then cooled to ambient temperature, filtered, and the precipitate was rinsed with CH_3_CN. The CH_3_CN was removed to give an orange residue, then CH_2_Cl_2_ and hexane were added and removed to give a light orange solid that was dried under vacuum. The solid was dissolved in CH_2_Cl_2_, poured onto dry silica (45 mm h × 45 mm i.d.), and eluted under vacuum with CH_2_Cl_2_ (350 mL) to give a colorless residue. Purification by radial chromatography (2 mm silica): %CH_2_Cl_2_/hexane–25% (50 mL), 50% (125 mL) gave a white solid (0.046 g) that was purified again by radial chromatography (1 mm silica): %CH_2_Cl_2_/hexane–25% (100 mL), 35% (100 mL), 50% (50 mL) to afford **20** (0.032 g, 27%) as a white solid: ^1^H NMR (400 MHz, CDCl_3_) δ 8.37 (s, 2H), 7.93 (dd, 1H, *J* = 10.8 Hz, *J* = 2.0 Hz), 7.46 (dd, 1H, *J* = 8.4 Hz, *J* = 5.6 Hz), 7.02 (td, 1H, *J* = 8.8 Hz, *J* = 2.0 Hz), 6.51 (s, 1H), 4.03 (t, 4H, *J* = 6.0 Hz), 2.02 (septet, 4H, *J* = 6.0 Hz), 1.48 (s, 9H) HRMS (ESI) [M + H]^+^ Calcd for C_22_H_24_O_2_N_4_F_3_: 433.1846, found: 433.1836.

#### 6-Fluoro-2-(2-(piperidin-1-yl)pyrimidin-5-yl)-1*H*-indole (**21**)

Compound **18** (0.041 g, 0.10 mmol) was dissolved in trifluoroacetic acid (TFA) (1 mL, 13 mmol, 130 equiv), stirred at ambient temperature for 15 min, then poured into a mixture of NaHCO_3_ (1.23 g, 14.64 mmol, 1.1 equiv TFA), H_2_O (30 mL), and CH_2_Cl_2_ (30 mL). The mixture was stirred, the layers were separated, and the aqueous layer was extracted with CH_2_Cl_2_ (10 mL). The combined CH_2_Cl_2_ layers were washed with brine (15 mL) and dried over MgSO_4_. The solution was concentrated, poured onto dry silica (33 mm h × 33 mm i.d.), and eluted under vacuum: hexane/EtOAc/NEt_3_ v/v/v 90:8:2 (50 mL), 75:20:5 (50 mL), 50:45:5 (75 mL) to afford **21** (0.027 g, 88%) as a light yellow solid: ^1^H NMR (300 MHz, CDCl_3_) δ 8.56 (s, 2H), 8.20 (br s, 1H), 7.49 (dd, 1H, *J* = 8.7 Hz, *J* = 5.4 Hz), 7.08 (dd, 1H, *J* = 9.6 Hz, *J* = 2.1 Hz), 6.88 (ddd, 1H, *J* = 9.6 Hz, *J* = 8.7 Hz, *J* = 2.4 Hz), 6.62 (dd, 1H, *J* = 2.1 Hz, *J* = 0.9 Hz), 3.84 (t, 4H, *J* = 5.4 Hz), 1.68 (m, 6 H); ^13^C NMR (125 MHz, DMSO-*d*_6_) δ 160.08, 158.71 (d, ^1^*J*_FC_ = 234.4 Hz), 154.49, 136.68 (d, *J*_FC_ = 13.0 Hz), 134.17 (d, *J*_FC_ = 3.4 Hz), 125.44, 120.46 (d, *J*_FC_ = 10.2 Hz), 114.48, 107.73 (d, *J*_FC_ = 24.1 Hz), 97.05 (d, *J*_FC_ = 25.7 Hz), 96.91, 44.34, 25.25, 24.27; ^19^F NMR (470.6 MHz, DMSO-*d*_6_) δ −121.61 (m); HRMS (ESI) [M + H]^+^ Calcd for C_17_H_18_FN_4_: 297.1510, found: 297.1515.

#### 6-Fluoro-2-(2-(4-fluoropiperidin-1-yl)pyrimidin-5-yl)-1*H*-indole (**22**)

Compound **19** (0.059 g, 0.14 mmol) was dissolved in TFA (1.5 mL, 19.5 mmol, 139 equiv), stirred at ambient temperature for 20 min, then poured into a mixture of NaHCO_3_ (1.820 g, 21.67 mmol, 1.1 equiv TFA), H_2_O (45 mL), and CH_2_Cl_2_ (45 mL). The mixture was stirred until the color was gone, then the layers were separated, and the aqueous layer was extracted with CH_2_Cl_2_ (10 mL). The combined CH_2_Cl_2_ layers were washed with brine (20 mL) and dried over MgSO_4_. The solution was concentrated, poured onto dry silica (33 mm h × 33 mm i.d.), and eluted under vacuum: hexane/EtOAc/NEt_3_ v/v/v 90:8:2 (50 mL), 75:20:5 (50 mL), 50:45:5 (100 mL) to afford **22** (0.044 g, 99%) as a light tan solid: ^1^H NMR (300 MHz, CDCl_3_) δ 8.58 (s, 2H), 8.21 (br s, 1H), 7.50 (dd, 1H, *J* = 8.7 Hz, *J* = 5.4 Hz), 7.08 (dd, 1H, *J* = 9.6 Hz, *J* = 2.1 Hz), 6.89 (ddd, 1H, *J* = 9.6 Hz, *J* = 8.7 Hz, *J* = 2.1 Hz), 6.64 (dd, 1H, *J* = 2.1 Hz, *J* = 0.9 Hz), 5.00 (tt, 0.5 H, *J* = 6.0 Hz, *J* = 3.3 Hz) and 4.83 (dt, 0.5 H, *J* = 9.6 Hz, *J* = 4.8 Hz) (^2^*J*_HF_ = 48.3 Hz), 3.97 (m, 4H), 1.96 (m, 4 H); ^1^H NMR (300 MHz, DMSO-*d*_6_) δ 11.58 (s, 1H), 8.83 (s, 2H), 7.49 (dd, 1H, *J* = 8.7 Hz, *J* = 5.4 Hz), 7.13 (dd, 1H, *J* = 9.9 Hz, *J* = 2.1 Hz), 6.85 (ddd, 1H, *J* = 9.9 Hz, *J* = 8.7 Hz, *J* = 2.1 Hz), 6.82 (d, 1H, *J* = 1.8 Hz), 4.93 (dtt, 1H, ^2^*J*_HF_ = 48.6 Hz, *J* = 7.2 Hz, *J* = 3.6 Hz), 3.97 (m, 2H), 3.78 (m, 2H), 1.93 (m, 2H), 1.72 (m, 2H); HRMS (ESI) [M + H]^+^ Calcd for C_17_H_17_F_2_N_4_: 315.1416, found: 315.1420.

#### 2-(2-(4,4-Difluoropiperidin-1-yl)pyrimidin-5-yl)-6-fluoro-1*H*-indole (**23**)

Compound **20** (0.027 g, 0.062 mmol) was dissolved in TFA (0.6 mL, 7.8 mmol, 126 equiv), stirred at ambient temperature for 20 min, then poured into a mixture of NaHCO_3_ (0.856 g, 10.2 mmol, 1.3 equiv TFA), H_2_O (20 mL), and CH_2_Cl_2_ (20 mL). The mixture was stirred for 15 min, then the layers were separated, and the aqueous layer was extracted with CH_2_Cl_2_ (5 mL × 2). The combined CH_2_Cl_2_ layers were washed with brine (15 mL) and dried over MgSO_4_. The solution was poured onto dry silica (33 mm h × 33 mm i.d.), and eluted under vacuum: CH_2_Cl_2_ (50 mL) %MeOH/CH_2_Cl_2_–1% (50 mL), 2% (25 mL), 4% (50 mL) to afford **23** (0.018 g, 87%) as an off-white solid: ^1^H NMR (300 MHz, CDCl_3_) δ 8.60 (s, 2H), 8.23 (br s, 1H), 7.51 (dd, 1H, *J* = 8.7 Hz, *J* = 5.4 Hz), 7.08 (dd, 1H, *J* = 9.6 Hz, *J* = 2.1 Hz), 6.90 (ddd, 1H, *J* = 9.6 Hz, *J* = 8.7 Hz, *J* = 2.1 Hz), 6.66 (d, 1H, *J* = 1.5 Hz), 4.03 (t, 4H, *J* = 5.7 Hz), 2.04 (tt, 4H, ^3^*J*_HF_ = 13.5 Hz, *J* = 5.7 Hz); HRMS (ESI) [M + H]^+^ Calcd for C_17_H_16_F_3_N_4_: 333.1322, found: 333.1314.

#### 2-(2-(3-Fluoro-4-hydroxypyrrolidin-1-yl)pyrimidin-5-yl)-1*H*-indole-6-carbonitrile (**24**)

Compound **16** (0.029 g, 0.11 mmol), trans/racemic-4-fluoro-3-hydroxypyrrolidine·HCl (0.033 g, 0.23 mmol, 2 equiv), K_2_CO_3_ (0.120 g, 0.868 mmol, 7.9 equiv), and CH_3_CN (10 mL) were stirred at reflux under N_2(g)_ for 3 h, cooled, and CH_3_CN was removed to give a dark orange residue. The residue was dissolved/suspended in CH_2_Cl_2_/MeOH, filtered, and the precipitate was rinsed with CH_2_Cl_2_. The solvent was removed from the filtrate to give an orange residue that was dried under vacuum briefly (0.056 g). The residue was dissolved in CH_2_Cl_2_/MeOH, poured onto dry silica (33 mm h × 33 mm i.d.), and eluted under vacuum: %MeOH/CH_2_Cl_2_–1% (100 mL), 2.5% (100 mL), 5% (100 mL) to afford **24** (0.009 g, 24%) as a light tan solid: ^1^H NMR (300 MHz, DMSO-*d*_6_) δ 12.08 (s, 1H), 8.92 (s, 2H), 7.82 (s, 1H), 7.67 (d, 1H, *J* = 8.1 Hz), 7.33 (dd, 1H, *J* = 8.1 Hz, *J* = 1.5 Hz), 6.98 (d, 1H, *J* = 1.5 Hz), 5.61 (d, 1H, *J* = 3.9 Hz), 5.10 (dm, 1H, ^2^*J*_HF_ = 49.8 Hz), 4.37 (m, 1H), 3.88 (s, 1H), 3.77 (m, 1H), 3.69 (s, 2H), HRMS (ESI) [M – H]^+^ Calcd for C_17_H_13_FN_5_O: 322.1099, found: 322.1093.

#### *tert*-Butyl 6-Fluoro-2-(2-(3-fluoro-4-hydroxypyrrolidin-1-yl)pyrimidin-5-yl)-1*H*-indole-1-carboxylate (**25**)

Compound **13** (0.056 g, 0.16 mmol), trans/racemic-4-fluoro-3-hydroxypyrrolidine·HCl (0.052 g, 0.37 mmol, 2.3 equiv), K_2_CO_3_ (0.168 g, 1.22 mmol, 7.5 equiv), and CH_3_CN (15 mL) were stirred at reflux under N_2(g)_ for 3 h, then cooled to ambient temperature. The CH_3_CN was removed to give an off-white solid that was dissolved/suspended in CH_2_Cl_2_, filtered, and the precipitate was rinsed with CH_2_Cl_2_. The CH_2_Cl_2_ was removed from the filtrate to give a yellow syrup that was dried under vacuum (89 mg), then dissolved in CH_2_Cl_2_/MeOH, poured onto dry silica (45 mm h × 45 mm i.d.), and eluted under vacuum: %MeOH/CH_2_Cl_2_–1% (50 mL), 2% (75 mL), 3% (100 mL), 4% (50 mL). The desired fractions were combined and purified by radial chromatography (1 mm silica): %MeOH/CH_2_Cl_2_–1% (50 mL), 2% (75 mL), 3% (25 mL) to afford **25** (0.041 g, 61%) as an off-white solid: ^1^H NMR (300 MHz, CDCl_3_) δ 8.39 (s, 2H), 7.91 (dd, 1H, *J* = 10.8 Hz, *J* = 2.4 Hz), 7.46 (dd, 1H, *J* = 8.7 Hz, *J* = 5.7 Hz), 7.01 (td, 1H, *J* = 8.7 Hz, *J* = 2.4 Hz), 5.11 (dm, 1H, ^2^*J*_HF_ = 51.0 Hz), 4.59 (dm, 1H, *J* = 6.9 Hz), 4.03 (m, 1H), 3.90 (m, 3H), 1.92 (br d, 1H, *J* = 2.7 Hz), 1.50 (s, 9H).

#### *tert*-Butyl 2-(2-(3-Fluoro-4-hydroxypyrrolidin-1-yl)pyrimidin-5-yl)-1*H*-indole-1-carboxylate (**26**)

Compound **17** (0.079 g, 0.24 mmol), trans/racemic-4-fluoro-3-hydroxypyrrolidine·HCl (0.097 g, 0.69 mmol, 2.9 equiv), K_2_CO_3_ (0.158 g, 1.14 mmol, 4.7 equiv), and CH_3_CN (15 mL) were stirred at reflux under N_2(g)_ for 5 h, then cooled to ambient temperature. The CH_3_CN was removed to give a residue that was dried under vacuum briefly, then dissolved in CH_2_Cl_2_, poured onto dry silica (33 mm h × 33 mm i.d.), and eluted under vacuum: CH_2_Cl_2_ (25 mL), %MeOH/CH_2_Cl_2_–1% (50 mL), 2% (75 mL), 3% (50 mL) to afford **26** (0.085 g, 89%) as an off-white foam: ^1^H NMR (300 MHz, CDCl_3_) δ 8.41 (s, 2H), 8.17 (d, 1H, *J* = 8.4 Hz), 7.55 (d, 1H, *J* = 7.5 Hz), 7.33 (partially resolved ddd, 1H, *J* = 7.8 Hz, *J* = 1.2 Hz), 7.25 (m–partially obscured by CHCl_3_ resonance, 1H), 6.55 (s, 1H), 5.10 (dm, 1H, ^2^*J*_HF_ = 50.7 Hz), 4.59 (m, 1H), 4.03 (m, 1H), 3.91 (m, 3H), 1.89 (d, 1H, *J* = 3.9 Hz), 1.50 (s, 9H); ^13^C NMR (125 MHz, CDCl_3_) δ 161.10 (d, ^1^*J*_FC_ = 238.8 Hz), 159.49, 157.47, 150.07, 137.50 (d, *J*_FC_ = 12.5 Hz), 135.44 (d, *J*_FC_ = 3.5 Hz), 125.59, 121.21 (d, *J*_FC_ = 10.0 Hz), 117.49, 111.60 (d, *J*_FC_ = 25.0 Hz), 110.38, 103.44 (d, *J*_FC_ = 28.8 Hz), 95.00 (d, ^1^*J*_FC_ = 178.8 Hz), 84.94, 73.02 (d, *J*_FC_ = 27.5 Hz), 52.91, 51.04 (d, *J*_FC_ = 22.5 Hz), 28.13; HRMS (ESI) [M + H]^+^ Calcd for C_21_H_23_O_3_N_4_F_2_: 417.1733, found: 417.1739.

#### 4-Fluoro-1-(5-(6-fluoro-1*H*-indol-2-yl)pyrimidin-2-yl)pyrrolidin-3-ol (**27**)

Compound **25** (0.027 g, 0.065 mmol) was dissolved in TFA (0.75 mL, 9.7 mmol, 149 equiv), stirred at ambient temperature for 15 min, then poured into a mixture of NaHCO_3_ (0.910 g, 10.8 mmol, 1.1 equiv TFA), H_2_O (25 mL), and CH_2_Cl_2_ (25 mL). The mixture was stirred for 5 min, then the layers were separated, and the aqueous layer was extracted with CH_2_Cl_2_ (5 mL × 2). The combined CH_2_Cl_2_ layers were dried over MgSO_4_ and the solvent was removed to give an off-white solid that was dried under vacuum to afford **27** (0.017 g, 83%): ^1^H NMR (300 MHz, acetone-*d*_6_) δ 10.71 (br s, 1H), 8.80 (s, 2H), 7.52 (dd, 1H, *J* = 8.7 Hz, *J* = 5.4 Hz), 7.11 (dd, 1H, *J* = 9.9 Hz, *J* = 2.1 Hz), 6.84 (ddd, 1H, *J* = 9.9 Hz, *J* = 8.7 Hz, *J* = 2.4 Hz), 6.79 (m, 1H), 5.12 (dm, 1H, ^2^*J*_HF_ = 51.0 Hz), 4.67 (d, 1H, *J* = 3.9 Hz), 4.51 (m, 1H), 3.96 (d, 1H, *J* = 2.1 Hz), 3.85 (m, 1H), 3.79 (m, 2H); ^13^C NMR (125 MHz, DMSO-*d*_6_) δ 158.74 (d, ^1^*J*_FC_ = 234.6 Hz), 159.10, 154,57, 136.75 (d, *J*_FC_ = 12.6 Hz), 134.13 (d, *J*_FC_ = 3.5 Hz), 125.43, 120.51, 115.14, 107.76 (d, *J*_FC_ = 24.3 Hz), 97.09 (d, *J*_FC_ = 25.5 Hz), 97.07, 95.16 (d, *J*_FC_ = 177.0 Hz), 71.40 (d, *J*_FC_ = 27.2 Hz), 52.55, 50.71 (d, *J*_FC_ = 21.9 Hz); ^19^F NMR (470.6 MHz, DMSO-*d*_6_) δ −121.54 (m), −181.85 (m); HRMS (ESI) [M – H]^+^ Calcd for C_16_H_13_F_2_N_4_O: 315.1052, found: 315.1048.

#### 1-(5-(1*H*-Indol-2-yl)pyrimidin-2-yl)-4-fluoropyrrolidin-3-ol (**28**)

Compound **26** (0.079 g, 0.20 mmol) was dissolved in CH_2_Cl_2_ (5 mL), HCl/1,4-dioxane (4 M, 1 mL, 4 mmol) was added, the mixture was stirred for 5 h, then filtered, and the precipitate was rinsed with EtOEt (2 mL × 3). The solvent was removed from the filtrate to give a light yellow solid that was dried under vacuum briefly, and then dissolved in CH_2_Cl_2_ (10 mL). H_2_O (10 mL) was added followed by conc. NH_4_OH_(aq)_ (5 drops) and the mixture was stirred for 5 min (pH 11–12). Brine (5 mL) was added, the layers were mixed and separated, and the CH_2_Cl_2_ layer was dried over MgSO_4_. Analysis by TLC (3% MeOH/CH_2_Cl_2_) indicated *N*-Boc protected **26** remained. The solvent was removed to give a white foam that was dried under vacuum (0.050 g). The foam was dissolved in TFA (2 mL, 26 mmol), stirred for 15 min, then poured into a mixture of CH_2_Cl_2_ (40 mL), H_2_O (40 mL), and NaHCO_3_ (2.420 g, 28.81 mmol, 1.1 equiv TFA). The mixture was stirred for 15 min, then the layers were separated, and the H_2_O layer was extracted with CH_2_Cl_2_ (10 mL). The combined CH_2_Cl_2_ layers were washed with brine (25 mL), dried over MgSO_4_, and the solvent was removed to give an off-white solid (24 mg). Purification by flash column chromatography on silica (3% MeOH/CH_2_Cl_2_) afforded **28** (0.021 g, 36%) as an off-white solid: ^1^H NMR (400 MHz, DMSO-*d*_6_) δ 11.47 (s, 1H), 8.86 (s, 2H), 7.50 (d, 1H, *J* = 8.0 Hz), 7.38 (d, 1H, *J* = 8.0 Hz), 7.08 (partially resolved ddd, 1H, *J* = 7.2 Hz, *J* = 1.2 Hz), 6.99 (partially resolved ddd, 1H, *J* = 7.2 Hz, *J* = 1.2 Hz), 6.81 (d 1 H, *J* = 1.2 Hz), 5.60 (d, 1H, *J* = 3.6 Hz), 5.09 (dm, 1H, ^2^*J*_HF_ = 50.8 Hz), 4.37 (m, 1H), 3.86 (s, 1H), 3.79 (m, 1H), 3.67 (s, 2H); HRMS (ESI) [M – H]^+^ Calcd for C_16_H_14_FN_4_O: 297.1152, found: 297.1145.

#### 5-Bromo-2-(4-methoxypiperidin-1-yl)pyrimidine (**29**)

5-Bromo-2-chloropyrimidine (0.880 g, 4.55 mmol), 4-methoxypiperidine (0.65 mL, 5.3 mmol, 1.2 equiv), K_2_CO_3_ (0.890 g, 6.44 mmol, 1.4 equiv), and CH_3_CN (25 mL) were stirred at reflux under N_2(g)_ for 1 h, then cooled to ambient temperature, filtered, and the precipitate was rinsed with CH_3_CN. The CH_3_CN was removed to give a faint yellow residue that was dissolved in CH_2_Cl_2_, poured onto dry silica (55 mm h × 45 mm i.d.), and eluted under vacuum: CH_2_Cl_2_ (50 mL), %MeOH/CH_2_Cl_2_–1% (100 mL), 2% (100 mL), 3% (100 mL), 4% (100 mL) to afford **29** (1.21 g, 98%) as a white solid: ^1^H NMR (300 MHz, CDCl_3_) δ 8.27 (s, 2H), 4.19 (m, 2H), 3.44 (m, 3H), 3.39 (s, 3H), 1.92 (m, 2H), 1.57 (m, 2H); ^13^C NMR (100 MHz, CDCl_3_) δ 160.03, 158.08, 105.53, 76.35, 55.92, 41.66, 30.65; HRMS (ESI) [M + H]^+^ Calcd for C_10_H_15_ON_3_Br: 272.0393, found: 272.0398.

#### 4-(5-Bromopyrimidin-2-yl)morpholine (**30**)

5-Bromo-2-chloropyrimidine (1.48 g, 7.65 mmol), morpholine (1.0 mL, 12 mmol, 1.6 equiv), K_2_CO_3_ (1.30 g, 9.41 mmol, 1.2 equiv), and CH_3_CN (55 mL) were stirred at reflux under N_2(g)_ for 3 h, then cooled to ambient temperature, filtered, and the precipitate was rinsed with CH_3_CN. The solvent was removed from the filtrate to give a white solid that was dissolved in CH_2_Cl_2_, poured onto dry silica (55 mm h × 45 mm i.d.), and eluted under vacuum: CH_2_Cl_2_ (100 mL), %MeOH/CH_2_Cl_2_–1% (100 mL), 2% (100 mL), 3% (100 mL), 4% (100 mL) to afford **30** (1.74 g, 93%) as a white solid: ^1^H NMR (300 MHz, CDCl_3_) δ 8.31 (s, 2H), 3.76 (s, 8H); ^13^C NMR (125 MHz, CDCl_3_) δ 160.15, 158.09, 106.39, 66.85, 44.52; HRMS (ESI) [M + H]^+^ Calcd for C_8_H_11_BrN_3_O: 244.0080, found: 244.0084.

#### (*R*)-5-Bromo-2-(3-fluoropyrrolidin-1-yl)pyrimidine ((*R*)-**31**)

5-Bromo-2-chloropyrimidine (0.510 g, 2.64 mmol), (*R*)-3-fluoropyrrolidine·HCl (0.490 g, 3.90 mmol, 1.5 equiv), K_2_CO_3_ (1.13 g, 8.18 mmol, 3.1 equiv), and CH_3_CN (30 mL) were stirred at reflux under N_2(g)_ for 90 min, then cooled to ambient temperature, filtered, and the precipitate was rinsed with EtOAc 3×. The solvent was removed from the filtrate to give a white solid that was dissolved in CH_2_Cl_2_, poured onto dry silica (55 mm h × 45 mm i.d.), and eluted under vacuum: %CH_2_Cl_2_/hexane–50% (50 mL), 75% (100 mL), CH_2_Cl_2_ (200 mL), %MeOH/CH_2_Cl_2_–1% (100 mL), 2% (100 mL), 3% (100 mL), 5% (100 mL), 10% (50 mL) to afford (*R*)-**31** (0.500 g, 77%) as an off-white solid: ^1^H NMR (300 MHz, CDCl_3_) δ 8.32 (s, 2H), 5.36 (dt, 1H, ^2^*J*_HF_ = 52.8, *J* = 3.3 Hz), 3.99–3.57 (m, 4H), 2.45–1.99 (m, 2H); ^13^C NMR (100 MHz, CDCl_3_) δ 158.72, 158.18, 106.12, 92.93 (*J*_CF_ = 174.0 Hz), 53.74 (*J*_CF_ = 23.0 Hz), 44.69, 32.50 (*J*_CF_ = 21.0 Hz); HRMS (ESI) [M + H]^+^ Calcd for C_8_H_10_N_3_BrF: 246.0037, found: 246.0042.

#### 1-(5-Bromopyrimidin-2-yl)piperidin-4-ol (**32**)

5-Bromo-2-chloropyrimidine (0.970 g, 5.01 mmol), 4-hydroxy-piperidine (0.560 g, 5.54 mmol, 1.1 equiv), K_2_CO_3_ (0.920 g, 6.66 mmol, 1.3 equiv), and CH_3_CN (30 mL) were stirred at reflux under N_2(g)_ for 1 h, then cooled to ambient temperature, filtered, and the precipitate was rinsed with CH_3_CN. The CH_3_CN was removed from the filtrate to give a solid that was dissolved/suspended in CH_2_Cl_2_, poured onto dry silica (55 mm h × 45 mm i.d.), and eluted under vacuum: %MeOH/CH_2_Cl_2_–1% (100 mL), 2% (100 mL), 3% (200 mL), 5% (150 mL) to afford **32** (1.27 g, 98%) as a white solid: ^1^H NMR (300 MHz, CDCl_3_) δ 8.27 (s, 2H), 4.33 (dtd, 2 H, *J* = 13.8 Hz, *J* = 4.8 Hz, *J* = 0.9 Hz), 3.96 (apparent octet, 1H, *J* = 4.2 Hz), 3.33 (ddd, 2 H, *J* = 13.5 Hz, *J* = 9.6 Hz, *J* = 3.3 Hz), 1.94 (m, 2H), 1.53 (m, 3 H); ^13^C NMR (75 MHz, CDCl_3_) δ 160.02, 158.11, 105.66, 68.19, 41.75, 34.22; HRMS (ESI) [M + H]^+^ Calcd for C_9_H_13_ON_3_Br: 258.0237, found: 258.0242.

#### 1-(5-Bromopyrimidin-2-yl)piperidin-4-one (**33**)

Compound **32** (0.540 g, 2.09 mmol) was dissolved in CH_2_Cl_2_ (25 mL), then H_2_O (0.05 mL) was added, followed by Dess–Martin periodinane (1.03 g, 2.43 mmol, 1.2 equiv). The reaction mixture was stirred at reflux for 7 h, then cooled to ambient temperature, stirred for 17 h, and poured into a mixture of CH_2_Cl_2_ (25 mL), H_2_O (50 mL), Na_2_S_2_O_3_·5H_2_O (2.17 g, 8.74 mmol), and NaHCO_3_ (2.25 g, 26.8 mmol). The mixture was stirred for 2 h, then the layers were separated, and the H_2_O layer was extracted with CH_2_Cl_2_ (10 mL × 2). The combined CH_2_Cl_2_ layers were washed with brine (25 mL) and dried over MgSO_4_. The solution was concentrated, poured onto dry silica (55 mm h × 45 mm i.d.), and eluted under vacuum: CH_2_Cl_2_ (200 mL), %MeOH/CH_2_Cl_2_–1% (100 mL), 2.5% (100 mL), 5% (200 mL) to give an off-white solid (0.55 g). Purification by radial chromatography (4 mm silica): %MeOH/CH_2_Cl_2_–1% (100 mL), 2% (100 mL), 4% (50 mL), 5% (100 mL) afforded recovered **32** (0.110 g, 20%) and **33** (0.380 g, 71%) as a white solid: ^1^H NMR (300 MHz, CDCl_3_) δ 8.35 (s, 2H), 4.10 (t, 4H, *J* = 6.3 Hz), 2.50 (t, 4H, *J* = 6.3 Hz); ^13^C NMR (125 MHz, CDCl_3_) δ 208.14, 159.69, 158.42, 107.07, 43.67, 41.04; HRMS (ESI) [M + H]^+^ Calcd for C_9_H_11_BrN_3_O: 256.0080; found: 256.0092.

#### 5-Bromo-2-(piperidin-1-yl)pyridine (**34**)

2,5-Dibromopyridine (0.870 g, 3.67 mmol) and piperidine (5.0 mL, 51 mmol, 14 equiv) were stirred under N_2(g)_ in a heated sand bath (105 °C) for 15 h, then cooled to ambient temperature. EtOAc (5 mL) was added, the mixture was filtered, and the precipitate was rinsed with EtOAc (5 mL × 2). The filtrate was concentrated to an oil/residue, then dissolved in CH_2_Cl_2_, poured onto dry silica (55 mm h × 45 mm i.d.), and eluted under vacuum: hexane (50 mL), hexane/EtOAc/NEt_3_ v/v/v 90:8:2 (100 mL), 75:20:5 (100 mL) to afford **34** (0.860 g, 97%) as a colorless oil: ^1^H NMR (300 MHz, CDCl_3_) δ 8.16 (d, 1H, *J* = 2.4 Hz), 7.48 (dd, 1H, *J* = 9.3 Hz, *J* = 2.4 Hz), 6.54 (d, 1H, *J* = 9.3 Hz), 3.50 (m, 4H), 1.63 (m, 6 H); ^13^C NMR (100 MHz, CDCl_3_) δ 158.35, 148.67, 139.73, 108.61, 106.78, 46.55, 25.59, 24.81; HRMS (ESI) [M + H]^+^ Calcd for C_10_H_14_BrN_2_: 241.0335, found: 241.0339.

#### 4-(5-Bromopyridin-2-yl)morpholine (**35**)

2,5-Dibromopyridine (0.870 g, 3.67 mmol) and morpholine (5.0 mL, 58 mmol, 16 equiv) were stirred under N_2(g)_ in a heated sand bath (∼110 °C) for 15 h, then cooled to ambient temperature. EtOAc (5 mL) was added, the mixture was filtered, and the precipitate was rinsed with EtOAc (5 mL × 2). The filtrate was concentrated, diluted with hexane, poured onto dry silica (55 mm h × 45 mm i.d.), and eluted under vacuum: hexane/EtOAc/NEt_3_ v/v/v 90:8:2 (100 mL), 75:20:5 (200 mL) to afford **35** (0.860 g, 96%) as an off-white solid: ^1^H NMR (300 MHz, CDCl_3_) δ 8.21 (d, 1H, *J* = 2.4 Hz), 7.56 (dd, 1H, *J* = 9.0 Hz, *J* = 2.4 Hz), 6.53 (d, 1H, *J* = 9.0 Hz), 3.81 (t, 4H, *J* = 4.8 Hz), 3.47 (t, 4H, *J* = 4.8 Hz); ^13^C NMR (125 MHz, CDCl_3_) δ 158.27, 148.74, 139.97, 108.41, 66.79, 45.71; HRMS (ESI) [M + H]^+^ Calcd for C_9_H_12_ON_2_Br: 243.0128, found: 243.0139.

#### 4-(5-Bromopyrazin-2-yl)morpholine (**36**)

Morpholine (0.3 mL, 3.5 mmol) was added to a suspension of NaH (0.087 g, 90%, 3.26 mmol) in THF (6 mL) at 0 °C under N_2(g)_, the mixture was stirred for 15 min, then 2,5-dibromopyrazine (0.806 g, 3.39 mmol) in THF (6 mL) was added, and the reaction mixture was stirred at reflux overnight. The solvent was evaporated, and the residue was dissolved in EtOAc (20 mL), washed with H_2_O (10 mL), and dried over MgSO_4_. The solvent was removed and the residue was purified by flash column chromatography (4:1 v/v hexanes/EtOAc) to afford **36** as a white solid (0.858 g, quantitative): ^1^H NMR (CDCl_3_, 500 MHz): δ 8.16 (d, 1H, *J* = 2.0 Hz), 7.86 (d, 1H, *J* = 2.5 Hz), 3.83 (apparent t, 4H, *J* = 8.0 Hz), 3.53 (apparent t, 4H, *J* = 8.0 Hz). HRMS (ESI) [M + H]^+^ Calcd for C_8_H_11_ON_3_Br: 244.0080, found: 244.0076.

#### *tert*-Butyl 6-Fluoro-2-(2-(4-methoxypiperidin-1-yl)pyrimidin-5-yl)-1*H*-indole-1-carboxylate (**37**)

Compound **10** (0.650 g, 1.63 mmol), compound **29** (0.490 g, 1.80 mmol, 1.1 equiv), Pd(dppf)Cl_2_ (0.110 g, 0.150 mmol, 0.09 equiv), and Na_2_CO_3_ (0.560 g, 5.28 mmol, 3.2 equiv) were flushed with N_2(g)_ for 10 min, then 1,4-dioxane (45 mL) was added. The reaction mixture was stirred at reflux under N_2(g)_ for 15 h, then cooled to ambient temperature, filtered through Celite, and the Celite was rinsed with 1,4-dioxane. The filtrate was concentrated to a brown syrup that was dried under vacuum, then dissolved in CH_2_Cl_2_, poured onto dry silica (55 mm h × 45 mm i.d.), and eluted under vacuum: CH_2_Cl_2_ (100 mL), %MeOH/CH_2_Cl_2_–1% (100 mL), 2.5% (100 mL), 5% (150 mL) to give a brown residue (0.65 g). The residue was dissolved in CH_2_Cl_2_, poured onto dry silica (55 mm h × 45 mm i.d.), and eluted under vacuum: hexane (25 mL), hexane/EtOAc/NEt_3_ v/v/v 75:20:5 (100 mL), 50:45:5 (100 mL), 20:75:5 (150 mL) to give a dark orange solid (0.520 g). Purification by radial chromatography (2 mm silica): CH_2_Cl_2_ (100 mL), 1% MeOH/CH_2_Cl_2_ (50 mL) gave crude **37** (0.300 g, light brown syrup) and crude **42** (0.091 g, light brown residue–further purified below).

Crude **37** was purified by radial chromatography (2 mm silica): hexane/EtOAc/NEt_3_ v/v/v 95:4:1 (100 mL), 90:8:2 (25 mL) to give a sticky, faint yellow residue that was again purified by radial chromatography (2 mm silica): CHCl_3_ (50 mL), %EtOH/CHCl_3_–1% (50 mL), 2% (25 mL) to afford **37** (0.260 g, 37%) as a faint yellow viscous syrup that slowly solidified: ^1^H NMR (300 MHz, CDCl_3_) δ 8.34 (s, 2H), 7.93 (dd, 1H, *J* = 10.8 Hz, *J* = 2.4 Hz), 7.45 (dd, 1H, *J* = 8.7 Hz, *J* = 5.4 Hz), 7.01 (td, 1H, *J* = 8.7 Hz, *J* = 2.4 Hz), 6.49 (s, 1H), 4.34 (m, 2H), 3.48 (m, 3H), 3.41 (s, 3H), 1.96 (m, 2H), 1.59 (m, 2H), 1.47 (s, 9H); HRMS (ESI) [M + H]^+^ Calcd for C_23_H_28_FN_4_O_3_: 427.2140, found: 427.2131.

#### *tert*-Butyl (*R*)-6-Fluoro-2-(2-(3-fluoropyrrolidin-1-yl)pyrimidin-5-yl)-1*H*-indole-1-carboxylate ((*R*)-**38**)

Compound **10** (0.400 g, 1.00 mmol), compound (*R*)-**31** (0.300 g, 1.22 mmol, 1.2 equiv), Pd(dppf)Cl_2_ (0.076 g, 1.1 mmol, 0.1 equiv), Na_2_CO_3_ (0.170 g, 1.60 mmol, 1.6 equiv), and 1,4-dioxane (25 mL) were stirred at reflux under N_2(g)_ for 6 h, then cooled to ambient temperature. The reaction mixture was filtered through Celite, the Celite was rinsed with EtOAC, the filtrate was concentrated to a brown syrup, then CH_2_Cl_2_ and hexane were added and removed to give a brown residue that was dried under vacuum. The residue was dissolved in CH_2_Cl_2_ (+ a few drops MeOH), poured onto dry silica (55 mm h × 45 mm i.d.), and eluted under vacuum: CH_2_Cl_2_ (100 mL), %MeOH/CH_2_Cl_2_–1% (100 mL), 2.5% (100 mL), 5% (200 mL) to give a dark orange/brown residue (0.370 g). The residue was dissolved in CH_2_Cl_2_/MeOH and purified by radial chromatography (2 mm silica): hexane/EtOAc/NEt_3_ v/v/v 90:8:2 (100 mL) to give a light yellow solid (0.170 g) that was purified again by radial chromatography (2 mm silica): hexane/EtOAc/NEt_3_ v/v/v 90:8:2 (100 mL), 75:20:5 (100 mL) to give a light yellow solid (0.140 g). A third purification by radial chromatography (2 mm silica): CHCl_3_ (150 mL), 1% EtOH/CHCl_3_ (50 mL) afforded (*R*)-**38** (0.130 g, 32%) as an off-white solid: ^1^H NMR (400 MHz, CDCl_3_) δ 8.39 (s, 2H), 7.92 (dd, 1H, *J* = 10.6 Hz, *J* = 2.4 Hz), 7.46 (dd, 1H, *J* = 8.8 Hz, *J* = 5.4 Hz), 7.01 (td, 1H, *J* = 8.8 Hz, *J* = 2.4 Hz), 6.51 (s, 1H), 5.40 (dt, 1H, ^2^*J*_HF_ = 52.8 Hz, *J* = 3.2 Hz), 4.03 (ddd, 1H, ^3^*J*_HF_ = 25.2 Hz, *J* = 13.6 Hz, *J* = 1.6 Hz), 3.93 (t, 1H, *J* = 10.0 Hz), 3.81 (dd, 0.5 H, *J* = 13.6 Hz, *J* = 3.6 Hz), 3.76–3.69 (m–overlapping resonances, 1.5 H), 2.46–2.37 (m, 1H), 2.26–2.06 (m, 1H), 1.49 (s, 9H); HRMS (ESI) [M + H]^+^ Calcd for C_21_H_23_O_2_N_4_F_2_: 401.1784, found: 401.1774.

#### *tert*-Butyl 6-Fluoro-2-(2-(4-oxopiperidin-1-yl)pyrimidin-5-yl)-1*H*-indole-1-carboxylate (**39**)

1,4-Dioxane was bubbled with N_2(g)_ for 30 min. Compound **10** (0.240 g, 0.603 mmol), compound **33** (0.180 g, 0.703 mmol, 1.2 equiv), Pd(dppf)Cl_2_ (0.047 g, 0.064 mmol, 0.1 equiv), and Na_2_CO_3_ (0.140 g, 1.32 mmol, 2.2 equiv) were flushed with N_2(g)_ for 10 min, then 1,4-dioxane (25 mL) was added, the mixture was stirred at reflux under N_2(g)_ for 5 h, then cooled to ambient temperature. The mixture was filtered through Celite, and the Celite was rinsed with EtOAc. The filtrate was concentrated to a dark orange oil, then CH_2_Cl_2_ and hexane were added and removed to give a dark orange residue that was dissolved in CH_2_Cl_2_, poured onto dry silica (55 mm h × 45 mm i.d.), and eluted under vacuum: hexane/EtOAc/NEt_3_ v/v/v 90:8:2 (100 mL), 75:20:5 (100 mL), 50:45:5 (200 mL), 20:75:5 (100 mL) to give a light orange solid (0.134 g). Purification by radial chromatography (2 mm silica): hexane/EtOAc/NEt_3_ v/v/v 90:8:2 (100 mL), 75:20:5 (100 mL) gave an off-white solid (0.079 g) that was again purified by radial chromatography (2 mm silica): %MeOH/CH_2_Cl_2_–1% (50 mL), 2% (50 mL) to afford **39** (0.070 g, 28%) as an off-white solid: ^1^H NMR (300 MHz, CDCl_3_) δ 8.42 (s, 2H), 7.93 (dd, 1H, *J* = 10.8 Hz, *J* = 2.4 Hz), 7.47 (dd, 1H, *J* = 8.7 Hz, *J* = 5.4 Hz), 7.02 (td, 1H, *J* = 8.7 Hz, *J* = 2.4 Hz), 6.53 (s, 1H), 4.20 (t, 4H, *J* = 6.3 Hz), 2.54 (t, 4H, *J* = 6.3 Hz), 1.49 (s, 9H); HRMS (ESI) [M + H]^+^ Calcd for C_22_H_24_O_3_N_4_F: 411.1827, found: 411.1815.

#### *tert*-Butyl 6-Fluoro-2-(6-(piperidin-1-yl)pyridin-3-yl)-1*H*-indole-1-carboxylate (**40**)

1,4-Dioxane (30 mL) was purged with N_2(g)_ for 30 min. Compound **10** (0.440 g, 1.11 mmol), compound **34** (0.290 g, 1.20 mmol, 1.1 equiv), Pd(dppf)Cl_2_ (0.100 g, 0.140 mmol, 0.1 equiv), and Na_2_CO_3_ (0.540 g, 5.09 mmol, 4.6 equiv) were flushed with N_2(g)_ for 10 min, then 1,4-dioxane was added. The reaction mixture was stirred at reflux under N_2(g)_ for 5 h, then cooled to ambient temperature, filtered through Celite, and the Celite was rinsed with EtOAc. The solvent was removed from the filtrate to give a brown oil, then CH_2_Cl_2_ and hexane were added and removed to give a brown residue that was dried under vacuum. The residue was dissolved in CH_2_Cl_2_, poured onto dry silica (55 mm h × 45 mm i.d.), and eluted under vacuum: %CH_2_Cl_2_/hexane–50% (100 mL), 75% (100 mL), CH_2_Cl_2_ (200 mL), %MeOH/CH_2_Cl_2_–1% (100 mL), 2.5% (100 mL), 5% (100 mL) to give recovered **10** (0.220 g crude, subsequently purified to give 0.094 g, 21% recovery) and a brown residue (0.310 g). The brown residue was dissolved in CH_2_Cl_2_, poured onto dry silica (55 mm h × 45 mm i.d.), and eluted under vacuum: CH_2_Cl_2_ (100 mL), %MeOH/CH_2_Cl_2_–1% (100 mL), 2% (100 mL), 3% (200 mL), 5% (200 mL) to give a brown residue (0.270 g) that was purified by radial chromatography (2 mm silica): CHCl_3_ (100 mL) to give a light brown solid (0.120 g). Purification by radial chromatography (2 mm silica): hexane/EtOAc/NEt_3_ v/v/v 90:8:2 (100 mL), 75:20:5 (25 mL) afforded **40** (0.089 g, 20%) as an off-white solid: ^1^H NMR (300 MHz, CDCl_3_) δ 8.22 (d, 1H, *J* = 2.4 Hz), 7.92 (dd, 1H, *J* = 10.8 Hz, *J* = 2.4 Hz), 7.45 (m, 2H), 6.99 (td, 1H, *J* = 8.7 Hz, 2.4 Hz), 6.67 (d, 1H, *J* = 8.7 Hz), 6.46 (s, 1H), 3.59 (m, 4H), 1.67 (m, 6H), 1.43 (s, 9H); HRMS (ESI) [M + H]^+^ Calcd for C_23_H_27_FN_3_O_2_: 396.2082, found: 396.2081.

#### *tert*-Butyl 6-Fluoro-2-(6-morpholinopyridin-3-yl)-1*H*-indole-1-carboxylate (**41**)

Compound **10** (0.410 g, 1.03 mmol), compound **35** (0.260 g, 1.07 mmol, 1 equiv), Pd(dppf)Cl_2_ (0.081 g, 0.11 mmol, 0.1 equiv), Na_2_CO_3_ (0.320 g, 3.02 mmol, 2.9 equiv) and 1,4-dioxane (25 mL) were stirred at reflux under N_2(g)_ for 6 h, then cooled to ambient temperature. The reaction mixture was filtered through Celite, the Celite was rinsed with EtOAC, the filtrate was concentrated to a brown oil, then CH_2_Cl_2_ and hexane were added and removed to give a brown residue that was dried under vacuum. The residue was dissolved in CH_2_Cl_2_, poured onto dry silica (55 mm h × 45 mm i.d.), and eluted under vacuum: hexane/EtOAc/NEt_3_ v/v/v 90:8:2 (125 mL), 75:20:5 (100 mL), 50:45: (150 mL), 20:75:5 (200 mL) to give a yellow syrup (0.14 g). Purification by radial chromatography (2 mm silica): hexane/EtOAc/NEt_3_ v/v/v 90:8:2 (125 mL), 75:20:5 (100 mL) afforded **41** (0.066 g, 16%) as a light yellow foam: ^1^H NMR (400 MHz, CDCl_3_) δ 8.26 (d, 1H, *J* = 2.0 Hz), 7.91 (dd, 1H, *J* = 10.6 Hz, *J* = 2.0 Hz), 7.54 (dd, 1H, *J* = 8.8 Hz, *J* = 2.4 Hz), 7.45 (dd, 1H, *J* = 8.8 Hz, *J* = 5.4 Hz), 7.00 (td, 1H, *J* = 8.8 Hz, *J* = 2.4 Hz), 6.67 (d, 1H, *J* = 8.8 Hz), 6.48 (s, 1H), 3.85 (t, 4H, *J* = 4.8 Hz), 3.56 (t, 4H, *J* = 4.8 Hz), 1.44 (s, 9H); HRMS (ESI) [M + H]^+^ Calcd for C_22_H_25_FN_3_O_3_: 398.1875, found: 398.1866.

#### 6-Fluoro-2-(2-(4-methoxypiperidin-1-yl)pyrimidin-5-yl)-1*H*-indole (**42**)

Crude **42** (0.091 g–from coupling reaction of **37** above) was purified by radial chromatography (1 mm silica): CH_2_Cl_2_ (50 mL), %MeOH/CH_2_Cl_2_–1% (50 mL), 2% (75 mL) to afford **42** (0.046 g, 9%) as a tan solid: ^1^H NMR (300 MHz, CDCl_3_) δ 8.57 (s, 2H), 8.23 (br s, 1H), 7.50 (dd, 1H, *J* = 8.7 Hz, *J* = 5.4 Hz), 7.08 (dd, 1H, *J* = 9.6 Hz, *J* = 2.1 Hz), 6.89 (ddd, 1H, *J* = 9.7 Hz, *J* = 8.7 Hz, *J* = 2.4 Hz), 6.63 (d, 1H, *J* = 1.5 Hz), 4.31 (m, 2H), 3.50 (m, 3H), 3.41 (s, 3H), 1.97 (m, 2H), 1.62 (m, 2H); ^13^C NMR (125 MHz, DMSO-*d*_6_) δ 160.04, 158.74 (d, ^1^*J*_FC_ = 234.4 Hz), 154.52, 136.70 (d, *J*_FC_ = 12.7 Hz), 134.06 (d, *J*_FC_ = 3.5 Hz), 125.42, 120.51 (d, *J*_FC_ = 10.2 Hz), 114.81, 107.76 (d, *J*_FC_ = 24.4 Hz), 97.08 (d, *J*_FC_ = 25.7 Hz), 97.05, 75.49, 54.97, 40.97, 30.20; ^19^F NMR (470.6 MHz, DMSO-*d*_6_) δ −121.53 (m).

##### Compound **42** from *N*-Boc Deprotection of **37**

Compound **37** (0.065 g, 0.15 mmol) and TFA (1.5 mL, 20 mmol, 129 equiv) were stirred at ambient temperature for 15 min, then poured into a mixture of NaHCO_3_ (1.84 g, 21.9 mmol, 1.1 equiv TFA), H_2_O (45 mL), and CH_2_Cl_2_ (45 mL). The mixture was stirred, then the layers were separated, and the H_2_O layer was extracted with CH_2_Cl_2_ (10 mL). The combined CH_2_Cl_2_ layers were washed with brine (20 mL) and dried over MgSO_4_. The solution was concentrated, poured onto dry silica (33 mm h × 33 mm i.d.), and eluted under vacuum: hexane/EtOAc/NEt_3_ v/v/v 90:8:2 (50 mL), 75:20:5 (50 mL), 50:45:5 (100 mL) to afford **42** (0.030 g, 61%) as a light yellow solid: ^1^H NMR (300 MHz, CDCl_3_) δ 8.57 (s, 2H), 8.24 (br s, 1H), 7.49 (dd, 1H, *J* = 8.7 Hz, *J* = 5.4 Hz), 7.07 (dd, 1H, *J* = 9.3 Hz, *J* = 2.1 Hz), 6.89 (ddd, 1H, *J* = 9.6 Hz, *J* = 8.7 Hz, *J* = 2.4 Hz), 6.62 (partially resolved dd, 1H, *J* = 2.1 Hz, *J* = 0.6 Hz), 4.31 (m, 2H), 3.50 (m, 3H), 3.41 (s, 3H), 1.97 (m, 2H), 1.62 (m, 2H); HRMS (ESI) [M + H]^+^ Calcd for C_18_H_20_FN_4_O: 327.1616, found: 327.1620.

#### (*R*)-6-Fluoro-2-(2-(3-fluoropyrrolidin-1-yl)pyrimidin-5-yl)-1*H*-indole ((*R*)-**43**)

Compound (*R*)-**38** (0.120 g, 0.300 mmol) was dissolved in TFA (3 mL, 39 mmol, 130 equiv), stirred at ambient temperature for 20 min, then poured into a mixture of NaHCO_3_ (3.95 g, 47.0 mmol, 1.2 equiv TFA), H_2_O (75 mL), and CH_2_Cl_2_ (75 mL). The mixture was stirred for 20 min, then the layers were separated, and the aqueous layer was extracted with CH_2_Cl_2_ (25 mL × 2). The combined CH_2_Cl_2_ layers were washed with brine (25 mL), dried over MgSO_4_, and concentrated (precipitate formed). MeOH was added to redissolve the precipitate, then the solution was poured onto dry silica (55 mm h × 45 mm i.d.) and eluted under vacuum: %MeOH/CH_2_Cl_2_–1% (100 mL), 2.5% (100 mL), 5% (200 mL), 10% (50 mL) to give a yellow solid (85 mg). The solid was placed in a medium-fritted filter, rinsed with 1:1 v/v CH_2_Cl_2_/hexane (1 mL × 7), and dried under vacuum to afford (*R*)-**43** (0.067 g, 74%) as a light yellow solid: ^1^H NMR (300 MHz, acetone-d_6_) δ 10.71 (br s, 1H), 8.80 (s, 2H), 7.52 (dd, 1H, *J* = 8.4 Hz, *J* = 5.4 Hz), 7.10 (dd, 1H, *J* = 9.9 Hz, *J* = 2.1 Hz), 6.84 (ddd, 1H, *J* = 9.9 Hz, *J* = 8.7 Hz, *J* = 2.4 Hz), 6.79 (s, 1H), 5.45 (dm, 1H, ^2^*J*_HF_ = 52.8 Hz), 4.01–3.58 (m, 4H), 2.37–2.12 (m, 2H); ^13^C NMR (MHz, CDCl_3_) δ 158.76 (d, ^1^*J*_FC_ = 233.8 Hz), 158.93, 154.54, 136.80 (d, *J* = 12.5 Hz), 134.17 (d, *J* = 3.8 Hz), 125.45, 120.53 (d, *J* = 10.0 Hz), 115.03, 107.79 (d, *J* = 23.8 Hz), 97.10 (d, *J* = 26.3 Hz), 97.07, 93.10 (d, *J* = 171.3 Hz), 53.24 (d, *J* = 22.5 Hz), 44.28, 31.50 (d, *J* = 21.3 Hz); HRMS (ESI) [M + H]^+^ Calcd for C_16_H_15_N_4_F_2_: 301.1259, found: 301.1253.

#### 1-(5-(6-Fluoro-1*H*-indol-2-yl)pyrimidin-2-yl)piperidin-4-one (**44**)

Compound **39** (0.065 g, 0.16 mmol) was dissolved in TFA (1.6 mL, 21 mmol, 131 equiv), stirred at ambient temperature for 20 min, then poured into a mixture of NaHCO_3_ (2.13 g, 25.4 mmol, 1.2 equiv TFA), H_2_O (40 mL), and CH_2_Cl_2_ (40 mL). The mixture was stirred for 10 min, then the layers were separated, and the aqueous layer was extracted with CH_2_Cl_2_ (15 mL × 2). The combined CH_2_Cl_2_ layers were washed with brine (25 mL), dried over MgSO_4_, concentrated, poured onto dry silica (55 mm h × 45 mm i.d.), and eluted under vacuum: %MeOH/CH_2_Cl_2_–1% (100 mL), 2.5% (100 mL), 5% (200 mL), 10% (50 mL) to give an off-white solid (43 mg). Purification by radial chromatography (1 mm silica): %MeOH/CH_2_Cl_2_–1% (50 mL), 2.5% (25 mL) afforded **44** (0.038 g, 77%) as a light tan solid: ^1^H NMR (300 MHz, CDCl_3_) δ 8.65 (s, 2H), 8.27 (br s, 1H), 7.52 (dd, 1H, *J* = 8.7 Hz, *J* = 5.4 Hz), 7.10 (dd, 1H, *J* = 9.3 Hz, *J* = 2.1 Hz), 6.91 (ddd, 1H, *J* = 9.6 Hz, *J* = 8.7 Hz, *J* = 2.1 Hz), 6.68 (dd, 1H, *J* = 2.1 Hz, *J* = 0.6 Hz), 4.20 (t, 4H, *J* = 6.0 Hz), 2.55 (t, 4H, *J* = 6.0 Hz); HRMS (ESI) [M + H]^+^ Calcd for C_17_H_16_FN_4_O: 311.1303, found: 311.1296. X-ray quality crystals were grown by slow evaporation of acetone.

#### 6-Fluoro-2-(6-(piperidin-1-yl)pyridin-3-yl)-1*H*-indole (**45**)

Compound **40** (0.082 g, 0.21 mmol) was dissolved in TFA (2 mL, 26 mmol, 126 equiv), stirred at ambient temperature for 20 min, then poured into a mixture of Na_2_CO_3_ (2.43 g, 28.9 mmol, 1.1 equiv TFA), H_2_O (60 mL), and CH_2_Cl_2_ (60 mL). The mixture was stirred for 10 min, then the layers were separated and the aqueous layer was extracted with CH_2_Cl_2_ (10 mL × 3). The combined CH_2_Cl_2_ layers were washed with brine (30 mL), dried over MgSO_4_, then poured onto dry silica (33 mm h × 33 mm i.d.), and eluted under vacuum: %MeOH/CH_2_Cl_2_–1% (50 mL), 2% (75 mL), 3% (50 mL) to give an off-white solid (0.058 g). Purification by radial chromatography (1 mm silica): hexane/EtOAc/NEt_3_ v/v/v 90:8:2 (40 mL), 75:20:5 (50 mL) afforded **45** (0.050 g, 82%) as an off-white solid: ^1^H NMR (300 MHz, CDCl_3_) δ 8.46 (d, 1H, *J* = 2.4 Hz), 8.28 (br s, 1H), 7.68 (dd, 1H, *J* = 9.0 Hz, *J* = 2.4 Hz), 7.48 (dd, 1H, *J* = 8.7 Hz, *J* = 5.4 Hz), 7.06 (dd, 1H, *J* = 9.6 Hz, *J* = 1.8 Hz), 6.86 (ddd, 1H, *J* = 9.6 Hz, *J* = 8.7 Hz, *J* = 2.1 Hz), 6.70 (d, 1H, *J* = 8.7 Hz), 6.61 (d, 1H, *J* = 1.5 Hz), 3.59 (m, 4H), 1.67 (m, 6 H); ^13^C NMR (125 MHz, DMSO-*d*_6_) δ 158.57 (d, ^1^*J*_FC_ = 233.9 Hz), 157.98, 144.42, 136.86 (d, *J*_FC_ = 3.5 Hz), 136.66 (d, *J*_FC_ = 12.6 Hz), 134.13, 125.61, 120.25 (d, *J*_FC_ = 9.9 Hz), 116.64, 107.49 (d, *J*_FC_ = 24.0 Hz), 106.98, 96.95 (d, *J*_FC_ = 25.5 Hz), 96.51, 45.50, 24.99, 24.29; ^19^F NMR (470.6 MHz, DMSO-*d*_6_) δ −122.04 (m); HRMS (ESI) [M + H]^+^ Calcd for C_18_H_19_FN_3_: 296.1558, found: 296.1568.

#### 4-(5-(6-Fluoro-1*H*-indol-2-yl)pyridin-2-yl)morpholine (**46**)

Compound **41** (0.061 g, 0.15 mmol) was dissolved in TFA (1.5 mL, 20 mmol, 127 equiv), stirred at ambient temperature for 20 min, then poured into a mixture of Na_2_CO_3_ (1.82 g, 21.7 mmol, 1.1 equiv TFA), H_2_O (40 mL), and CH_2_Cl_2_ (40 mL). The mixture was stirred for 15 min, then the layers were separated, and the aqueous layer was extracted with CH_2_Cl_2_ (15 mL × 2). The combined CH_2_Cl_2_ layers were washed with brine (25 mL), dried over MgSO_4_, then poured onto dry silica (33 mm h × 33 mm i.d.), and eluted under vacuum: %MeOH/CH_2_Cl_2_–1% (50 mL), 2% (75 mL), 3% (50 mL) to afford **46** (0.042 g, 92%) as a light tan solid: ^1^H NMR (300 MHz, CDCl_3_) δ 8.50 (d, 1H, *J* = 2.1 Hz), 8.28 (br s, 1H), 7.75 (dd, 1H, *J* = 8.7 Hz, *J* = 2.4 Hz), 7.49 (dd, 1H, *J* = 8.7 Hz, *J* = 5.4 Hz), 7.07 (dd, 1H, *J* = 9.3 Hz, *J* = 2.1 Hz), 6.88 (ddd, 1H, *J* = 9.6 Hz, *J* = 8.7 Hz, *J* = 2.1 Hz), 6.71 (d, 1H, *J* = 8.7 Hz), 6.65 (dd, 1H, *J* = 2.1 Hz, *J* = 0.6 Hz), 3.85 (t, 4H, *J* = 4.8 Hz), 3.57 (t, 4H, *J* = 4.8 Hz); ^13^C NMR (125 MHz, DMSO-*d*_6_) δ 158.66 (d, ^1^*J*_FC_ = 234.3 Hz), 158.19, 144.28, 136.72 (d, *J*_FC_ = 12.7 Hz), 136.54 (d, *J*_FC_ = 3.4 Hz), 134.19, 125.55, 120.41 (d, *J*_FC_ = 10.1 Hz), 117.95, 107.60 (d, *J*_FC_ = 24.3 Hz), 106.92, 97.00 (d, *J*_FC_ = 25.5 Hz), 96.99, 65.91, 45.04; ^19^F NMR (470.6 MHz, DMSO-*d*_6_) δ −121.79; HRMS (ESI) [M + H]^+^ Calcd for C_17_H_17_FN_3_O: 298.1350, found: 298.1345.

#### 1-(5-(5-Fluorobenzofuran-2-yl)pyrimidin-2-yl)piperidin-4-ol (**49**)

1,4-Dioxane (30 mL) and H_2_O (6 mL) were combined and purged with N_2(g)_ for 45 min. 5-Fluorobenzofuran-2-boronic acid (**47**) (0.250 g, 1.39 mmol), compound **32** (0.420 g, 1.63 mmol, 1.2 equiv), Pd(dppf)Cl_2_·CH_2_Cl_2_ (0.097 g, 0.12 mmol, 0.09 equiv), and Na_2_CO_3_ (0.710 g, 6.70 mmol, 4.8 equiv) were flushed with N_2(g)_ for 10 min, then the H_2_O/1,4-dioxane mixture (17 mL) was added. The reaction mixture was stirred under N_2(g)_ in a heated sand bath (∼95 °C) for 14 h, then cooled to ambient temperature, filtered through Celite, and the Celite was rinsed with EtOAc. The filtrate was concentrated, then EtOAc was added and removed to give a brown residue that was dissolved in CH_2_Cl_2_, poured onto dry silica (55 mm h × 45 mm i.d.), and eluted under vacuum: hexane/EtOAc/NEt_3_ v/v/v 75:20:5 (100 mL), 50:45:5 (150 mL), 20:75:5 (250 mL), EtOAc (50 mL). The desired fractions were combined and purified by radial chromatography (2 mm silica): %EtOH/CHCl_3_–1% (50 mL), 2% (75 mL), 3% (100 mL) to give a tan solid (77 mg). The solid was suspended in CHCl_3_ (2 mL), filtered, rinsed with CHCl_3_ (1.5 mL × 2) and dried under vacuum to afford **49** (0.047 g, 11%) as a white solid: ^1^H NMR (300 MHz, DMSO-*d*_6_) δ 8.85 (s, 2H), 7.60 (dd, 1H, *J* = 9.0 Hz, *J* = 4.2 Hz), 7.43 (dd, 1H, *J* = 9.0 Hz, *J* = 2.4 Hz), 7.26 (s, 1H), 7.10 (td, 1H, *J* = 9.0 Hz, *J* = 2.4 Hz), 4.77 (d, 1H, *J* = 4.2 Hz), 4.30 (partially resolved dddd, 2 H, *J* = 13.5 Hz, *J* = 4.5 Hz), 3.77 (apparent octet, 1H, *J* = 4.2 Hz), 3.39 (ddd–partially obscured by H_2_O resonance, 2 H, *J* = 9.6 Hz, *J* = 3.3 Hz), 1.80 (m, 2H), 1.36 (m, 2H); ^13^C NMR (125 MHz, CDCl_3_) δ 160.37, 158.80 (d, ^1^*J*_FC_ = 235.0 Hz), 154.72, 154.02, 150.27, 129.89 (d, *J* = 11.3 Hz), 112.27, 111.90 (d, *J* = 10.0 Hz), 111.31 (d, *J* = 26.3 Hz), 106.16 (d, *J* = 25.0 Hz), 100.00 (d, *J* = 3.8 Hz), 65.86, 41.17, 33.91; HRMS (ESI) [M + H]^+^ Calcd for C_17_H_17_FN_3_O_2_: 314.1299, found: 314.1296.

#### *tert*-Butyl 2-(2-(4-Hydroxypiperidin-1-yl)pyrimidin-5-yl)-1*H*-indole-1-carboxylate (**50**)

1,4-Dioxane (10 mL) and H_2_O (2 mL) were combined and purged with N_2(g)_ for 10 min. Compound **32** (0.240 g, 0.930 mmol), compound **12** (0.260 g, 0.996 mmol, 1.1 equiv), Pd(dppf)Cl_2_ (0.094 g, 0.12 mmol, 0.1 equiv), and Na_2_CO_3_ (0.450 g, 4.25 mmol, 4.6 equiv) were flushed with N_2(g)_ for 15 min, then the H_2_O/1,4-dioxane mixture was added. The reaction mixture was stirred under N_2(g)_ in a heated sand bath (85 °C) for 16 h, then cooled to ambient temperature, filtered through Celite, and the Celite was rinsed with EtOAc. The solvent was removed to give a black syrup that was dissolved in CH_2_Cl_2_, poured onto dry silica (45 mm h × 45 mm i.d.), and eluted under vacuum: CH_2_Cl_2_ (100 mL), %MeOH/CH_2_Cl_2_–1% (100 mL), 2.5% (100 mL), 5% (100 mL). The desired fractions were combined, concentrated, poured onto dry silica (45 mm h × 45 mm i.d.), and eluted under vacuum: hexane/EtOAc/NEt_3_ v/v/v 90:8:2 (50 mL), 75:20:5 (100 mL), 50:45:5 (100 mL), 20:75:5 (200 mL) to give an orange/tan foam (0.25 g). Purification by radial chromatography (2 mm silica): CHCl_3_ (50 mL), %EtOH/CHCl_3_–1% (50 mL), 2% (75 mL), 3% (50 mL) afforded **50** (0.230 g, 63%) as a light yellow foam: ^1^H NMR (400 MHz, CDCl_3_) δ 8.36 (s, 2H), 8.19 (d, 1H, *J* = 8.4 Hz), 7.54 (d, 1H, *J* = 6.0 Hz), 7.32 (apparent t, 1H, *J* = 7.6 Hz), 7.25 (apparent t–partially obscured by CHCl_3_ resonance, 1H, *J* = 7.6 Hz), 6.53 (s, 1H), 4.46 (partially resolved dddd, 2 H, *J* = 13.6 Hz, *J* = 4.4 Hz), 3.99 (br s, 1H), 3.40 (partially resolved ddd, 2 H, *J* = 11.4 Hz, *J* = 2.8 Hz), 1.98 (m, 2H), 1.57 (m, 2H), 1.48 (s, 10 H); ^13^C NMR (125 MHz, CDCl_3_) δ 160.70, 157.39, 150.29, 137.39, 135.32, 129.34, 124.63, 123.28, 120.56, 117.25, 115.87, 110.52, 84.25, 68.31, 41.75, 34.32, 28.13; HRMS (ESI) [M + H]^+^ Calcd for C_22_H_27_O_3_N_4_: 395.2078, found: 395.2073.

#### *tert*-Butyl 5-Fluoro-2-(2-(4-hydroxypiperidin-1-yl)pyrimidin-5-yl)-1*H*-indole-1-carboxylate (**51**)

1,4-Dioxane (30 mL) and H_2_O (6 mL) were combined and purged with N_2(g)_ for 45 min. Compound **48** (0.250 g, 0.896 mmol), compound **32** (0.250 g, 0.969 mmol, 1.1 equiv), Pd(dppf)Cl_2_·CH_2_Cl_2_ (0.079 g, 0.097 mmol, 0.1 equiv), and Na_2_CO_3_ (0.470 g, 4.43 mmol, 5 equiv) were flushed with N_2(g)_ for 10 min, then the H_2_O/1,4-dioxane mixture (16 mL) was added. The reaction mixture was stirred under N_2(g)_ in a heated sand bath (∼95 °C) for 14 h, then cooled to ambient temperature, filtered through Celite, and the Celite was rinsed with EtOAc. The filtrate was concentrated to an oil, then EtOAc was added and removed to give a brown residue that was dissolved in CH_2_Cl_2_, poured onto dry silica (55 mm h × 45 mm i.d.), and eluted under vacuum: hexane/EtOAc/NEt_3_ v/v/v 75:20:5 (100 mL), 50:45:5 (150 mL), 20:75:5 (200 mL) to give a tan foam (0.230 g). Purification by radial chromatography (2 mm silica): %EtOH/CHCl_3_–1% (50 mL), 2% (75 mL) afforded **51** (0.170 g, 46%) as an off-white foam: ^1^H NMR (400 MHz, CDCl_3_) δ 8.35 (s, 2H), 8.14 (dd, 1H, *J* = 8.8 Hz, *J* = 4.8 Hz), 7.19 (dd, 1H, *J* = 8.8 Hz, *J* = 2.4 Hz), 7.04 (td, 1H, *J* = 9.2 Hz, *J* = 2.4 Hz), 6.49 (s, 1H), 4.46 (partially resolved dddd, 2 H, *J* = 13.6 Hz, *J* = 4.4 Hz), 4.00 (apparent octet, 1H, *J* = 4.4 Hz), 3.40 (ddd, 1H, *J* = 10.0 Hz, *J* = 3.2 Hz), 1.98 (m, 2H), 1.56 (m, 3H), 1.47 (s, 9H); ^13^C NMR (125 MHz, CDCl_3_) δ 160.81, 159.58 (d, ^1^*J*_FC_ = 238.0 Hz), 157.42, 150.10, 137.01, 133.80, 130.17 (d, *J*_FC_ = 9.9 Hz), 116.99 (d, *J*_FC_ = 4.9 Hz), 116.94, 112.32 (d, *J*_FC_ = 24.6 Hz), 110.12 (d, *J*_FC_ = 3.9 Hz), 105.91 (d, *J*_FC_ = 23.5 Hz), 84.56, 68.36, 41.75, 34.36, 28.14; HRMS (ESI) [M + H]^+^ Calcd for C_22_H_26_O_3_N_4_F: 413.1984, found: 413.1987.

#### 1-(5-(1*H*-Indol-2-yl)pyrimidin-2-yl)piperidin-4-ol (**52**)

Compound **50** (0.083 g, 0.21 mmol) was dissolved in TFA (2.0 mL, 26 mmol, 124 equiv), stirred at ambient temperature for 20 min, then poured into a mixture of NaHCO_3_ (2.40 g, 28.6 mmol, 1.1 equiv TFA), H_2_O (60 mL), and CH_2_Cl_2_ (60 mL). The mixture was stirred until gas evolution ceased, then the layers were separated, and the aqueous layer was extracted with CH_2_Cl_2_ (10 mL). The combined CH_2_Cl_2_ layers were washed with brine (25 mL), dried over MgSO_4_, and concentrated which produced a precipitate. MeOH was added (several drops) until the precipitate dissolved, then the solution was poured onto dry silica (55 mm h × 45 mm i.d.), and eluted under vacuum: %MeOH/CH_2_Cl_2_–1% (100 mL), 2.5% (100 mL), 5% (150 mL), 10% (50 mL) to give an off-white solid. The solid was dissolved/suspended in CHCl_3_ (∼4 mL), then MeOH (∼0.5 mL) was added to get the remaining solid to dissolve. The solution was purified by radial chromatography (2 mm silica): CHCl_3_ (25 mL), %EtOH/CHCl_3_–1% (50 mL), 2% (75 mL), 3% (50 mL), 4% (50 mL), 10% (60 mL) to afford **52** (0.058 g, 94%) as an off-white solid: ^1^H NMR (400 MHz, acetone-*d*_6_) δ 10.59 (br s, 1H), 8.80 (s, 2H), 7.54 (d, 1H, *J* = 7.6 Hz), 7.37 (d, 1H, *J* = 8.4 Hz), 7.08 (td, 1H, *J* = 7.6 Hz, *J* = 1.2 Hz), 7.01 (td, 1H, *J* = 7.6 Hz, *J* = 1.2), 6.78 (d, 1H, *J* = 1.2 Hz), 4.42 (partially resolved dddd, 2 H, *J* = 13.6 Hz, *J* = 4.4 Hz), 3.91 (apparent octet, 1H, *J* = 4.4 Hz), 3.84 (d, 1H, *J* = 4.4 Hz), 3.42 (ddd, 2 H, *J* = 13.4 Hz, *J* = 9.8 Hz, *J* = 3.2 Hz), 1.90 (m, 2H), 1.48 (m, 2H); HRMS (ESI) [M + H]^+^ Calcd for C_17_H_19_N_4_O: 295.1553, found: 295.1553.

#### 1-(5-(5-Fluoro-1*H*-indol-2-yl)pyrimidin-2-yl)piperidin-4-ol (**53**)

Compound **51** (0.090 g, 0.22 mmol) was dissolved in TFA (2 mL, 26 mmol, 119 equiv), stirred at ambient temperature for 20 min, then poured into a mixture of NaHCO_3_ (2.45 g, 29.2 mmol, 1.1 equiv TFA), H_2_O (60 mL), and CH_2_Cl_2_ (60 mL). The mixture was stirred for 20 min, the layers were separated, and the aqueous layer was extracted with CH_2_Cl_2_ (10 mL). The combined CH_2_Cl_2_ layers were washed with brine (25 mL) and dried over MgSO_4_. The solution was concentrated, poured onto dry silica (55 mm h × 45 mm i.d.), and eluted under vacuum: CH_2_Cl_2_ (50 mL), %MeOH/CH_2_Cl_2_–1% (100 mL), 2.5% (100 mL), 5% (100 mL), 10% (100 mL) to give a faint yellow solid (54 mg). Purification by radial chromatography (2 mm silica): hexane/EtOAc/NEt_3_ v/v/v 75:20:5 (100 mL), 50:45:5 (100 mL), 20:75:5 (250 mL) afforded **53** (0.047 g, 69%) as a light tan solid: ^1^H NMR (300 MHz, acetone-*d*_6_) δ 10.67 (br s, 1H), 8.79 (s, 2H), 7.36 (dd, 1H, *J* = 8.7 Hz, *J* = 5.4 Hz), 7.23 (dd, 1H, *J* = 9.6 Hz, *J* = 2.7 Hz), 6.87 (ddd, 1H, *J* = 9.6 Hz, *J* = 8.7 Hz, *J* = 2.7 Hz), 6.77 (m, 1H), 4.41 (partially resolved dddd, 2 H, *J* = 13.5 Hz, *J* = 4.5 Hz), 3.92 (apparent octet, 1H, *J* = 4.2 Hz), 3.83 (d, 1H, *J* = 4.2 Hz), 3.43 (ddd, 2 H, *J* = 13.2 Hz, *J* = 9.6 Hz, *J* = 3.3 Hz), 1.90 (m, 2H), 1.48 (dddd, 2 H, *J* = 13.2 Hz, *J* = 9.0 Hz, *J* = 4.2 Hz); ^13^C NMR (125 MHz, DMSO-*d*_6_) δ 160.16, 157.19 (d, ^1^*J*_FC_ = 230.0 Hz), 154.77, 135.39, 133.50, 128.96 (d, *J* = 10.0 Hz), 114.52, 111.83 (d, *J* = 10.0 Hz), 109.11 (d, *J* = 25.0 Hz), 104.15 (d, *J* = 23.8 Hz), 97.18 (d, *J* = 5.0 Hz), 66.04, 41.23, 33.92; HRMS (ESI) [M + H]^+^ Calcd for C_17_H_18_FN_4_O: 313.1459, found: 313.1456.

#### 2-Fluoro-6-(piperidin-1-yl)pyridine (**54**)

2,6-Difluoropyridine (1.340 g, 11.64 mmol), piperidine (1.2 mL, 12 mmol, 1 equiv), *i*-Pr_2_NEt (3.2 mL, 18 mmol, 1.6 equiv), and 1,4-dioxane (10 mL) were stirred at reflux under N_2(g)_ for 90 min, then cooled to ambient temperature. The reaction mixture was concentrated to a yellow oil, CH_2_Cl_2_ and hexane were added, the solution was poured onto dry silica (55 mm h × 45 mm i.d.), and eluted under vacuum: hexane (100 mL), % CH_2_Cl_2_/hexane–10% (100 mL), 25% (200 mL), 50% (100 mL), 75% (100 mL), CH_2_Cl_2_ (100 mL) to afford **54** (1.500 g, 71%) as a colorless oil: ^1^H NMR (300 MHz, CDCl_3_) δ 7.48 (dd, 1H, *J*_HF_ = 16.7 Hz, *J* = 8.1 Hz), 6.40 (dd, 1H, *J* = 8.1 Hz, *J* = 2.7 Hz), 6.09 (dd, 1H, *J* = 7.8 Hz, *J* = 3.0 Hz), 3.51 (m, 4H), 1.64 (m, 6 H); ^19^F NMR (282.5 MHz, CDCl_3_) δ −68.60 (d, *J* = 6.5 Hz); ^13^C NMR (125 MHz, CDCl_3_) δ 162.96 (d, ^1^*J*_CF_ = 233.0 Hz), 158.58 (d, ^3^*J*_CF_ = 15.9 Hz), 141.73 (d, ^3^*J*_CF_ = 8.4 Hz), 102.69 (d, ^4^*J*_CF_ = 4.0 Hz), 94.86 (d, ^2^*J*_CF_ = 37.6 Hz), 46.12, 25.46, 24.70; HRMS (ESI) [M + H]^+^ Calcd for C_10_H_14_FN_2_: 181.1136, found: 181.1133.

#### 4-(6-Fluoropyridin-2-yl)morpholine (**55**)

2,6-Difluoropyridine (1.340 g, 11.64 mmol), morpholine (1.0 mL, 12 mmol, 1 equiv), *i*-Pr_2_NEt (3 mL, 17 mmol, 1.5 equiv), and 1,4-dioxane (10 mL) were stirred at reflux under N_2(g)_ for 90 min, cooled to ambient temperature, and concentrated to an oil that was dried under vacuum to give an off-white solid. The solid was dissolved in CH_2_Cl_2_, poured onto dry silica (55 mm h × 45 mm i.d.), and eluted under vacuum: hexane (50 mL), % CH_2_Cl_2_/hexane–25% (50 mL), 50% (75 mL), 75% (100 mL), CH_2_Cl_2_ (500 mL), %MeOH/CH_2_Cl_2_–2% (75 mL), 5% (50 mL), 10% (100 mL) to afford **55** (0.720 g, 34%) as a colorless oil that slowly became a white solid: ^1^H NMR (300 MHz, CDCl_3_) δ 7.55 (dd, 1H, *J*_CF_ = 16.5 Hz, *J* = 8.1 Hz), 6.41 (dd, 1H, *J* = 8.1 Hz, *J* = 2.7 Hz), 6.21 (dd, 1H, *J* = 7.8 Hz, *J* = 2.7 Hz), 3.80 (t, 4H, *J* = 4.8 Hz), 3.50 (t, 4H, *J* = 4.8 Hz); ^19^F NMR (282.5 MHz, CDCl_3_) δ −68.47 (d, *J* = 7.1 Hz); ^13^C NMR (125 MHz, CDCl_3_) δ 162.87 (d, ^1^*J*_CF_ = 234.6 Hz), 158.64 (d, ^3^*J*_CF_ = 15.5 Hz), 142.07 (d, ^3^*J*_CF_ = 8.3 Hz), 102.74 (d, ^4^*J*_CF_ = 4.0 Hz), 96.74 (d, ^2^*J*_CF_ = 37.1 Hz), 66.65, 45.40; HRMS (ESI) [M + H]^+^ Calcd for C_9_H_12_FN_2_O: 183.0928, found: 183.0926.

#### 2-Nitro-6-(piperidin-1-yl)pyridine (**56**)

2-Chloro-6-nitropyridine (0.280 g, 1.77 mmol), piperidine (0.2 mL, 2 mmol, 1.1 equiv), *i*-Pr_2_NEt (0.5 mL, 3 mmol, 1.6 equiv), and 1,4-dioxane (5 mL) were stirred at reflux under N_2(g)_ for 2 h, then cooled to ambient temperature. The reaction mixture was poured onto dry silica (55 mm h × 45 mm i.d.) and eluted under vacuum: hexane (50 mL), hexane/EtOAc/NEt_3_ v/v/v 90:8:2 (100 mL), 75:20:5 (150 mL) to give a yellow/orange syrup (0.270 g). Purification by radial chromatography (2 mm silica): hexane/EtOAc/NEt_3_ v/v/v 90:8:2 (100 mL), 75:20:5 (25 mL) afforded **56** (0.160 g, 44%) as an orange syrup: ^1^H NMR (300 MHz, CDCl_3_) δ 7.64 (dd, 1H, *J* = 8.4 Hz, *J* = 7.5 Hz), 7.37 (d, 1H, *J* = 7.5 Hz), 6.89 (d, 1H, *J* = 8.4 Hz), 3.63 (m, 4H), 1.67 (m, 6 H); ^13^C NMR (125 MHz, CDCl_3_) δ 157.98, 156.15, 140.02, 111.88, 104.61, 46.03, 25.55, 24.62; HRMS (ESI) [M + H]^+^ Calcd for C_10_H_14_O_2_N_3_: 208.1081, found: 208.1083.

#### 4-(6-Nitropyridin-2-yl)morpholine (**57**)

2-Chloro-6-nitropyridine (0.280 g, 1.77 mmol), morpholine (0.2 mL, 2 mmol, 1.3 equiv), *i*-Pr_2_NEt (0.5 mL, 3 mmol, 1.6 equiv), and 1,4-dioxane (5 mL) were stirred at reflux under N_2(g)_ for 4 h, then cooled to ambient temperature, filtered, and the precipitate was rinsed with EtOAc. The solvent was removed from the filtrate to give an orange residue that was dissolved in CH_2_Cl_2_, poured onto dry silica (55 mm h × 45 mm i.d.), and eluted under vacuum: CH_2_Cl_2_ (200 mL), %MeOH/CH_2_Cl_2_–1% (100 mL), 2.5% (100 mL), 5% (150 mL) to give a yellow/orange solid. Purification by radial chromatography (2 mm silica): hexane/EtOAc/NEt_3_ v/v/v 90:8:2 (100 mL), 75:20:5 (75 mL) afforded **57** (0.140 g, 38%) as a yellow/orange solid: ^1^H NMR (300 MHz, CDCl_3_) δ 7.73 (dd, 1H, *J* = 8.4 Hz, *J* = 7.5 Hz), 7.49 (d, 1H, *J* = 7.5 Hz), 6.90 (d, 1H, *J* = 8.4 Hz), 3.83 (t, 4H, *J* = 5.1 Hz), *J* = 3.63 (t, 4H, *J* = 5.1 Hz); ^13^C NMR (125 MHz, CDCl_3_) δ 158.17, 156.06, 140.49, 111.79, 106.21, 66.67, 45.27; HRMS (ESI) [M + H]^+^ Calcd for C_9_H_12_N_3_O_3_: 210.0873, found: 210.0871.

#### 3-Bromo-2-fluoro-6-(piperidin-1-yl)pyridine (**58**)

Compound **54** (0.770 g, 4.27 mmol) was dissolved in CH_3_CN (25 mL) under N_2(g)_ and cooled to 0 °C. NBS (0.860 g, 4.83 mmol, 1.1 equiv) was added, the reaction mixture was stirred at 0 °C for 5 min, then warmed to ambient temperature and stirred for 6 h. The CH_3_CN was removed to give a light tan residue that was dissolved in CH_2_Cl_2_, poured onto dry silica (55 mm h × 45 mm i.d.), and eluted under vacuum with CH_2_Cl_2_ (150 mL) to give a colorless oil (1.26 g). Purification by radial chromatography (2 mm silica): %CH_2_Cl_2_/hexane–10% (100 mL), 25% (50 mL) afforded **58** (0.780 g, 70%) as a colorless oil: ^1^H NMR (300 MHz, CDCl_3_) δ 7.57 (apparent t, 1H, *J* = 8.7 Hz), 6.33 (dd, 1H, *J* = 8.7 Hz, *J* = 1.5 Hz), 3.49 (m, 4H), 1.62 (m, 6 H); ^19^F NMR (470.6 MHz, CDCl_3_) δ −66.52 (d, *J* = 8.5 Hz); ^13^C NMR (125 MHz, CD_3_OD) δ 159.43 (d, ^1^*J*_CF_ = 229.3 Hz), 158.53 (d, ^3^*J*_CF_ = 14.9 Hz), 145.54 (d, ^3^*J*_CF_ = 2.4 Hz), 106.08 (d, ^4^*J*_CF_ = 4.4 Hz), 87.26 (d, ^2^*J*_CF_ = 38.4 Hz), 47.17, 26.48, 25.66; HRMS (ESI) [M + H]^+^ Calcd for C_10_H_13_^79^BrFN_2_: 259.0241, found: 259.0238.

#### 4-(5-Bromo-6-fluoropyridin-2-yl)morpholine (**59**)

Compound **55** (0.360 g, 1.98 mmol) was dissolved in CH_3_CN (12 mL) under N_2(g)_ and cooled to 0 °C. NBS (0.390 g, 2.19 mmol, 1.1 equiv) was added, the reaction mixture was stirred at 0 °C for 5 min, then warmed to ambient temperature and stirred for 6 h. The CH_3_CN was removed to give a red/brown syrup that was dissolved in CH_2_Cl_2_, poured onto dry silica (45 mm h × 45 mm i.d.), and eluted under vacuum with CH_2_Cl_2_ (300 mL), %MeOH/CH_2_Cl_2_–1% (100 mL), 2% (100 mL) to give a white solid (0.54 g). Purification by radial chromatography (2 mm silica): %CH_2_Cl_2_/hexane–25% (100 mL), 50% (100 mL), 75% (100 mL) afforded **59** (0.280 g, 54%) as a white solid: ^1^H NMR (300 MHz, CDCl_3_) δ 7.65 (apparent t, 1H, *J* = 8.7 Hz), 6.34 (dd, 1H, *J* = 8.7 Hz, *J* = 1.5 Hz), 3.79 (t, 4H, *J* = 4.8 Hz), 3.48 (t, 4H, *J* = 4.8 Hz); ^19^F NMR (470.6 MHz, CDCl_3_) δ −66.22 (d, *J* = 8.5 Hz); ^13^C NMR (125 MHz, CDCl_3_) δ 158.35 (d, ^1^*J*_CF_ = 241.5 Hz), 157.37 (d, ^3^*J*_CF_ = 23.8 Hz), 144.64 (d, ^3^*J*_CF_ = 2.6 Hz), 104.63 (d, ^4^*J*_CF_ = 4.5 Hz), 89.09 (d, ^2^*J*_CF_ = 38.5 Hz), 66.60, 45.44; HRMS (ESI) [M + H]^+^ Calcd for C_9_H_11_^79^BrFN_2_O: 261.0033, found: 261.0063.

#### 3-Bromo-2-nitro-6-(piperidin-1-yl)pyridine (**60**)

Compound **56** (0.160 g, 0.772 mmol) was flushed with N_2(g)_, then dissolved in CH_3_CN (10 mL) and cooled to 0 °C. NBS (0.140 g, 0.787 mmol) was added, the reaction mixture was warmed to ambient temperature, and stirred under N_2(g)_ for 5.5 h. The CH_3_CN was removed to give an orange syrup, then CH_2_Cl_2_ and hexane were added and removed to give an orange residue that was dissolved in CH_2_Cl_2_, poured onto dry silica (45 mm h × 45 mm i.d.), and eluted under vacuum: hexane (50 mL), %CH_2_Cl_2_/hexane–25% (100 mL), 50% (100 mL), CH_2_Cl_2_ (150 mL) to give a dark yellow syrup. Purification by radial chromatography (2 mm silica): %CH_2_Cl_2_/hexane–25% (100 mL), 50% (50 mL) afforded **60** (0.150 g, 68%) as a yellow syrup: ^1^H NMR (500 MHz, CDCl_3_) δ 7.64 (d, 1H, *J* = 9.0 Hz), 6.66 (d, 1H, *J* = 9.0 Hz), 3.54 (t, 4H, *J* = 5.5 Hz), 1.66 (m, 2H), 1.62 (m, 4 H); ^13^C NMR (125 MHz, CDCl_3_) δ 156.45, 156.14, 144.12, 111.10, 92.85, 46.17, 25.49, 24.52; HRMS (ESI) [M + H]^+^ Calcd for C_10_H_13_^79^BrN_3_O_2_: 286.0186, found: 286.0172.

#### 4-(5-Bromo-6-nitropyridin-2-yl)morpholine (**61**)

Compound **57** (0.120 g, 0.574 mmol) was dissolved in CH_3_CN (10 mL), cooled to 0 °C, and NBS (0.150 g, 0.843 mmol, 1.5 equiv) was added. The reaction mixture was stirred in a capped flask at ambient temperature for 6 h, concentrated to an orange oil, then CH_2_Cl_2_ and hexane were added and removed 2× to give an orange solid that was dried under vacuum. The solid was dissolved in CH_2_Cl_2_, poured onto dry silica (45 mm h × 45 mm i.d.), and eluted under vacuum: CH_2_Cl_2_ (400 mL), 1% MeOH/CH_2_Cl_2_ (100 mL) to give a yellow solid (0.130 g). Purification by radial chromatography (2 mm silica) with CHCl_3_ (100 mL) afforded **61** (0.082 g, 50%) as a yellow solid: ^1^H NMR (400 MHz, CDCl_3_) δ 7.76 (d, 1H, *J* = 9.2 Hz), 6.68 (d, 1H, *J* = 9.2 Hz), 3.80 (t, 4H, *J* = 4.8 Hz), 3.54 (t, 4H, *J* = 4.8 Hz); ^13^C NMR (125 MHz, CDCl_3_) δ 156.65, 156.07, 144.61, 110.99, 94.88, 66.51, 45.21; HRMS (ESI) [M + H]^+^ Calcd for C_9_H_11_^79^BrN_3_O_3_: 287.9978, found: 287.9981.

#### 4-(4-Fluoropyridin-2-yl)morpholine (**62**)

2-Bromo-4-fluoropyridine (1.00 g, 5.68 mmol), morpholine (0.500 g, 5.74 mmol), sodium *tert*-butoxide (0.580 g, 6.04 mmol), toluene (50 mL), Pd_2_(dba)_3_ (0.073 g, 0.080 mmol) and XantPhos (0.140 g, 0.242 mmol) were flushed with N_2(g)_ for 10 min, then heated at 100 °C overnight. The reaction mixture was cooled to ambient temperature, filtered through a short silica gel pad, and washed with EtOAc. The filtrate was evaporated to dryness and the residue was purified by radial chromatography (hexane/EtOAc v/v 95:5 to 80:20) to afford **62** (0.880 g, 85%) as white solid: ^1^H NMR (CDCl_3_, 300 MHz): δ 8.13 (dd, 1H, *J* = 9.4 Hz, *J* = 5.4 Hz), 6.41 (ddd, 1H, *J* = 8.1 Hz, *J* = 5.4 Hz, *J* = 2.1 Hz), 6.28 (dd, 1H, *J* = 12.3 Hz, *J* = 2.1 Hz), 3.81 (apparent t, 4H, *J* = 4.8 Hz), 3.49 (apparent t, 4H, *J* = 4.8 Hz); HRMS (ESI) [M + H]^+^ Calcd for C_9_H_12_ON_2_F: 183.0928, found: 183.0931.

#### 4-(5-Bromo-4-fluoropyridin-2-yl)morpholine (**63**)

Compound **62** (0.870 g, 4.78 mmol) was dissolved in CH_3_CN (50 mL), cooled to 0 °C, then NBS (0.850 g, 4.78 mmol) was added. The reaction mixture was stirred at 0 °C for 90 min, the solvent was removed, and the residue was purified by flash column chromatography (4:1 v/v hexane/EtOAc) to afford **63** (1.20 g, 96%) as an off-white solid: ^1^H NMR (300 MHz, CDCl_3_) δ 8.21 (d, 1H, *J* = 9.9 Hz), 6.36 (d, 1H, *J* = 11.5 Hz), 3.80 (apparent t, 4H, *J* = 4.8 Hz), 3.48 (apparent t, 4H, *J* = 4.8 Hz). HRMS (ESI) [M + H]^+^ Calcd for C_9_H_11_ON_2_BrF: 261.0033, found: 261.0029.

#### 5-Bromo-3-fluoro-2-(piperidin-1-yl)pyridine (**64**)

2,5-Dibromo-3-fluoropyridine (0.270 g, 1.06 mmol), piperidine (0.085 g, 1.0 mmol), sodium *tert*-butoxide (0.144 g, 1.50 mmol), toluene (20 mL), Pd_2_(dba)_3_ (0.018 g, 0.020 mmol), and XantPhos (0.035 g, 0.061 mmol) were flushed with N_2(g)_ for 5 min, then heated at 100 °C overnight. The reaction mixture was cooled to ambient temperature, filtered through a short silica gel pad, and washed with EtOAc. The filtrate was evaporated to dryness and the residue was purified by radial chromatography (7:3 v/v hexane/EtOAc) to afford **64** (0.150 g, 58%) as a white solid: ^1^H NMR (300 MHz, CDCl_3_): δ 8.02 (dd, 1H, *J* = 2.1 Hz, *J* = 0.9 Hz), 7.33 (dd, 1H, *J* = 12.0 Hz, *J* = 2.1 Hz), 3.41 (m, 4H), 1.65 (m, 6 H); HRMS (ESI) [M + H]^+^ Calcd for C_10_H_13_N_2_BrF: 259.0241, found: 259.0237.

#### 4-(5-Bromo-3-fluoropyridin-2-yl)morpholine (**65**)

2,5-Dibromo-3-fluoropyridine (0.270 g, 1.06 mmol), morpholine (0.087 mg, 1.0 mmol), sodium *tert*-butoxide (0.144 g, 1.50 mmol), toluene (20 mL), Pd_2_(dba)_3_ (0.018 g, 0.020 mmol), and XantPhos (0.035 g, 0.061 mmol) were flushed with N_2(g)_ for 5 min, then heated at 100 °C overnight. The reaction mixture was cooled to ambient temperature, filtered through a short silica gel pad, and washed with EtOAc (10 mL × 4). The filtrate was evaporated to dryness and the residue was purified by radial chromatography (hexane/EtOAc v/v 95:5 to 80:20) to afford **65** (0.160 g, 61%) as white solid: ^1^H NMR (300 MHz, CDCl_3_) δ 8.05 (dd, 1H, *J* = 1.8 Hz, *J* = 0.9 Hz), 7.39 (dd, 1H, *J* = 12.0 Hz, *J* = 1.8 Hz), 3.82 (apparent t, 4H, *J* = 4.8 Hz), 3.46 (apparent t, 4H, *J* = 4.8 Hz); HRMS (ESI) [M + H]^+^ Calcd for C_9_H_11_ON_2_BrF: 261.0033, found: 261.0044.

#### 2-Chloro-3-fluoro-5-(piperidin-1-yl)pyridine (**66**)

5-Bromo-2-chloro-3-fluoropyridine (0.270 g, 1.28 mmol), piperidine (0.088 g, 1.03 mmol), sodium *tert*-butoxide (0.144 g, 1.50 mmol), toluene (20 mL), Pd_2_(dba)_3_ (0.018 g, 0.020 mmol), and XantPhos (0.035 g, 0.061 mmol) were flushed with N_2(g)_ for 5 min, then heated at 100 °C overnight. The reaction mixture was cooled to ambient temperature, filtered through a short silica gel pad, and washed with EtOAc. The filtrate was evaporated to dryness and the residue was purified by radial chromatography (7:3 v/v hexane/EtOAc) to afford **66** (0.220 g, 99%) as white solid: ^1^H NMR (300 MHz, CDCl_3_) δ 7.84 (d, 1H, *J* = 2.7 Hz), 6.95 (dd, 1H, *J* = 11.4 Hz, *J* = 2.7 Hz), 3.20 (m, 4H), 1.67 (m, 6 H); HRMS (ESI) [M + H]^+^ Calcd for C_10_H_13_N_2_ClF: 215.0746, found: 215.0743.

#### 4-(6-Chloro-5-fluoropyridin-3-yl)morpholine (**67**)

5-Bromo-2-chloro-3-fluoropyridine (0.270 g, 1.28 mmol), morpholine (0.110 g, 1.26 mmol), sodium *tert*-butoxide (0.144 g, 1.50 mmol), toluene (20 mL), Pd_2_(dba)_3_ (0.018 g, 0.020 mmol), and XantPhos (0.035 g, 0.061 mmol) were flushed with N_2(g)_ for 5 min, then heated at 100 °C overnight. The reaction mixture was cooled to ambient temperature, filtered through a short silica gel pad, and washed with EtOAc. The filtrate was evaporated to dryness and the residue was purified by radial chromatography (7:3 v/v hexane/EtOAc) to afford **67** (0.250 g, 92%) as white solid: ^1^H NMR (300 MHz, CDCl_3_) δ 7.85 (d, 1H, *J* = 2.7 Hz), 6.97 (dd, 1H, *J* = 10.8 Hz, *J* = 2.7 Hz), 3.87 (m–AA’XX’, 4H, *J* = 4.8 Hz, *J* = 1.8 Hz), 3.19 (m–AA’XX’, 4H, *J* = 4.8 Hz, *J* = 1.8 Hz); HRMS (ESI) [M + H]^+^ Calcd for C_9_H_11_ON_2_ClF: 217.0539, found: 217.0536.

#### 4-(6-Chloro-4-fluoropyridin-3-yl)morpholine (**68**)

2-Chloro-4-fluoro-5-bromopyridine (0.270 g, 1.28 mmol), morpholine (0.087 g, 1.0 mmol), sodium *tert*-butoxide (0.144 g, 1.50 mmol), toluene (20 mL), Pd_2_(dba)_3_ (0.018 g, 0.020 mmol), and XantPhos (0.035 g, 0.061 mmol) were flushed with N_2(g)_ for 5 min, then heated at 100 °C overnight. The reaction mixture was cooled to ambient temperature, filtered through a short silica gel pad, and washed with EtOAc. The filtrate was evaporated to dryness and the residue was purified by radial chromatography (hexane/EtOAc v/v 95:5 to 80:20) to afford **68** (0.097 g, 45%) as white solid: ^1^H NMR (300 MHz, CDCl_3_) δ 7.98 (d, 1H, *J* = 10.5 Hz), 7.04 (d, 1H, *J* = 11.1 Hz), 3.86 (m–AA’XX’, 4H, *J* = 4.8 Hz, *J* = 1.8 Hz), 3.12 (m–AA’XX’, 4H, *J* = 4.8 Hz, *J* = 1.8 Hz); HRMS (ESI) [M + H]^+^ Calcd for C_9_H_11_ON_2_ClF: 217.0539, found: 217.0536.

#### *tert*-Butyl 2-(2-Fluoro-6-(piperidin-1-yl)pyridin-3-yl)-1*H*-indole-1-carboxylate (**69**)

1,4-Dioxane (30 mL) and H_2_O (6 mL) were combined and purged with N_2(g)_ for 30 min. Compound **12** (0.250 g, 0.958 mmol), compound **58** (0.310 g, 1.20 mmol, 1.2 equiv), Pd(dppf)Cl_2_ (0.095 g, 0.13 mmol, 0.14 equiv), and Na_2_CO_3_ (0.470 g, 4.43 mmol, 4.6 equiv) were flushed with N_2(g)_ for 10 min, then the H_2_O/1,4-dioxane mixture (15 mL) was added. The reaction mixture was stirred at reflux under N_2(g)_ for 4 h, then cooled to ambient temperature, filtered through Celite, and the Celite was rinsed with 1,4-dioxane, then EtOAc. The solvent was removed from the filtrate to give a brown oil that was dried under vacuum to give a brown residue. The residue was dissolved in CH_2_Cl_2_, poured onto dry silica (55 mm h × 45 mm i.d.), and eluted under vacuum with CH_2_Cl_2_ (250 mL) to give a faint tan syrup (0.19 g). Purification by radial chromatography (2 mm silica) with 90:8:2 v/v/v hexane/EtOAc/NEt_3_ (100 mL) afforded **69** (0.087 g, 23%) as a white foam: ^1^H NMR (300 MHz, CDCl_3_) δ 8.20 (d, 1H, *J* = 8.4 Hz), 7.55 (m, 2H), 7.31 (ddd, 1H, *J* = 8.4 Hz, *J* = 7.2 Hz, *J* = 1.2 Hz), 7.22 (td, 1H, *J* = 7.2 Hz, *J* = 0.9 Hz), 6.53 (s, 1H), 6.49 (dd, 1H, *J* = 8.4 Hz, *J* = 1.8 Hz), 3.58 (m, 4H), 1.66 (m, 6H), 1.45 (s, 9H); ^19^F NMR (282.5 MHz, CDCl_3_) δ −67.96 (d, *J* = 9.6 Hz); HRMS (ESI) [M + H]^+^ Calcd for C_23_H_27_FN_3_O_2_: 396.2082, found: 396.2074.

#### *tert*-Butyl 2-(2-Fluoro-6-morpholinopyridin-3-yl)-1*H*-indole-1-carboxylate (**70**)

1,4-Dioxane (30 mL) and H_2_O (6 mL) were combined and purged with N_2(g)_ for 30 min. Compound **12** (0.240 g, 0.919 mmol), compound **59** (0.250 g, 0.958 mmol), Pd(dppf)Cl_2_ (0.110 g, 0.150 mmol, 0.16 equiv), and Na_2_CO_3_ (0.300 g, 2.83 mmol, 3.1 equiv) were flushed with N_2(g)_ for 15 min, then the H_2_O/1,4-dioxane mixture (15 mL) was added. The reaction mixture was stirred at reflux under N_2(g)_ for 4 h, then cooled to ambient temperature, filtered through Celite, and the Celite was rinsed with 1,4-dioxane, then EtOAc. The solvent was removed from the filtrate to give a brown oil that was dried under vacuum to give a brown residue. The residue was dissolved in CH_2_Cl_2_, poured onto dry silica (55 mm h × 45 mm i.d.), and eluted under vacuum: CH_2_Cl_2_ (100 mL), %MeOH/CH_2_Cl_2_–1% (100 mL), 2.5% (100 mL), 5% (150 mL). The desired fractions were combined, concentrated, poured onto dry silica (55 mm h × 45 mm i.d.), and eluted under vacuum: hexane/EtOAc/NEt_3_ v/v/v 75:20:5 (300 mL), 50:45:5 (100 mL) to give a brown residue (0.23 g). Purification by radial chromatography (2 mm silica) hexane/EtOAc/NEt_3_ v/v/v 90:8:2 (100 mL), 75:20:5 (100 mL) afforded **70** (0.200 g, 55%) as a white foam: ^1^H NMR (300 MHz, CDCl_3_) δ 8.20 (d, 1H, *J* = 8.1 Hz), 7.62 (dd, 1H, *J* = 9.6 Hz, *J* = 8.1 Hz), 7.54 (d, 1H, *J* = 7.2 Hz), 7.32 (ddd, 1H, *J* = 8.4 Hz, *J* = 7.2 Hz, *J* = 1.2 Hz), 7.23 (td, 1H, *J* = 7.5 Hz, *J* = 0.9 Hz), 6.55 (s, 1H), 6.48 (dd, 1H, *J* = 8.1 Hz, *J* = 1.8 Hz), 3.82 (t, 4H, *J* = 4.8 Hz), 3.54 (t, 4H, *J* = 4.8 Hz), 1.46 (s, 9H); ^13^C NMR (125 MHz, CDCl_3_) δ 159.39 (d, ^1^*J*_FC_ = 237.7 Hz), 158.10 (d, *J*_FC_ = 15.6 Hz), 150.26, 142.03 (d, *J*_FC_ = 4.8 Hz), 137.27, 133.70 (d, *J*_FC_ = 4.3 Hz), 129.22, 124.62, 123.01, 120.57, 115.69, 110.61, 105.72 (d, *J*_FC_ = 31.2 Hz), 102.33 (d, *J*_FC_ = 3.8 Hz), 83.81, 66.73, 45.64, 28.01; ^19^F NMR (282.5 MHz, CDCl_3_) δ −67.85 (d, *J* = 9.9 Hz); HRMS (ESI) [M + H]^+^ Calcd for C_22_H_25_FN_3_O_3_: 398.1875, found: 398.1867.

#### *tert*-Butyl 2-(2-Nitro-6-(piperidin-1-yl)pyridin-3-yl)-1*H*-indole-1-carboxylate (**71**)

1,4-Dioxane (15 mL) and H_2_O (3 mL) were combined and purged with N_2(g)_ for 30 min. Compound **12** (0.092 g, 0.35 mmol), compound **60** (0.110 g, 0.384 mmol), Pd(dppf)Cl_2_ (0.032 g, 0.044 mmol, 0.1 equiv), and Na_2_CO_3_ (0.196 g, 1.85 mmol, 5.2 equiv) were flushed with N_2(g)_ for 10 min, then the H_2_O/1,4-dioxane mixture (10 mL) was added. The reaction mixture was stirred at reflux under N_2(g)_ for 5 h, then cooled to ambient temperature, filtered through Celite, and the Celite was rinsed with EtOAc. The solvent was removed from the filtrate to give a dark green/black residue that was dissolved in CH_2_Cl_2_, poured onto dry silica (45 mm h × 45 mm i.d.), and eluted under vacuum with 75:20:5 v/v/v hexane/EtOAc/NEt_3_ (200 mL) to give a dark orange syrup (0.140 g). Purification by radial chromatography (2 mm silica): hexane/EtOAc/NEt_3_ v/v/v 90:8:2 (100 mL), 75:20:5 (100 mL) gave a yellow solid (0.065 g) that was again purified by radial chromatography (2 mm silica) with CHCl_3_ (50 mL) to give an orange foam (0.056 g). Purification by radial chromatography (1 mm silica): %CH_2_Cl_2_/hexane–25% (25 mL), 50% (50 mL), CH_2_Cl_2_ (25 mL) afforded **71** (0.048 g, 32%) as a yellow/orange foam: ^1^H NMR (400 MHz, CDCl_3_) δ 8.20 (d, 1H, *J* = 8.4 Hz), 7.58 (d, 1H, *J* = 8.8 Hz), 7.53 (d, 1H, *J* = 8.0 Hz), 7.33 (apparent t, 1H, *J* = 7.6 Hz), 7.24 (apparent t - partially obscured by CHCl_3_ resonance, 1H, *J* = 7.2 Hz), 6.86 (d, 1H, *J* = 8.8 Hz), 6.50 (s, 1H), 3.66 (m, 4H), 1.67 (m, 6H), 1.41 (s, 9H); ^13^C NMR (125 MHz, CDCl_3_) δ 157.10, 154.90, 150.07, 142.88, 136.97, 134.21, 129.21, 124.83, 123.08, 120.72, 116.06, 111.29, 110.65, 109.53, 83.90, 46.24, 27.97, 25.60, 24.74; HRMS (ESI) [M + H]^+^ Calcd for C_23_H_27_N_4_O_4_: 423.2027, found: 423.2030. X-ray quality crystals were grown by slow evaporation of CH_2_Cl_2_/hexane.

#### *tert*-Butyl 2-(6-Morpholino-2-nitropyridin-3-yl)-1*H*-indole-1-carboxylate (**72**)

1,4-Dioxane (15 mL) and H_2_O (3 mL) were combined and purged with N_2(g)_ for 30 min. Compound **12** (0.060 g, 0.23 mmol), compound **61** (0.066 g, 0.23 mmol), Pd(dppf)Cl_2_ (0.019 g, 0.026 mmol, 0.1 equiv), and Na_2_CO_3_ (0.117 g, 1.10 mmol, 4.8 equiv) were flushed with N_2(g)_, then the H_2_O/1,4-dioxane mixture (10 mL) was added. The reaction mixture was stirred at reflux under N_2(g)_ for 4 h, then cooled to ambient temperature, filtered through Celite, and the Celite was rinsed with EtOAc. The filtrate was concentrated, then EtOAc was added and removed to give a dark green/brown oil. CH_2_Cl_2_ and hexane were added and removed to give a residue that was dissolved in CH_2_Cl_2_, poured onto dry silica (45 mm h × 45 mm i.d.), and eluted under vacuum: CH_2_Cl_2_ (200 mL), %MeOH/CH_2_Cl_2_–1% (100 mL), 2% (150 mL) to give a dark yellow/brown residue (69 mg). Purification by radial chromatography (1 mm silica): CH_2_Cl_2_ (100 mL) afforded **72** (0.036 g, 37%) as a yellow/orange foam: ^1^H NMR (400 MHz, CDCl_3_) δ 8.18 (d, 1H, *J* = 8.1 Hz), 7.67 (d, 1H, *J* = 8.1 Hz), 7.54 (d, 1H, *J* = 7.6 Hz), 7.34 (ddd, 1H, *J* = 8.4 Hz, *J* = 7.2 Hz, *J* = 1.2 Hz), 7.25 (m - partially obscured by CHCl_3_ resonance, 1H), 6.88 (d, 1H, *J* = 8.4 Hz), 6.52 (s, 1H), 3.84 (t, 4H, *J* = 4.8 Hz), 3.65 (t, 4H, *J* = 4.8 Hz), 1.43 (s, 9H); ^13^C NMR (100 MHz, CDCl_3_) δ 157.25, 154.70, 150.06, 143.25, 136.89, 133.83, 129.17, 125.00, 123.17, 120.83, 116.09, 113.03, 110.87, 109.56, 84.12, 66.67, 45.35, 28.01; HRMS (ESI) [M + H]^+^ Calcd for C_22_H_25_N_4_O_5_: 425.1820, found: 425.1815. X-ray quality crystals were grown by slow evaporation of CH_2_Cl_2_/hexane.

#### 4-(5-(1*H*-Indol-2-yl)-6-nitropyridin-2-yl)morpholine (**73**)

Isolated as a side-product from the reaction of **72**: ^1^H NMR (400 MHz, acetone-*d*_6_) δ 10.53 (br s, 1H), 8.09 (d, 1H, *J* = 8.8 Hz), 7.56 (d, 1H, *J* = 7.6 Hz), 7.41 (d, 1H, *J* = 8.4 Hz), 7.20 (d, 1H, *J* = 8.8 Hz), 7.13 (t, 1H, *J* = 7.6 Hz), 7.04 (t, 1H, *J* = 7.6 Hz), 6.49 (s, 1H), 3.77 (t, 4H, *J* = 4.8 Hz), 3.62 (t, 4H, *J* = 4.8 Hz); ^13^C NMR (125 MHz, DMSO-*d*_6_) δ 156.31, 154.79, 140.95, 136.77, 130.23, 128.21, 121.94, 120.14, 119.51, 111.30, 110.32, 107.21, 100.09, 65.66, 44.65; HRMS (ESI) [M + H]^+^ Calcd for C_17_H_17_N_4_O_3_: 325.1295, found: 325.1295.

#### 2-(2-Fluoro-6-(piperidin-1-yl)pyridin-3-yl)-1*H*-indole (**74**)

Compound **69** (0.071 g, 0.18 mmol) and TFA (1.8 mL, 23 mmol, 130 equiv) were stirred for 20 min, then poured into a mixture of NaHCO_3_ (2.290 g, 27.26 mmol, 1.2 equiv TFA), H_2_O (45 mL), and CH_2_Cl_2_ (45 mL). The mixture was stirred for 10 min, the layers were separated, and the aqueous layer was extracted with CH_2_Cl_2_ (20 mL). The combined CH_2_Cl_2_ layers were washed with brine (25 mL), dried over MgSO_4_, concentrated, poured onto dry silica (33 mm h × 33 mm i.d.), and eluted under vacuum: hexane/EtOAc/NEt_3_ v/v/v 90:8:2 (25 mL), 75:20:5 (200 mL), 50:45:5 (50 mL) to afford **74** (0.025 g, 47%) as an off-white solid: ^1^H NMR (400 MHz, CDCl_3_) δ 8.77 (br s, 1H), 7.92 (dd, 1H, *J* = 10.6 Hz, *J* = 8.4 Hz), 7.59 (d, 1H, *J* = 8.0 Hz), 7.39 (d, 1H, *J* = 7.6 Hz), 7.17 (td, 1H, *J* = 7.6 Hz, *J* = 0.8 Hz), 7.10 (td, 1H, *J* = 7.6 Hz, *J* = 0.8 Hz), 6.72 (d, 1H, *J* = 1.6 Hz), 6.54 (dd, 1H, *J* = 8.4 Hz, *J* = 2.4 Hz), 3.59 (m, 4H), 1.67 (m, 6 H); ^13^C NMR (125 MHz, DMSO-*d*_6_) δ 158.00 (d, ^1^*J*_FC_ = 236.7 Hz), 156.04 (d, *J*_FC_ = 16.5 Hz), 139.16 (d, *J*_FC_ = 4.4 Hz), 136.42, 131.47 (d, *J*_FC_ = 7.3 Hz), 128.57, 121.11, 119.60, 119.21, 111.00, 103.91 (d, *J*_FC_ = 3.5 Hz), 100.65 (d, *J*_FC_ = 28.0 Hz), 99.81 (d, *J*_FC_ = 8.3 Hz), 45.44, 24.93, 24.09; ^19^F NMR (470.6 MHz, DMSO-*d*_6_) δ −67.52; ^19^F NMR (376.5 MHz, CDCl_3_) δ −70.32; HRMS (ESI) [M + H]^+^ Calcd for C_18_H_19_FN_3_: 296.1558, found: 296.1604.

#### 4-(6-Fluoro-5-(1*H*-indol-2-yl)pyridin-2-yl)morpholine (**75**, JSS20–183A)

Compound **70** (0.076 g, 0.19 mmol) and TFA (1.8 mL, 23 mmol, 123 equiv) were stirred for 20 min, then poured into a mixture of NaHCO_3_ (2.220 g, 26.43 mmol, 1.1 equiv TFA), H_2_O (45 mL), and CH_2_Cl_2_ (45 mL). The mixture was stirred for 10 min, the layers were separated, and the aqueous layer was extracted with CH_2_Cl_2_ (20 mL). The combined CH_2_Cl_2_ layers were washed with brine (25 mL), dried over MgSO_4_, concentrated, poured onto dry silica (33 mm h × 33 mm i.d.), and eluted under vacuum: hexane/EtOAc/NEt_3_ v/v/v 75:20:5 (100 mL), 50:45:5 (200 mL), 20:75:5 (75 mL) to afford **75** (0.054 g, 95%) as a light tan solid: ^1^H NMR (400 MHz, CDCl_3_) δ 8.78 (br s, 1H), 7.99 (dd, 1H, *J* = 10.4 Hz, *J* = 8.8 Hz), 7.60 (d, 1H, *J* = 7.6 Hz), 7.40 (d, 1H, *J* = 8.0 Hz), 7.18 (td, 1H, *J* = 7.6 Hz, *J* = 0.8 Hz), 7.11 (td, 1H, *J* = 7.6 Hz, *J* = 0.8 Hz), 6.77 (d, 1H, *J* = 1.2 Hz), 6.55 (dd, 1H, *J* = 8.4 Hz, *J* = 2.0 Hz), 3.82 (t, 4H, *J* = 4.8 Hz), 3.57 (t, 4H, *J* = 4.8 Hz); ^1^H NMR (500 MHz, DMSO-*d*_6_) δ 11.31 (s, 1H), 8.18 (dd, 1H, *J* = 10.5 Hz, *J* = 8.5 Hz), 7.51 (d, 1H, *J* = 8.0 Hz), 7.40 (d, 1H, *J* = 8.0 Hz), 7.08 (t, 1H, *J* = 7.5 Hz), 6.99 (t, 1H, *J* = 7.5 Hz), 6.88 (d, 1H, *J* = 8.5 Hz), 6.73 (s, 1H), 3.71 (t, 4H, *J* = 5.0 Hz), 3.51 (t, 4H, *J* = 5.0 Hz); ^1^H NMR (500 MHz, acetone-*d*_6_) δ 10.45 (br s, 1H), 8.16 (dd, 1H, *J* = 10.5 Hz, *J* = 9.0 Hz), 7.54 (d, 1H, *J* = 8.0 Hz), 7.42 (d, 1H, *J* = 8.0 Hz), 7.09 (t, 1H, *J* = 7.5 Hz), 7.01 (t, 1H, *J* = 7.5 Hz), 6.79 (overlapping resonances, 2H), 3.76 (t, 4H, *J* = 5.0 Hz), 3.56 (t, 4H, *J* = 5.0 Hz); ^13^C NMR (125 MHz, DMSO-*d*_6_) δ 157.86 (d, ^1^*J*_FC_ = 237.2 Hz), 156.31 (d, *J*_FC_ = 16.1 Hz), 139.26 (d, *J*_FC_ = 4.4 Hz), 136.47, 131.13 (d, *J*_FC_ = 7.2 Hz), 128.50, 121.30, 119.71, 119.27, 111.06, 104.14 (d, *J*_FC_ = 3.6 Hz), 102.11 (d, *J*_FC_ = 27.5 Hz), 100.23 (d, *J*_FC_ = 8.6 Hz), 65.71, 44.83; ^19^F NMR (376.5 MHz, CDCl_3_) δ −70.37; HRMS (ESI) [M + H]^+^ Calcd for C_17_H_17_FN_3_O: 298.1350, found: 298.1396. X-ray quality crystals were grown by slow evaporation of CDCl_3._

#### *tert*-Butyl 5-Fluoro-2-(2-morpholinopyrimidin-5-yl)-1*H*-indole-1-carboxylate (**76**)

Compound **48** (0.210 g, 0.752 mmol), compound **30** (0.100 g, 0.410 mmol), and Pd(dppf)Cl_2_ (0.020 g, 0.027 mmol) were dissolved in 1,4-dioxane (10 mL), then K_2_CO_3(aq)_ (2 M, 0.5 mL) was added. The reaction mixture was flushed with N_2(g)_ for 5 min, then heated at 100 °C under N_2(g)_ overnight. The reaction mixture was cooled to ambient temperature, evaporated to dryness, the residue was dissolved in EtOAc (30 mL), washed with H_2_O (10 mL), dried over MgSO_4_, and filtered. The filtrate was passed through a silica gel pad, the silica was washed with EtOAc, and the filtrate was concentrated to dryness. The residue was purified by radial chromatography (hexane/EtOAc v/v 90:10 to 80:20) to afford **76** (0.150 g, 92%) as a solid: ^1^H NMR (300 MHz, CDCl_3_) δ 8.38 (s, 2H), 8.13 (dd, 1H, *J* = 9.0 Hz, *J* = 4.5 Hz), 7.20 (dd, 1H, *J* = 8.7 Hz, *J* = 2.7 Hz), 7.05 (td, 1H, *J* = 9.0 Hz, *J* = 2.7 Hz), 6.50 (d, 1H, *J* = 0.3 Hz), 3.88 (m, 4H), 3.79 (m, 4H), 1.49 (s, 9H); HRMS (ESI) [M + H]^+^ Calcd for C_21_H_24_O_3_N_4_F: 399.1827, found: 399.1837.

#### *tert*-Butyl 5-Fluoro-2-(6-morpholinopyridin-3-yl)-1*H*-indole-1-carboxylate (**77**)

Compound **48** (0.210 g, 0.752 mmol), compound **35** (0.100 g, 0.411 mmol), Pd(dppf)Cl_2_ (0.020 g, 0.027 mmol), 1,4-dioxane (10 mL), and K_2_CO_3(aq)_ (2 M, 0.5 mL) were flushed with N_2(g)_ for 10 min, then stirred at 100 °C under N_2(g)_ overnight. The reaction mixture was cooled to ambient temperature, evaporated to dryness, the residue was dissolved in EtOAc (30 mL), washed with H_2_O (10 mL), dried over MgSO_4_, and filtered. The filtrate was passed through a silica gel pad, the silica was washed with EtOAc, and the filtrate was concentrated to dryness. The residue was purified by radial chromatography (hexane/EtOAc v/v 90:10 to 80:20) to afford **77** (0.120 g, 73%) as a solid: ^1^H NMR (300 MHz, CDCl_3_) δ 8.26 (d, 1H, *J* = 2.1 Hz), 8.12 (dd, 1H, *J* = 9.0 Hz, *J* = 4.5 Hz), 7.55 (dd, 1H, *J* = 8.7 Hz, *J* = 1.8 Hz), 7.19 (dd, 1H, *J* = 8.7 Hz, *J* = 2.4 Hz), 7.03 (td, 1H, *J* = 9.0 Hz, *J* = 2.4 Hz), 6.68 (d, 1H, *J* = 8.7 Hz), 6.48 (s, 1H), 3.85 (t, 4H, *J* = 4.8 Hz), 3.57 (partially resolved t, 4H, *J* = 4.8 Hz), 1.43 (s, 9H); HRMS (ESI) [M + H]^+^ Calcd for C_22_H_25_O_3_N_3_F: 398.1875, found: 398.1887.

#### 4-(5-(5-Fluoro-1*H*-indol-2-yl)pyrimidin-2-yl)morpholine (**78**)

Compound **76** (0.140 g, 0.351 mmol) was dissolved in TFA (2 mL) and stirred at ambient temperature for 2 h, then the TFA was removed with N_2(g)_ flow. The residue was neutralized with aqueous NaHCO_3_ to give a solid that was washed with cold CH_2_Cl_2_ to afford **78** (0.098 g, 93%) as an off-white solid: ^1^H NMR (300 MHz, acetone-*d*_6_) δ 10.80 (br s, 1H), 8.83 (s, 2H), 7.37 (dd, 1H, *J* = 8.7 Hz, *J* = 4.5 Hz), 7.23 (dd, 1H, *J* = 9.6 Hz, *J* = 2.4 Hz), 6.88 (dd, 1H, *J* = 9.6 Hz, *J* = 8.7 Hz, *J* = 2.4 Hz), 6.79 (m, 1H), 3.82 (m, 4H), 3.71 (m, 4 H); ^13^C NMR (125 MHz, DMSO-*d*_6_): 160.33, 157.23 (d, ^1^*J*_FC_ = 231.4 Hz), 154.69, 135.15, 133.59, 128.93 (d, *J*_FC_ = 10.3 Hz), 115.41, 111.92 (d, *J*_FC_ = 9.9 Hz), 109.27 (d, *J*_FC_ = 25.9 Hz), 104.31, 104.12, 97.50 (d, *J*_FC_ = 4.7 Hz), 65.95, 44.01; HRMS (ESI) [M + H]^+^ Calcd for C_16_H_16_ON_4_F: 299.1303, found: 299.1301.

#### 4-(5-(5-Fluoro-1*H*-indol-2-yl)pyridin-2-yl)morpholine (**79**)

Compound **77** (0.120 g, 0.302 mmol) was dissolved in TFA (2 mL) and stirred at ambient temperature for 2 h, then the TFA was removed with N_2(g)_ flow. The residue was neutralized with aqueous NaHCO_3_ to give a solid that was placed in a filter and rinsed with a small amount of acetone to afford **79** (0.066 g, 74%) as an off-white solid: ^1^H NMR (acetone-*d*_6_, 300 MHz): δ 10.62 (s, 1H), 8.65 (dd, 1H, *J* = 2.5 Hz, *J* = 0.7 Hz), 7.98 (dd, 1H, *J* = 8.9 Hz, *J* = 2.6 Hz), 7.34 (dd, 1H, *J* = 8.8 Hz, *J* = 4.5 Hz), 7.21 (dd, 1H, *J* = 9.9 Hz, *J* = 2.5 Hz), 6.85 (m, 2H), 6.74 (s, 1H), 3.74 (m, 4H), 3.56 (m, 4 H); ^13^C NMR (125 MHz, DMSO-*d*_6_): 158.30, 157.17 (d, ^1^*J*_FC_ = 231.5 Hz), 144.57, 137.81, 134.38, 133.54, 129.07 (d, *J*_FC_ = 10.6 Hz), 117.78, 111.75 (d, *J*_FC_ = 9.9 Hz), 108.88 (d, *J*_FC_ = 26.0 Hz), 106.86, 104.05 (d, *J*_FC_ = 23.1 Hz), 97.18 (d, *J*_FC_ = 4.5 Hz), 65.90, 45.01; HRMS (ESI) [M + H]^+^ Calcd for C_17_H_17_ON_3_F: 298.1350, found: 298.1348.

#### *tert*-Butyl 6-Fluoro-2-(2-fluoro-6-(piperidin-1-yl)pyridin-3-yl)-1*H*-indole-1-carboxylate (**80**)

1,4-Dioxane (25 mL) was purged with N_2(g)_ for 1 h. Compound **58** (0.250 g, 0.965 mmol), compound **10** (0.410 g, 1.03 mmol, 1.1 equiv), Na_2_CO_3_ (0.510 g, 4.81 mmol, 5 equiv), and Pd(dppf)Cl_2_ (0.089 g, 0.12 mmol, 0.1 equiv) were flushed with N_2(g)_ for 10 min, then the 1,4-dioxane was added. The reaction mixture was stirred at reflux under N_2(g)_ for 3 h, then cooled to ambient temperature, filtered through Celite, and the Celite was rinsed with EtOAc. The filtrate was concentrated to a brown oil, then hexane was added and removed to give a brown residue that was dissolved in CH_2_Cl_2_, poured onto dry silica (55 mm h × 45 mm i.d.), and eluted under vacuum: hexane (50 mL), hexane/EtOAc/NEt_3_ v/v/v 90:8:2 (100 mL), 75:20:5 (100 mL), 50:45:5 (50 mL) to give an orange syrup (0.43 g). Purification by radial chromatography (2 mm silica): hexane/EtOAc/NEt_3_ v/v/v 95:4:1 (100 mL), 90:8:2 (50 mL) gave an off-white foam (0.16 g) that was again purified by radial chromatography (2 mm silica): %CH_2_Cl_2_/hexane–25% (50 mL), 50% (75 mL), 75% (50 mL), CH_2_Cl_2_ (25 mL) to give an off-white solid (0.14 g). A final purification by radial chromatography (2 mm silica): hexane/EtOAc/NEt_3_ v/v/v 90:8:2 (125 mL), 75:20:5 (25 mL) afforded **80** (0.130 g, 33%) as a white solid: ^1^H NMR (300 MHz, CDCl_3_) δ 7.95 (dd, 1H, *J* = 10.8 Hz, *J* = 2.1 Hz), 7.54 (dd, 1H, *J* = 9.6 Hz, *J* = 8.4 Hz), 7.44 (dd, 1H, *J* = 8.4 Hz, *J* = 5.4 Hz), 6.99 (td, 1H, *J* = 8.7 Hz, *J* = 2.4 Hz), 6.54 (d, 1H, *J* = 7.5 Hz), 6.49 (s, 1H), 3.57 (m, 4H), 1.67 (s, 6H), 1.44 (s, 9H); ^13^C NMR (100 MHz, CDCl_3_) δ 161.06 (d, *J*_FC_ = 238.0 Hz), 159.46 (d, *J*_FC_ = 234.5 Hz), 158.04 (d, *J*_FC_ = 16.2 Hz), 150.10, 141.80 (d, *J*_FC_ = 4.8 Hz), 137.51 (d, *J*_FC_ = 12.8 Hz), 134.52 (t, *J*_FC_ = 4.3 Hz), 125.55 (d, *J*_FC_ = 1.1 Hz), 120.95 (d, *J*_FC_ = 9.8 Hz), 111.16 (d, *J*_FC_ = 24.1 Hz), 109.84, 103.46 (d, *J*_FC_ = 31.3 Hz), 103.11 (d, *J*_FC_ = 28.6 Hz), 102.25 (d, *J*_FC_ = 3.8 Hz), 84.08, 46.45, 27.93, 25.55, 24.84; HRMS (ESI) [M + H]^+^ Calcd for C_23_H_26_F_2_N_3_O_2_: 414.1988, found: 414.1989.

#### 6-Fluoro-2-(2-fluoro-6-(piperidin-1-yl)pyridin-3-yl)-1*H*-indole (**81**)

Compound **80** (0.092 g, 0.22 mmol) was dissolved in TFA (2.2 mL, 29 mmol, 128 equiv), stirred at ambient temperature for 20 min, then poured into a mixture of NaHCO_3_ (2.68 g, 31.9 mmol, 1.1 equiv TFA), H_2_O (55 mL), and CH_2_Cl_2_ (55 mL). The mixture was stirred for 10 min, then the layers were separated, and the H_2_O layer was extracted with CH_2_Cl_2_ (25 mL). The combined CH_2_Cl_2_ layers were washed with H_2_O (50 mL), brine (50 mL), and dried over MgSO_4_. The solution was concentrated, poured onto dry silica (55 mm h × 45 mm i.d.), and eluted under vacuum: hexane (50 mL), hexane/EtOAc/NEt_3_ v/v/v 75:20:5 (200 mL), 50:45:5 (150 mL) to give an off-white/tan solid (68 mg). The solid was suspended in CHCl_3_ (1 mL), filtered, rinsed with CHCl_3_ (1 mL × 3), then hexane (5 mL), and dried under vacuum to afford **81** (0.049 g, 70%) as a white solid: ^1^H NMR (300 MHz, DMSO-*d*_6_) δ 11.36 (s, 1H), 8.08 (dd, 1H, *J* = 10.8 Hz, *J* = 8.7 Hz), 7.49 (dd, 1H, *J* = 8.7 Hz, *J* = 5.7 Hz), 7.13 (dd, 1H, *J* = 9.9 Hz, *J* = 1.8 Hz), 6.84 (m, 2H), 6.69 (s, 1H), 3.56 (m, 4H), 1.59 (m, 6 H); ^13^C NMR (125 MHz, DMSO-*d*_6_) δ 158.71 (d, ^1^*J*_FC_ = 232.9 Hz), 157.84 (d, ^1^*J*_FC_ = 234.8 Hz), 157.05 (d, *J*_FC_ = 16.3 Hz), 139.06 (d, *J*_FC_ = 4.4 Hz), 136.33 (d, *J*_FC_ = 12.9 Hz), 132.23 (dd, *J*_FC_ = 7.3 Hz, *J*_FC_ = 3.6 Hz), 125.34, 120.53 (d, *J*_FC_ = 10.0 Hz), 107.61 (d, *J*_FC_ = 24.3 Hz), 103.91 (d, *J*_FC_ = 3.5 Hz), 100.40 (d, *J*_FC_ = 27.9 Hz), 99.70 (d, *J*_FC_ = 8.0 Hz), 97.03 (d, *J*_FC_ = 25.8 Hz), 45.41, 24.92, 24.06; HRMS (ESI) [M + H]^+^ Calcd for C_18_H_18_F_2_N_3_: 314.1463, found: 314.1454.

#### (1-(*tert*-Butoxycarbonyl)-6-fluoro-1*H*-indol-2-yl)boronic Acid (**82**)

Compound **110** (2.350 g, 9.989 mmol) was dissolved in THF (100 mL) under N_2(g)_, cooled to 0 °C, then triisopropyl borate (2.820 g, 14.99 mmol) was added. LDA (2 M THF/heptane/ethylbenzene, 6.5 mL, 13 mmol) was added dropwise over a period of 10 min, the reaction mixture was stirred at 0 °C for 2 h, then quenched with 1 M HCl_(aq)_. The reaction mixture was extracted with EtOAc (100 mL × 3), and the combined extracts were washed with H_2_O (50 mL × 2), dried over MgSO_4_, filtered, and evaporated to dryness. The residue was recrystallized from 1:2 v/v hexane/EtOAc to afford **82** (1.780 g, 64%) as a white solid: ^1^H NMR (300 MHz, CDCl_3_): δ 7.72 (dd, 1H, *J* = 11.1 Hz, *J* = 2.4 Hz), 7.52 (dd, 1H, *J* = 8.4 Hz, *J* = 5.7 Hz), 7.45 (d, 1H, *J* = 0.6 Hz), 7.02 (td, 1H, *J* = 8.7 Hz, *J* = 2.4 Hz) 6.88 (br s, 2H), 1.75 (s, 9H). Compound **82** decomposes in the mass spectrometer under both positive ion mode and negative ion mode.

#### *tert*-Butyl 6-Fluoro-2-(5-morpholinopyrazin-2-yl)-1*H*-indole-1-carboxylate (**83**)

Compound **82** (0.210 g, 0.752 mmol), compound **36** (0.100 g, 0.410 mmol), Pd(dppf)Cl_2_ (0.020 g, 0.027 mmol), 1,4-dioxane (10 mL), and K_2_CO_3(aq)_ (2 M, 0.5 mL) were flushed with N_2(g)_ for 10 min, then stirred under N_2(g)_ at 100 °C overnight. The reaction mixture was cooled to ambient temperature, evaporated to dryness, the residue was dissolved in EtOAc (30 mL), washed with H_2_O (10 mL), dried over MgSO_4_, and filtered. The filtrate was passed through a silica gel pad, the silica was washed with EtOAc, and the filtrate was concentrated to dryness. The residue was purified by radial chromatography (hexane/EtOAc v/v 90:10 to 80:20) to afford **83** (0.078 g, 48%) as a solid: ^1^H NMR (300 MHz, CDCl_3_) δ 8.27 (d, 1H, *J* = 1.2 Hz), 8.17 (d, 1H, *J* = 1.5 Hz), 7.88 (dd, 1H, *J* = 10.8 Hz, *J* = 2.4 Hz), 7.48 (dd, 1H, *J* = 8.7 Hz, *J* = 5.7 Hz), 7.01 (td, 1H, *J* = 9.0 Hz, *J* = 2.4 Hz), 6.69 (s, 1H), 3.86 (apparent t, 4H, *J* = 4.8 Hz), 3.62 (apparent t, 4H, *J* = 4.8 Hz), 1.44 (s, 9H); HRMS (ESI) [M + H]^+^ Calcd for C_21_H_24_O_3_N_4_F: 399.1827, found: 399.1829.

#### *tert*-Butyl 6-Fluoro-2-(4-fluoro-6-morpholinopyridin-3-yl)-1*H*-indole-1-carboxylate (**84**)

Compound **82** (0.340 g, 1.22 mmol), compound **63** (0.150 g, 0.575 mmol), Pd(dppf)Cl_2_ (0.028 g, 0.038 mmol), 1,4-dioxane (30 mL), and K_2_CO_3(aq)_ (2 M, 0.7 mL) were flushed with N_2(g)_ for 5 min, then stirred under N_2(g)_ at 100 °C overnight (TLC indicated unreacted **63** remaining). Additional **82** (0.150 g, 0.537 mmol) was added and the reaction mixture was stirred under N_2(g)_ at 100 °C overnight, then cooled to ambient temperature and evaporated to dryness. The residue was dissolved in H_2_O, extracted with EtOAc (10 mL × 3), and the combined extracts were dried over MgSO_4_, and filtered. The filtrate was passed through a silica gel pad, the silica was washed with EtOAc, and the solvent was removed to give a residue that was purified by radial chromatography (hexane/EtOAc v/v 99:1 to 80:20) to afford **84** (0.150 g, 63%) as a solid: ^1^H NMR (500 MHz, CDCl_3_) δ 8.20 (d, 1H, *J* = 10.5 Hz), 7.92 (dd, 1H, *J* = 10.5 Hz, *J* = 1.5 Hz), 7.47 (dd, 1H, *J* = 9.0 Hz, *J* = 5.5 Hz), 7.01 (td, 1H, *J* = 9.0 Hz, *J* = 2.5 Hz), 6.53 (s, 1H), 6.36 (d, 1H, *J* = 12.5 Hz), 3.83 (t, 4H, *J* = 5.0 Hz), 3.56 (partially resolved m, 4H), 1.47 (s, 9H); HRMS (ESI) [M + H]^+^ Calcd for C_22_H_24_O_3_N_3_F_2_: 416.1780, found: 416.1793.

#### *tert*-Butyl 6-Fluoro-2-(5-fluoro-6-(piperidin-1-yl)pyridin-3-yl)-1*H*-indole-1-carboxylate (**85**)

Compound **82** (0.060 g, 0.21 mmol), compound **64** (0.053 g, 0.21 mmol), Pd(dppf)Cl_2_ (0.010 g, 0.014 mmol), 1,4-dioxane (6 mL), and K_2_CO_3(aq)_ (2 M, 0.3 mL) were flushed with N_2(g)_ for 5 min, then stirred under N_2(g)_ at 100 °C overnight (TLC indicated unreacted **64** remaining). Additional **82** (0.064 g, 0.23 mmol) was added and the reaction mixture was stirred under N_2(g)_ at 100 °C overnight, then cooled to ambient temperature and evaporated to dryness. The residue was dissolved in H_2_O, extracted with EtOAc (10 mL × 3), and the combined extracts were dried over MgSO_4_, and filtered. The filtrate was passed through a silica gel pad, the silica was washed with EtOAc, and the solvent was removed to give a residue that was purified by radial chromatography (hexane/EtOAc v/v 99:1 to 80:20) to afford **85** (0.059 g, 70%) as a solid: ^1^H NMR (300 MHz, CDCl_3_) δ 8.00 (m, 1H), 7.89 (dd, 1H, *J* = 10.8 Hz, *J* = 1.8 Hz), 7.41 (m, 1H), 7.21 (dd, 1H, *J* = 13.8 Hz, *J* = 1.8 Hz), 6.96 (tt, 1H, *J* = 9.0 Hz, *J* = 2.1 Hz), 6.47 (s, 1H), 3.45 (m, 4H), 1.64 (m, 6H), 1.39 (s, 9H); HRMS (ESI) [M + H]^+^ Calcd for C_23_H_26_O_2_N_3_F_2_: 414.1988, found: 414.1988.

#### *tert*-Butyl 6-Fluoro-2-(5-fluoro-6-morpholinopyridin-3-yl)-1*H*-indole-1-carboxylate (**86**)

Compound **82** (0.190 g, 0.681 mmol), compound **65** (0.077 g, 0.295 mmol), Pd(dppf)Cl_2_ (0.012 g, 0.016 mmol), 1,4-dioxane (10 mL), and K_2_CO_3(aq)_ (2 M, 0.5 mL) were flushed with N_2(g)_ for 10 min, then stirred under N_2(g)_ at 100 °C overnight. The reaction mixture was cooled to ambient temperature, evaporated to dryness, the residue was dissolved in H_2_O, and extracted with EtOAc (10 mL × 3). The combined extracts were dried over MgSO_4_ and filtered, the filtrate was passed through a silica gel pad, and the silica was washed with EtOAc. The solvent was removed to give a residue that was purified by radial chromatography (hexane/EtOAc v/v 95:5 to 80:20) to afford **86** (0.086 g, 70%) as a solid: ^1^H NMR (400 MHz, CDCl_3_): δ 8.07 (s, 1H), 7.92 (d, 1H, *J* = 10.8 Hz), 7.46 (dd, 1H, *J* = 8.8 Hz, *J* = 5.6 Hz), 7.30 (d, 1H, *J* = 13.6 Hz), 7.02 (unresolved apparent td, 1H, *J* = 8.8 Hz), 6.53 (s, 1H), 3.86 (t, 4H, *J* = 4.4 Hz), 3.54 (t, 4H, *J* = 4.4 Hz), 1.45 (s, 9H); HRMS (ESI) [M + H]^+^ Calcd for C_22_H_24_O_3_N_3_F_2_: 416.1780, found: 416.1781.

#### 4-(5-(6-Fluoro-1*H*-indol-2-yl)pyrazin-2-yl)morpholine (**87**)

Compound **83** (0.078 g, 0.20 mmol) was dissolved in TFA (2 mL), stirred at ambient temperature for 2 h, then the TFA was removed with N_2(g)_ flow. The residue was neutralized with aqueous NaHCO_3_ to give a solid that was washed with cold CH_2_Cl_2_ to afford **87** (0.058 g, 99%) as an off-white solid: ^1^H NMR (500 MHz, acetone-*d*_6_) δ 10.75 (br s, 1H), 8.72 (d, 1H, *J* = 1.0 Hz), 8.26 (d, 1H, *J* = 1.5 Hz), 7.55 (dd, 1H, *J* = 9.0 Hz, *J* = 5.5 Hz), 7.23 (dd, 1H, *J* = 10.0 Hz, *J* = 2.0 Hz), 6.84 (ddd, 1H, *J* = 10.0 Hz, *J* = 8.5 Hz, *J* = 2.0 Hz), 3.79 (t, 4H, *J* = 5.0 Hz), 3.62 (t, 4H, *J* = 5.0 Hz); ^13^C NMR (125 MHz, DMSO-*d*_6_): 159.06 (d, ^1^*J*_FC_ = 234.3 Hz), 153.41, 138.40, 136.93 (d, *J*_FC_ = 13.2 Hz), 136.29, 135.17, 129.82, 125.49, 121.08 (d, *J*_FC_ = 9.6 Hz), 107.89 (d, *J*_FC_ = 24.9 Hz), 98.09, 97.51 (d, *J*_FC_ = 26.0 Hz), 65.81, 44.54; HRMS (ESI) [M + H]^+^ Calcd for C_16_H_16_ON_4_F: 299.1303, found: 299.1302.

#### 4-(4-Fluoro-5-(6-fluoro-1*H*-indol-2-yl)pyridin-2-yl)morpholine (**88**)

Compound **84** (0.150 g, 0.361 mmol) was dissolved in TFA (2 mL), stirred at ambient temperature for 1.5 h, then the TFA was removed with N_2(g)_ flow. The residue was neutralized with aqueous NaHCO_3_ to give a solid that was washed with cold CH_2_Cl_2_ to afford **88** (0.110 g, 97%) as an off-white solid: ^1^H NMR (300 MHz, CDCl_3_) δ 8.71 (br s, 1H), 8.61 (d, 1H, *J* = 11.1 Hz), 7.52 (dd, 1H, *J* = 8.7 Hz, *J* = 5.4 Hz), 7.08 (dd, 1H, *J* = 9.6 Hz, *J* = 2.1 Hz), 6.89 (ddd, 1H, *J* = 9.9 Hz, *J* = 8.7 Hz, *J* = 2.1 Hz), 6.80 (dd, 1H, *J* = 1.2 Hz), 6.40 (d, 1H, *J* = 14.7 Hz), 3.85 (apparent t, 4H, *J* = 4.8 Hz), 3.57 (apparent t, 4H, *J* = 4.8 Hz); ^13^C NMR (125 MHz, DMSO-*d*_6_): 166.26 (d, ^1^*J*_FC_ = 257.9 Hz), 159.88 (d, *J*_FC_ = 4.5 Hz), 158.99 (d, *J*_FC_ = 241.6 Hz), 147.42 (d, *J*_FC_ = 4.9 Hz), 136.47 (d, *J*_FC_ = 12.7 Hz), 130.55, 125.23, 120.82 (d, *J*_FC_ = 10.1 Hz), 107.83 (d, *J*_FC_ = 24.4 Hz), 107.36 (d, *J*_FC_ = 10.9 Hz), 100.68 (d, *J*_FC_ = 7.0 Hz), 97.18 (d, *J*_FC_ = 25.7 Hz), 93.74 (d, *J*_FC_ = 22.6 Hz), 65.83, 45.03; HRMS (ESI) [M + H]^+^ Calcd for C_17_H_16_ON_3_F_2_: 316.1256, found: 316.1246.

#### 6-Fluoro-2-(5-fluoro-6-(piperidin-1-yl)pyridin-3-yl)-1*H*-indole (**89**)

Compound **85** (0.059 g, 0.14 mmol) was dissolved in TFA (1 mL), stirred at ambient temperature for 2.5 h, then the TFA was removed with N_2(g)_ flow. The residue was neutralized with aqueous NaHCO_3_ to give a solid that was purified by radial chromatography (hexanes/EtOAc v/v 95:5 to 80:20), then purified again by radial chromatography (CHCl_3_) to afford **89** (0.008 g, 18%) as an off-white solid: ^1^H NMR (300 MHz, acetone-*d*_6_) δ 10.74 (br s, 1H), 8.47 (t, 1H, *J* = 1.8 Hz), 7.78 (dd, 1H, *J* = 14.7 Hz, *J* = 2.1 Hz), 7.53 (dd, 1H, *J* = 8.7 Hz, *J* = 5.4 Hz), 7.10 (dd, 1H, *J* = 9.9 Hz, *J* = 2.4 Hz), 6.86 (m, 1H), 6.84 (ddd, 1H, *J* = 9.9 Hz, *J* = 8.4 Hz, *J* = 2.4 Hz), 3.51 (m, 4H), 1.67 (m, 6 H); ^13^C NMR (125 MHz, DMSO-*d*_6_): 159.12 (d, *J*_FC_ = 235.1 Hz), 149.17 (d, *J*_FC_ = 255.3 Hz), 148.54 (d, *J*_FC_ = 6.4 Hz), 139.22 (d, *J*_FC_ = 4.5 Hz), 137.06 (d, *J*_FC_ = 12.7 Hz), 135.17, 125.53, 120.91 (d, *J*_FC_ = 10.1 Hz), 120.73, 119.83 (d, *J*_FC_ = 20.8 Hz), 107.98 (d, *J*_FC_ = 24.4 Hz), 98.56, 97.22 (d, *J*_FC_ = 25.5 Hz), 48.37 (d, *J*_FC_ = 5.7 Hz), 25.49, 24.31; HRMS (ESI) [M + H]^+^ Calcd for C_18_H_18_N_3_F_2_: 314.1463, found: 314.1455.

#### 4-(3-Fluoro-5-(6-fluoro-1*H*-indol-2-yl)pyridin-2-yl)morpholine (**90**)

Compound **86** (0.080 g, 0.19 mmol) was dissolved in TFA (2 mL), stirred at ambient temperature for 1.5 h, then the TFA was removed with N_2(g)_ flow. The residue was neutralized with aqueous NaHCO_3_ to give a solid that was washed with cold CH_2_Cl_2_ to afford **90** (0.060 g, 98%) as an off-white solid: ^1^H NMR (300 MHz, acetone-*d*_6_) δ 10.79 (br s, 1H), 8.51 (apparent t, 1H, *J* = 1.8 Hz), 7.84 (dd, 1H, *J* = 14.4 Hz, *J* = 1.8 Hz), 7.54 (dd, 1H, *J* = 8.7 Hz, *J* = 5.4 Hz), 7.11 (apparent dd, 1H, *J* = 9.9 Hz, *J* = 2.4 Hz), 6.89 (m, 1H), 6.85 (ddd, 1H, *J* = 9.9 Hz, *J* = 8.7 Hz, *J* = 2.4 Hz), 3.78 (apparent t, 4H, *J* = 4.8 Hz), 3.50 (apparent t, 4H, *J* = 4.8 Hz); ^13^C NMR (125 MHz, DMSO-*d*_6_): 159.01 (d, *J*_FC_ = 235.2 Hz), 149.21 (d, *J*_FC_ = 255.4 Hz), 147.86 (d, *J*_FC_ = 6.8 Hz), 139.13 (d, *J*_FC_ = 4.7 Hz), 136.94 (d, *J*_FC_ = 12.7 Hz), 134.79 (d, *J*_FC_ = 3.2 Hz), 125.33, 121.48 (d, *J*_FC_ = 3.0 Hz), 120.90 (d, *J*_FC_ = 10.1 Hz), 119.98 (d, *J*_FC_ = 20.6 Hz), 107.98 (d, *J*_FC_ = 24.4 Hz), 98.82, 97.17 (d, *J*_FC_ = 25.5 Hz), 65.97, 47.67 (d, *J*_FC_ = 5.4 Hz); HRMS (ESI) [M + H]^+^ Calcd for C_17_H_16_ON_3_F_2_: 316.1256, found: 316.1253.

#### *tert*-Butyl 2-(4-Fluoro-6-morpholinopyridin-3-yl)-1*H*-indole-1-carboxylate (**91**)

Compound **12** (0.300 g, 1.15 mmol), compound **63** (0.150 g, 0.575 mmol), Pd(dppf)Cl_2_ (0.028 g, 0.038 mmol), 1,4-dioxane (30 mL), and K_2_CO_3(aq)_ (2 M, 0.7 mL) were flushed with N_2(g)_ for 10 min, stirred under N_2(g)_ at 100 °C overnight, then cooled to ambient temperature, and evaporated to dryness. The residue was dissolved in H_2_O and extracted with EtOAc (10 mL × 3), and the extracts were combined, dried over MgSO_4_, and filtered. The filtrate was passed through a silica gel pad, and the silica was washed with EtOAc. The solvent was removed to give a residue that was purified by radial chromatography (hexane/EtOAc v/v 95:5 to 80:20) to afford **91** (0.160 g, 70%) as a solid: ^1^H NMR (300 MHz, CDCl_3_) δ 8.22 (d, 1H, *J* = 10.5 Hz), 8.19 (d, 1H, *J* = 7.5 Hz), 7.56 (dm, 1H, *J* = 7.5 Hz), 7.33 (ddd, 1H, *J* = 8.4 Hz, *J* = 7.2 Hz, *J* = 1.5 Hz), 7.24 (td, 1H, *J* = 7.5 Hz, *J* = 1.2 Hz), 6.57 (s, 1H), 6.36 (d, 1H, *J* = 12.9 Hz), 3.83 (apparent t, 4H, *J* = 4.8 Hz), 3.56 (apparent t, 4H, *J* = 4.8 Hz), 1.48 (s, 9H); HRMS (ESI) [M + H]^+^ Calcd for C_22_H_25_O_3_N_3_F: 398.1875, found: 398.1887.

#### *tert*-Butyl 2-(5-Fluoro-6-(piperidin-1-yl)pyridin-3-yl)-1*H*-indole-1-carboxylate (**92**)

Compound **12** (0.090 g, 0.34 mmol), compound **64** (0.090 g, 0.35 mmol), Pd(dppf)Cl_2_ (0.019 g, 0.026 mmol), 1,4-dioxane (10 mL), and K_2_CO_3(aq)_ (2 M, 0.5 mL) were flushed with N_2(g)_ for 5 min, then stirred under N_2(g)_ at 100 °C overnight (TLC indicated unreacted **64** remaining). Additional **12** (0.085 g, 0.33 mmol) was added, and the reaction mixture was stirred under N_2(g)_ at 100 °C for 5 h, then cooled to ambient temperature and evaporated to dryness. The residue was dissolved in H_2_O and extracted with EtOAc (10 mL × 3), and the extracts were combined, dried over MgSO_4_, and filtered. The filtrate was passed through a silica gel pad, and the silica was washed with EtOAc. The solvent was removed to give a residue that was purified by radial chromatography (hexane/EtOAc v/v 95:5 to 80:20) to afford **92** (0.120 g, 87%) as a solid:

^1^H NMR (300 MHz, CDCl_3_) δ 8.19 (d, 1H, *J* = 8.4 Hz), 8.07 (t, 1H, *J* = 1.5 Hz), 7.54 (unresolved apparent dd, 1H, *J* = 7.8 Hz), 7.33 (partially resolved ddd, 1H, *J* = 8.1 Hz, *J* = 7.2 Hz, *J* = 1.2 Hz), 7.26 (m–obscured by CHCl_3_ resonance, 3 H) 6.55 (s, 1H), 3.50 (m, 4H), 1.69 (m, 6H), 1.44 (s, 9H); HRMS (ESI) [M + H]^+^ Calcd for C_23_H_27_O_2_N_3_F: 396.2082, found: 396.2082.

#### *tert*-Butyl 2-(5-Fluoro-6-morpholinopyridin-3-yl)-1*H*-indole-1-carboxylate (**93**)

Compound **12** (0.170 g, 0.651 mmol), compound **65** (0.075 g, 0.29 mmol), Pd(dppf)Cl_2_ (0.011 g, 0.015 mmol), 1,4-dioxane (10 mL), and K_2_CO_3(aq)_ (2 M, 0.5 mL) were flushed with N_2(g)_ for 5 min, then stirred under N_2(g)_ at 100 °C overnight. The reaction mixture was cooled to ambient temperature and evaporated to dryness. The residue was dissolved in H_2_O and extracted with EtOAc (10 mL × 3), and the extracts were dried over MgSO_4_ and filtered. The filtrate was passed through a silica gel pad, and the silica was washed with EtOAc. The solvent was removed to give a residue that was purified by radial chromatography (hexane/EtOAc v/v 95:5 to 80:20) to afford **93** (0.092 g, 80%) as an off-white solid: ^1^H NMR (400 MHz, CDCl_3_) δ 8.18 (d, 1H, *J* = 8.4 Hz), 8.09 (s, 1H), 7.55 (d, 1H, *J* = 7.6 Hz), 7.33 (m, 2H), 7.26 (m–obscured by CHCl_3_ resonance, 1H), 6.57 (s, 1H), 3.86 (t, 4H, *J* = 4.4 Hz), 3.54 (t, 4H, *J* = 4.4 Hz), 1.45 (s, 9H); HRMS (ESI) [M + H]^+^ Calcd for C_22_H_25_O_3_N_3_F: 398.1875, found: 398.1863.

#### 4-(4-Fluoro-5-(1*H*-indol-2-yl)pyridin-2-yl)morpholine (**94**)

Compound **91** (0.054 g, 0.14 mmol) was dissolved in TFA (2 mL), stirred at ambient temperature for 2 h, then the TFA was removed with N_2(g)_ flow. The residue was neutralized with aqueous NaHCO_3_ solution to give a solid that was washed with cold CH_2_Cl_2_, then purified by radial chromatography (hexane/EtOAc v/v 90:10 to 80:20) to afford **94** (0.030 g, 74%) as an off-white solid: ^1^H NMR (300 MHz, acetone-*d*_6_) δ 10.52 (br s, 1H), 8.66 (d, 1H, *J* = 11.4 Hz), 7.56 (d, 1H, *J* = 8.1 Hz), 7.42 (d, 1H, *J* = 8.1 Hz), 7.10 (partially resolved ddd, 1H, *J* = 8.1 Hz, *J* = 6.9 Hz, *J* = 1.2 Hz), 7.02 (partially resolved ddd, 1H, *J* = 7.8 Hz, *J* = 7.2 Hz, *J* = 1.2 Hz), 6.81 (m, 1H), 6.71 (d, 1H, *J* = 14.7 Hz), 3.76 (apparent t, 4H, *J* = 4.8 Hz), 3.59 (apparent t, 4H, *J* = 4.8 Hz); ^13^C NMR (125 MHz, DMSO-*d*_6_) δ 166.33 (d, *J*_FC_ = 257.8 Hz), 159.93 (d, *J*_FC_ = 11.4 Hz), 147.61 (d, *J*_FC_ = 3.8 Hz), 136.49, 129.80, 128.42, 121.48, 119.78, 119.32, 111.12, 107.52 (d, *J*_FC_ = 10.1 Hz), 100.69 (d, *J*_FC_ = 6.3 Hz), 93.66 (d, *J*_FC_ = 22.5 Hz), 65.81, 45.00; HRMS (ESI) [M + H]^+^ Calcd for C_17_H_17_ON_3_F: 298.1350, found: 298.1343.

#### 2-(5-Fluoro-6-(piperidin-1-yl)pyridin-3-yl)-1*H*-indole (**95**)

Compound **92** (0.110 g, 0.278 mmol) was dissolved in TFA (2 mL), stirred at ambient temperature for 2 h, then the TFA was removed with N_2(g)_ flow. The residue was neutralized with aqueous NaHCO_3_ to give a solid which was washed with cold CH_2_Cl_2_, then purified by radial chromatography (hexane/EtOAc v/v 90:10) to afford **95** (0.066 g, 80%) as an off-white solid: ^1^H NMR (300 MHz, acetone-*d*_6_) δ 10.61 (br s, 1H), 8.50 (t, 1H, *J* = 1.8 Hz), 7.80 (dd, 1H, *J* = 14.7 Hz, *J* = 1.8 Hz), 7.54 (apparent d, 1H, *J* = 8.1 Hz), 7.38 (dd, 1H, *J* = 8.7 Hz, *J* = 0.6 Hz), 7.10 (ddd, 1H, *J* = 8.1 Hz, *J* = 6.9 Hz, *J* = 1.2 Hz), 7.02 (ddd, 1H, *J* = 8.1 Hz, *J* = 6.9 Hz, *J* = 1.2 Hz), 6.85 (m, 1H), 3.50 (m, 4H), 1.67 (m, 6 H); ^13^C NMR (125 MHz, DMSO-*d*_6_): 149.03 (d, *J*_FC_ = 255.2 Hz), 148.34 (d, *J*_FC_ = 6.4 Hz), 139.27 (d, *J*_FC_ = 4.6 Hz), 137.00, 134.25, 128.59, 121.49, 120.81 (d, *J*_FC_ = 2.8 Hz), 119.82 (d, *J*_FC_ = 20.8 Hz), 119.80, 119.43, 111.08, 98.44, 48.25 (d, *J*_FC_ = 5.8 Hz), 25.37, 24.19; HRMS (ESI) [M + H]^+^ Calcd for C_18_H_19_N_3_F: 296.1558, found: 296.1550.

#### 4-(3-Fluoro-5-(1*H*-indol-2-yl)pyridin-2-yl)morpholine (**96**)

Compound **93** (0.090 g, 0.23 mmol) was dissolved in TFA (2 mL), stirred at ambient temperature for 1 h, then the TFA was removed with N_2(g)_ flow. The residue was neutralized with aqueous NaHCO_3_ to give a solid that was purified by radial chromatography twice (hexane/EtOAc v/v 95:5 to 90:10) to afford **96** (0.025 g, 37%) as a solid: ^1^H NMR (300 MHz, acetone-*d*_6_) δ 10.65 (br s, 1H), 8.54 (apparent t, 1H, *J* = 1.8 Hz), 7.86 (dd, 1H, *J* = 14.4 Hz, *J* = 1.8 Hz), 7.55 (d, 1H, *J* = 7.8 Hz), 7.39 (partially resolved dd, 1H, *J* = 8.1 Hz, *J* = 0.9 Hz), 7.11 (ddd, 1H, *J* = 8.7 Hz, *J* = 6.9 Hz, *J* = 1.2 Hz), 7.02 (partially resolved ddd, 1H, *J* = 8.1 Hz, *J* = 7.8 Hz, *J* = 1.2 Hz), 6.88 (m, 1H), 3.78 (apparent t, 4H, *J* = 4.8 Hz), 3.50 (apparent t, 4H, *J* = 4.8 Hz); ^13^C NMR (125 MHz, DMSO-*d*_6_): 149.29 (d, *J*_FC_ = 255.4 Hz), 147.87 (d, *J*_FC_ = 6.9 Hz), 139.24 (d, *J*_FC_ = 4.8 Hz), 137.09, 136.93, 133.83 (d, *J*_FC_ = 10.2 Hz), 128.49 (d, *J*_FC_ = 10.4 Hz), 121.65, 120.08 (d, *J*_FC_ = 20.5 Hz), 119.88, 119.49, 111.10, 98.78, 66.01, 47.73 (d, *J*_FC_ = 5.4 Hz); HRMS (ESI) [M + H]^+^ Calcd for C_17_H_17_ON_3_F: 298.1350, found: 298.1348.

#### *tert*-Butyl 2-(3-Fluoro-5-(piperidin-1-yl)pyridin-2-yl)-1*H*-indole-1-carboxylate (**97**)

Compound **12** (0.220 g, 0.843 mmol), compound **66** (0.090 g, 0.42 mmol), Pd(dppf)Cl_2_ (0.019 g, 0.026 mmol), 1,4-dioxane (10 mL), and K_2_CO_3(aq)_ (2 M, 0.5 mL) were flushed with N_2(g)_ for 5 min, then stirred under N_2(g)_ at 100 °C overnight (TLC indicated unreacted **66** remaining). Additional **12** (0.090 g, 0.35 mmol) was added and stirring under N_2(g)_ at 100 °C was continued overnight, then the reaction mixture was cooled to ambient temperature and evaporated to dryness. The residue was dissolved in H_2_O and extracted with EtOAc (10 mL × 3), and the extracts were dried over MgSO_4_ and filtered. The filtrate was passed through a silica gel pad, and the silica was washed with EtOAc. The solvent was removed to give a residue that was purified by radial chromatography (hexane/EtOAc v/v 95:5 to 80:20) to afford **97** (0.075 g, 45%) as a solid: ^1^H NMR (300 MHz, CDCl_3_) δ 8.19 (dd, 1H, *J* = 8.4 Hz, *J* = 0.6 Hz), 8.15 (dd, 1H, *J* = 2.7 Hz, *J* = 1.5 Hz), 7.57 (d, 1H, *J* = 7.5 Hz), 7.33 (ddd, 1H, *J* = 8.4 Hz, *J* = 7.2 Hz, *J* = 1.2 Hz), 7.23 (apparent td–partially obscured by CHCl_3_ resonance, 1H, *J* = 7.5 Hz, *J* = 0.9 Hz), 6.91 (dd, 1H, *J* = 12.6 Hz, *J* = 2.4 Hz), 6.78 (s, 1H), 3.28 (m, 4H), 1.70 (m, 6H), 1.42 (s, 9H); HRMS (ESI) [M + H]^+^ Calcd for C_23_H_27_O_2_N_3_F: 396.2082, found: 396.2082.

#### 2-(3-Fluoro-5-(piperidin-1-yl)pyridin-2-yl)-1*H*-indole (**98**)

Compound **97** (0.075 g, 0.19 mmol) was dissolved in TFA (1 mL), stirred at ambient temperature for 1 h, then the TFA was removed with N_2(g)_ flow. The residue was neutralized with aqueous NaHCO_3_ to give a solid which was washed with cold CH_2_Cl_2_ to afford **98** (0.044 g, 78%) as an off-white solid: ^1^H NMR (300 MHz, acetone-*d*_6_) δ 10.60 (br s, 1H), 8.20 (t, 1H, *J* = 2.4 Hz), 7.58 (partially resolved dd, 1H, *J* = 8.1 Hz), 7.55 (partially resolved dd, 1H, *J* = 8.1 Hz, *J* = 0.9 Hz), 7.19 (dd, 1H, *J* = 14.7 Hz, *J* = 2.4 Hz), 7.12 (ddd, 1H, *J* = 8.1 Hz, *J* = 7.2 Hz, *J* = 1.2 Hz), 7.01 (ddd, 1H, *J* = 7.8 Hz, *J* = 7.2 Hz, *J* = 1.2 Hz), 6.94 (m, 1H), 3.37 (m, 4H), 1.70 (m, 6 H); ^13^C NMR (125 MHz, DMSO-*d*_6_): 157.07 (d, *J*_FC_ = 258.7 Hz), 147.40 (d, *J*_FC_ = 5.4 Hz), 136.20, 132.75 (d, *J*_FC_ = 7.2 Hz), 132.42, 128.70, 127.30 (d, *J*_FC_ = 12.2 Hz), 121.70, 120.22, 119.15, 111.69, 108.77 (d, *J*_FC_ = 22.6 Hz), 100.67 (d, *J*_FC_ = 11.9 Hz), 48.18, 24.72, 23.64; HRMS (ESI) [M + H]^+^ Calcd for C_18_H_19_N_3_F: 296.1558, found: 296.1562.

#### *tert*-Butyl 2-(3-Fluoro-5-morpholinopyridin-2-yl)-1*H*-indole-1-carboxylate (**99**)

Compound **12** (0.170 g, 0.651 mmol), compound **67** (0.078 g, 0.36 mmol), Pd(dppf)Cl_2_ (0.018 g, 0.025 mmol), 1,4-dioxane (10 mL), and K_2_CO_3(aq)_ (2 M, 0.5 mL) were flushed with N_2(g)_ for 5 min, then stirred under N_2(g)_ at 100 °C overnight. The reaction mixture was cooled to ambient temperature and evaporated to dryness. The residue was dissolved in H_2_O, extracted with EtOAc (10 mL × 3), and the extracts were dried over MgSO_4_ and filtered. The filtrate was passed through a silica gel pad, and the silica was washed with EtOAc. The solvent was removed to give a residue that was purified by radial chromatography (hexane/EtOAc v/v 95:5 to 80:2) to afford recovered **67** (0.058 g, 74%) and **99** (0.029 g, 20%) as a solid: ^1^H NMR (400 MHz, CDCl_3_) δ 8.18 (d, 1H, *J* = 8.4 Hz), 8.15 (s, 1H), 7.58 (d, 1H, *J* = 7.6 Hz), 7.34 (apparent t, 1H, *J* = 7.6 Hz), 7.24 (apparent t, 1H, *J* = 7.2 Hz), 6.92 (dd, 1H, *J* = 12.0 Hz, *J* = 2.0 Hz), 6.81 (s, 1H), 3.90 (t, 4H, *J* = 4.8 Hz), 3.26 (t, 4H, *J* = 4.8 Hz), 1.43 (s, 9H); HRMS (ESI) [M + H]^+^ Calcd for C_22_H_25_O_3_N_3_F: 398.1875, found: 398.1865.

#### *tert*-Butyl 6-Fluoro-2-(3-fluoro-5-morpholinopyridin-2-yl)-1*H*-indole-1-carboxylate (**100**)

Compound **82** (0.190 g, 0.681 mmol), compound **67** (0.087 g, 0.40 mmol), Pd(dppf)Cl_2_ (0.019 g, 0.026 mmol), 1,4-dioxane (10 mL), and K_2_CO_3(aq)_ (2 M, 0.5 mL) were flushed with N_2(g)_ for 5 min, then stirred under N_2(g)_ at 100 °C overnight (TLC indicated unreacted **67** remaining). Additional **82** (0.093 g, 0.33 mmol) was added, and the reaction mixture was stirred under N_2(g)_ at 100 °C for 5 h, then cooled to ambient temperature and evaporated to dryness. The residue was dissolved in H_2_O and extracted with EtOAc (10 mL × 3), and the extracts were combined, dried over MgSO_4_, and filtered. The filtrate was passed through a silica gel pad, and the silica was washed with EtOAc. The solvent was removed to give a residue that was purified by radial chromatography (hexane/EtOAc v/v 95:5 to 80:20) to afford recovered **67** (0.023 g, 26%) and **100** (0.096 g, 57%) as a solid: ^1^H NMR (400 MHz, CDCl_3_) δ 8.15 (s, 1H), 7.91 (partially resolved dd, 1H, *J* = 10.8 Hz, *J* = 1.2 Hz), 7.49 (dd, 1H, *J* = 8.4 Hz, *J* = 5.6 Hz), 7.00 (apparent td, 1H, *J* = 9.6 Hz, *J* = 1.6 Hz), 6.92 (apparent dd, 1H, *J* = 12.4 Hz, *J* = 1.6 Hz), 6.76 (s, 1H), 3.90 (t, 4H, *J* = 4.8 Hz), 3.26 (t, 4H, *J* = 4.8 Hz), 1.43 (s, 9H); HRMS (ESI) [M + H]^+^ Calcd for C_22_H_24_O_3_N_3_F_2_: 416.1780, found: 416.1767.

#### *tert*-Butyl 2-(4-Fluoro-5-morpholinopyridin-2-yl)-1*H*-indole-1-carboxylate (**101**)

Compound **12** (0.085 g, 0.33 mmol), compound **68** (0.078 g, 0.36 mmol), Pd(dppf)Cl_2_ (0.018 g, 0.025 mmol), 1,4-dioxane (5 mL), and K_2_CO_3(aq)_ (2 M, 0.5 mL) were stirred at 100 °C under N_2(g)_ overnight. The reaction mixture was cooled to ambient temperature, evaporated to dryness, and the residue was dissolved in H_2_O and extracted with EtOAc (10 mL × 3). The combined extracts were dried over MgSO_4_, filtered, and the filtrate was passed through a silica gel pad and washed with EtOAc. The solution was concentrated to dryness and the residue was purified by radial chromatography (hexane/EtOAc v/v 95:5 to 70:30) to give recovered **68** (0.032 g, 41%) and compound **101** (0.045 g, 35%): ^1^H NMR (300 MHz, CDCl_3_) δ 8.28 (d, 1H, *J* = 11.1 Hz), 8.15 (d, 1H, *J* = 8.1 Hz), 7.57 (d, 1H, *J* = 7.8 Hz), 7.35 (partially resolved ddd, 1H, *J* = 8.4 Hz, *J* = 7.2 Hz, *J* = 1.2 Hz), 7.25 (m–partially obscured by CHCl_3_ resonance, 1H), 7.19 (d, 1H, *J* = 12.9 Hz), 6.75 (s, 1H), 3.90 (apparent t, 4H, *J* = 4.5 Hz), 3.21 (apparent t, 4H, *J* = 4.5 Hz), 1.42 (s, 9H); HRMS (ESI) [M + H]^+^ Calcd for C_22_H_25_O_3_N_3_F: 398.1875, found: 398.1865.

#### *tert*-Butyl 6-Fluoro-2-(4-fluoro-5-morpholinopyridin-2-yl)-1*H*-indole-1-carboxylate (**102**)

Compound **82** (0.230 g, 0.824 mmol), compound **68** (0.097 g, 0.45 mmol), Pd(dppf)Cl_2_ (0.018 g, 0.025 mmol), 1,4-dioxane (10 mL), and K_2_CO_3(aq)_ (2 M, 0.5 mL) were flushed with N_2(g)_ for 5 min, then stirred under N_2(g)_ and heated at 100 °C overnight. The reaction mixture was cooled to ambient temperature, evaporated to dryness, the residue was dissolved in H_2_O and extracted with EtOAc (10 mL × 3). The combined extracts were dried over MgSO_4_, filtered, and the filtrate was passed through a silica gel pad and washed with EtOAc. The solution was concentrated to dryness and the residue was purified by radial chromatography (hexane/EtOAc v/v 95:5 to 80:20) to afford recovered **68** (0.068 g, 70%) and **102** (0.029 g, 16%) as a solid: ^1^H NMR (300 MHz, CDCl_3_) δ 8.27 (d, 1H, *J* = 10.8 Hz), 7.88 (dd, 1H, *J* = 10.5 Hz, *J* = 2.4 Hz), 7.48 (dd, 1H, *J* = 8.7 Hz, *J* = 5.4 Hz), 7.18 (d, 1H, *J* = 12.9 Hz), 7.01 (td, 1H, *J* = 9.0 Hz, *J* = 2.4 Hz), 6.70 (s, 1H), 3.90 (m–AA’XX’, 4H, *J* = 4.8 Hz, *J* = 1.8 Hz), 3.21 (m–AA’XX’, 4H, *J* = 4.8 Hz, *J* = 1.8 Hz), 1.41 (s, 9H); HRMS (ESI) [M + H]^+^ Calcd for C_22_H_24_O_3_N_3_F_2_: 416.1780, found: 416.1767.

#### 4-(5-Fluoro-6-(1*H*-indol-2-yl)pyridin-3-yl)morpholine (**103**)

Compound **99** (0.029 g, 0.073 mmol) was dissolved in TFA (1 mL), stirred at ambient temperature for 2 h, then the TFA was removed with N_2(g)_ flow. The residue was neutralized with aqueous NaHCO_3_ to give a solid that was washed with cold CH_2_Cl_2_ to afford **103** (0.017 g, 78%) as an off-white solid: ^1^H NMR (400 MHz, acetone-*d*_6_) δ 10.65 (br s, 1H), 8.22 (t, 1H, *J* = 2.0 Hz), 7.60 (unresolved dq, 1H, *J* = 8.0 Hz), 7.56 (partially resolved dq, 1H, *J* = 8.0 Hz, *J* = 0.8 Hz), 7.24 (dd, 1H, *J* = 14.4 Hz, *J* = 2.4 Hz), 7.14 (ddd, 1H, *J* = 8.8 Hz, *J* = 7.2 Hz, *J* = 1.2 Hz), 7.02 (ddd, 1H, *J* = 7.8 Hz, *J* = 7.2 Hz, *J* = 1.2 Hz), 6.98 (dm, 1H, *J* = 4.4 Hz), 3.81 (apparent t, 4H, *J* = 4.8 Hz), 3.32 (partially resolved m–AA’XX’, 4H, *J* = 4.8 Hz); ^13^C NMR (125 MHz, DMSO-*d*_6_): 156.94 (d, *J*_FC_ = 258.6 Hz), 147.20 (d, *J*_FC_ = 4.3 Hz), 136.24, 132.53 (d, *J*_FC_ = 6.4 Hz), 132.14, 128.65, 128.46 (d, *J*_FC_ = 10.9 Hz), 121.88, 120.33, 119.22, 111.74, 108.95 (d, *J*_FC_ = 22.1 Hz), 101.06 (d, *J*_FC_ = 11.9 Hz), 65.69, 47.23; HRMS (ESI) [M + H]^+^ Calcd for C_17_H_17_ON_3_F: 298.1350, found: 298.1353.

#### 4-(5-Fluoro-6-(6-fluoro-1*H*-indol-2-yl)pyridin-3-yl)morpholine (**104**)

Compound **100** (0.031 g, 0.075 mmol) was dissolved in TFA (1 mL), stirred at ambient temperature for 1 h, and then the TFA was removed with N_2(g)_ flow. The residue was neutralized with aqueous NaHCO_3_ to give a solid that was washed with cold CH_2_Cl_2_ to afford **104** (0.023 g, 98%) as an off-white solid: ^1^H NMR (300 MHz, acetone-*d*_6_) δ 10.77 (br s, 1H), 8.21 (t, 1H, *J* = 1.8 Hz), 7.58 (dd, 1H, *J* = 8.7 Hz, *J* = 5.4 Hz), 7.30 (dd, 1H, *J* = 10.2 Hz, *J* = 2.4 Hz), 7.23 (dd, 1H, *J* = 14.4 Hz, *J* = 2.4 Hz), 6.96 (dm, 1H, *J* = 4.2 Hz), 6.85 (ddd, 1H, *J* = 9.9 Hz, *J* = 8.7 Hz, *J* = 2.4 Hz), 3.81 (apparent t, 4H, *J* = 4.8 Hz), 3.32 (apparent t, 4H, *J* = 4.8 Hz); ^13^C NMR (125 MHz, DMSO-*d*_6_): 159.18 (d, *J*_FC_ = 235.3 Hz), 156.82 (d, *J*_FC_ = 258.8 Hz), 147.26 (d, *J*_FC_ = 5.5 Hz), 136.24 (d, *J*_FC_ = 13.2 Hz), 133.33 (unresolved m), 132.13 (d, *J*_FC_ = 2.8 Hz), 128.15 (d, *J*_FC_ = 11.9 Hz), 125.49, 121.46 (d, *J*_FC_ = 10.2 Hz), 108.93 (d, *J*_FC_ = 22.8 Hz), 107.83 (d, *J*_FC_ = 24.5 Hz), 101.06 (d, *J*_FC_ = 12.1 Hz), 97.51 (d, *J*_FC_ = 25.7 Hz), 65.69, 47.21; HRMS (ESI) [M + H]^+^ Calcd for C_17_H_16_ON_3_F_2_: 316.1256, found: 316.1258.

#### 4-(4-Fluoro-6-(1*H*-indol-2-yl)pyridin-3-yl)morpholine (**105**)

Compound **101** (0.040 g, 0.10 mmol) was dissolved in TFA (1.5 mL), stirred at ambient temperature for 1.5 h, then the TFA was removed with N_2(g)_ flow. The residue was neutralized with aqueous NaHCO_3_ to give a solid that was washed with hexanes and cold CH_2_Cl_2_ to afford **105** (0.029 g, 97%) as an off-white solid: ^1^H NMR (300 MHz, acetone-*d*_6_) δ 10.73 (br s, 1H), 8.28 (d, 1H, *J* = 11.1 Hz), 7.72 (d, 1H, *J* = 13.8 Hz), 7.58 (d, 1H, *J* = 8.1 Hz), 7.53 (d, 1H, *J* = 8.1 Hz, *J* = 0.9 Hz), 7.14 (ddd, 1H, *J* = 8.1 Hz, *J* = 7.2 Hz, *J* = 1.2 Hz), 7.07 (m, 1H), 7.02 (ddd, 1H, *J* = 7.8 Hz, *J* = 7.2 Hz, *J* = 0.9 Hz), 3.82 (m–AA’XX’, 4H, *J* = 4.8 Hz, *J* = 1.8 Hz), 3.19 (m–AA’XX’, 4H, *J* = 4.8 Hz, *J* = 1.8 Hz); ^13^C NMR (125 MHz, DMSO-*d*_6_): 160.55 (d, *J*_FC_ = 258.7 Hz), 146.18 (d, *J*_FC_ = 8.0 Hz), 140.75 (unresolved d), 137.10, 136.37 (unresolved d), 134.62 (d, *J*_FC_ = 5.2 Hz), 128.33, 122.15, 120.43, 119.43, 111.87, 107.55 (d, *J*_FC_ = 18.4 Hz), 100.24, 65.99, 50.08; HRMS (ESI) [M + H]^+^ Calcd for C_17_H_17_ON_3_F: 298.1350, found: 298.1347.

#### 4-(4-Fluoro-6-(6-fluoro-1*H*-indol-2-yl)pyridin-3-yl)morpholine (**106**)

Compound **102** (0.029 g, 0.070 mmol) was dissolved in TFA (1 mL), stirred at ambient temperature for 2 h, then the TFA was removed with N_2(g)_ flow. The residue was neutralized with aqueous NaHCO_3_ to give a solid which was washed with cold CH_2_Cl_2_ to afford **106** (0.021 g, 95%) as an off-white solid: ^1^H NMR (300 MHz, acetone-*d*_6_) δ 10.86 (br s, 1H), 8.27 (d, 1H, *J* = 11.1 Hz), 7.71 (d, 1H, *J* = 13.8 Hz), 7.57 (dd, 1H, *J* = 8.7 Hz, *J* = 5.4 Hz), 7.26 (dd, 1H, *J* = 9.9 Hz, *J* = 2.4 Hz), 7.08 (m, 1H), 6.85 (ddd, 1H, *J* = 9.9 Hz, *J* = 8.7 Hz, *J* = 2.4 Hz), 3.82 (m–AA’XX’, 4H, *J* = 4.8 Hz, *J* = 1.8 Hz), 3.19 (m–AA’XX’, 4H, *J* = 4.8 Hz, *J* = 1.8 Hz); ^13^C NMR (125 MHz, DMSO-*d*_6_): 160.56 (d, *J*_FC_ = 258.8 Hz), 159.30 (d, *J*_FC_ = 235.7 Hz), 145.85 (d, *J*_FC_ = 8.3 Hz), 140.71 (d, *J*_FC_ = 3.0 Hz), 137.16 (dd, *J*_FC_ = 3.6 Hz), 137.03 (d, *J*_FC_ = 13.2 Hz), 134.69 (d, *J*_FC_ = 5.8 Hz), 125.17, 121.59 (d, *J*_FC_ = 10.2 Hz), 108.11 (d, *J*_FC_ = 24.5 Hz), 107.46 (d, *J*_FC_ = 18.5 Hz), 100.26, 97.63 (d, *J*_FC_ = 25.8 Hz), 65.99, 50.07 (d, *J*_FC_ = 2.6 Hz); HRMS (ESI) [M + H]^+^ Calcd for C_17_H_16_ON_3_F_2_: 316.1256, found: 316.1245.

#### *tert*-Butyl 2-(2-(4-(2-(2-Fluoroethoxy)ethoxy)piperidin-1-yl)pyrimidin-5-yl)-1*H*-indole-1-carboxylate (**107**)

Compound **50** (0.079 g, 0.20 mmol) was flushed with N_2(g)_, dissolved in *N*,*N*-dimethylacetamide (DMA) (1 mL), and cooled to 0 °C. NaH (60%, 0.026 g, 0.65 mmol, 3.2 equiv) was added, the mixture was stirred at 0 °C under N_2(g)_ for 5 min, then a solution of 2-(2-fluoroethoxy)ethyl 4-methylbenzenesulfonate (0.110 g, 0.419 mmol, 2.1 equiv) in DMA (0.5 mL) was added. The reaction mixture was stirred at ambient temperature under N_2(g)_ for 2 h, then H_2_O (5 drops) was added, the mixture was stirred for 10 min, then poured into a mixture of H_2_O (20 mL), EtOAc (20 mL), hexane (10 mL). The layers were mixed and separated, and the organic layer was washed with H_2_O (10 mL × 2) and brine (10 mL) and dried over MgSO_4_. The solvent was removed to give a yellow residue (0.138 g) that was dissolved in CH_2_Cl_2_, poured onto dry silica (55 cm h × 45 cm i.d.), and eluted under vacuum: CH_2_Cl_2_ (100 mL), %MeOH/CH_2_Cl_2_–1% (100 mL), 2% (100 mL), 3% (100 mL), 5% (100 mL) to give a faint brown syrup (0.126 g). Purification by radial chromatography (1 mm silica): CH_2_Cl_2_ (50 mL), %MeOH/CH_2_Cl_2_–1% (50 mL), 2% (25 mL) afforded **107** (0.032 g, 33%) as a colorless residue: ^1^H NMR (300 MHz, CDCl_3_) δ 8.48 (s, 2H), 7.62 (d, 1H, *J* = 7.8 Hz), 7.41 (d, 1H, *J* = 8.1 Hz), 7.24 (td, 1H, *J* = 8.1 Hz, *J* = 0.9 Hz), 7.15 (partially resolved td, 1H, *J* = 7.8 Hz, *J* = 0.9 Hz), 6.50 (s, 1H), 4.86 (apparent septet, 1H, *J* = 4.2 Hz), 4.52 (apparent t, 1H, *J* = 4.2 Hz), 4.39 (m, 3H), 4.30 (t, 2 H, *J* = 6.0 Hz), 3.80 (t, 2 H, *J* = 6.0 Hz), 3.55 (m, 4H), 2.06 (m, 2H), 1.77 (m, 2H), 1.52 (s, 9H); ^13^C NMR (125 MHz, CDCl_3_) δ 160.98, 158.41, 153.18, 137.63, 136.29, 128.37, 122.06, 120.70, 120.35, 115.32, 110.13, 102.87, 83.83, 82.43 (d, *J*_FC_ = 13.8 Hz), 73.40, 70.64 (d, *J*_FC_ = 18.8 Hz), 70.01, 44.09, 41.48, 30.83, 28.02; HRMS (ESI) [M + H]^+^ Calcd for C_26_H_34_O_4_N_4_F: 485.2559, found: 485.2556.

#### 2-(2-(4-(2-(2-Fluoroethoxy)ethoxy)piperidin-1-yl)pyrimidin-5-yl)-1*H*-indole (**108**)

Compound **107** (0.031 g, 0.064 mmol) and TFA (0.65 mL, 8.4 mmol, 132 equiv) were stirred for 20 min, then poured into a mixture of NaHCO_3_ (0.790 g, 9.40 mmol, 1.1 equiv TFA), H_2_O (25 mL), and CH_2_Cl_2_ (25 mL). The mixture was stirred for 5 min, the layers were separated, and the aqueous layer was extracted with CH_2_Cl_2_ (5 mL × 2). The combined CH_2_Cl_2_ layers were washed with brine (20 mL), dried over MgSO_4_, concentrated, poured onto dry silica (33 mm h × 33 mm i.d.), and eluted under vacuum: %MeOH/CH_2_Cl_2_–1% (50 mL), 2% (75 mL), 3% (100 mL) to afford **108** (0.016 g, 65%) as an off-white solid: ^1^H NMR (MHz, CDCl_3_) δ 8.48 (s, 2H), 7.62 (d, 1H, *J* = 7.6 Hz), 7.41 (d, 1H, *J* = 8.0 Hz), 7.24 (td, 1H, *J* = 8.0 Hz, *J* = 0.8 Hz), 7.15 (partially resolved td, 1H, *J* = 7.6 Hz, *J* = 0.8 Hz), 6.50 (s, 1H), 4.48 (dt, 2 H, *J* = 13.4 Hz, *J* = 4.4 Hz), 4.44 (dt, 2 H, ^2^*J*_HF_ = 48.0 Hz, *J* = 4.4 Hz), 4.30 (t, 2 H, *J* = 6.0 Hz), 4.01 (apparent septet, 1H, *J* = 4.4 Hz), 3.80 (t, 2 H, *J* = 6.0 Hz), 3.55 (dt, 2 H, ^3^*J*_HF_ = 29.2 Hz, *J* = 4.4 Hz), 3.42 (ddd, 2 H, *J* = 13.4 Hz, *J* = 10.0 Hz, *J* = 3.6 Hz), 2.01 (m, 2H), 1.58 (m, 2H); HRMS (ESI) [M + H]^+^ Calcd for C_21_H_26_FN_4_O_2_: 385.2034, found: 385.2041.

#### 4-(5-(6-Fluoro-1-methyl-1*H*-indol-2-yl)pyrimidin-2-yl)morpholine (**109**)

Compound **8** (0.021 g, 0.070 mmol) was flushed with N_2(g)_, then dissolved in DMA (1 mL), and cooled to 0 °C. NaH (60%, 0.010 g, 0.24 mmol, 3.4 equiv) was added, the reaction mixture was stirred at 0 °C under N_2(g)_ for 18 min, then MeOTs (0.027 g, 0.14 mmol, 2 equiv) was added. The reaction mixture was warmed to ambient temperature, stirred for 2 h, then H_2_O (1 drop) was added. The reaction mixture was combined with EtOAc (15 mL), hexane (5 mL), and H_2_O (10 mL), the layers were mixed and separated, and the organic layer was washed with H_2_O (10 mL × 2) and brine (10 mL) and dried over MgSO_4_. The solvent was removed to give an off-white solid that was dissolved in CH_2_Cl_2_, poured onto dry silica (33 mm h × 33 mm i.d.), and eluted under vacuum: %MeOH/CH_2_Cl_2_–1% (100 mL), 2% (50 mL), 3% (50 mL) to afford **109** (0.019 g, 86%) as an off-white solid: ^1^H NMR (400 MHz, CDCl_3_) δ 8.46 (s, 2H), 7.52 (dd, 1H, *J* = 8.4 Hz, *J* = 5.4 Hz), 7.02 (dd, 1H, *J* = 10.0 Hz, *J* = 2.0 Hz), 6.91 (td, 1H, *J* = 9.0 Hz, *J* = 2.0 Hz), 6.49 (s, 1H), 3.88 (m, 4H), 3.81 (m, 4H), 3.67 (s, 3H); HRMS (ESI) [M + H]^+^ Calcd for C_17_H_18_FN_4_O: 313.1459, found: 313.1455.

#### *tert*-Butyl 6-Fluoro-1*H*-indole-1-carboxylate (**110**)

6-Fluoroindole (3.28 g, 24.3 mmol), Boc_2_O (6.59 g, 30.2 mmol, 1.2 equiv), *i*-Pr_2_NEt (7.0 mL, 40 mmol, 1.7 equiv), DMAP (0.310 g, 2.54 mmol, 0.1 equiv), and CH_2_Cl_2_ (210 mL) were stirred at ambient temperature under N_2(g)_ for 16 h. The reaction mixture was concentrated to a yellow/orange oil, hexane was added, and the mixture was purified by vacuum flash chromatography on silica (16 cm h × 4 cm i.d.): hexane (100 mL), %CH_2_Cl_2_/hexane–10% (200 mL), 25% (400 mL) to afford **110** (5.67 g, 99%) as a colorless oil: ^1^H NMR (300 MHz, CDCl_3_) δ 7.86 (br d, 1H, *J* = 10.2 Hz), 7.56 (d, 1H, *J* = 3.6 Hz), 7.46 (dd, 1H, *J* = 8.4 Hz, *J* = 5.4 Hz), 6.98 (td, 1H, *J* = 9.0 Hz, *J* = 2.4 Hz), 6.53 (d, 1H, *J* = 3.6 Hz), 1.67 (s, 9H); ^13^C NMR (125 MHz, CDCl_3_) δ 161.05 (d, ^1^*J*_FC_ = 238.8 Hz), 149.71, 135.55 (d, *J*_FC_ = 12.5 Hz), 126.95, 126.35 (d, *J*_FC_ = 2.5 Hz), 121.62 (d, *J*_FC_ = 10.0 Hz), 111.10 (d, *J*_FC_ = 23.8 Hz), 107.19, 102.74 (d, *J*_FC_ = 28.8 Hz), 84.25, 28.35; HRMS (ESI) [M + H]^+^ Calcd for C_13_H_15_O_2_NF: 236.1081, found: 236.1092.

#### 2-(2-[^18^F]Fluoro-6-(piperidin-1-yl)pyridin-3-yl)-1*H*-indole ([^18^F]**74**)

Et_4_NHCO_3_ (70 mg) was dissolved in Milli-Q H_2_O (10 mL) to give a 7 mg/mL solution. A Waters Sep-Pak Plus Light QMA (WAT023525) was rinsed with Et_4_NHCO_3(aq)_ solution (8 mL, 7 mg/mL), then Milli-Q H_2_O (10 mL) and then air (25 mL). A Waters C_18_ Sep-Pak (WAT020515) was rinsed with EtOH (10 mL) and then Milli-Q H_2_O (10 mL). No-carrier added [^18^F]F^–^ was produced via the ^18^O(p,n)^18^F reaction (Siemens Eclipse HP cyclotron) and transferred in [^18^O]H_2_O to a lead-shielded hot cell (radiolabeling was then performed remotely with the aid of remote manipulators). [^18^F]F^–^ (26.6 GBq (719 mCi)) was trapped on the QMA and eluted with Et_4_NHCO_3(aq)_ solution (1 mL, 7 mg/mL), then CH_3_CN (1 mL) (451.4 MBq (12.2 mCi) remained on the QMA), and collected in a 5 mL V-vial. The solvents were evaporated under argon flow at 110 °C, then the Et_4_N^18^F was azeotropically dried with CH_3_CN aliquots (1 mL × 3) at 110 °C under argon flow. Compound **71** (3.8 mg) was dissolved in DMSO (0.6 mL) and added to the dried Et_4_N^18^F, the V-vial was capped and mixed gently for ∼10 s until the solution turned faint pink, then heated at ∼140 °C for 30 min. The V-vial was cooled for ∼1 min, the solution was diluted with HPLC solvent (0.5 mL, 55:45 v/v CH_3_CN/0.1 M NH_4_HCO_2_ pH 4.2), loaded onto the HPLC loop, the V-vial was rinsed with HPLC solvent (0.5 mL), the solvent rinse was loaded onto the HPLC loop, and the sample was purified by prep-HPLC (Phenomenex Gemini, 250 × 10 mm + guard; 5 mL/min for 6 min, then 10 mL/min). The HPLC radioactive peak at ∼24–25 min was collected, diluted with Milli-Q H_2_O (50 mL), passed through the C_18_ Sep-Pak, and the Sep-Pak was rinsed with Milli-Q H_2_O (10 mL). The radiotracer was eluted from the Sep-Pak with EtOH (1 mL), then 0.9% sterile saline (9 mL), passed through a 0.2 μm filter, and collected in a sterile vial to afford [^18^F]**74** (1.2 GBq (31.9 mCi); 4.4% radiochemical yield (not decay-corrected); 99.4% radiochemical purity (Phenomenex Gemini, 5 μm, NX-C18 110 Å, 100 × 4.6 mm 60:40 v/v CH_3_CN/0.1 M NH_4_HCO_2_ pH 4.2; 1 mL/min; 254 nm); apparent^95,96^*A*_m_ = 129.5 MBq/nmol (3.5 mCi/nmol); pH 5). Radiochemical identity was confirmed by coinjection with **74**. The endotoxin level of the final formulation was <2.00 EU/mL (Charles Rivers Endosafe).

#### 4-(6-[^18^F]Fluoro-5-(1*H*-indol-2-yl)pyridin-2-yl)morpholine ([^18^F]**75**, [^18^F]JSS20–183A)

Et_4_NHCO_3_ (70 mg) was dissolved in Milli-Q H_2_O (10 mL) to give a 7 mg/mL solution. A Waters Sep-Pak Plus Light QMA (WAT023525) was rinsed with Et_4_NHCO_3(aq)_ solution (8 mL, 7 mg/mL), then Milli-Q H_2_O (10 mL), then air (25 mL). A Waters C_18_ Sep-Pak (WAT020515) was rinsed with EtOH (10 mL) and then Milli-Q H_2_O (10 mL). No-carrier added [^18^F]F^–^ was produced via the ^18^O(p,n)^18^F reaction (Siemens Eclipse HP cyclotron) and transferred in [^18^O]H_2_O to a lead-shielded hot cell (radiolabeling was then performed remotely with the aid of remote manipulators). [^18^F]F^–^ (30.3 GBq (818 mCi)) was trapped on the QMA and eluted with Et_4_NHCO_3(aq)_ solution (1 mL, 7 mg/mL), then CH_3_CN (1 mL), then air (1 mL) (296 MBq (8.0 mCi) remained on the QMA), and collected in a 5 mL V-vial. The solvents were evaporated under argon flow at 110 °C, then the Et_4_N^18^F was azeotropically dried with CH_3_CN aliquots (1 mL × 3) at 110 °C under argon flow. Compound **72** (2.5 mg) was dissolved in DMSO (0.5 mL), added to the dried Et_4_N^18^F (the solution turned light purple), the V-vial was capped, and heated at ∼140 °C for 30 min. The V-vial was cooled for ∼1 min, the solution was diluted with HPLC solvent (0.5 mL, 45:55 v/v CH_3_CN/0.1 M NH_4_HCO_2_ pH 4.2), loaded onto the HPLC loop, the V-vial was rinsed with HPLC solvent (0.5 mL), the solvent rinse was loaded onto the HPLC loop, and the sample was purified by prep-HPLC (Phenomenex Gemini, 250 mm × 10 mm + guard; 5 mL/min for 6 min, then 8 mL/min). The HPLC radioactive peak at ca. 20.4–22 min was collected, diluted with Milli-Q H_2_O (50 mL), passed through the C_18_ Sep-Pak, and the Sep-Pak was rinsed with Milli-Q H_2_O (10 mL). The radiotracer was eluted from the Sep-Pak with EtOH (1 mL), then 0.9% sterile saline (9 mL), passed through a 0.2 μm filter, and collected in a sterile vial to afford [^18^F]**75** (3.5 GBq (94.3 mCi)); 11.5% radiochemical yield (not decay-corrected); 98.9% radiochemical purity (Phenomenex Gemini, 5 μm, NX-C18 110 Å, 100 mm × 4.6 mm; 45:55 v/v CH_3_CN/0.1 M NH_4_HCO_2_ pH 4.2; 1 mL/min; 254 nm); apparent^95,96^*A*_m_ = 51.8 MBq/nmol (1.4 mCi/nmol); pH 5. Radiochemical identity was confirmed by co-injection with **75**. The endotoxin level of the final formulation was <2.00 EU/mL (Charles Rivers Endosafe).

#### Log *D*_7.4_ Determination of **[**^**18**^**F]74**

To four 8 mL glass vials with plastic screw caps (pulp/vinyl liner) were added octanol (2 mL) and 20 mM NaH_2_PO_4(aq)_ solution pH 7.4 (2 mL), and the vials were vortexed for 15 s. To each vial was added radiotracer solution (50 μL, ∼170 kBq (∼4.6 μCi)), then each vial was vortexed for 30 s and let stand for 30 min to allow the layers to separate. From each of the four glass vials, samples of the octanol layer (0.5 mL) were removed using a pipettor with a plastic tip and placed in four counting tubes. Glass pipettes were then used to transfer samples of the aqueous layer (∼1.5 mL) from the four glass vials to glass test tubes. A sample from each aqueous layer (0.5 mL) was removed using a pipettor with a plastic tip and placed in a counting tube. The tubes were counted, and the log(octanol counts/aqueous counts) value was calculated for each pair of tubes and then averaged (log *D*_7.4_ = 1.80 ± 0.01 (*n* = 4)).

#### Log *D*_7.4_ Determination of **[**^**18**^**F]75**

To four 8 mL glass vials with plastic screw caps (pulp/vinyl liner) was added octanol (2 mL) and 20 mM NaH_2_PO_4(aq)_ solution pH 7.4 (2 mL), and the vials were vortexed for 15 s. To each vial was added radiotracer solution (20 μL, ∼11.1 kBq (∼0.3 μCi)) that had been diluted with NaH_2_PO_4(aq)_ solution, then each vial was vortexed for 30 s and let stand for 30 min to allow the layers to separate. From each of the four glass vials samples of the octanol layer (0.5 mL) were removed using a pipettor with a plastic tip and placed in four counting tubes. Glass pipettes were then used to transfer samples of the aqueous layer (∼1.5 mL) from the four glass vials to glass test tubes. A sample from each aqueous layer (0.5 mL) was removed using a pipettor with a plastic tip and placed in counting tubes. The tubes were counted, and the log(octanol counts/aqueous counts) value was calculated for each pair of tubes and then averaged (log *D*_7.4_ = 1.96 ± 0.03 (*n* = 4)).

#### PET/CT Imaging of [^18^F]**74** and **[**^**18**^**F]75**

Nonhuman primate (NHP) imaging studies in male macaque (*m. mulatta*) monkeys (12 and 13 kg) were carried out in accordance with the guidelines set forth by the Institutional Animal Care and Use Committee (IACUC) at the University of Pittsburgh under an IACUC-approved protocol. PET/CT imaging was performed with a Siemens Biograph mCT Flow 64–4 R PET/CT scanner (22.1 mm axial FOV, maximum intrinsic spatial resolution of 4.3 mm fwhm). NHP were initially sedated with ketamine (15 mg/kg, i.m.) and glycopyrrolate (0.01 mg/kg) prior to intubation and induction of isoflurane anesthesia (0.5–2.0%). A venous catheter was placed in the saphenous vein for intravenous injection of **[**^**18**^**F]74** or **[**^**18**^**F]75** and venous blood sampling. NHPs were positioned using a scout planar radiograph to place the brain in the center of the field of view, after which a low-dose (19 mrem) helical CT scan was collected for attenuation correction of PET emission data. List-mode acquisition of PET emission data commenced with the start of slow (20 s) bolus injections of **[**^**18**^**F]74** (101.8 MBq, molar activity 110.6 MBq/nmol) or **[**^**18**^**F]75** (96.0 MBq, molar activity 88.4 MBq/nmol) and continued for 90 min. Emission data were binned into a dynamic series of 32 frames of increasing duration (20 s to 10 min) and reconstructed using filtered back projection with Fourier rebinning with standard corrections applied for attenuation, scatter, electronics dead-time, and physical decay.

#### Radiometabolite Analysis of **[**^**18**^**F]74** and **[**^**18**^**F]75**

Venous blood samples (3 mL) were collected at 2, 10, 30, 45, and 90 min after radiotracer administration. Whole blood samples were transferred from the sample syringe into centrifuge tubes and centrifuged (Eppendorf MiniSpin 5452) for 45 s at 11,300*g*. The plasma supernatant (∼0.5 mL) was transferred to a centrifuge tube and diluted with an equal volume of acetonitrile. The resultant solution was vortexed for 1 min and then centrifuged for 45 s at 12,500 rpm. The resulting supernatant was analyzed by reverse-phase HPLC (Phenomenex Gemini, 5 μm, NX-C18 110 Å, 100 mm × 4.6 mm; 45:55 v/v CH_3_CN/0.1 M NH_4_HCO_2_ pH 4.2; 1 mL/min) and an inline 3″ NaI(Tl) scintillation detector (GABI Star, Elysia-Raytest, GmBH) enveloping a large-volume (2.0 mL) flow cell for measuring radioactivity in the eluent. Analysis of radiochromatograms was performed using GINA Star software v. 4.07 (Elysia-Raytest, GmBH) to determine the fraction of unmetabolized **[**^**18**^**F]74** or **[**^**18**^**F]75** at each time point. System suitability of the analytical system was evaluated by injection of the nonradioactive standard using the same analytical system described above using a Waters UV detector (Model 481, 254 nm).

#### Post-Mortem Tissues

Post-mortem human brain tissues were collected by brain banks at the University of Pittsburgh Alzheimer’s Disease Research Center (ADRC), the University of Pittsburgh Brain Tissue Donation Program, the Center for Neurodegenerative Disease Research (CNDR) at the University of Pennsylvania, the Neurodegenerative Disease Brain Bank at the University of California, San Francisco, and Dr. Thomas Beach at Banner/Sun Health AZ (through the Michael J. Fox Foundation) following informed consent of the donors and utilized at the University of Pittsburgh under the approval of the Committee for Oversight of Research and Clinical Training Involving Decedents (CORID no. 295). The binding assays utilized fresh frozen post-mortem human tissue from autopsy-confirmed cases. Brain tissue blocks (1 cm^3^) contained only frequent mixed 3R/4R-tau neurofibrillary tangles and amyloid-β plaque aggregates (AD middle frontal gyrus), or only 4R-tau aggregates (PSP superior frontal gyrus and CBD middle frontal gyrus), or only 3R-tau aggregates (PiD middle frontal gyrus) and no detectable amyloid-β or TDP-43 aggregates. Fresh frozen, autopsy-confirmed PD anterior cingulate cortex tissue blocks (1 cm^3^) contained frequent α-synuclein aggregates and no detectable amyloid-β or TDP-43 aggregates. Fresh frozen brain tissue blocks (1 cm^3^) of midfrontal gyrus from an autopsy-confirmed FTLD-TDP case contained TDP-43 pathology (score 5), but no amyloid-β, tau, or α-synuclein pathology. Fresh frozen brain tissue blocks (1 cm^3^) of cortical gray matter (prefrontal, parietal, occipital, and temporal pole) from a young autopsy-confirmed healthy control contained no neuropathological changes. Fresh frozen brain tissue blocks (1 cm^3^) of midfrontal gyrus from an autopsy-confirmed healthy elderly control contained no amyloid-β, tau, or α-synuclein pathology. The frozen tissue blocks were separately thawed and homogenized in ice-cold pH 7.0 phosphate-buffered saline (PBS) at 300 mg/mL on ice using a glass homogenizer, diluted 30-fold with PBS to 10 mg/mL and homogenized a second time with a Brinkmann Polytron homogenizer before storage at −80 °C. At the time of binding assays, frozen brain tissue homogenates were thawed to room temperature and diluted 10-fold in Tris buffer (pH 7.2) to a concentration of 1 mg/mL.

Fresh frozen whole brains were obtained from three 9-month-old female P301L-JNPL3 mice from the laboratory of Dr. Peter Davies (Feinstein Institute for Medical Research, North Shore-LIJ Health System, Long Island). These mice carry a transgene encoding human tau with four microtubule-binding repeat domains and no N-terminal inserts (4R/0N). The transgene contains the P301L mutation and expression is driven by the mouse prion promoter. Tau inclusions develop in an age- and gene-dose-dependent manner as early as 4.5 months. At 9 months of age, the mice have widespread and abundant tau pathology in the brain, deep cerebellar nuclei, medulla, and spinal cord.^84^ For the assays, brain tissue homogenates were pooled together from all three mice and samples were frozen until processed for the binding assays as described above.

#### In Vitro Competition (*K*_i_) Assays

The unlabeled competitor compound equilibrium inhibition constant (*K*_i_) values were determined versus tritium-labeled radioligands according to previously published procedures^46,65,66^ with minor modifications. A solution of each competitor compound was made in DMSO-*d*_6_ containing dimethyl sulfone (1 mM) as internal standard, and the concentration of the competitor compound was then determined by quantitative NMR.^97^ The solution was then diluted with DMSO to give a stock solution (400 μM) of the competitor compound. Various concentrations of the competitor compound were then made by diluting with Tris buffer. The appropriate concentrations (0.1 nM–1 μM) of unlabeled competitor (400 μL) were combined with solutions of tritium-labeled radioligand in Tris buffer (500 μL, ∼1 nM final concentration of radioligand). Brain tissue homogenate solution (100 μL) in Tris buffer (1 mg/mL) was then added (final concentration = 100 μg brain tissue/mL, 0.25% DMSO). The binding assay solution was incubated at ambient temperature for 60 min, then filtered (Whatman GF/B glass filter) using a Brandel M-24R cell harvester, and rapidly washed with Tris buffer (3 mL × 3). The filter circles were combined with CytoScint-ES, vortexed thoroughly, and counted using a liquid scintillation counter. Complete inhibition (100%) of radioligand-specific binding was defined as the number of counts displaced by a solution (1 μM) of the unlabeled version of the radioligand. All assays were performed in triplicate at each concentration.

#### Binding Affinity (*K*_D_) Assays

Compound [^3^H]**75** homologous^81^ binding affinity (*K*_D_) assays using AD, PSP, CBD, PiD, young CT, elderly CT, PD, TDP-43, and P301L transgenic mouse brain homogenates were performed according to previously published procedures^46,65,66^ with minor modifications. A solution of **75** in DMSO (400 μM) was prepared as described above, diluted with Tris buffer to give a 20-μM solution (5% DMSO/Tris), then diluted serially with 5% DMSO/Tris. The solutions of **75** (50 μL) were each combined with a solution of [^3^H]**75** (50 μL), Tris buffer (800 μL), and brain tissue homogenate solution (100 μL) (final assay solution: 100 μg brain tissue/mL, ∼1 nM [^3^H]**75**, 0.2–1000 nM **75**, 0.25% DMSO/Tris buffer). Incubation, filtration, and counting were performed as described above. The concentration of bound [^3^H]**75** was determined from the radioactivity retained on the filter (corrected for the nondisplaceable radioactivity—defined as that remaining with ∼1 μM **75**) and the molar activity of [^3^H]**75** after dilution with varying concentrations of **75**. A Scatchard plot of the bound/free versus bound radioligand values at the different ligand concentrations was used to determine the *K*_D_ value (slope = −1/ *K*_D_). The Bmax value was determined by the *x*-axis intercept of the bound/free versus bound line. All assays were performed at least in triplicate.

### In Vitro Real-Time Autoradiography and Immunohistochemistry (IHC)

Formalin-fixed paraffin-embedded human brain tissues from AD, PSP, CBD, and elderly CT cases were acquired from the Center for Neurodegenerative Disease Research (CNDR) at the University of Pennsylvania. Clinical demographic data is shown in Table S9 (Supporting Information).

In vitro autoradiography was performed using 6-μm-thick deparaffinized sections derived from AD, PSP, CBD, and elderly CT brains. Brain sections underwent deparaffinization and antigen retrieval step using Citrate buffer (0.5 M, pH 6.0) for 1 h at 70 °C in a preheated water bath. All slides were subsequently equilibrated for 30 min in 1× PBS (Dulbecco’s phosphate-buffered saline) and then incubated for 90 min at ambient temperature with 4.5 nM [^3^H]**75**. The sections were rinsed two times in cold buffer 1× PBS + 20% EtOH for 5 min, followed by a quick dip in cold distilled water. Nonspecific binding was determined using 1 μM of unlabeled **75**. Slides were then allowed to air-dry before being exposed and scanned in a real-time autoradiography system (BeaQuant instrument, ai4R) for 24 h. ROI delineation and quantification of signal were performed by using the image analysis software Beamage (ai4R). Specific binding was determined by subtracting the nonspecific signal (NSB) from the total signal and expressed as counts/min/mm^2^.

Immunohistochemistry was performed on deparaffinized sections adjacent to those used for autoradiography. Sections were permeabilized with 0.1% Triton X-100 for 10 min followed by 3 × 5 min washes in PBS Tween 20 (PBST) buffer. Sections underwent hydrogen peroxide blocking for 15 min and PBS + 10% goat serum +1% BSA + 0.1% Tween 20 blocking for 1 h at ambient temperature. Sections were immunostained using Phospho-Tau (Ser202, Thr205) Monoclonal Antibody (AT8) and Antibeta Amyloid antibody (mOC23) (Table S10, Supporting Information) used at 1:500 dilution overnight at 4 °C. After a series of thorough washes with PBST buffer, the slides were incubated with the secondary antibody Goat Anti-Mouse IgG H&L (HRP) and Goat antirabbit IgG H&L (HRP) (Table S11, Supporting Information) at 1:10,000 for 1 h at ambient temperature. The sections were washed again with PBST buffer, 3 × 5 min, treated with DAB substrate for 10 min, and counterstained with Meyer’s hematoxylin dye. Subsequently, the sections were dehydrated sequentially into 50, 75, 95, and 100% ethanol and xylene baths for 5 min each, then mounted with Limonene mounting media and coverslipped for microscopy. Images were captured with a Leica Aperio slide scanner (RRID: SCR_022420) at 40× magnification.

## Supporting Information Available

Results of the compound **75** (JSS20–183A) Eurofins CNS SafetyScreen assays (XLSX)
SMILES strings for all target compounds tested (CSV)
Additional synthetic schemes, additional binding data tables, analytical HPLC purity of tested compounds, radiolabeling HPLC chromatograms, CNS prediction model data, autoradiography tissue demographics, X-ray crystallography data, and synthesized compound spectral data (PDF)

## Abbreviations Used

AD: Alzheimer’s disease
CBD: corticobasal degeneration
CNS: central nervous system
CT: control
CTE: chronic traumatic encephalopathy
FFPE: formalin-fixed, paraffin-embedded
IHC: immunohistochemistry
PD: Parkinson’s disease
PET: positron emission tomography
PiB: Pittsburgh Compound-B
PiD: Pick’s disease
PSP: progressive supranuclear palsy
SAR: structure–activity relationship
SUV: standardized uptake value
TDP-43: transactive response DNA-binding protein 43
