# Supplementary material for: Structure–Activity Relationships and Evaluation of 2-(Heteroaryl-cycloalkyl)-1H-indoles as Tauopathy Positron Emission Tomography Radiotracers
Source: J Med Chem. 2025 Mar 11;68(6):6462–92. doi: 10.1021/acs.jmedchem.4c02988 (PMC11956013; doi:10.1021/acs.jmedchem.4c02988)
Supplement: Supplementary file 3 — jm4c02988_si_003.pdf [file jm4c02988_si_003.pdf]

Supporting Information

Structure-Activity Relationships and Evaluation of 2-(Heteroaryl-Cycloalkyl)-1*H*-Indoles as Tauopathy Positron Emission Tomography Radiotracers

Jeffrey S. Stehouwer,*[,1] Guofeng Huang,[1] Dinahlee Saturnino Guarino,[2] Manik L. Debnath,[3] Ashok Polu,[1] Steven J. Geib,[4] Brian Lopresti,[1] Milos D. Ikonomovic,[3,5] Neale Mason,[1] Robert H. Mach,[2] Chester A. Mathis[1]

* jeff.stehouwer@pitt.edu

1) Department of Radiology, University of Pittsburgh, Pittsburgh, Pennsylvania 15213, United States.
2) Department of Radiology, University of Pennsylvania, Philadelphia, Pennsylvania 19104-6323, United States.
3) Department of Psychiatry, University of Pittsburgh, Pittsburgh, Pennsylvania 15213, United States.
4) X-ray Crystallography Laboratory, Department of Chemistry, University of Pittsburgh, Pittsburgh, Pennsylvania 15213, United States.
5) Geriatric Research and Clinical Education, VA Pittsburgh Healthcare System, Pittsburgh, Pennsylvania 15240, United States.

Table of Contents

Scheme S1. Synthesis of compounds **29** – **36** Page S3
Figure S1. X-ray crystal structure of **44**. Page S3
Scheme S2. Synthesis of compounds **58** – **61**, and **63** Page S3
Scheme S3. Synthesis of compounds **64** – **68** Page S4
Table S1. Inhibition Constant (K_i) Values of Candidate Ligands versus [^{3}H]**7**, [^{3}H]**8**, and [^{3}H]**3** in AD, PSP, and CBD Brain Tissue Homogenates. Pages S5
Table S2. Comparison of Inhibition Constant (K_i) Values of Pyrimidine-4-Hydroxypiperidine Compounds in AD, PSP, and CBD Brain Tissue Homogenates. Page S6
Table S3. Comparison of Inhibition Constant (K_i) Values of Compounds Where Substituents on the Piperidine Ring Are Varied in AD, PSP, and CBD Brain Tissue Homogenates. Pages S6
Table S4. Comparison of Inhibition Constant (K_i) Values of Piperidine-Substituted Compounds in AD, PSP, and CBD Brain Tissue Homogenates. Page S7
Table S5. Comparison of Inhibition Constant (K_i) Values of Morpholine-Substituted Compounds in AD, PSP, and CBD Brain Tissue Homogenates. Page S8
Table S6. Comparison of Inhibition Constant (K_i) Values of Pyrrolidine-Substituted Compounds in AD, PSP, and CBD Brain Tissue Homogenates. Page S9
Table S7. Inhibition Constant (K_i) Values of Selected Ligands versus [^{3}H]**5** in AD, PSP, and CBD Brain Tissue Homogenates, and versus [^{3}H]PiB in AD Brain Tissue Homogenates. Page S9
Table S8. Analytical HPLC data of compounds screened in the *in vitro* binding assays, ordered by *k*' values for each solvent ratio. Page S10
Figure S2. Analytical HPLC chromatograms of compounds **109**, **22**, **45**, **23**, **21**, **74**, **81**, **89**, **98**, and **96**. Page S11
Figure S3. Analytical HPLC chromatograms of compounds **46**, (*R*)-**43**, **75**, **42**, **45**, **21**, **96**, **105**, and **90**. Page S12
Figure S4. Analytical HPLC chromatograms of compounds **106**, **103**, **104**, **94**, **88**, **87**, **78**, and **79**. Page S13

Figure S5. Analytical HPLC chromatogram of compound **44**. Page S14
Figure S6. Analytical HPLC chromatograms of compounds **24**, **28**, and **27**. Page S14
Figure S7. Analytical HPLC chromatograms of compounds **52**, **53**, **108**, and **49**. Page S15
Figure S8. MPO and BBB Score results for **74**. Page S16
Figure S9. MPO and BBB Score results for **75**. Page S16
Figure S10. SwissADME BOILED-Egg plot of **74** and **75**. Page S17
Figure S11. HPLC chromatogram of crude [^{18}F]**74** reaction mixture. Page S17
Figures S12 – S14. HPLC chromatograms of crude [^{18}F]**75** reaction mixture. Pages S18 – S19
Figure S15. Semi-preparatory HPLC chromatogram of the purification of [^{18}F]**74**. Page S19
Figure S16. Semi-preparatory HPLC chromatogram of the purification of [^{18}F]**75**. Page S20
Figure S17. Analytical HPLC chromatograms of the [^{18}F]**74** final product co-injected with **74**. Page S20
Figure S18. Analytical HPLC chromatograms of the [^{18}F]**75** final product co-injected with **75**. Page S21
Figure S19. Unmetabolized venous fraction of [^{18}F]**74** and [^{18}F]**75** in male rhesus macaques. Page S21
Figure S20. Results of Eurofins IC_{50} determination of **75** (JSS20-183A) at human MAO-B. Page S22
Table S9. Selected demographics of cases used for autoradiography and immunohistochemistry. Page S23
Table S10. Primary antibody selected for immunohistochemistry. Page S23
Table S11. Secondary antibody selected for immunohistochemistry. Page S23
Table S12. Sample and crystal data for **44**. Page S24
Table S13. Sample and crystal data for **71**. Page S24
Table S14. Sample and crystal data for **72**. Page S25
Table S15. Sample and crystal data for **75**. Page S25
Figures S21 – S118. NMR spectra and high-resolution mass spectra of synthesized compounds. Pages S26 – S123

Scheme S1. Synthesis of compounds 29 – 36

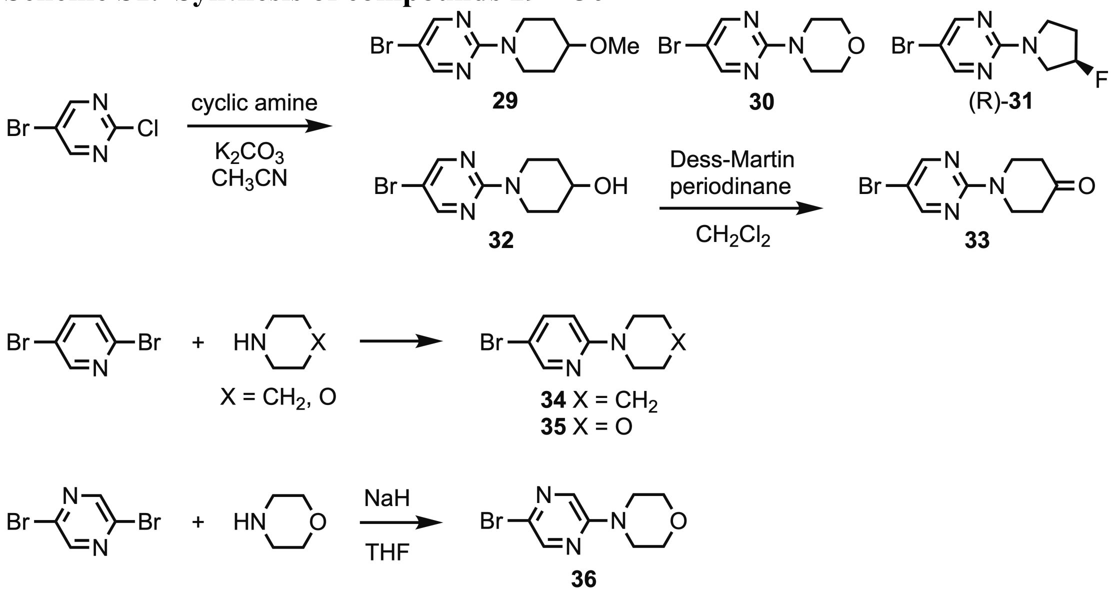

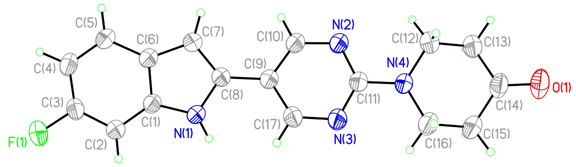

Figure S1. X-ray crystal structure of **44** (CCDC Deposition Number 2403956).

Scheme S2. Synthesis of compounds 58 – 61, and 63

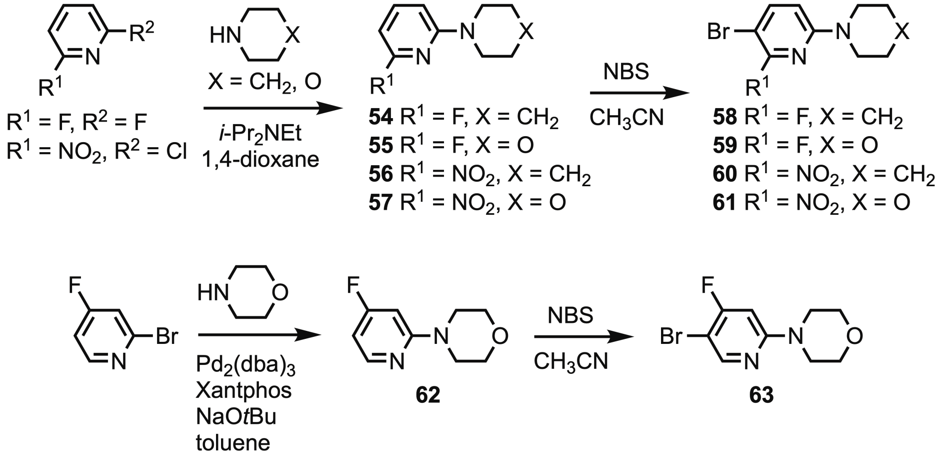

Scheme S3. Synthesis of compounds 64 – 68

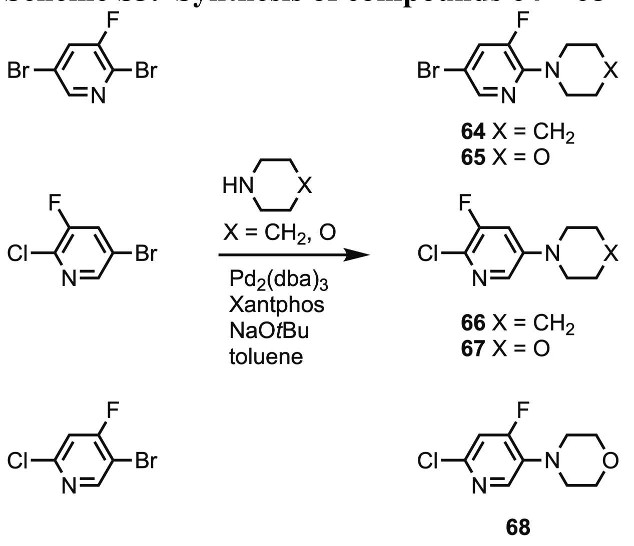

Table S1. Inhibition Constant (K_i) Values of Candidate Ligands versus [^{3}H]**7**, [^{3}H]**8**, and [^{3}H]**3** in AD, PSP, and CBD Brain Tissue Homogenates.[a]

	[^{3}H]**7**			[^{3}H]**8**			[^{3}H]**3**		
	K_i (nM)			K_i (nM)			K_i (nM)		
Cmpd	AD tissue	PSP tissue	CBD tissue	AD tissue	PSP tissue	CBD tissue	AD tissue	PSP tissue	CBD tissue
**7**	12±7.3[b]	24	22±12[b]	6.7	15	14	18[e]	20[e]	22[e]
**8**	8.4	13	11	7.7±0.6[c]	8.5±1.2[d]	11±1.3[d]	22[e]	18[e]	19[e]
**9**	11	8.9	8.9	10	11	15	33	37	40
**21**	3.5	6.2	6.3	7.7	9.2	12	27	26	28
**22**	11	14	11	14	18	15	35	37	47
**23**	37	23	n.d.	n.d.	n.d.	n.d.	n.d.	n.d.	n.d.
**24**	120	129	n.d.	74	76	n.d.	n.d.	n.d.	n.d.
**27**	9.4	8.9	9.0	11	13	10	26	33	38
**28**	39	32	n.d.	34	31	n.d.	n.d.	n.d.	n.d.
**42**	5.0	10	8.2	9.5	10	14	24	19	22
(*R*)-**43**	16	14	15	17	23	20	48	41	46
**44**	12	13	17	17	14	15	35	31	36
**45**	4.6	4.6	6.2	4.9	5.2	5.9	25	28	27
**46**	9.5	7.9	11	13	11	12	25	24	26
**49**	45	91	n.d.	n.d.	n.d.	n.d.	n.d.	n.d.	n.d.
**52**	11	24	n.d.	n.d.	n.d.	n.d.	n.d.	n.d.	n.d.
**53**	21	20	n.d.	n.d.	n.d.	n.d.	n.d.	n.d.	n.d.
**74**	8.0	6.6	6.4	7.8	7.7	7.8	29	30	33
**75**	9.2	10	13	6.5	10	11	22	28	30
**78**	n.d.	n.d.	n.d.	9.2	9.5	10	n.d.	n.d.	n.d.
**79**	n.d.	n.d.	n.d.	5.0	6.3	7.7	n.d.	n.d.	n.d.
**81**	n.d.	n.d.	n.d.	29	34	26	n.d.	n.d.	n.d.
**87**	n.d.	n.d.	n.d.	8.0	7.3	8.2	n.d.	n.d.	n.d.
**88**	n.d.	n.d.	n.d.	11	12	11	n.d.	n.d.	n.d.
**89**	n.d.	n.d.	n.d.	7.5	7.8	8.7	n.d.	n.d.	n.d.
**90**	n.d.	n.d.	n.d.	24	20	18	n.d.	n.d.	n.d.
**94**	n.d.	n.d.	n.d.	13	15	12	n.d.	n.d.	n.d.
**95**	n.d.	n.d.	n.d.	16	17	14	n.d.	n.d.	n.d.
**96**	n.d.	n.d.	n.d.	7.9	8.6	9.5	n.d.	n.d.	n.d.
**98**	n.d.	n.d.	n.d.	15	20	16	n.d.	n.d.	n.d.
**103**	n.d.	n.d.	n.d.	21	25	22	n.d.	n.d.	n.d.
**104**	n.d.	n.d.	n.d.	17	20	20	n.d.	n.d.	n.d.
**105**	n.d.	n.d.	n.d.	50	44	49	n.d.	n.d.	n.d.
**106**	n.d.	n.d.	n.d.	115	65	56	n.d.	n.d.	n.d.
**108**	19	20	39	20	23	30	n.d.	n.d.	n.d.
**109**	22	32	n.d.	n.d.	n.d.	n.d.	n.d.	n.d.	n.d.

[a)] $n = 1$, except where noted. n.d. = not determined. [b)] $n = 2$. [c)] $n = 5$. [d)] $n = 4$. [e)] Data from reference 46.

Table S2. Comparison of Inhibition Constant (K_i) Values of Pyrimidine-4-Hydroxypiperidine Compounds in AD, PSP, and CBD Brain Tissue Homogenates.[a]

	[^{3}H]**7**			[^{3}H]**8**		
		K_i (nM)			K_i (nM)	
Compound	AD tissue	PSP tissue	CBD tissue	AD tissue	PSP tissue	CBD tissue
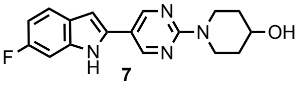 **7**	12±7.3 (n = 2)	24	22±12 (n = 2)	6.7	15	14
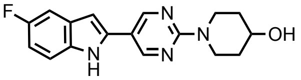 **53**	21	20	n.d.	n.d.	n.d.	n.d.
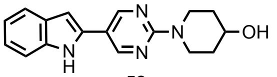 **52**	11	24	n.d.	n.d.	n.d.	n.d.
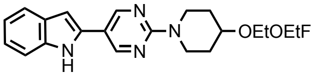 **108**	19	20	39	20	23	30
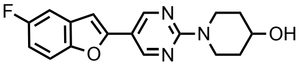 **49**	45	91	n.d.	n.d.	n.d.	n.d.

[a] n = 1, except where noted. n.d. = not determined.

Table S3. Comparison of Inhibition Constant (K_i) Values of Compounds Where Substituents on the Piperidine Ring Are Varied in AD, PSP, and CBD Brain Tissue Homogenates.[a]

	[^{3}H]**7**			[^{3}H]**8**			[^{3}H]**3**		
		K_i (nM)			K_i (nM)			K_i (nM)	
Compound	AD tissue	PSP tissue	CBD tissue	AD tissue	PSP tissue	CBD tissue	AD tissue	PSP tissue	CBD tissue
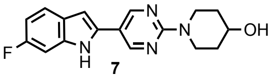 **7**	12±7.3 (n = 2)	24	22±12 (n = 2)	6.7	15	14	18	20	22
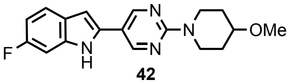 **42**	5.0	10	8.2	9.5	10	14	24	19	22
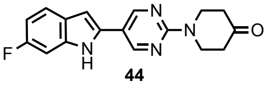 **44**	12	13	17	17	14	15	35	31	36
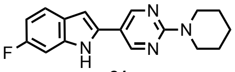 **21**	3.5	6.2	6.3	7.7	9.2	12	27	26	28
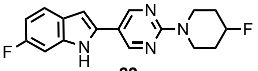 **22**	11	14	11	14	18	15	35	37	47
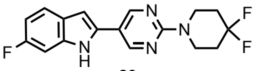 **23**	37	23	n.d.	n.d.	n.d.	n.d.	n.d.	n.d.	n.d.

[a] n = 1, except where noted. n.d. = not determined.

Table S4. Comparison of Inhibition Constant (K_i) Values of Piperidine-Substituted Compounds in AD, PSP, and CBD Brain Tissue Homogenates.[a]

Compound	[^{3}H]**7** K_i (nM) AD tissue	[^{3}H]**7** K_i (nM) PSP tissue	[^{3}H]**7** K_i (nM) CBD tissue	[^{3}H]**8** K_i (nM) AD tissue	[^{3}H]**8** K_i (nM) PSP tissue	[^{3}H]**8** K_i (nM) CBD tissue	[^{3}H]**3** K_i (nM) AD tissue	[^{3}H]**3** K_i (nM) PSP tissue	[^{3}H]**3** K_i (nM) CBD tissue
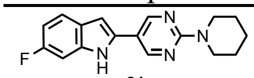 **21**	3.5	6.2	6.3	7.7	9.2	12	27	26	28
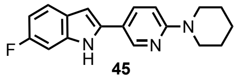 **45**	4.6	4.6	6.2	4.9	5.2	5.9	25	28	27
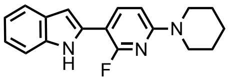 **74**	8.0	6.6	6.4	7.8	7.7	7.8	29	30	33
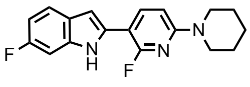 **81**	n.d.	n.d.	n.d.	29	34	26	n.d.	n.d.	n.d.
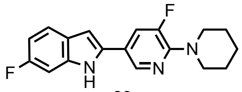 **89**	n.d.	n.d.	n.d.	7.5	7.8	8.7	n.d.	n.d.	n.d.
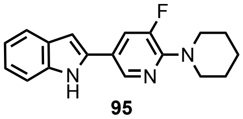 **95**	n.d.	n.d.	n.d.	16	17	14	n.d.	n.d.	n.d.
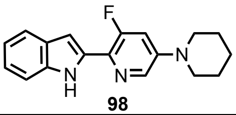 **98**	n.d.	n.d.	n.d.	15	20	16	n.d.	n.d.	n.d.

[a] $n = 1$. n.d. = not determined.

Table S5. Comparison of Inhibition Constant (K_i) Values of Morpholine-Substituted Compounds in AD, PSP, and CBD Brain Tissue Homogenates.[a]

Compound	[^{3}H]**7**			[^{3}H]**8**			[^{3}H]**3**		
	K_i (nM)			K_i (nM)			K_i (nM)		
	AD tissue	PSP tissue	CBD tissue	AD tissue	PSP tissue	CBD tissue	AD tissue	PSP tissue	CBD tissue
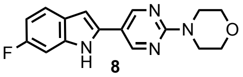 **8**	8.4	13	11	7.7±0.6 (n = 5)	8.5±1.2 (n = 4)	11±1.3 (n = 4)	22	18	19
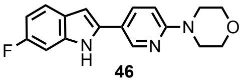 **46**	9.5	7.9	11	13	11	12	25	24	26
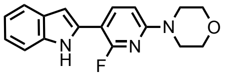 **75**	9.2	10	13	6.5	10	11	22	28	30
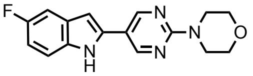 **78**	n.d.	n.d.	n.d.	9.2	9.5	10	n.d.	n.d.	n.d.
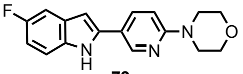 **79**	n.d.	n.d.	n.d.	5.0	6.3	7.7	n.d.	n.d.	n.d.
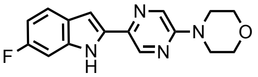 **87**	n.d.	n.d.	n.d.	8.0	7.3	8.2	n.d.	n.d.	n.d.
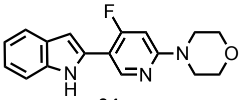 **94**	n.d.	n.d.	n.d.	13	15	12	n.d.	n.d.	n.d.
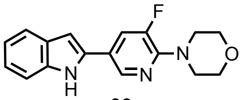 **96**	n.d.	n.d.	n.d.	7.9	8.6	9.5	n.d.	n.d.	n.d.
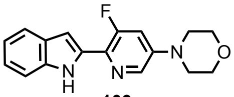 **103**	n.d.	n.d.	n.d.	21	25	22	n.d.	n.d.	n.d.
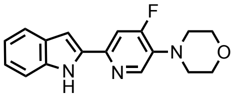 **105**	n.d.	n.d.	n.d.	50	44	49	n.d.	n.d.	n.d.
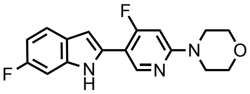 **88**	n.d.	n.d.	n.d.	11	12	11	n.d.	n.d.	n.d.
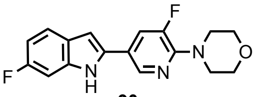 **90**	n.d.	n.d.	n.d.	24	20	18	n.d.	n.d.	n.d.
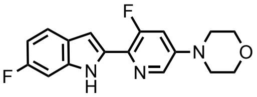 **104**	n.d.	n.d.	n.d.	17	20	20	n.d.	n.d.	n.d.
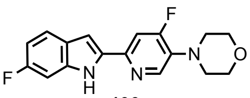 **106**	n.d.	n.d.	n.d.	115	65	56	n.d.	n.d.	n.d.
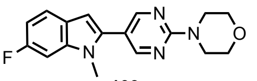 **109**	22	32	n.d.	n.d.	n.d.	n.d.	n.d.	n.d.	n.d.

[a] n = 1, except where noted. n.d. = not determined.

Table S6. Comparison of Inhibition Constant (K_i) Values of Pyrrolidine-Substituted Compounds in AD, PSP, and CBD Brain Tissue Homogenates.[a]

Compound	[^{3}H]**7**			[^{3}H]**8**			[^{3}H]**3**		
		K_i (nM)			K_i (nM)			K_i (nM)	
	AD tissue	PSP tissue	CBD tissue	AD tissue	PSP tissue	CBD tissue	AD tissue	PSP tissue	CBD tissue
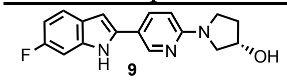 **9**	11	8.9	8.9	10	11	15	33	37	40
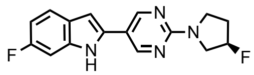 (*R*)-**43**	16	14	15	17	23	20	48	41	46
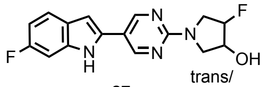 **27** trans/ racemic	9.4	8.9	9.0	11	13	10	26	33	38
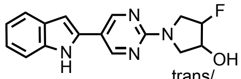 **28** trans/ racemic	39	32	n.d.	34	31	n.d.	n.d.	n.d.	n.d.
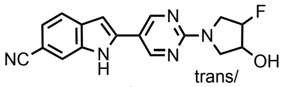 **24** trans/ racemic	120	129	n.d.	74	76	n.d.	n.d.	n.d.	n.d.

[a]) $n = 1$. n.d. = not determined.

Table S7. Inhibition Constant (K_i) Values of Selected Ligands versus [^{3}H]**5** in AD, PSP, and CBD Brain Tissue Homogenates, and versus [^{3}H]PiB in AD Brain Tissue Homogenates.[a]

	[^{3}H]**5**			[^{3}H]PiB
		K_i (nM)		K_i (nM)
Compound	AD tissue	PSP tissue	CBD tissue	AD tissue
**21**	182	185	189	49
**27**	214	219	273	n.d.
**45**	188	218	221	70
**46**	185	227	229	53
**74**	221	203	222	n.d.
**75**	203	335	360	61 ± 3 [b]

[a]) $n = 1$, except where noted. [b]) $n = 3$. n.d. = not determined.

Table S8. Analytical HPLC data of compounds screened in the *in vitro* binding assays, ordered by *k*' values for each solvent ratio.[a]

Compound	Purity (%)	solvent ratio	t_R (min)	*k*'
**109**	99.23	55:45	6.403	5.97
**22**	99.59	55:45	8.411	8.15
**45**	98.58	55:45	10.673	10.61
**23**	99.79	55:45	11.218	11.21
**21**	99.76	55:45	12.547	12.65
**96**	97.05	55:45	18.157	18.65
**74**	99.42	55:45	19.562	20.29
**98**	97.19	55:45	19.923	20.56
**89**	99.90	55:45	21.576	22.35
**81**	99.29	55:45	23.439	24.50
**79**	99.67	45:55	7.386	7.12
**46**	97.67	45:55	7.537	7.20
**78**	97.28	45:55	7.511	7.25
(*R*)-**43**	98.74	45:55	7.660	7.34
**8**	98.38	45:55	8.296	8.03
**87**	98.34	45:55	8.457	8.29
**94**	98.82	45:55	9.834	9.81
**105**	99.38	45:55	10.006	10.00
**96**	99.56	45:55	11.047	11.14
**103**	97.54	45:55	12.172	12.38
**88**	99.10	45:55	12.224	12.43
**106**	99.88	45:55	12.489	12.72
**75**	99.78	45:55	12.682	12.80
**42**	97.82	45:55	13.011	13.16
**90**	98.74	45:55	13.522	13.86
**104**	96.46	45:55	15.361	15.88
**45**	99.35	45:55	22.511	23.50
**21**	99.75	45:55	30.419	32.10
**44**	99.88	40:60	11.356	11.36
**46**	97.48	40:60	12.943	13.08
**24**	95.59	35:65	6.549	6.13
**28**	98.70	35:65	7.188	6.82
**27**	98.49	35:65	9.904	9.78
**52**	99.82	35:65	8.170	7.89
**53**	99.97	35:65	10.369	10.28
**108**	99.79	35:65	19.259	19.96
**49**	96.55	35:65	20.565	21.38
**8**	99.46	35:65	26.067	27.36

[a)] Phenomenex Gemini, 5 µm, NX-C18 110Å, 100 x 4.6 mm; (solvent ratio) v/v CH_3CN/0.1 M NH_4HCO_2 pH 4.2; 1 mL/min; 254 nm. *k*' = (retention time - void time)/void time. Void time was measured by injection of a sample of thiourea.

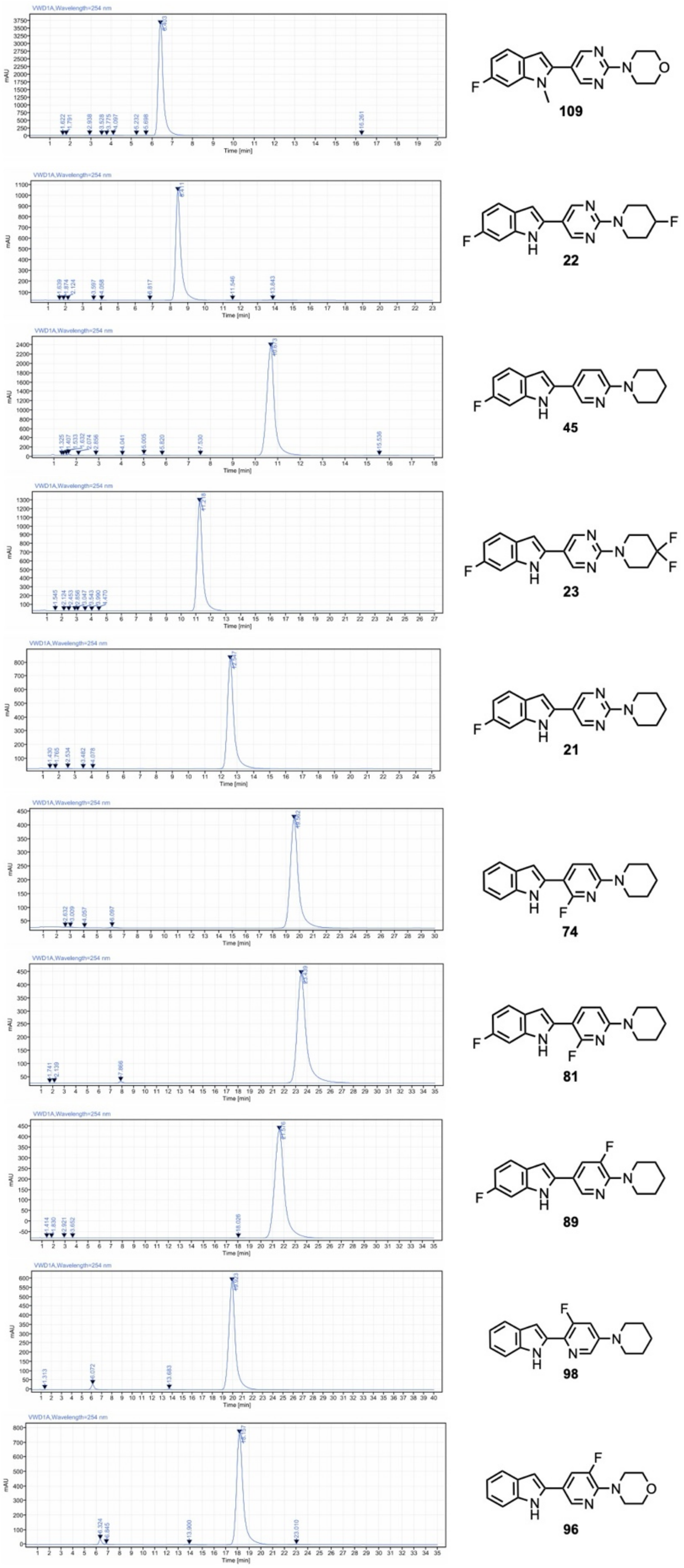

Figure S2. Analytical HPLC chromatograms of compounds **109**, **22**, **45**, **23**, **21**, **74**, **81**, **89**, **98**, and **96** (Phenomenex Gemini, 5 μm, NX-C18 110Å, 100 x 4.6 mm; 55:45 v/v CH_3CN/0.1 M NH_4HCO_2 pH 4.2; 1 mL/min; 254 nm).

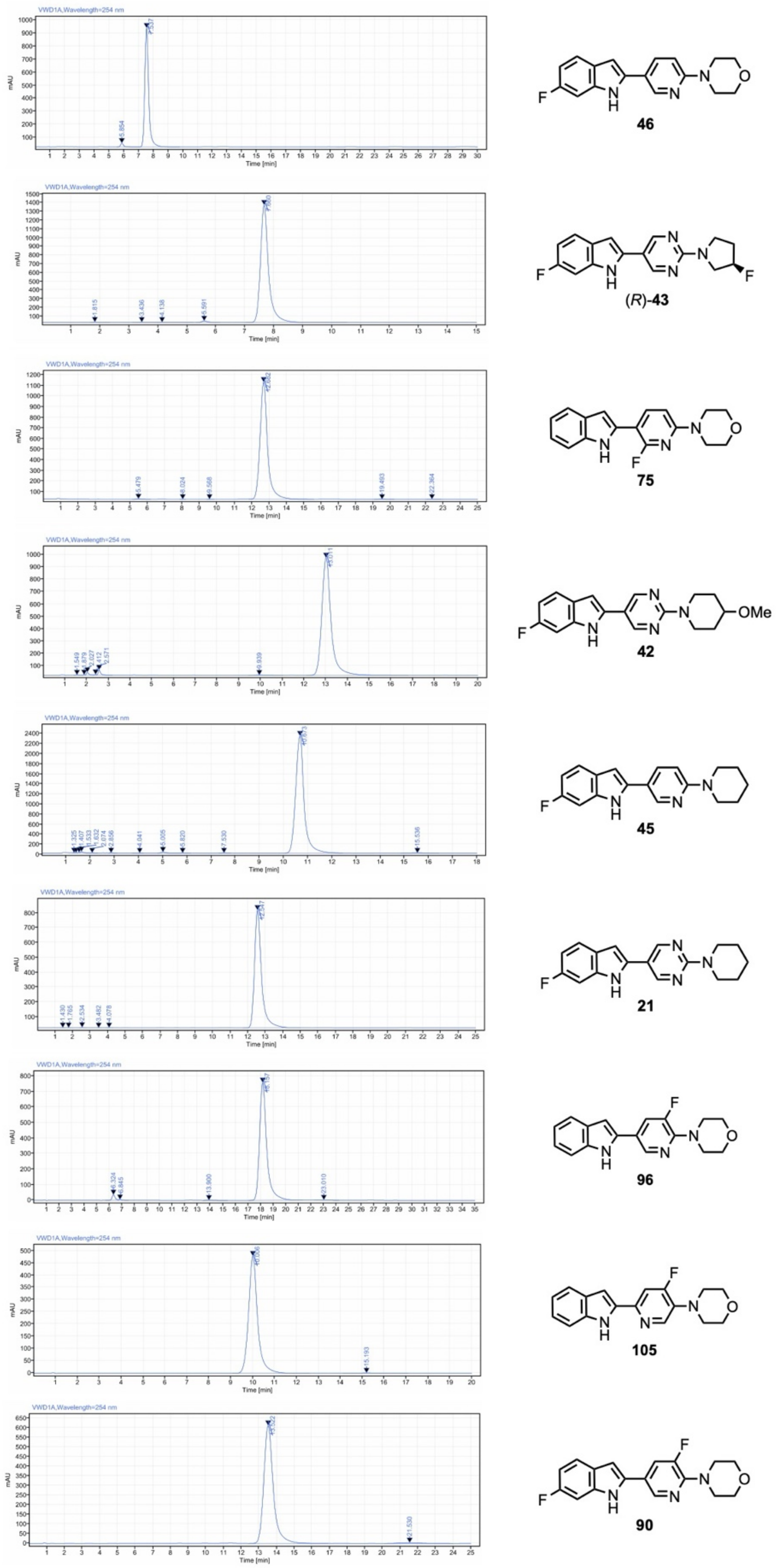

Figure S3. Analytical HPLC chromatograms of compounds **46**, (*R*)-**43**, **75**, **42**, **45**, **21**, **96**, **105**, and **90** (Phenomenex Gemini, 5 μm, NX-C18 110Å, 100 x 4.6 mm; 45:55 v/v CH_3CN/0.1 M NH_4HCO_2 pH 4.2; 1 mL/min; 254 nm).

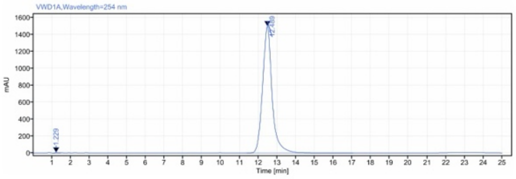

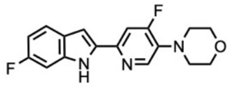

106

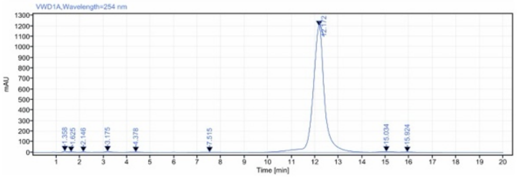

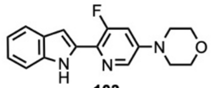

103

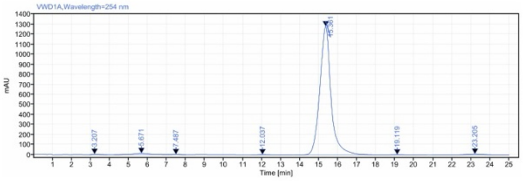

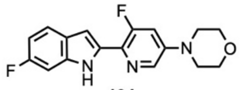

104

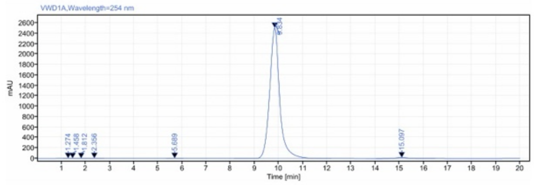

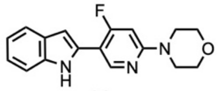

94

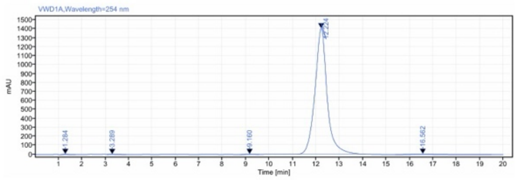

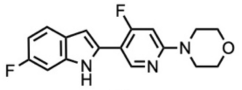

88

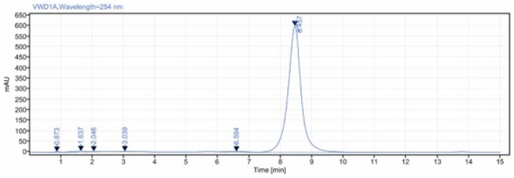

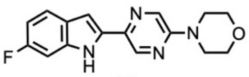

87

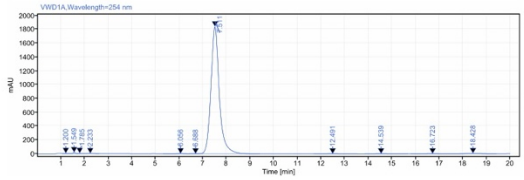

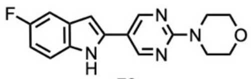

78

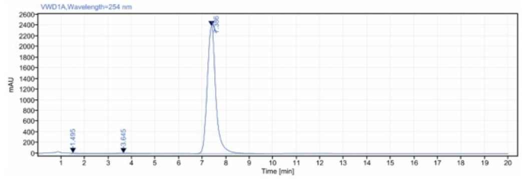

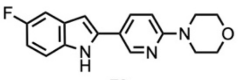

79

Figure S4. Analytical HPLC chromatograms of compounds **106**, **103**, **104**, **94**, **88**, **87**, **78**, and **79** (Phenomenex Gemini, 5 μm, NX-C18 110Å, 100 x 4.6 mm; 45:55 v/v CH_3CN/0.1 M NH_4HCO_2 pH 4.2; 1 mL/min; 254 nm).

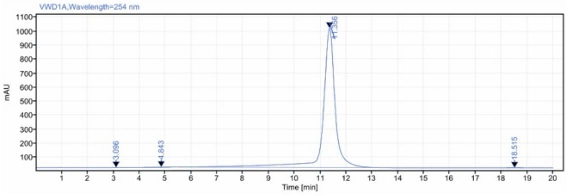

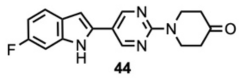

44

Figure S5. Analytical HPLC chromatogram of compound **44** (Phenomenex Gemini, 5 μm, NX-C18 110Å, 100 x 4.6 mm; 40:60 v/v CH_3CN/0.1 M NH_4HCO_2 pH 4.2; 1 mL/min; 254 nm).

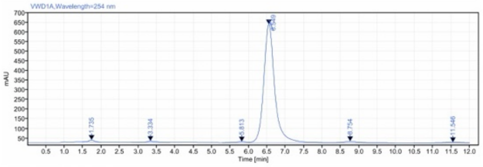

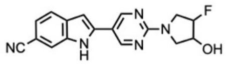

24

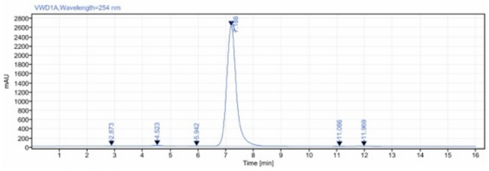

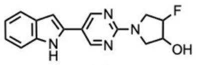

28

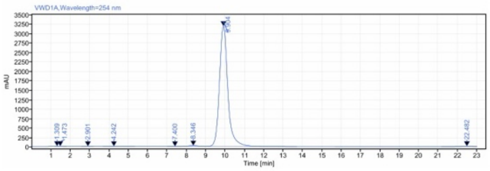

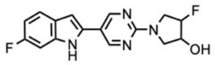

27

Figure S6. Analytical HPLC chromatograms of compounds **24**, **28**, and **27** (Phenomenex Gemini, 5 μm, NX-C18 110Å, 100 x 4.6 mm; 35:65 v/v CH_3CN/0.1 M NH_4HCO_2 pH 4.2; 1 mL/min; 254 nm).

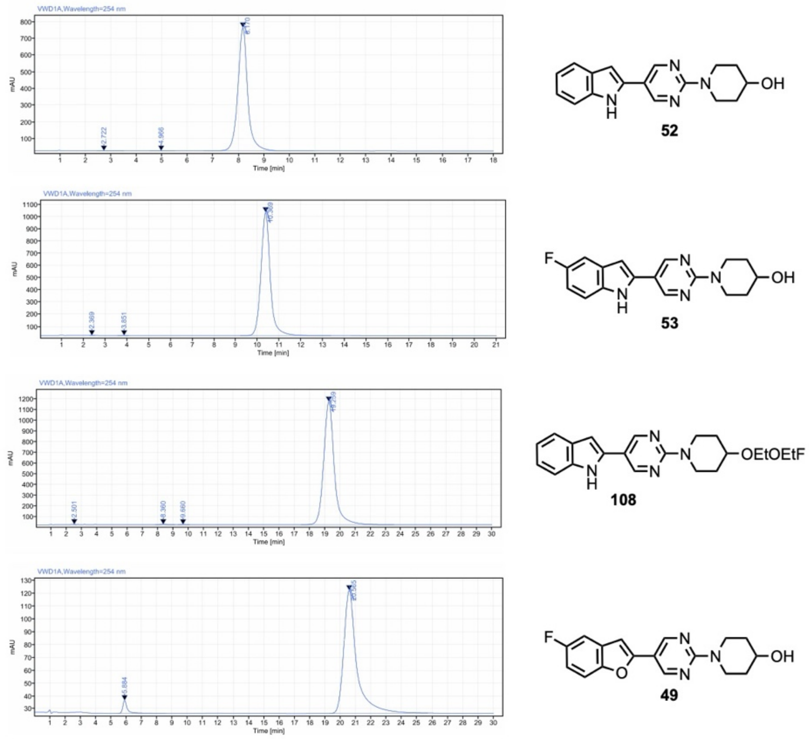

Figure S7. Analytical HPLC chromatograms of compounds **52**, **53**, **108**, and **49** (Phenomenex Gemini, 5 µm, NX-C18 110Å, 100 x 4.6 mm; 35:65 v/v CH_3CN/0.1 M NH_4HCO_2 pH 4.2; 1 mL/min; 254 nm).

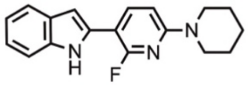

74

CNS MPO and PET MPO			
Property	Value	CNS MPO	CNS PET MPO
ClogP	3.86	0.570	0.117
ClogD	4.51	0.000	0.000
TPSA	31.92	0.596	0.000
MW	295.36	1.000	1.000
HBD	1	0.833	1.000
pKa	14.49	0.000	0.000
	Total Score	3.0	2.1

BBB Score Calculator		
Property	Value	T_0
Number of Aromatic Rings (Aro_R)	2	1.00
Number of Heavy Atoms (HA)	22	0.98
Molecular Weight (MW)	295.36	
Number of Hydrogen Bond Acceptor (HBA)	3	
Number of Hydrogen Bond Donor (HBD)	1	
MWHBN [MWHBN = (MW^(-0.5)*HBN), where HBN=HBA+HBD]	0.23	0.98
Topological Polar Surface Area(TPSA)	31.92	0.78
pKa	14.49	0.00
BBB SCORE		5.01

Figure S8. MPO and BBB Score results for **74** (software used to calculate properties: SwissADME – ClogP (consensus Log $P_{o/w}$ value used), TPSA; ChemAxon – ClogD; Marvin – pK_a).

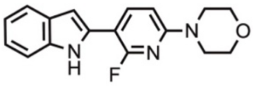

75

CNS MPO and PET MPO			
Property	Value	CNS MPO	CNS PET MPO
ClogP	3.07	0.965	0.775
ClogD	3.44	0.280	0.000
TPSA	41.15	1.000	0.708
MW	297.3334	1.000	1.000
HBD	1	0.833	1.000
pKa	14.49	0.000	0.000
	Total Score	4.1	3.5

Property	Value	T_0
Number of Aromatic Rings (Aro_R)	2	1.00
Number of Heavy Atoms (HA)	22	0.98
Molecular Weight (MW)	297.33	
Number of Hydrogen Bond Acceptor (HBA)	4	
Number of Hydrogen Bond Donor (HBD)	1	
MWHBN [MWHBN = (MW^(-0.5)*HBN), where HBN=HBA+HBD]	0.29	0.87
Topological Polar Surface Area(TPSA)	41.15	0.71
pKa	14.49	0.00
BBB SCORE		4.71

Figure S9. MPO and BBB Score results for **75** (software used to calculate properties: SwissADME – ClogP (consensus Log $P_{o/w}$ value used), TPSA; ChemAxon – ClogD; Marvin – pK_a).

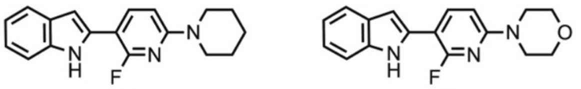

74 **75**

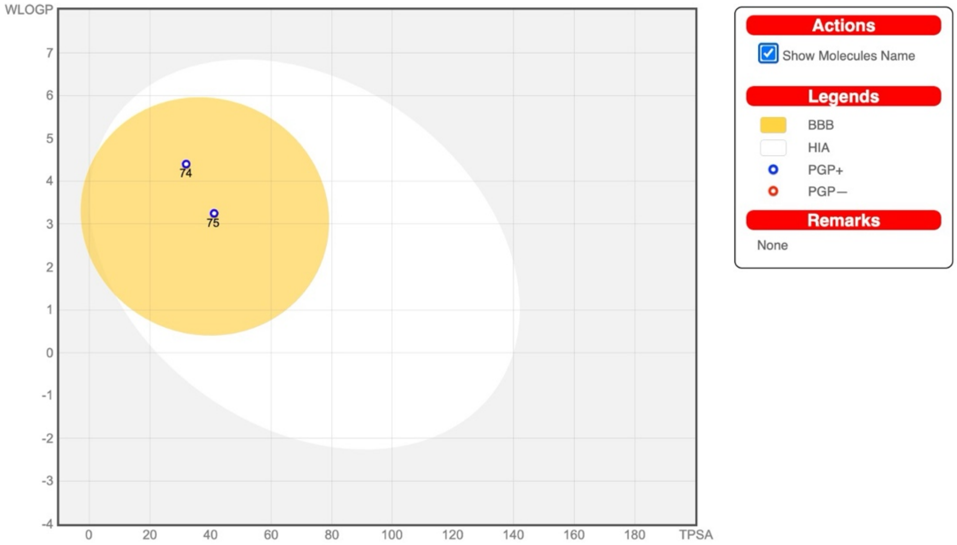

Figure S10. SwissADME BOILED-Egg plot of **74** and **75**.

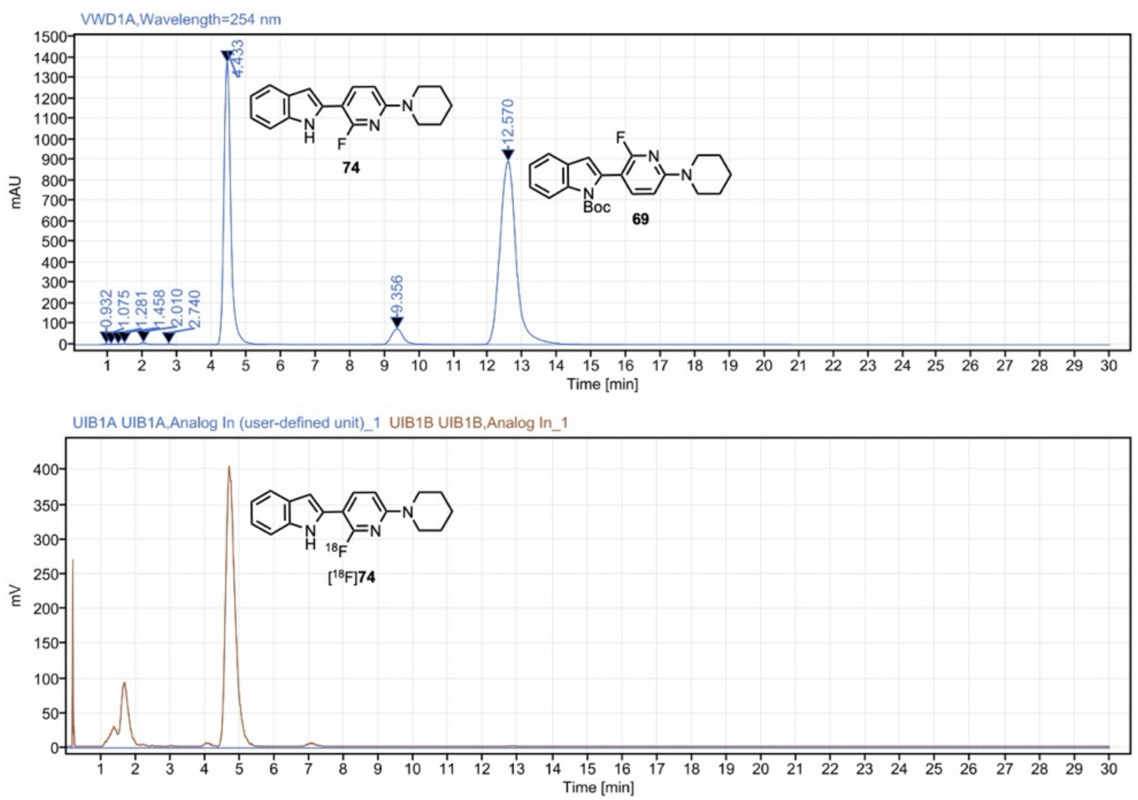

Figure S11. Analytical HPLC chromatograms of the [^{18}F]**74** crude reaction mixture after 45 min at ~140 °C, co-injected with compounds **69** and **74** (Phenomenex Gemini, 5 µm, NX-C18 110Å, 100 x 4.6 mm; 75:25 v/v CH_3CN/0.1 M NH_4HCO_2 pH 4.2; 1 mL/min; 254 nm). Note: the UV impurity at 9.36 min was in the sample of **69**.

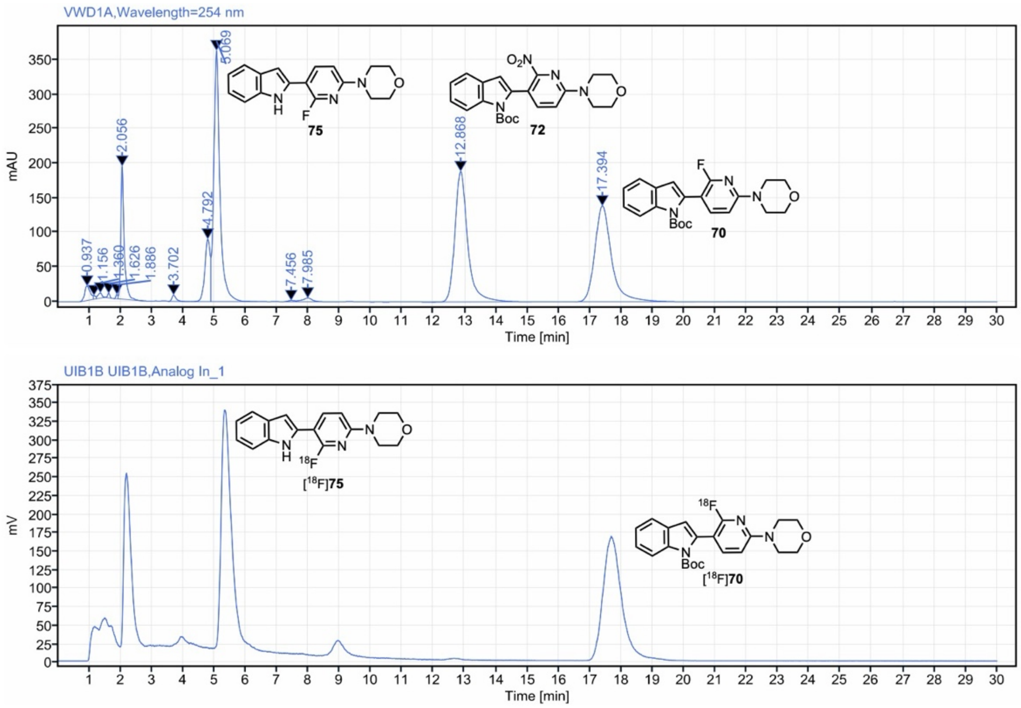

Figure S12. Analytical HPLC chromatograms of the [^{18}F]**75** crude reaction mixture after 15 min at ~140 °C, co-injected with compounds **70**, **72**, and **75** (Phenomenex Gemini, 5 µm, NX-C18 110Å, 100 x 4.6 mm; 55:45 v/v CH_3CN/0.1 M NH_4HCO_2 pH 4.2; 1 mL/min; 254 nm).

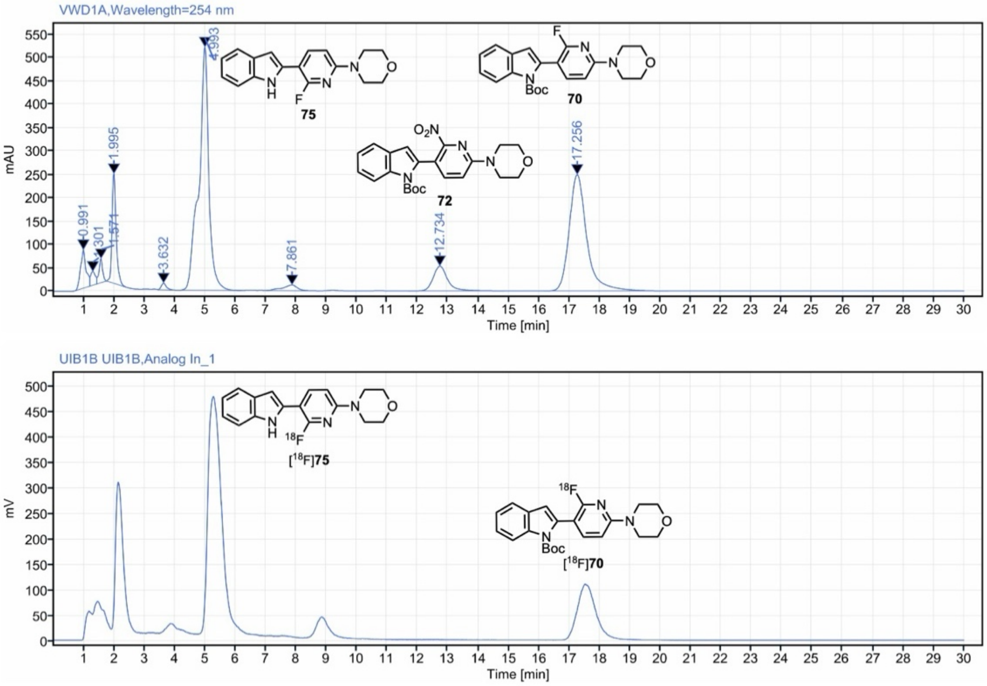

Figure S13. Analytical HPLC chromatograms of the [^{18}F]**75** crude reaction mixture after 30 min at ~140 °C, co-injected with compounds **70**, **72**, and **75** (Phenomenex Gemini, 5 µm, NX-C18 110Å, 100 x 4.6 mm; 55:45 v/v CH_3CN/0.1 M NH_4HCO_2 pH 4.2; 1 mL/min; 254 nm).

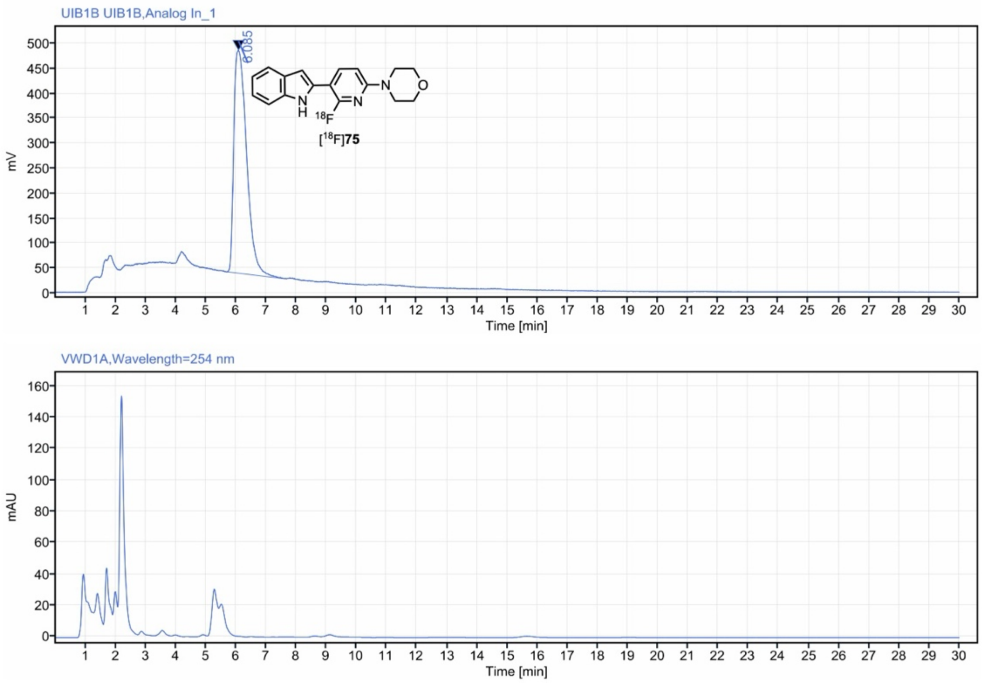

Figure S14. Analytical HPLC chromatograms of the [^{18}F]**75** crude reaction mixture after 45 min at ~140 ^{o}C (Phenomenex Gemini, 5 µm, NX-C18 110Å, 100 x 4.6 mm; 55:45 v/v CH_3CN/0.1 M NH_4HCO_2 pH 4.2; 1 mL/min; 254 nm).

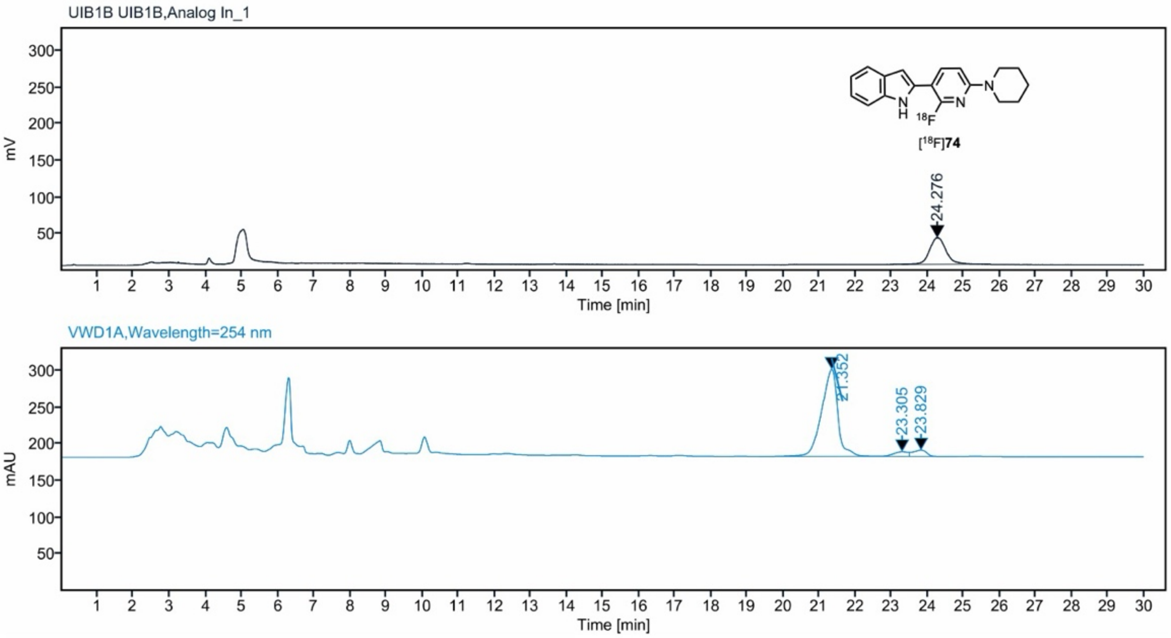

Figure S15. Semi-preparatory HPLC chromatogram of the purification of [^{18}F]**74** (Phenomenex Gemini 250 x 10 mm + guard; 55:45 v/v CH_3CN/0.1 M NH_4HCO_2 pH 4.2; 5 mL/min for 6 min, then 10 mL/min).

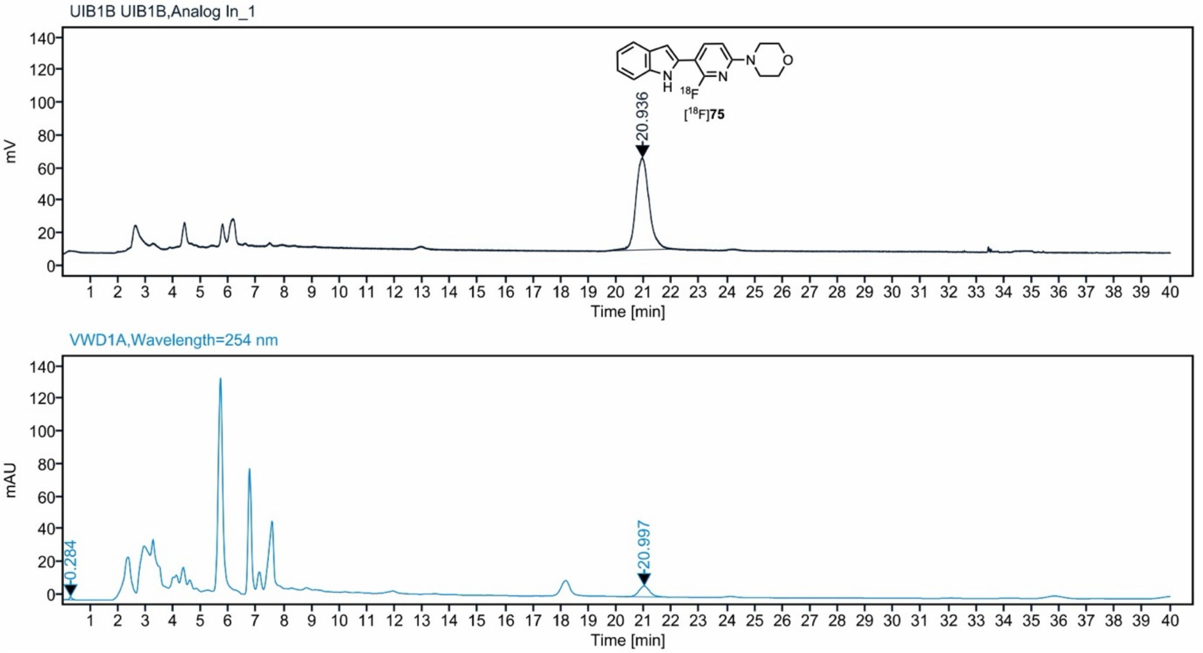

Figure S16. Semi-preparatory HPLC chromatogram of the purification of [^{18}F]**75** (Phenomenex Gemini 250 x 10 mm + guard; 45:55 v/v CH_3CN/0.1 M NH_4HCO_2 pH 4.2; 5 mL/min for 6 min, then 8 mL/min).

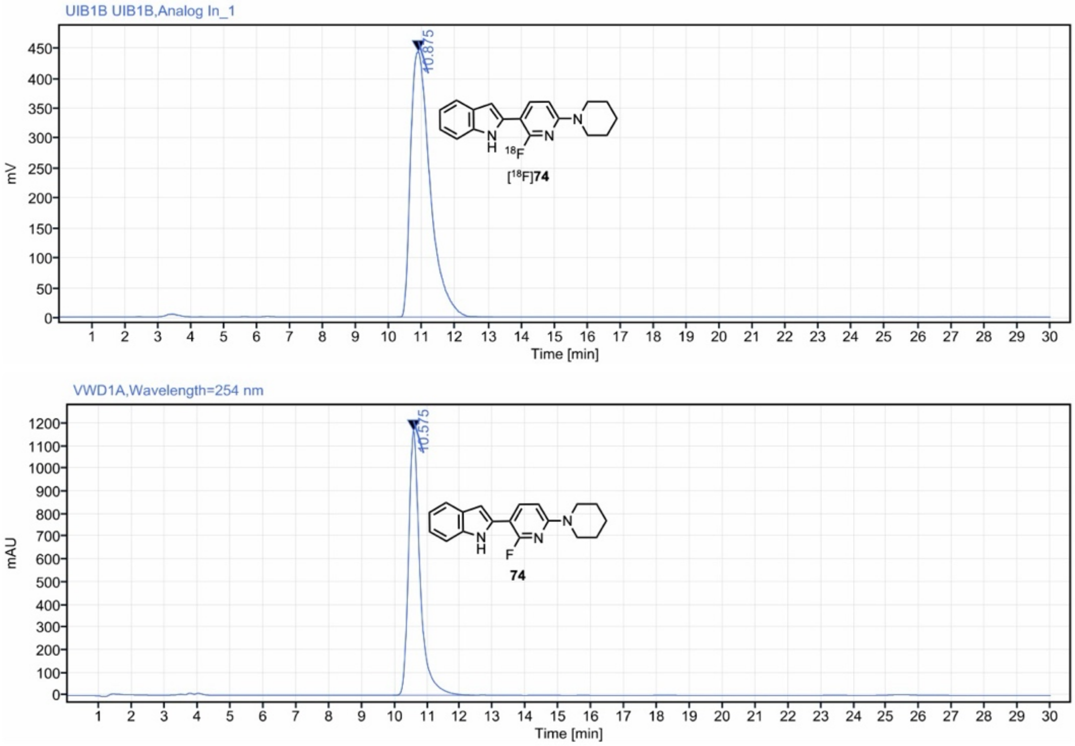

Figure S17. Analytical HPLC chromatograms of the [^{18}F]**74** final product co-injected with **74** (Phenomenex Gemini, 5 μm, NX-C18 110Å, 100 x 4.6 mm; 60:40 v/v CH_3CN/0.1 M NH_4HCO_2 pH 4.2; 1 mL/min; 254 nm).

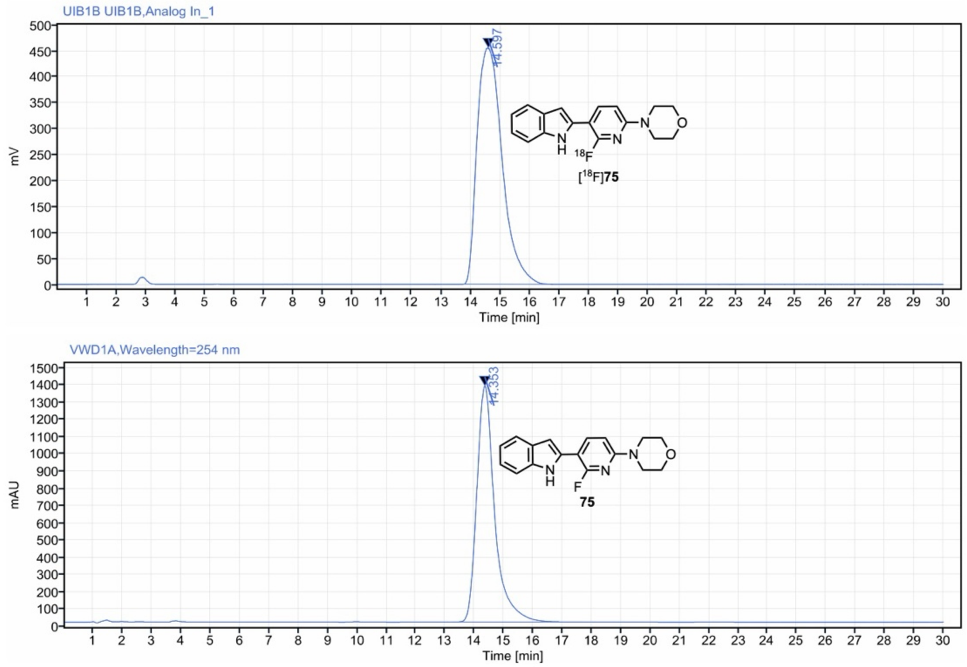

Figure S18. Analytical HPLC chromatograms of the [^{18}F]**75** final product co-injected with **75** (Phenomenex Gemini, 5 µm, NX-C18 110Å, 100 x 4.6 mm; 45:55 v/v CH_3CN/0.1 M NH_4HCO_2 pH 4.2; 1 mL/min; 254 nm).

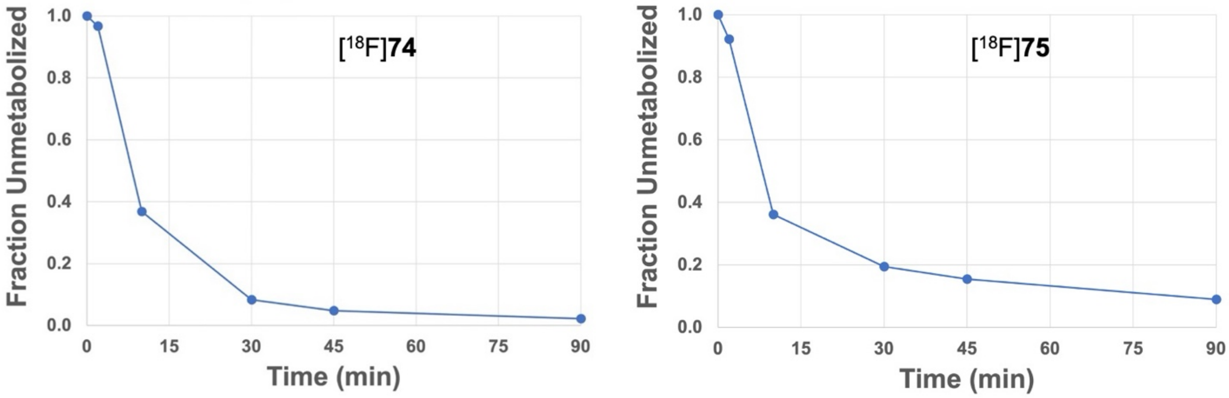

Figure S19. Unmetabolized venous fraction of [^{18}F]**74** (left) and [^{18}F]**75** (right) in male rhesus macaques.

Compound I.D.	Client Compound I.D.	IC_{50}(M)	nH	Test Concentration	% Inhibition of Control Values 1st	2nd	3rd	Mean
**MAO-B *(h)* recombinant enzyme**								
100071803-1	JSS20-183A	3.0E-06 M	1.6	1.0E-08 M	13.6	9.2	8.7	10.5
				3.0E-08 M	6.3	5.7	3.9	5.3
				1.0E-07 M	6.1	5.1	7.6	6.3
				2.0E-07 M	7.9	9.5	6.8	8.1
				5.0E-07 M	10.8	8.4	14.6	11.2
				1.0E-06 M	11.8	28.1	29.1	23.0
				2.0E-06 M	24.3	25.6	22.0	24.0
				5.0E-06 M	65.9	57.6	57.7	60.4
				1.0E-05 M	65.2	64.9	65.5	65.2
				2.0E-05 M	67.5	73.0	70.4	70.3
				5.0E-05 M	75.6	74.4	73.2	74.4
				1.0E-04 M	80.2	76.4	78.3	78.3

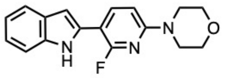

75
(JSS20-183A)

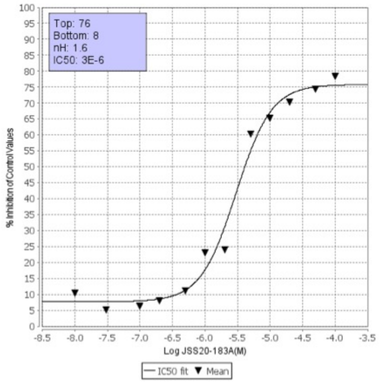

JSS20-183A on MAO-B *(h)* recombinant enzyme

Reference Compound Results

Compound I.D.	IC_{50}(M)	nH
**MAO-B *(h)* recombinant enzyme**		
deprenyl	5.0E-08 M	1.4

Figure S20. Results of Eurofins IC_{50} determination of **75** (JSS20-183A) at human MAO-B.

Table S9. Selected demographics of cases used for autoradiography and immunohistochemistry.

Case patient (P)/(C)	Gender (M/F)	Age of onset (Years)	Age at death (Years)	Tau	Aβ	α-syn	TDP-43	Region used	Diagnosis
P	F		80	3+	2+	0	0	Parietal	PSP
P	M		86	3+	0	0	0	Frontal	CBD
P	F		70	3+	3+	0	0	Parietal	AD
C	M		66	0	0	0	0	Cingulate gyrus	CT

Table S10. Primary antibody selected for immunohistochemistry.

Primary Antibodies	Source/Cat No	Host and clonality	Antibody dilution used	Incubation time
Phospho-Tau (Ser202, Thr205) Monoclonal Antibody (AT8)	Thermo Fisher Scientific #MN1020	Mouse monoclonal	1:500	Overnight at 4 ºC
Anti-beta Amyloid antibody [mOC23]	Abcam/ab205340	Rabbit monoclonal	1:500	Overnight at 4 ºC

Table S11. Secondary antibody selected for immunohistochemistry.

Secondary Antibody	Source/Cat No	Host and clonality	Antibody dilution used	Incubation time
Goat Anti-Mouse IgG H&L	Abcam/ab205719	Goat polyclonal	1:10000	1 h at ambient temperature
Goat Anti-Rabbit IgG H&L	Ab97051	Goat polyclonal	1:10000	1h at ambient temperature

Table S12. Sample and crystal data for **44** (CCDC Deposition Number 2403956).

**Identification code**	JSS20-116A	
**Chemical formula**	$C_{17}H_{15}FN_4O$	
**Formula weight**	310.33 g/mol	
**Temperature**	296(2) K	
**Wavelength**	1.54178 Å	
**Crystal size**	0.003 x 0.120 x 0.200 mm	
**Crystal habit**	clear colorless plate	
**Crystal system**	monoclinic	
**Space group**	P 1 21/n 1	
**Unit cell dimensions**	a = 12.5190(4) Å	α = 90°
	b = 7.8750(4) Å	β = 90.611(3)°
	c = 15.0667(5) Å	γ = 90°
**Volume**	1485.30(10) $Å^3$	
**Z**	4	
**Density (calculated)**	1.388 g/cm^3	
**Absorption coefficient**	0.817 mm^{-1}	
**F(000)**	648	

Table S13. Sample and crystal data for **71** (CCDC Deposition Number 2403955).

**Identification code**	JSS21-021B	
**Chemical formula**	$C_{23}H_{26}N_4O_4$	
**Formula weight**	422.48 g/mol	
**Temperature**	296(2) K	
**Wavelength**	1.54178 Å	
**Crystal size**	0.050 x 0.120 x 0.190 mm	
**Crystal habit**	clear yellow Chunk	
**Crystal system**	orthorhombic	
**Space group**	P 21 21 21	
**Unit cell dimensions**	a = 10.3939(2) Å	α = 90°
	b = 12.8366(3) Å	β = 90°
	c = 16.0313(4) Å	γ = 90°
**Volume**	2138.93(8) $Å^3$	
**Z**	4	
**Density (calculated)**	1.312 g/cm^3	
**Absorption coefficient**	0.747 mm^{-1}	
**F(000)**	896	

Table S14. Sample and crystal data for **72** (CCDC Deposition Number 2403957).

**Identification code**	JSS21-033A	
**Chemical formula**	$C_{22}H_{24}N_4O_5$	
**Formula weight**	424.45 g/mol	
**Temperature**	150(2) K	
**Wavelength**	1.54178 Å	
**Crystal size**	0.004 x 0.120 x 0.200 mm	
**Crystal system**	monoclinic	
**Space group**	P 1 21/c 1	
**Unit cell dimensions**	a = 8.68420(10) Å	α = 90°
	b = 12.4480(2) Å	β = 91.5310(10)°
	c = 19.3489(3) Å	γ = 90°
**Volume**	2090.89(5) $Å^3$	
**Z**	4	
**Density (calculated)**	1.348 g/cm^3	
**Absorption coefficient**	0.805 mm^{-1}	
**F(000)**	896	

Table S15. Sample and crystal data for **75** (CCDC Deposition Number 2403958).

**Identification code**	JSS20-183A	
**Chemical formula**	$C_{17}H_{16}FN_3O$	
**Formula weight**	297.33 g/mol	
**Temperature**	100(2) K	
**Wavelength**	0.71073 Å	
**Crystal size**	0.050 x 0.120 x 0.170 mm	
**Crystal habit**	clear colorless block	
**Crystal system**	monoclinic	
**Space group**	P 1 21/c 1	
**Unit cell dimensions**	a = 16.3426(5) Å	α = 90°
	b = 5.9494(2) Å	β = 105.0520(10)°
	c = 14.4881(4) Å	γ = 90°
**Volume**	1360.33(7) $Å^3$	
**Z**	4	
**Density (calculated)**	1.452 g/cm^3	
**Absorption coefficient**	0.102 mm^{-1}	
**F(000)**	624	

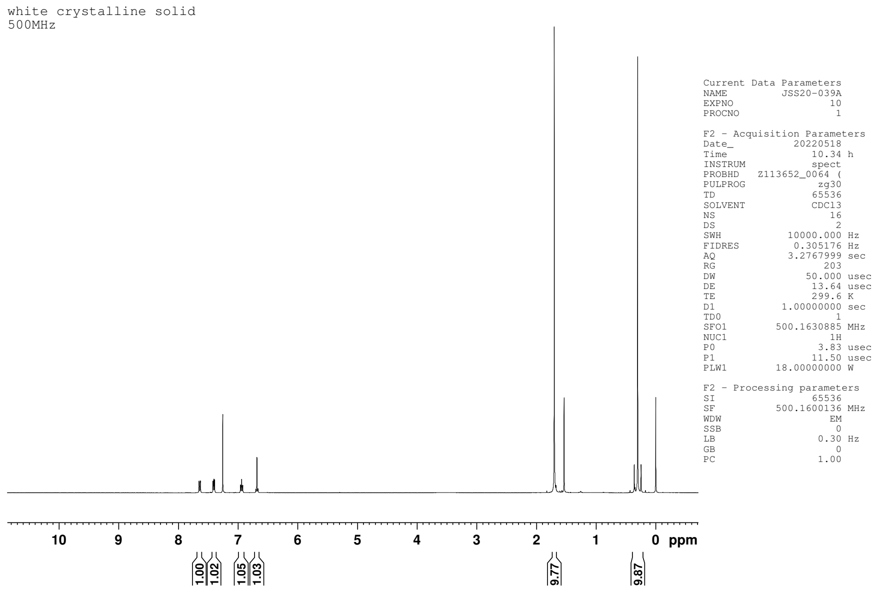

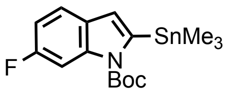

10

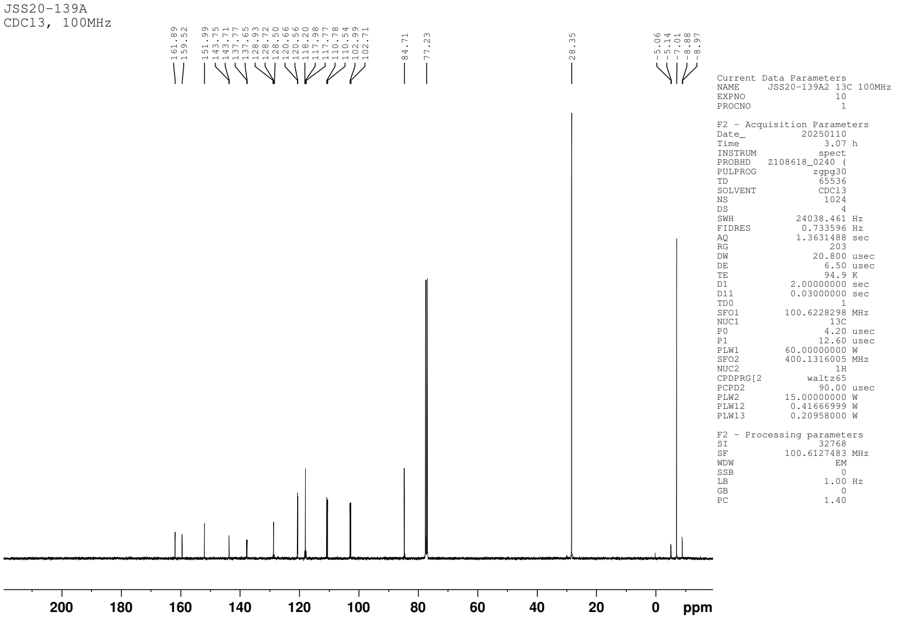

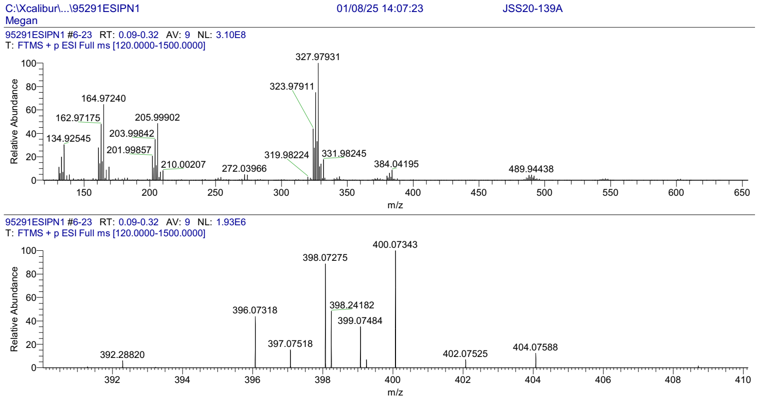

95291ESIPN1#7-23 RT: 0.09-0.32 AV: 9
T: FTMS + p ESI Full ms [120.0000-1500.0000]
m/z= 390.01313-410.13240

m/z	Intensity	Relative	Theo. Mass	Delta (ppm)	Composition
400.07343	1997257.5	100.00	400.07293	1.26	$C_{16}H_{23}O_2NFSn$

Figure S21. Compound **10** ^{1}H NMR spectrum (top), ^{13}C NMR spectrum (middle), and high-resolution mass spectrum (bottom).

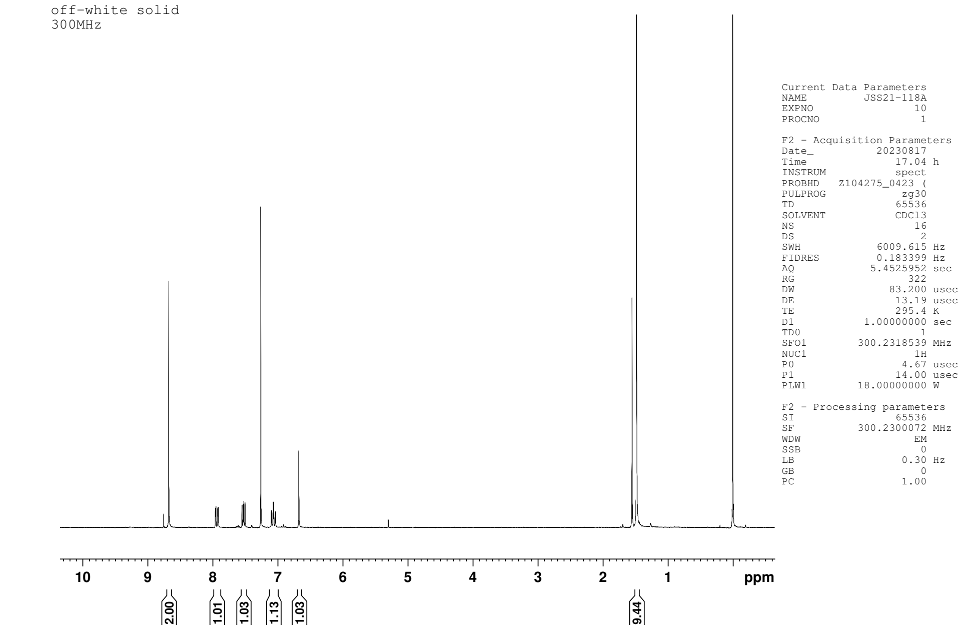

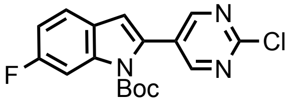

13

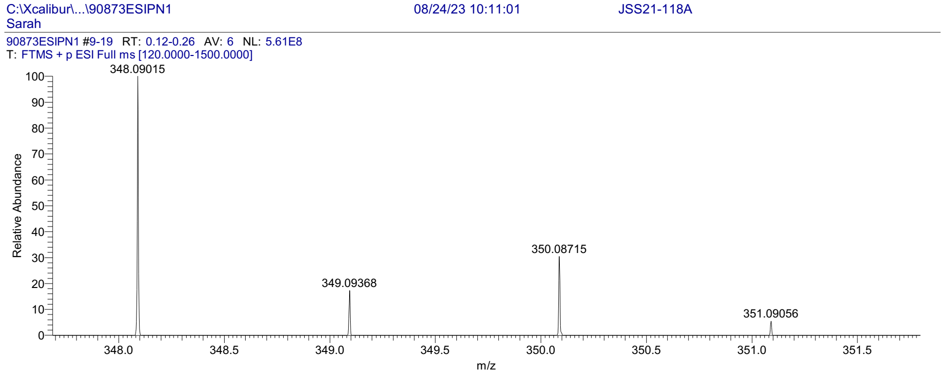

90873ESIPN1#9-19 RT: 0.12-0.26 AV: 6
T: FTMS + p ESI Full ms [120.0000-1500.0000]
m/z= 347.68580-351.79755

m/z	Intensity	Relative	Theo. Mass	Delta (ppm)	Composition
348.09015	562407296.0	100.00	348.09096	-2.33	$C_{17}H_{16}O_2N_3ClF$

Figure S22. Compound **13** ^{1}H NMR spectrum (top) and high-resolution mass spectrum (bottom).

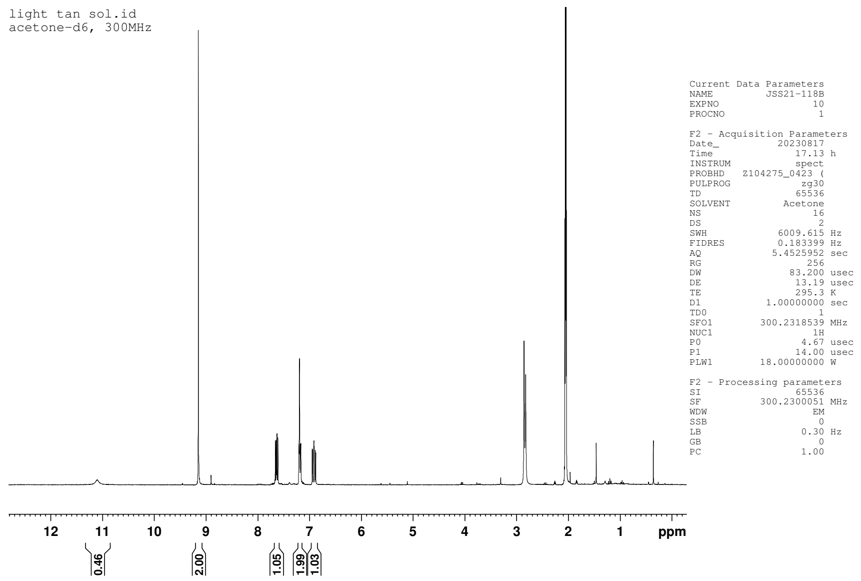

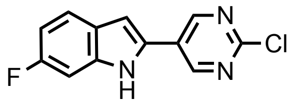

14

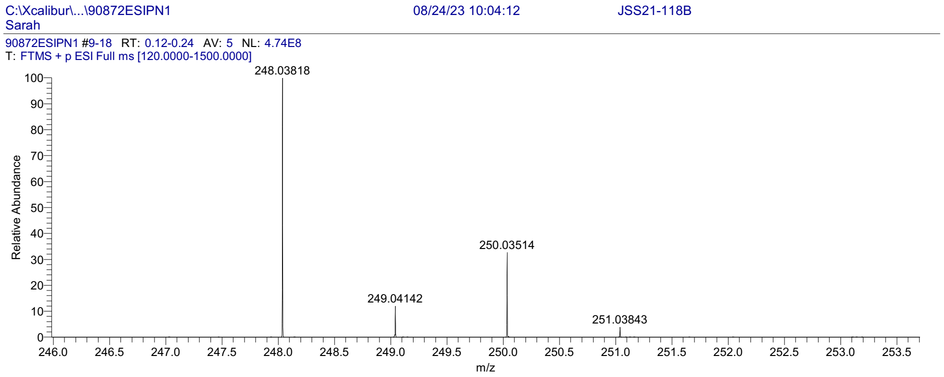

90872ESIPN1#9-17 RT: 0.12-0.24 AV: 5
T: FTMS + p ESI Full ms [120.0000-1500.0000]
m/z= 245.98475-253.70373

m/z	Intensity	Relative	Theo. Mass	Delta (ppm)	Composition
248.03818	474728576.0	100.00	248.03853	-1.41	$C_{12}H_8N_3ClF$

Figure S23. Compound **14** ^{1}H NMR spectrum (top) and high-resolution mass spectrum (bottom).

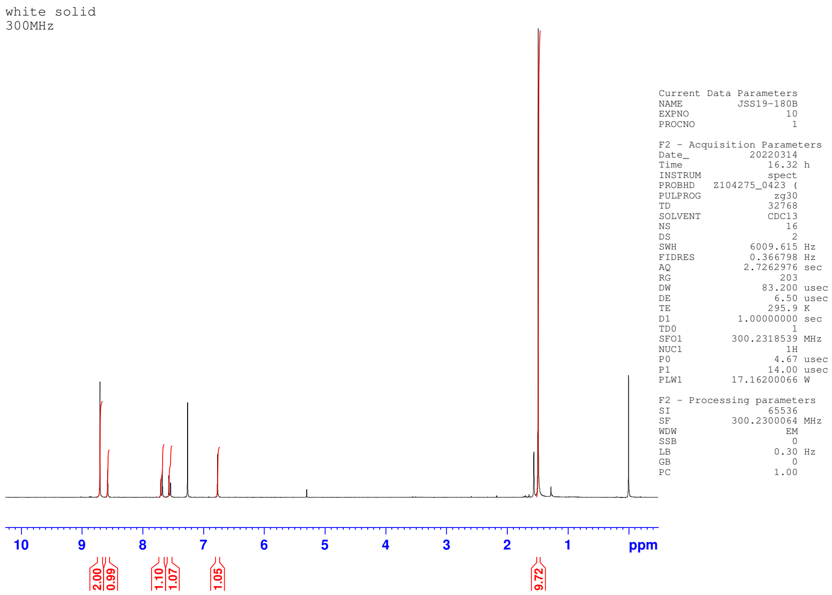

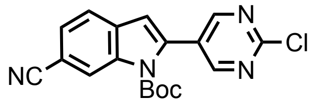

15

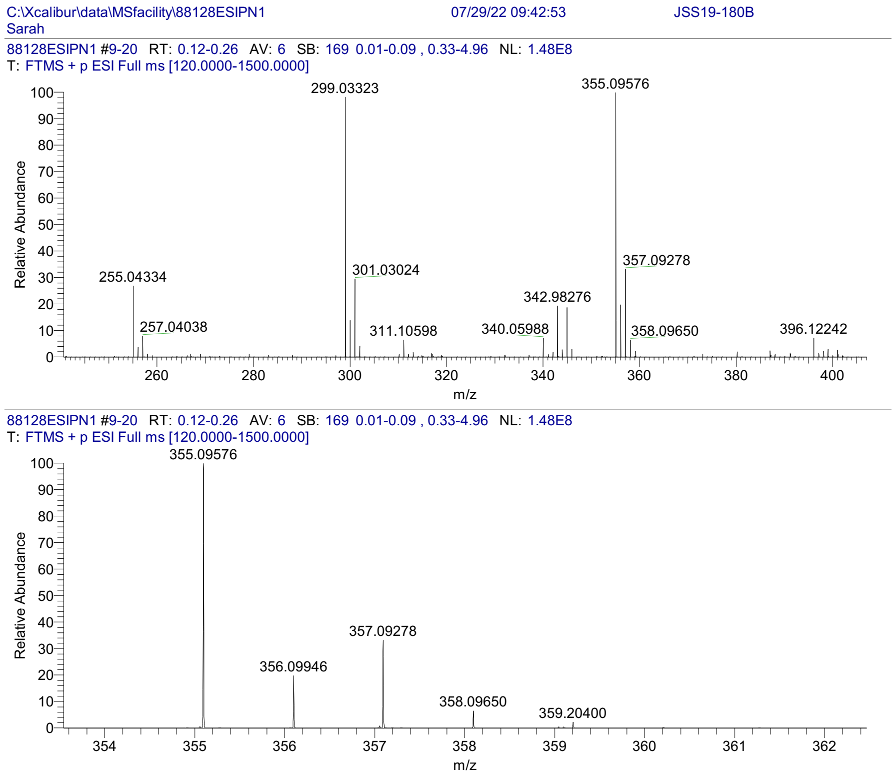

88128ESIPN1#9-19 RT: 0.12-0.26 AV: 6
SB: 169 0.01-0.09 , 0.33-4.96
T: FTMS + p ESI Full ms [120.0000-1500.0000]
m/z= 353.54046-362.46545

m/z	Intensity	Relative	Theo. Mass	Delta (ppm)	Composition
355.09576	151910704.0	100.00	355.09563	0.13	$C_{18}H_{16}O_2N_4Cl$

Figure S24. Compound **15** ^{1}H NMR spectrum (top) and high-resolution mass spectrum (bottom).

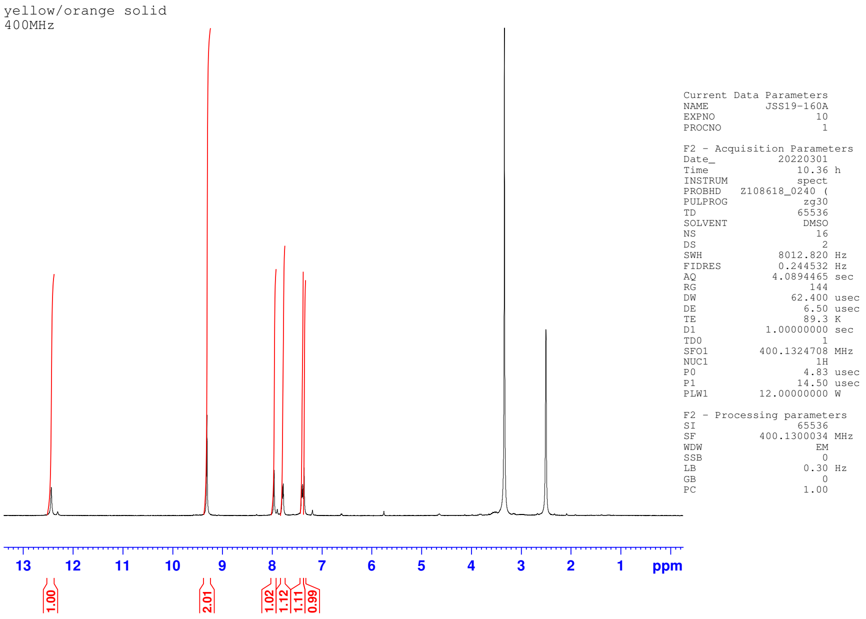

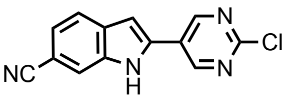

16

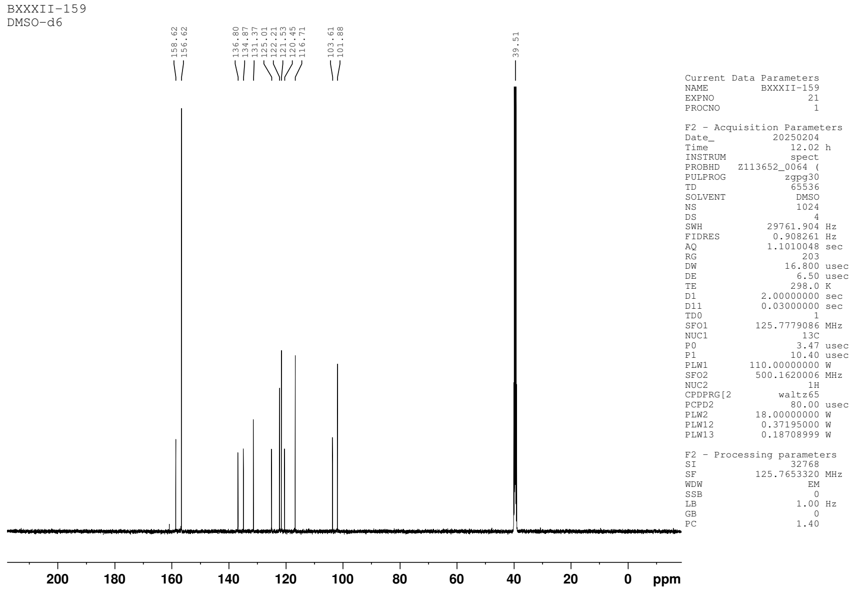

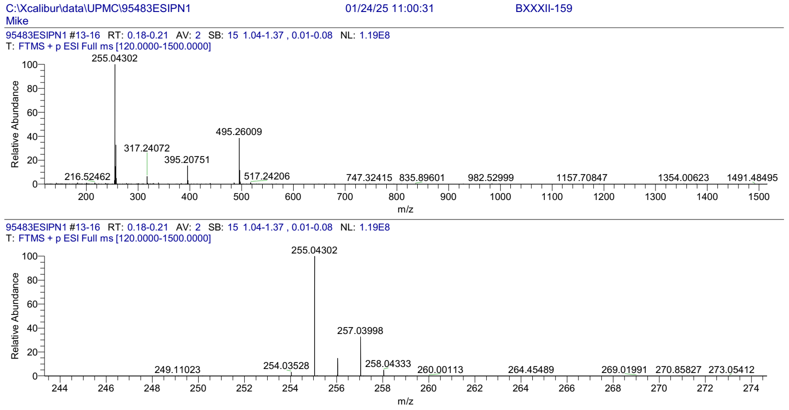

95483ESIPN1#13-15 RT: 0.18-0.21 AV: 2
SB: 15 1.04-1.37 , 0.01-0.08
T: FTMS + p ESI Full ms [120.0000-1500.0000]
m/z= 243.31745-274.67560

m/z	Intensity	Relative	Theo. Mass	Delta (ppm)	Composition
255.04302	123307864.0	100.00	255.04320	-0.72	$C_{13} H_8 N_4 Cl$

Figure S25. Compound **16** ^{1}H NMR spectrum (top), ^{13}C NMR spectrum (middle), high-resolution mass spectrum (bottom).

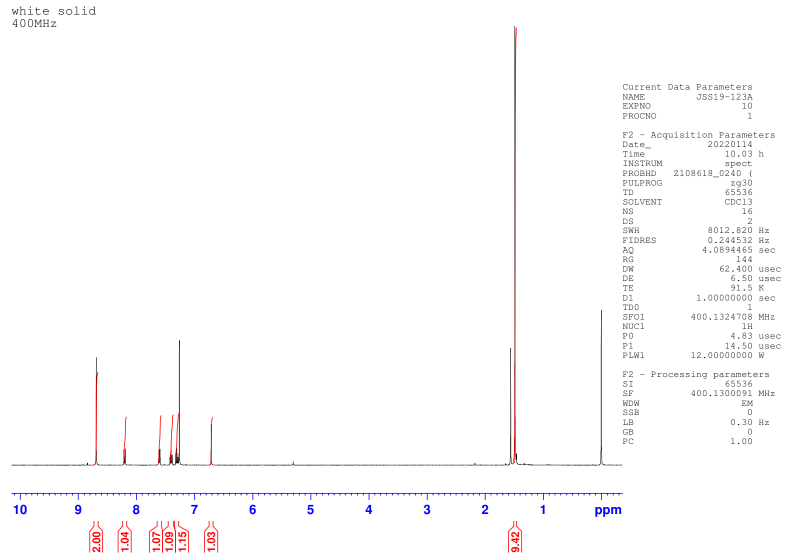

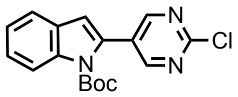

17

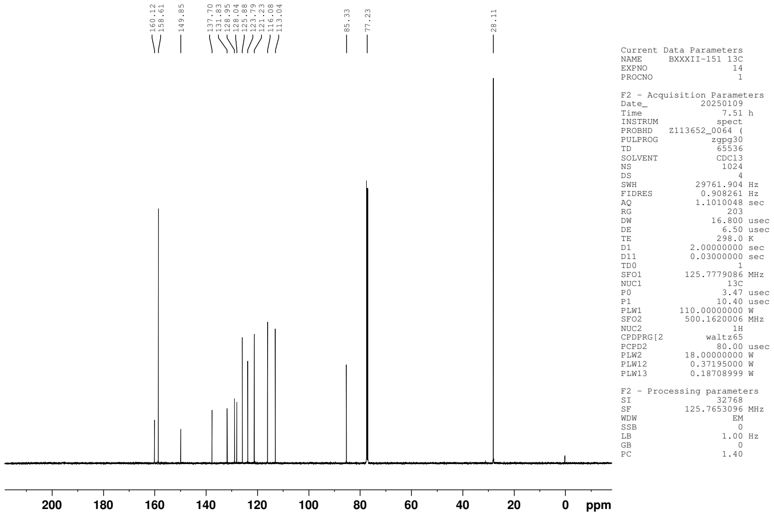

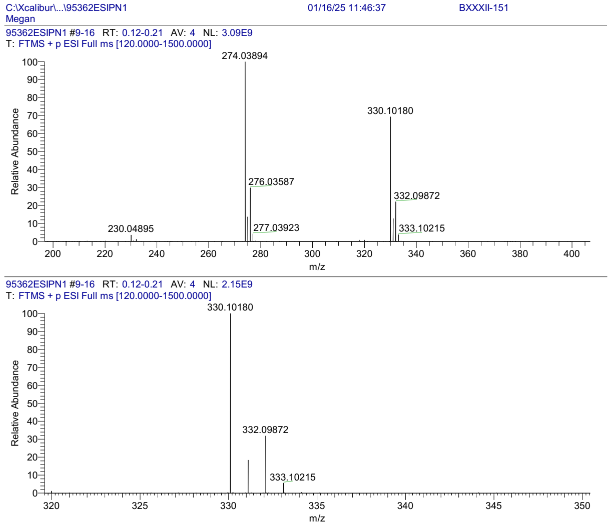

95362ESIPN1#9-15 RT: 0.12-0.21 AV: 4
T: FTMS + p ESI Full ms [120.0000-1500.0000]
m/z= 319.58971-350.43120

m/z	Intensity	Relative	Theo. Mass	Delta (ppm)	Composition
330.10180	2156589312.0	100.00	330.10038	4.29	$C_{17}H_{17}O_2N_3Cl$

Figure S26. Compound **17** ^{1}H NMR spectrum (top), ^{13}C NMR spectrum (middle), and high-resolution mass spectrum (bottom).

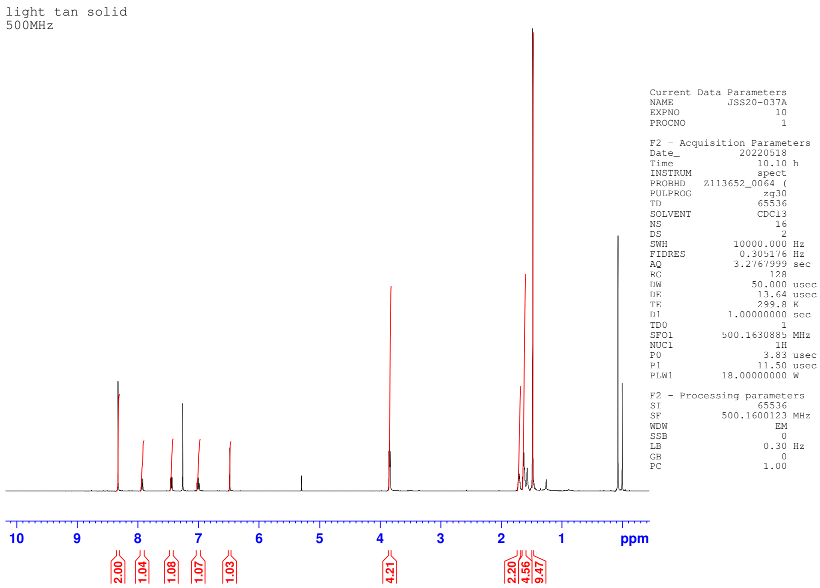

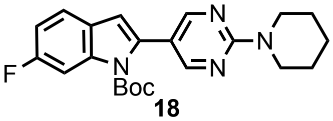

18

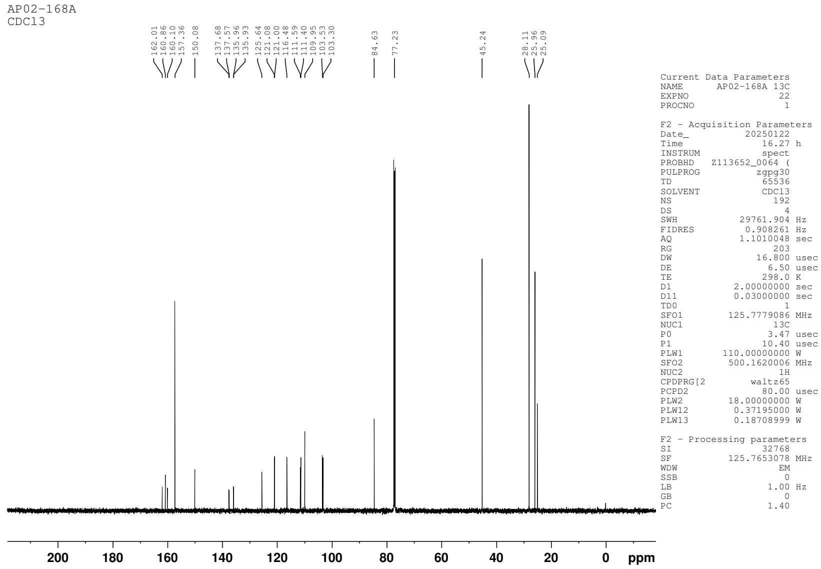

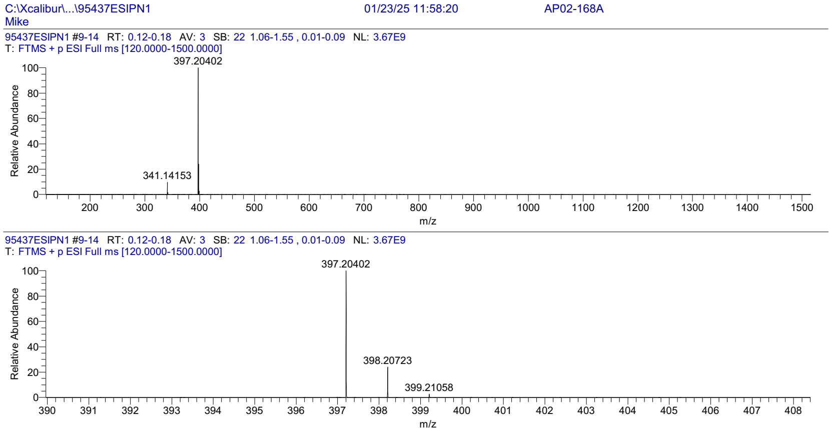

95437ESIPN1#9-13 RT: 0.12-0.18 AV: 3
SB: 22 1.06-1.55 , 0.01-0.09
T: FTMS + p ESI Full ms [120.0000-1500.0000]
m/z= 389.95883-408.40457

m/z	Intensity	Relative	Theo. Mass	Delta (ppm)	Composition
397.20402	3760296448.0	100.00	397.20343	1.49	$C_{22}H_{26}O_2N_4F$

Figure S27. Compound **18** ^{1}H NMR spectrum (top), ^{13}C NMR spectrum (middle), high-resolution mass spectrum (bottom).

white foam

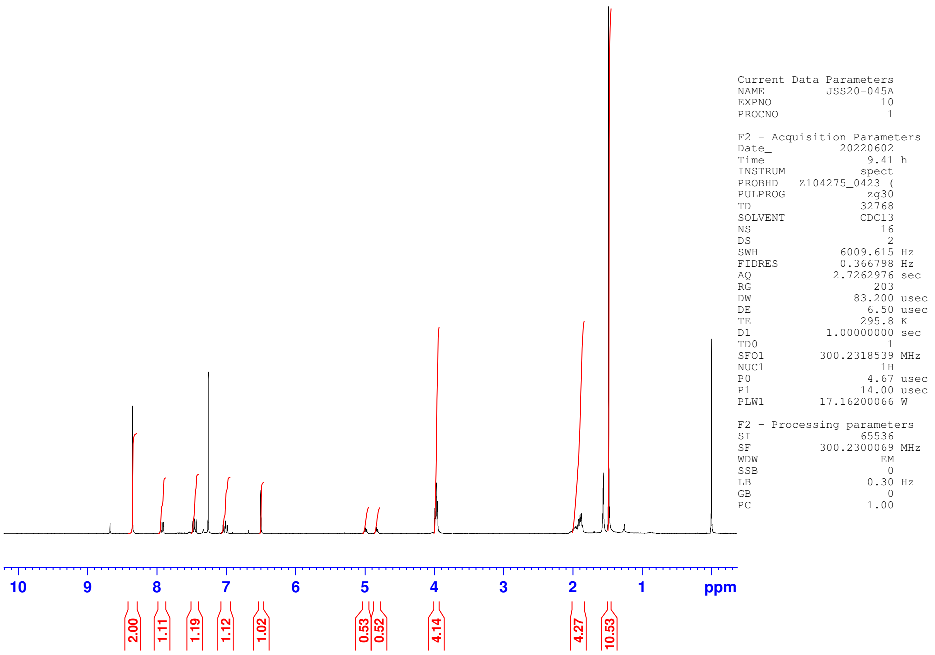

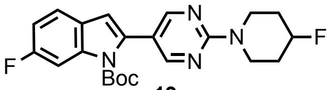

19

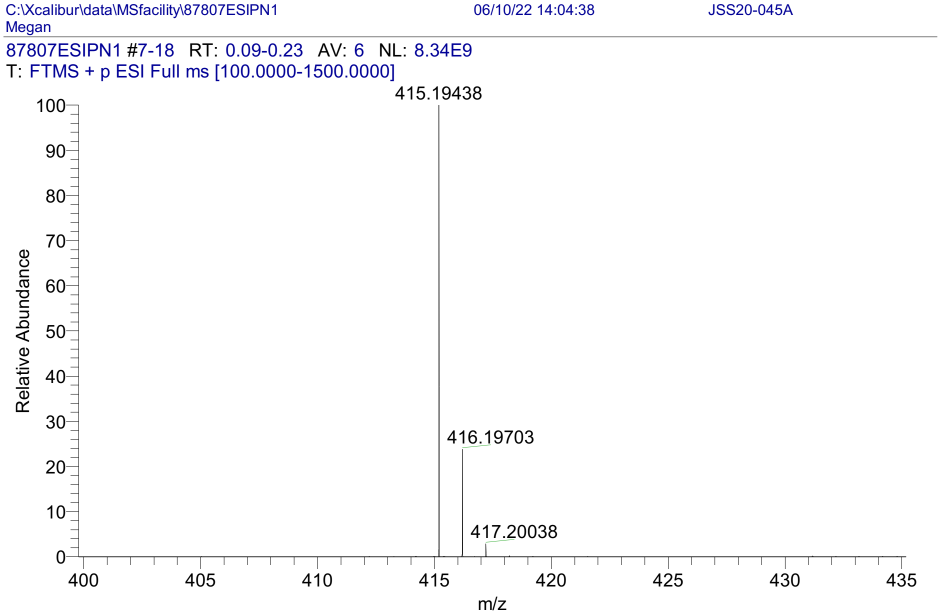

87807ESIPN1#7-17 RT: 0.09-0.23 AV: 6
T: FTMS + p ESI Full ms [100.0000-1500.0000]
m/z= 399.76971-435.17407

m/z	Intensity	Relative	Theo. Mass	Delta (ppm)	Composition
415.19438	8486300160.0	100.00	415.19401	0.88	$C_{22}H_{25}O_2N_4F_2$

Figure S28. Compound **19** ^{1}H NMR spectrum (top) and high-resolution mass spectrum (bottom).

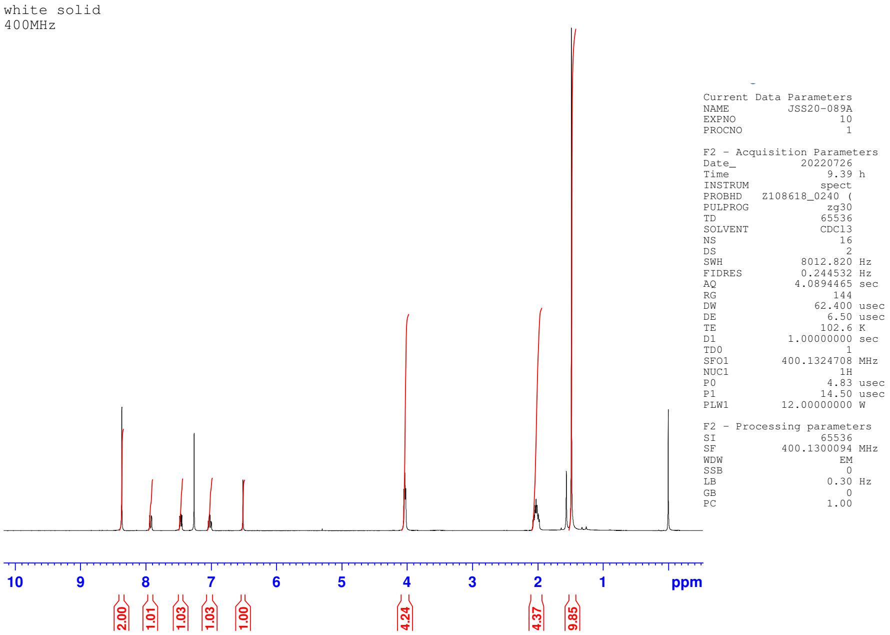

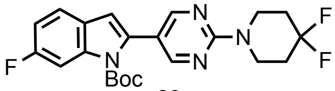

20

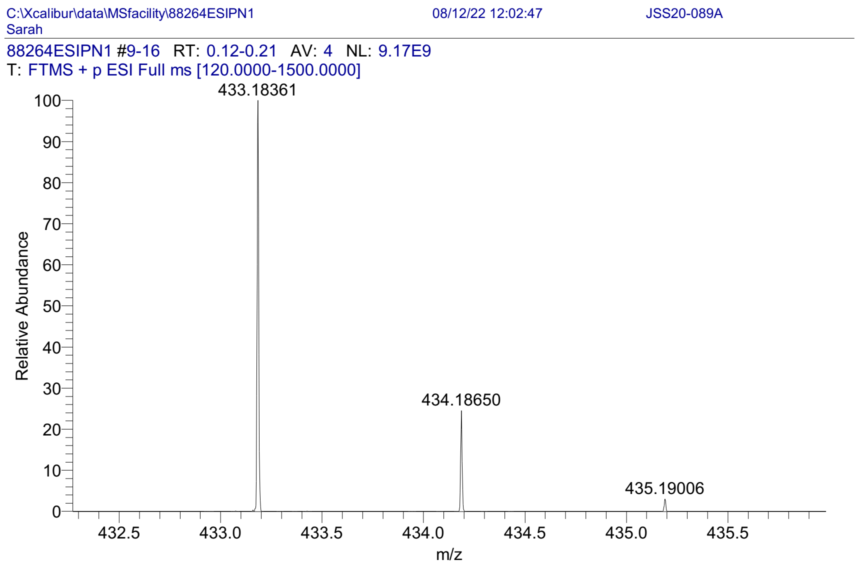

88264ESIPN1#9-15 RT: 0.12-0.21 AV: 4
T: FTMS + p ESI Full ms [120.0000-1500.0000]
m/z= 432.27043-435.98292

m/z	Intensity	Relative	Theo. Mass	Delta (ppm)	Composition
433.18361	9352170496.0	100.00	433.18459	-2.25	$C_{22} H_{24} O_2 N_4 F_3$

Figure S29. Compound **20** ^{1}H NMR spectrum (top) and high-resolution mass spectrum (bottom).

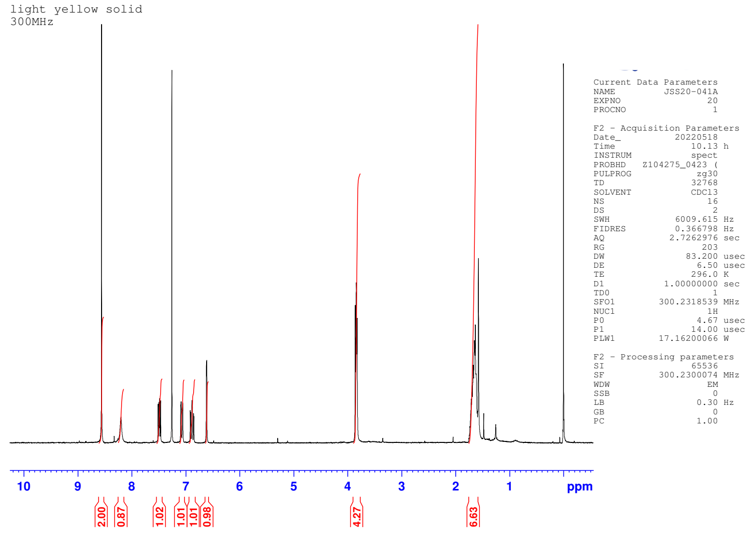

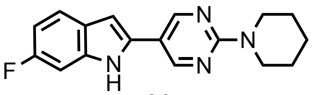

21

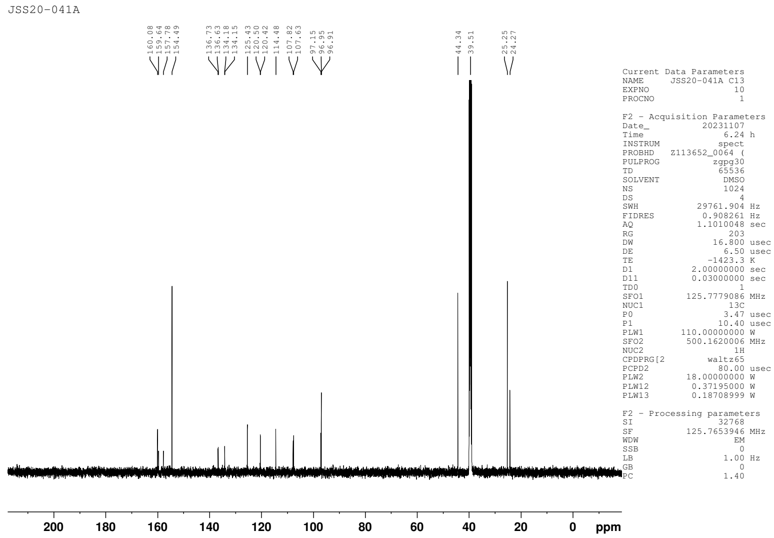

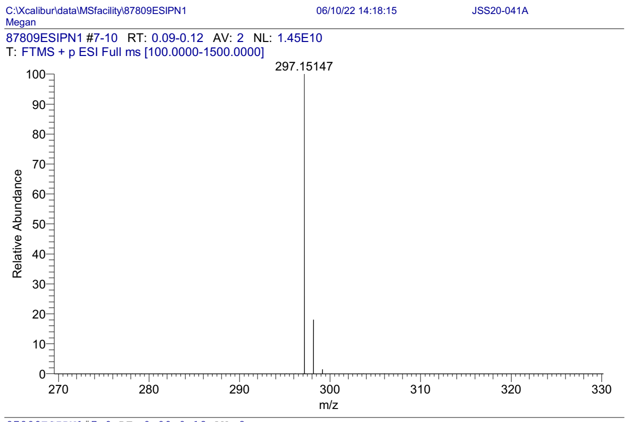

87809ESIPN1#7-9 RT: 0.09-0.12 AV: 2
T: FTMS + p ESI Full ms [100.0000-1500.0000]
m/z= 269.51625-330.17490

m/z	Intensity	Relative	Theo. Mass	Delta (ppm)	Composition
297.15147	14697496576.0	100.00	297.15100	1.59	$C_{17}H_{18}N_4F$

Figure S30. Compound **21** ^{1}H NMR spectrum (top), ^{13}C NMR spectrum (middle), and high-resolution mass spectrum (bottom).

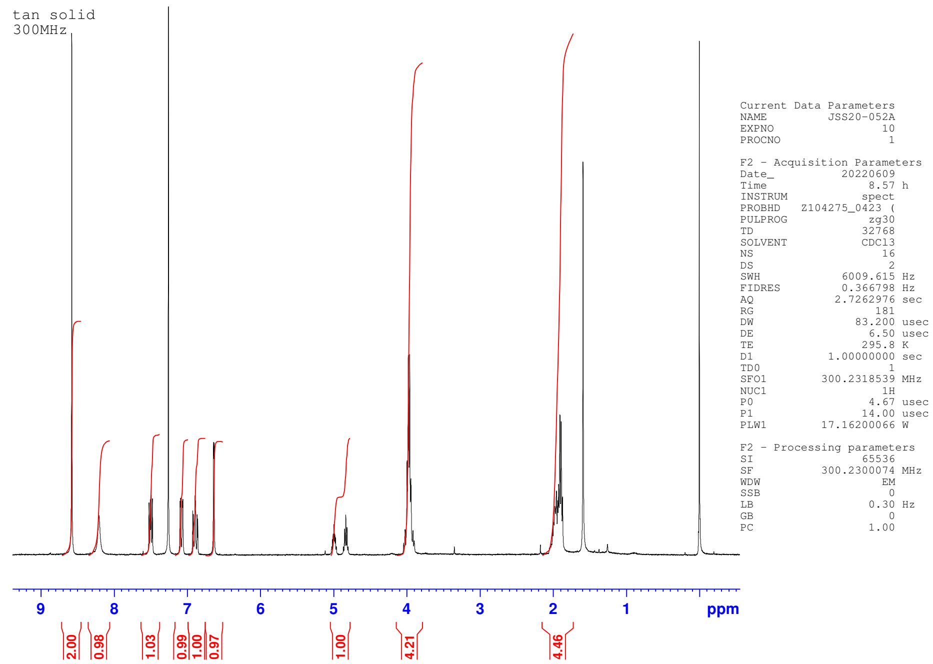

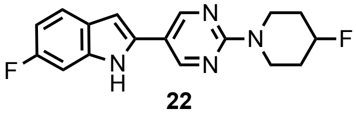

22

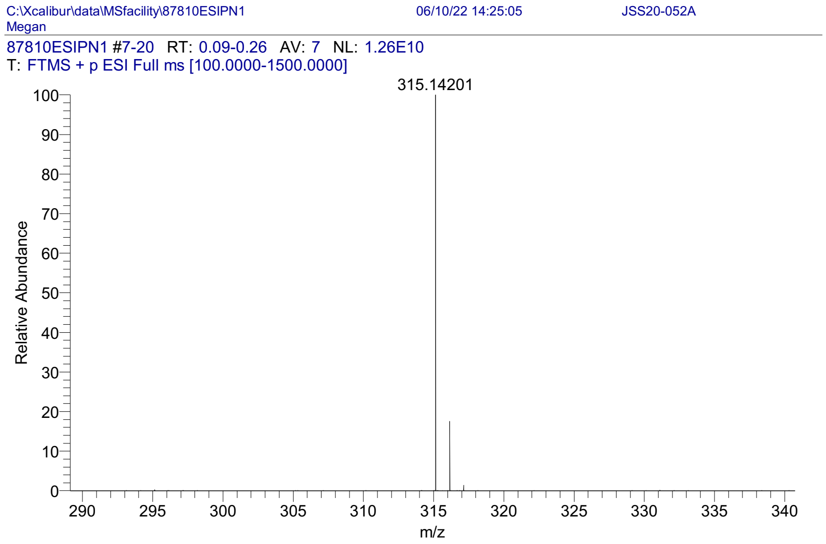

87810ESIPN1#7-19 RT: 0.09-0.26 AV: 7
T: FTMS + p ESI Full ms [100.0000-1500.0000]
m/z= 289.10871-340.72683

m/z	Intensity	Relative	Theo. Mass	Delta (ppm)	Composition
315.14201	12641028096.0	100.00	315.14158	1.35	$C_{17}H_{17}N_4F_2$

Figure S31. Compound **22** ^{1}H NMR spectrum (top) and high-resolution mass spectrum (bottom).

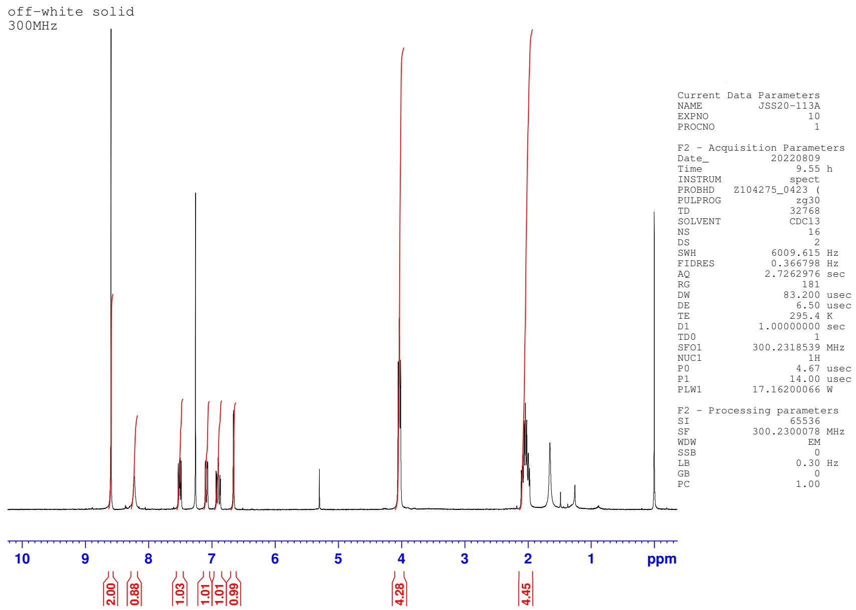

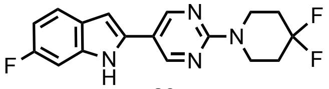

23

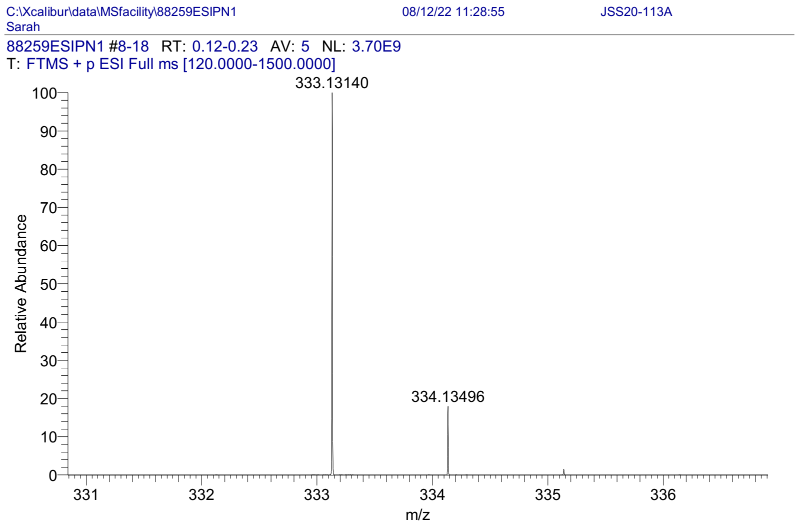

88259ESIPN1#9-17 RT: 0.12-0.23 AV: 5
T: FTMS + p ESI Full ms [120.0000-1500.0000]
m/z= 330.83944-336.90744

m/z	Intensity	Relative	Theo. Mass	Delta (ppm)	Composition
333.13140	3724583424.0	100.00	333.13216	-2.29	$C_{17}H_{16}N_4F_3$

Figure S32. Compound **23** ^{1}H NMR spectrum (top) and high-resolution mass spectrum (bottom).

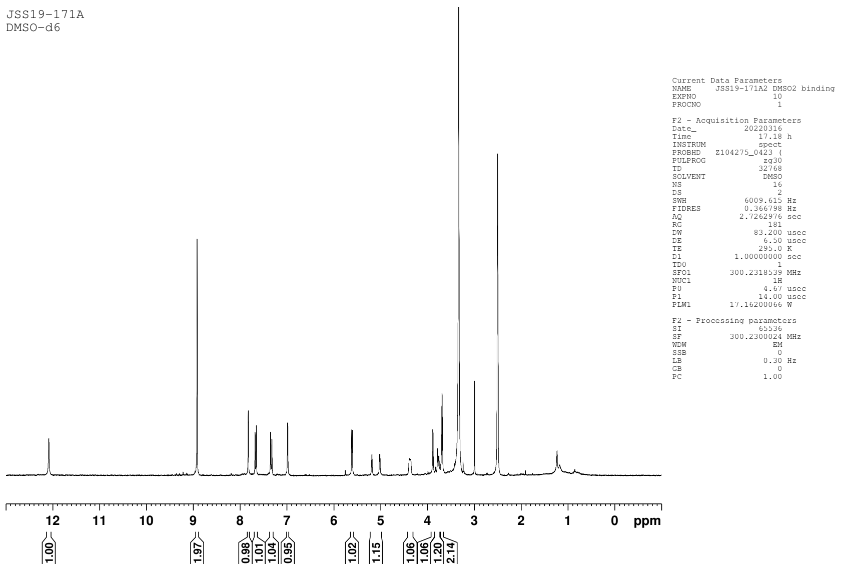

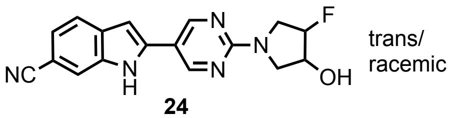

24

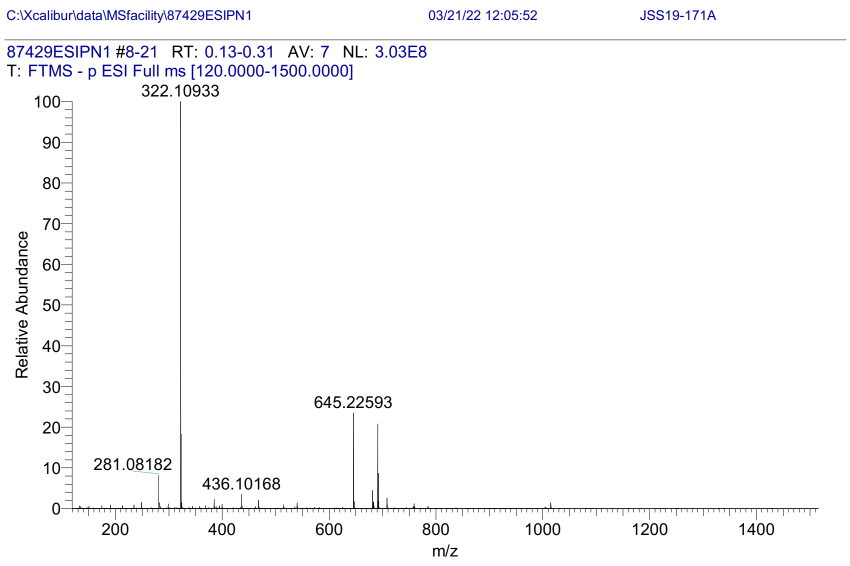

87429ESIPN1#8-20 RT: 0.13-0.31 AV: 7
T: FTMS - p ESI Full ms [120.0000-1500.0000]

m/z	Intensity	Relative	Theo. Mass	Delta (ppm)	Composition
322.10933	303485024.0	100.00	322.10986	-0.53	$C_{17}H_{13}ON_5F$

Figure S33. Compound **24** ^{1}H NMR spectrum (top) and high-resolution mass spectrum (bottom).

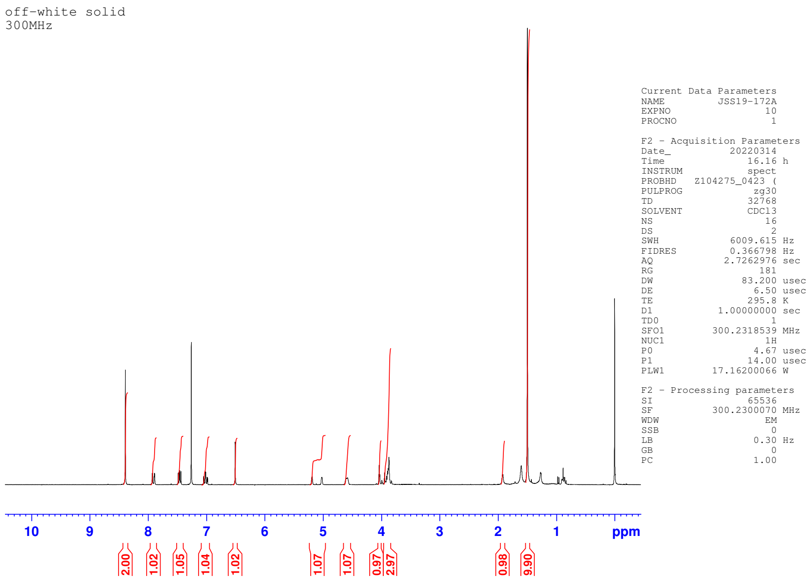

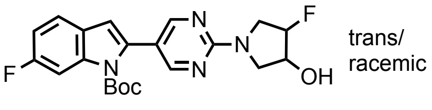

25

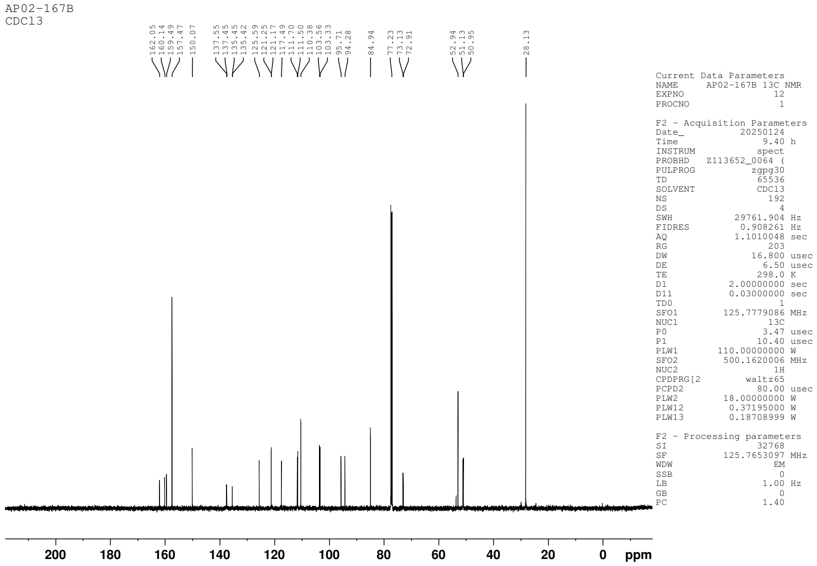

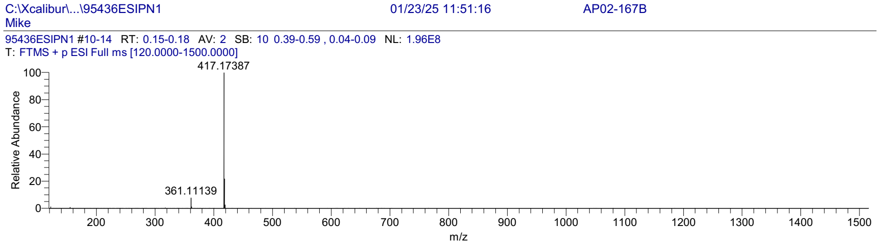

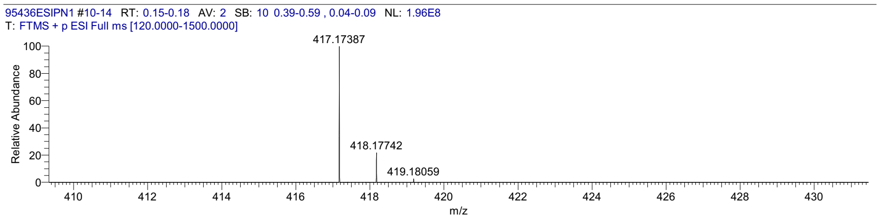

95436ESIPN1#11-13 RT: 0.15-0.18 AV: 2
SB: 10 0.39-0.59 , 0.04-0.09
T: FTMS + p ESI Full ms [120.0000-1500.0000]
m/z= 409.32826-431.46324

m/z	Intensity	Relative	Theo. Mass	Delta (ppm)	Composition
417.17387	203296880.0	100.00	417.17327	0.60	$C_{21}H_{23}O_3N_4F_2$

Figure S34. Compound **25** ^{1}H NMR spectrum (top), ^{13}C NMR spectrum (middle), and high-resolution mass spectrum (bottom).

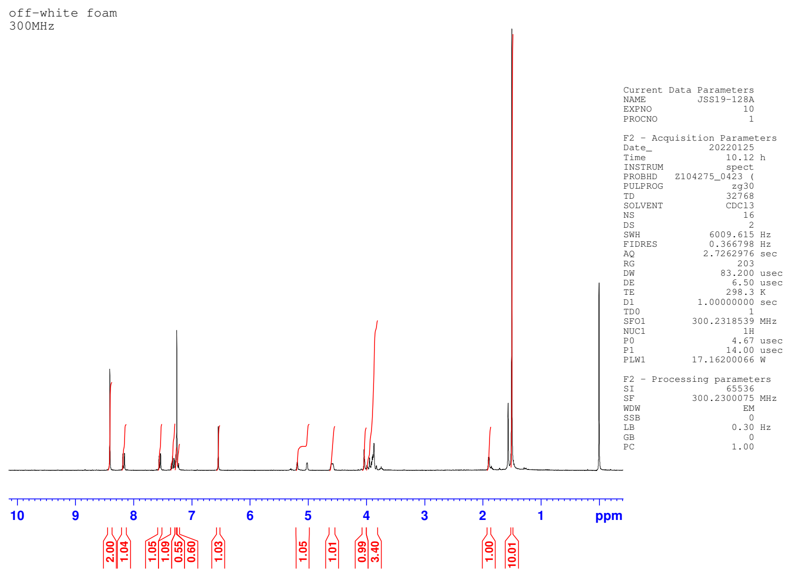

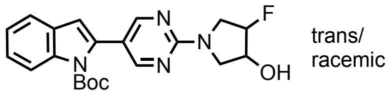

26

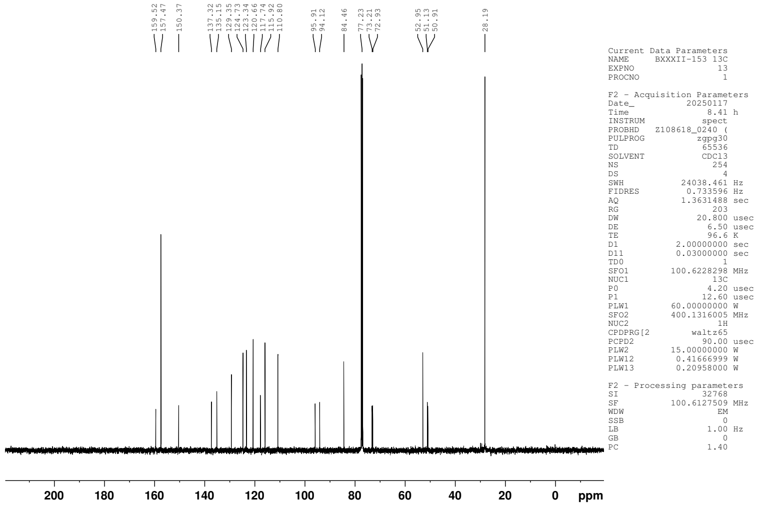

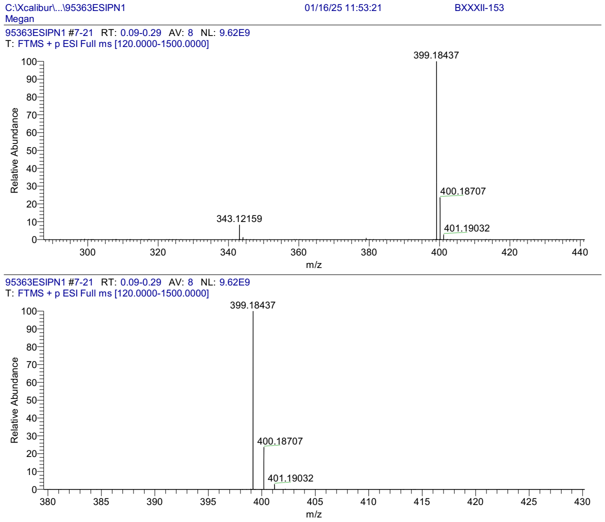

95363ESIPN1#7-21 RT: 0.09-0.29 AV: 8
T: FTMS + p ESI Full ms [120.0000-1500.0000]
m/z= 379.59164-430.18535

m/z	Intensity	Relative	Theo. Mass	Delta (ppm)	Composition
399.18437	9904142336.0	100.00	399.18270	4.21	$C_{21}H_{24}O_3N_4F$

Figure S35. Compound **26** ^{1}H NMR spectrum (top), ^{13}C NMR spectrum (middle), and high-resolution mass spectrum (bottom).

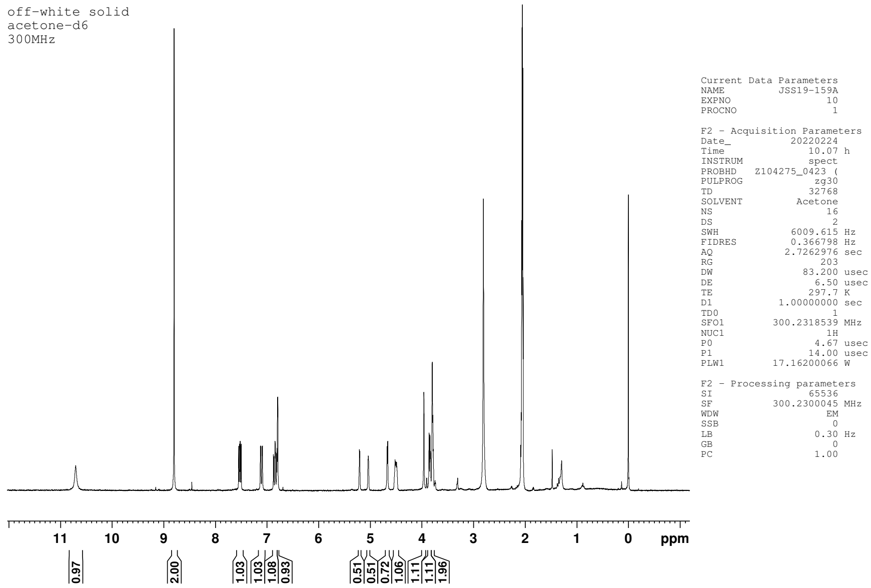

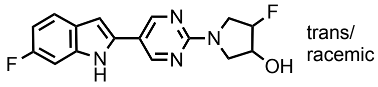

27

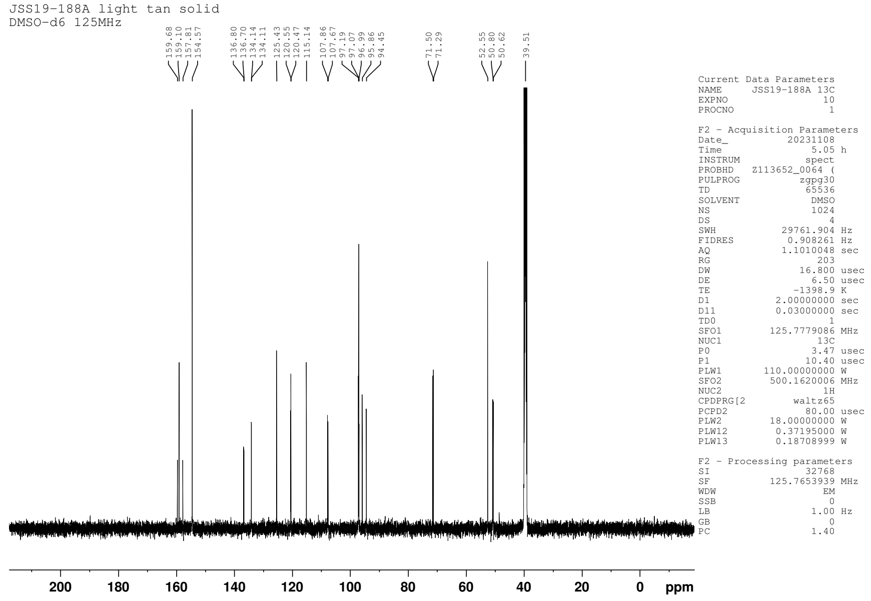

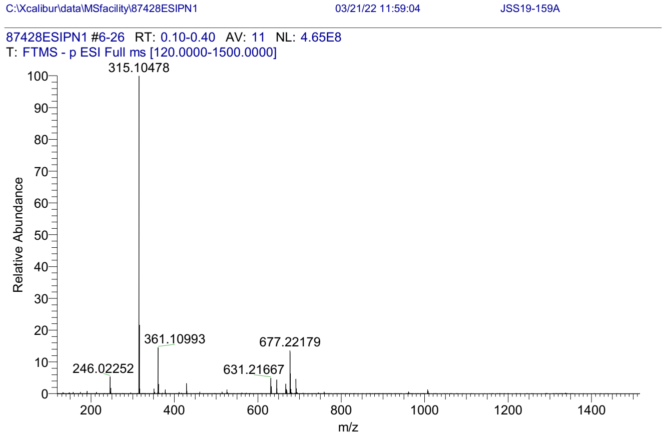

87428ESIPN1#6-26 RT: 0.10-0.40 AV: 11
T: FTMS - p ESI Full ms [120.0000-1500.0000]

m/z	Intensity	Relative	Theo. Mass	Delta (ppm)	Composition
315.10478	467651104.0	100.00	315.10519	-0.41	$C_{16}H_{13}ON_4F_2$

Figure S36. Compound **27** ^{1}H NMR spectrum (top), ^{13}C NMR spectrum (middle), and high-resolution mass spectrum (bottom).

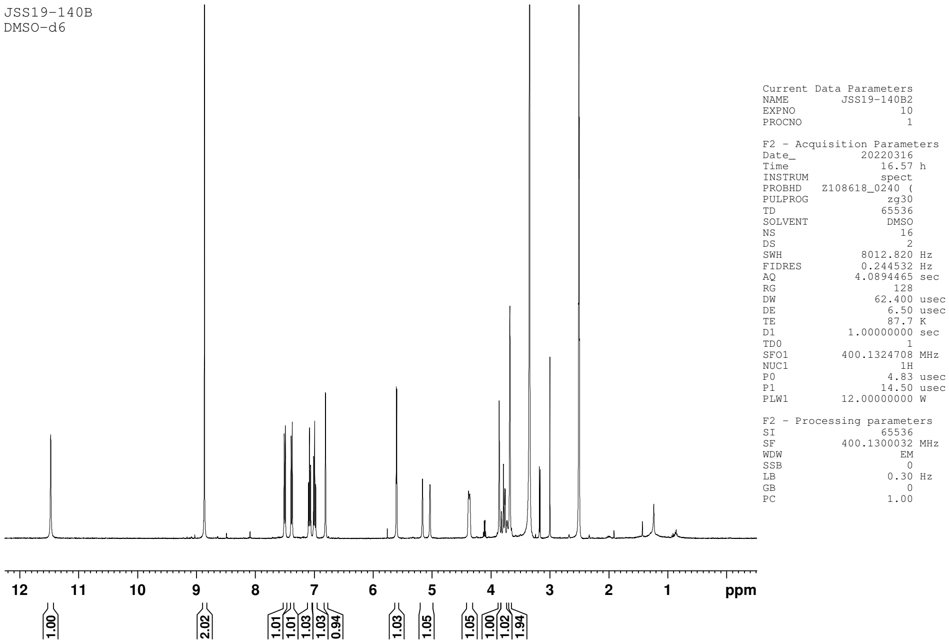

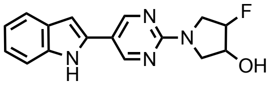

28

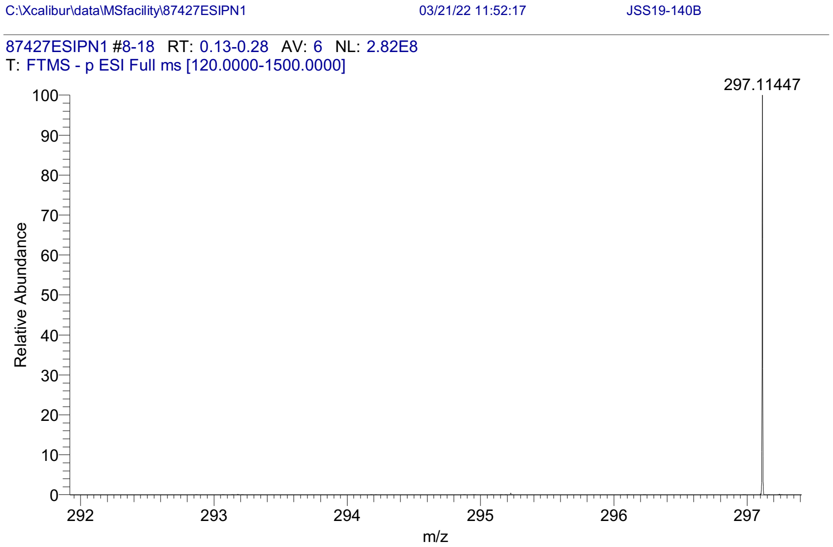

87427ESIPN1#8-18 RT: 0.13-0.28 AV: 6
T: FTMS - p ESI Full ms [120.0000-1500.0000]
m/z= 291.91784-297.40755

m/z	Intensity	Relative	Delta (ppm)	Composition
297.11447	292447040.0	100.00	-0.48	$C_{16}H_{14}ON_4F$

Figure S37. Compound **28** ^{1}H NMR spectrum (top) and high-resolution mass spectrum (bottom).

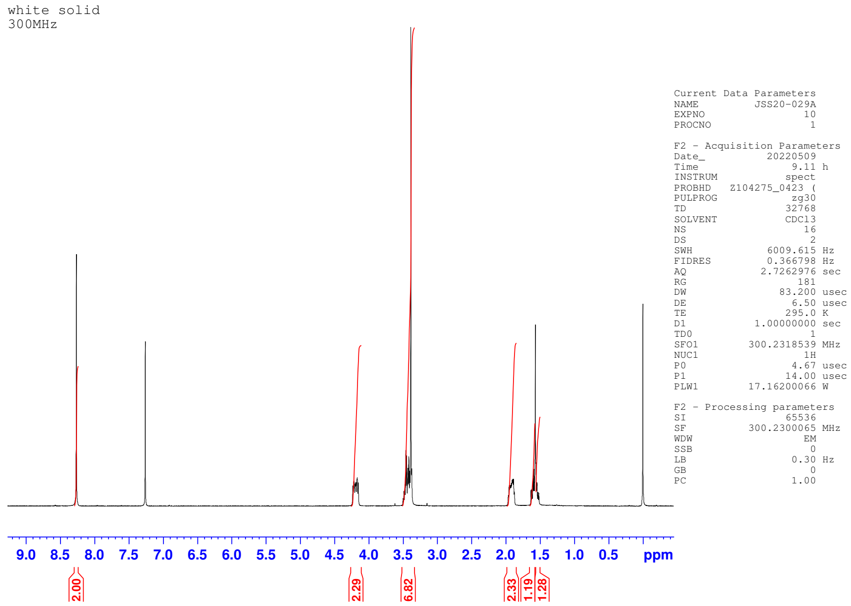

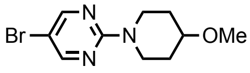

29

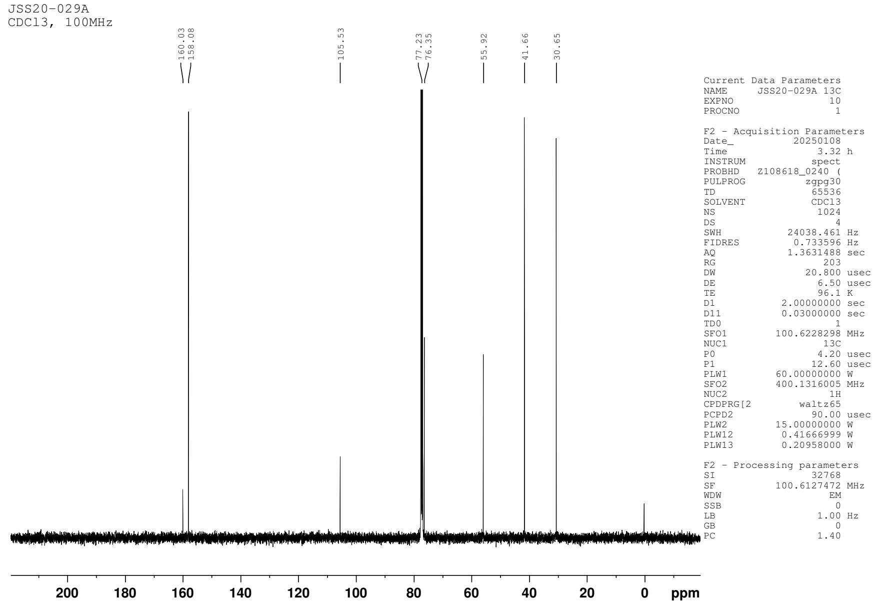

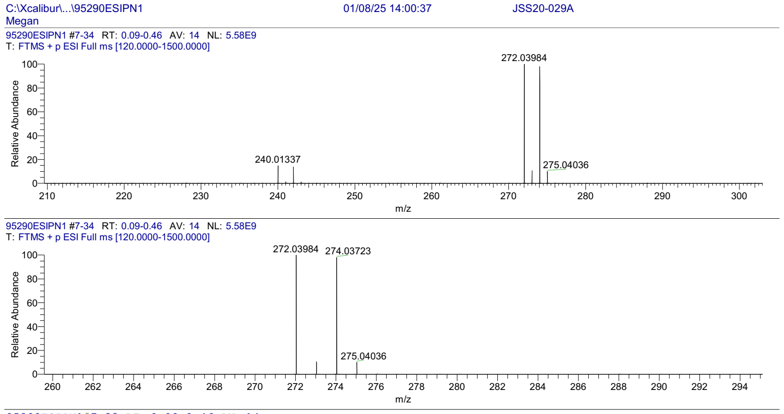

95290ESIPN1#7-33 RT: 0.09-0.46 AV: 14
T: FTMS + p ESI Full ms [120.0000-1500.0000]
m/z= 259.57150-295.10509

m/z	Intensity	Relative	Theo. Mass	Delta (ppm)	Composition
272.03984	5599183872.0	100.00	272.03930	2.00	$C_{10}H_{15}ON_3Br$

Figure S38. Compound **29** ^{1}H NMR spectrum (top), ^{13}C NMR spectrum (middle), high-resolution mass spectrum (bottom).

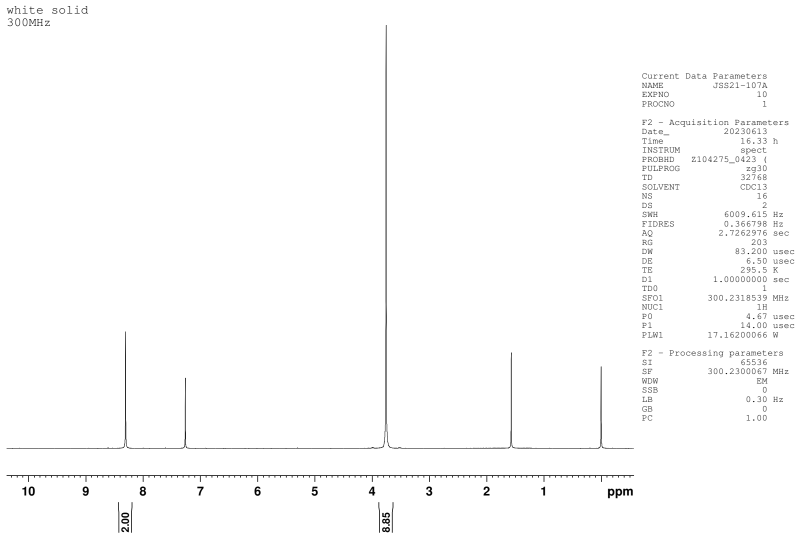

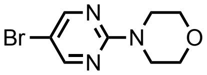

30

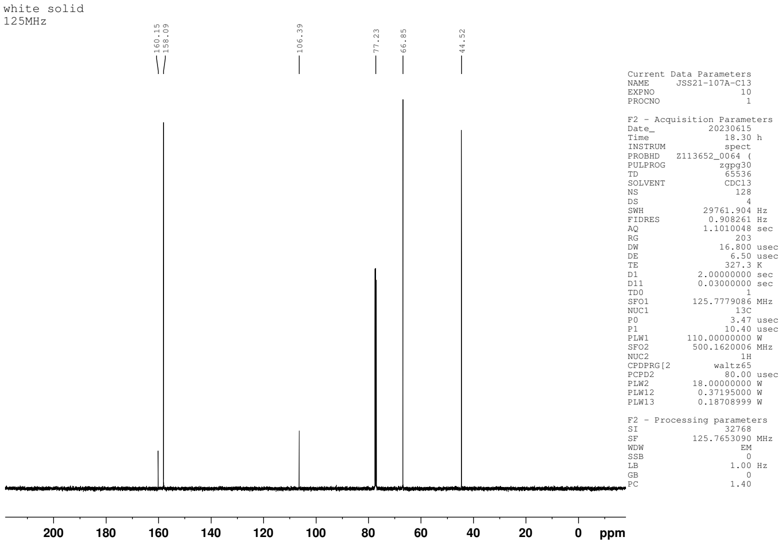

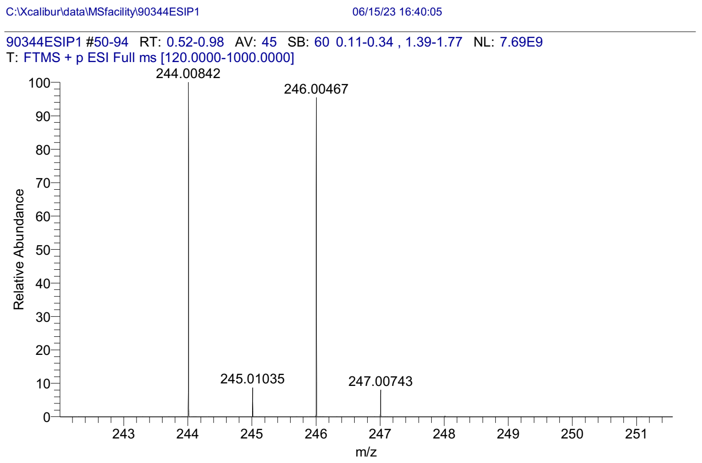

90344ESIP1#50-94 RT: 0.52-0.98 AV: 45
SB: 60 0.11-0.34 , 1.39-1.77
T: FTMS + p ESI Full ms [120.0000-1000.0000]
m/z= 242.00217-251.56310

m/z	Intensity	Relative	Theo. Mass	Delta (ppm)	Composition
244.00842	7738014208.0	100.00	244.00800	1.72	$C_8H_{11}ON_3Br$

Figure S39. Compound **30** ^{1}H NMR spectrum (top), ^{13}C NMR spectrum (middle), high-resolution mass spectrum (bottom).

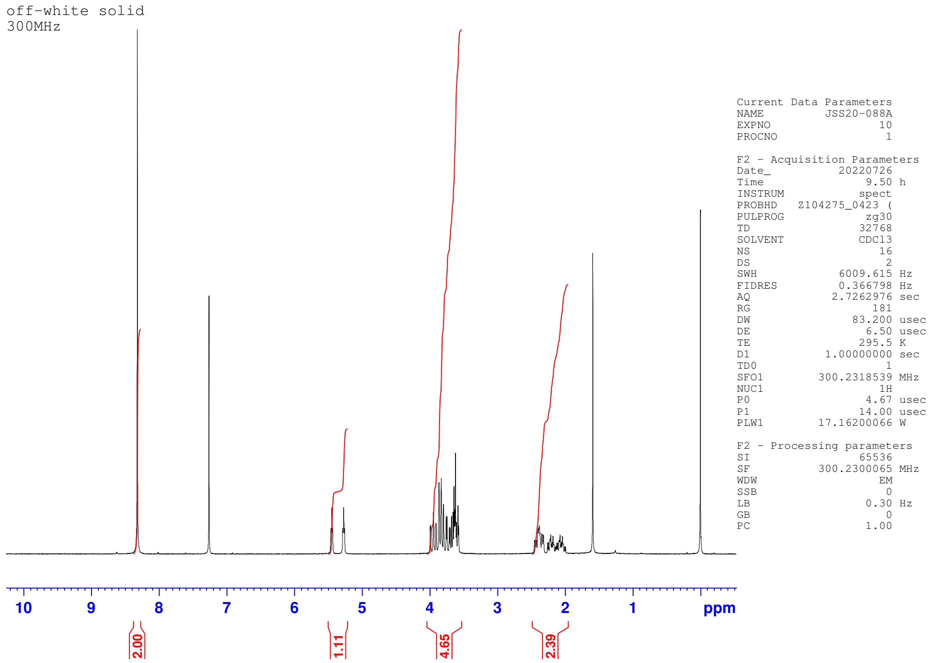

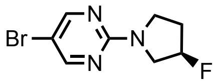

(R)-**31**

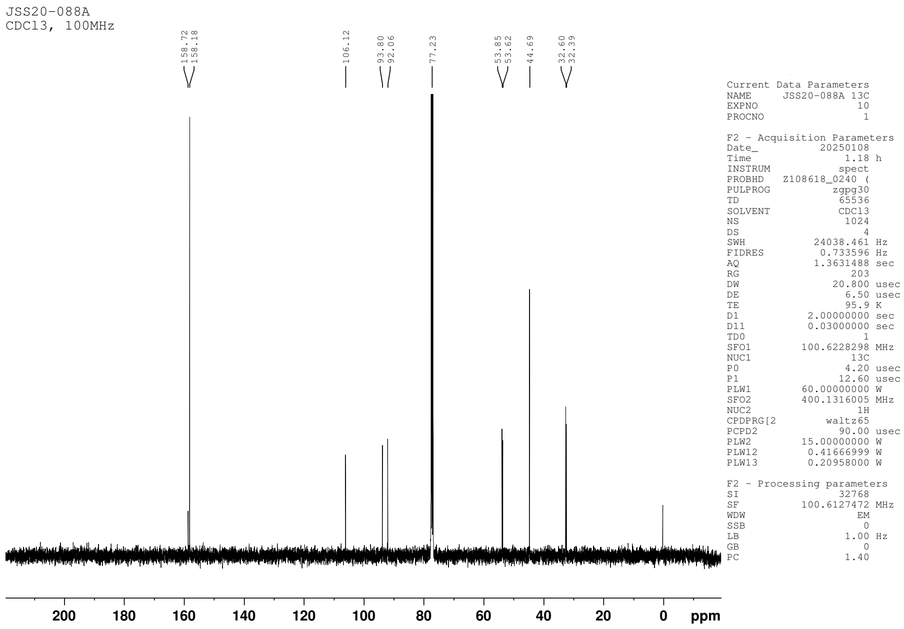

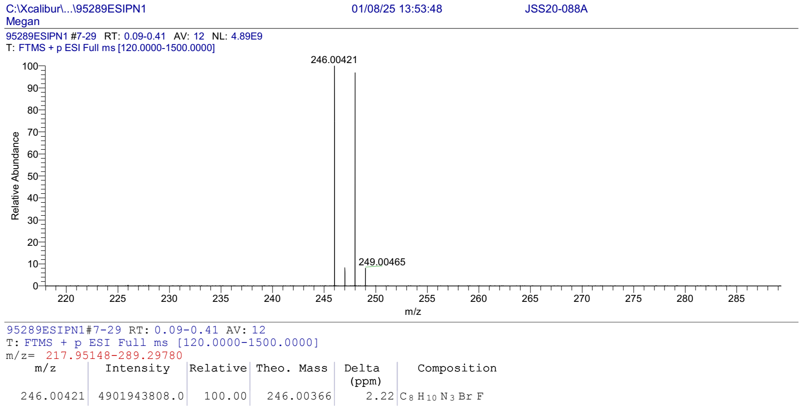

Figure S40. Compound **(*R*)-31** ^{1}H NMR spectrum (top), ^{13}C NMR spectrum (middle), high-resolution mass spectrum (bottom).

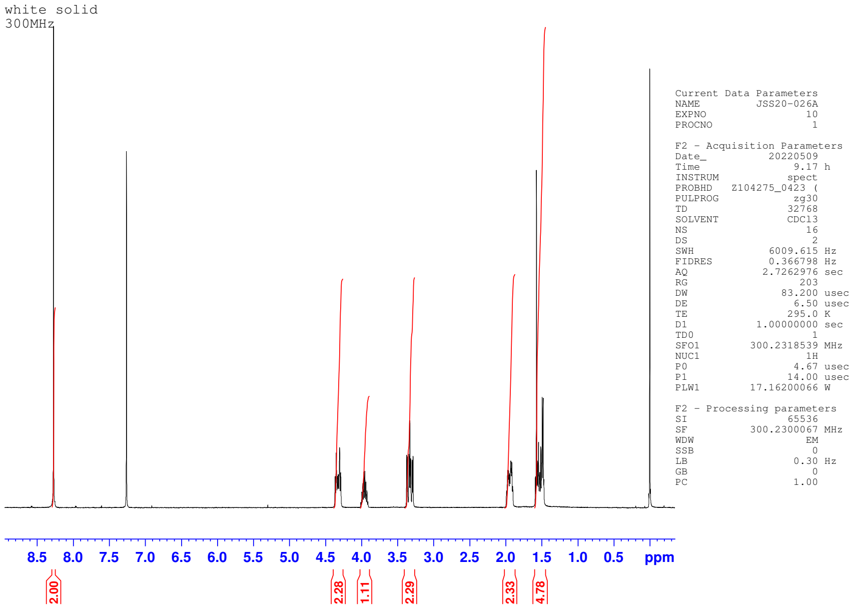

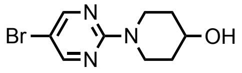

32

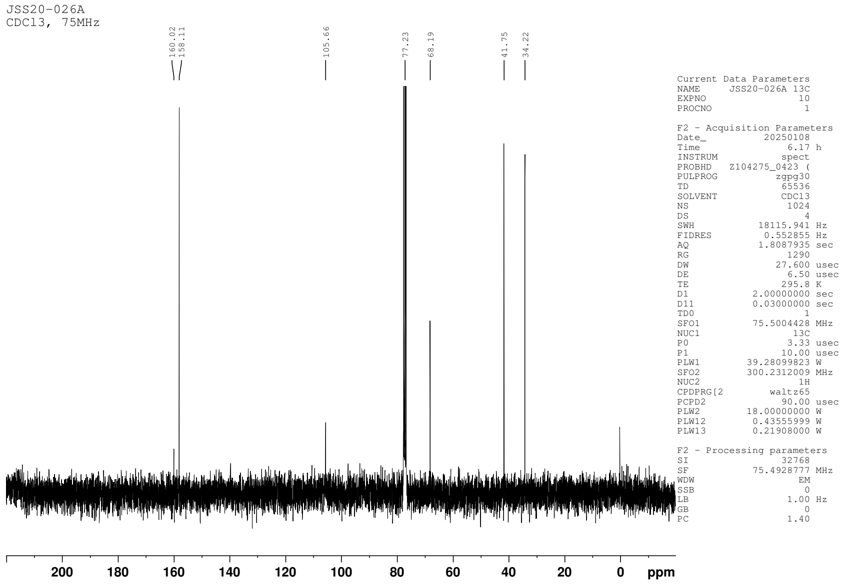

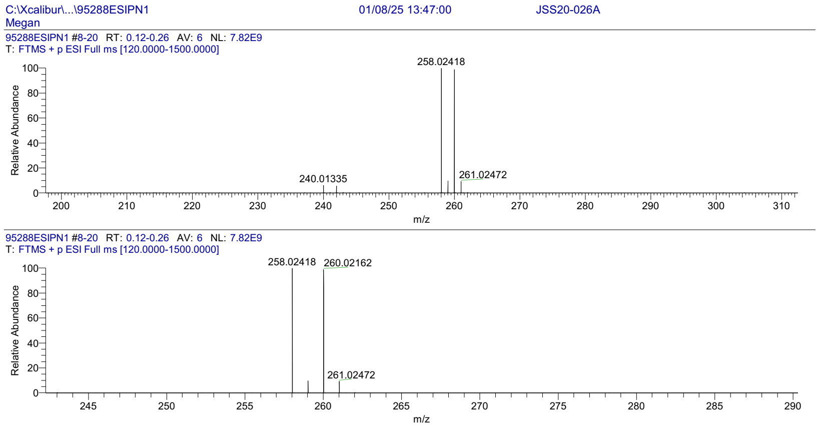

95288ESIPN1#9-19 RT: 0.12-0.26 AV: 6
T: FTMS + p ESI Full ms [120.0000-1500.0000]
m/z= 242.26525-290.30277

m/z	Intensity	Relative	Theo. Mass	Delta (ppm)	Composition
258.02418	7934893056.0	100.00	258.02365	2.05	$C_9H_{13}ON_3Br$

Figure S41. Compound **32** ^{1}H NMR spectrum (top), ^{13}C NMR spectrum (middle), high-resolution mass spectrum (bottom).

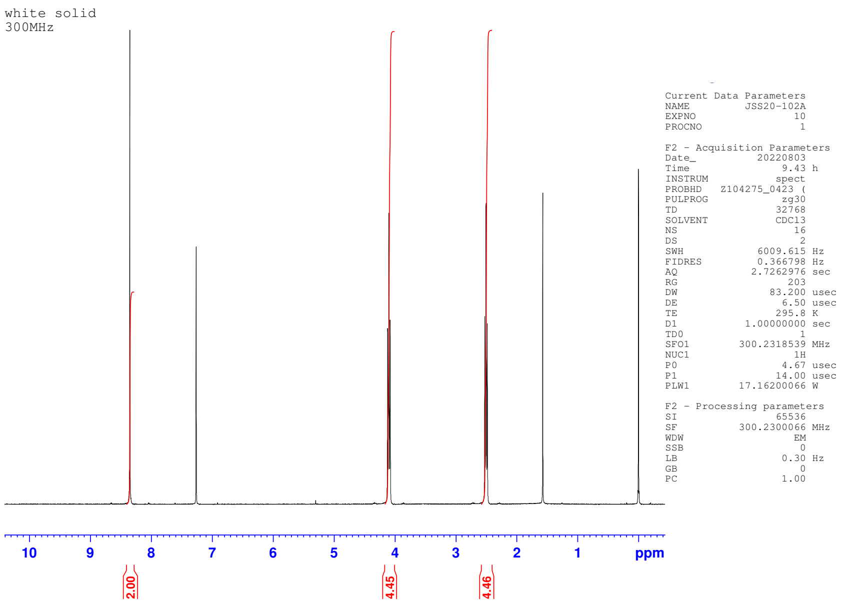

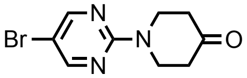

33

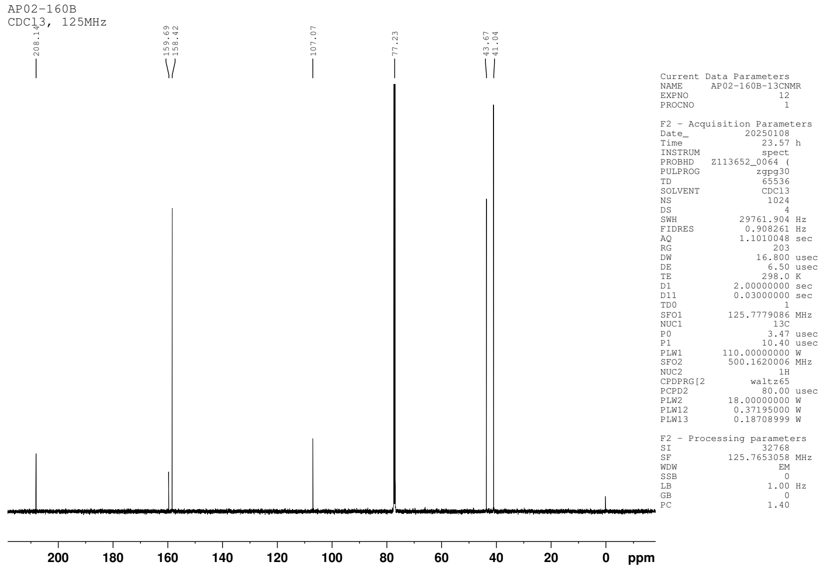

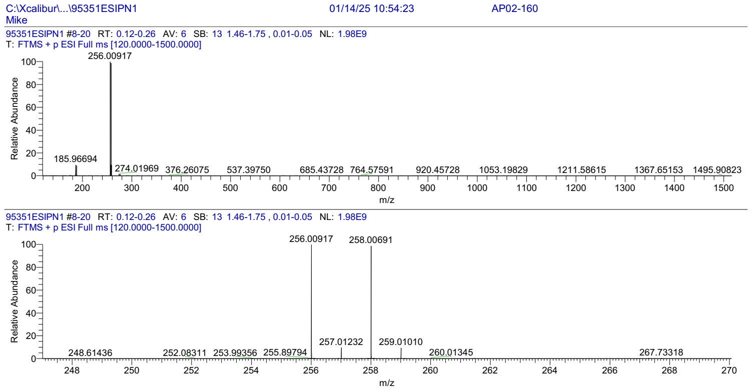

95351ESIPN1#9-19 RT: 0.12-0.26 AV: 6
SB: 13 1.46-1.75 , 0.01-0.05
T: FTMS + p ESI Full ms [120.0000-1500.0000]
m/z= 241.47375-285.74416

m/z	Intensity	Relative	Theo. Mass	Delta (ppm)	Composition
256.00917	2000862464.0	100.00	256.00800	4.56	$C_9 H_{11} O N_3 Br$

Figure S42. Compound **33** ^{1}H NMR spectrum (top), ^{13}C NMR spectrum (middle), high-resolution mass spectrum (bottom).

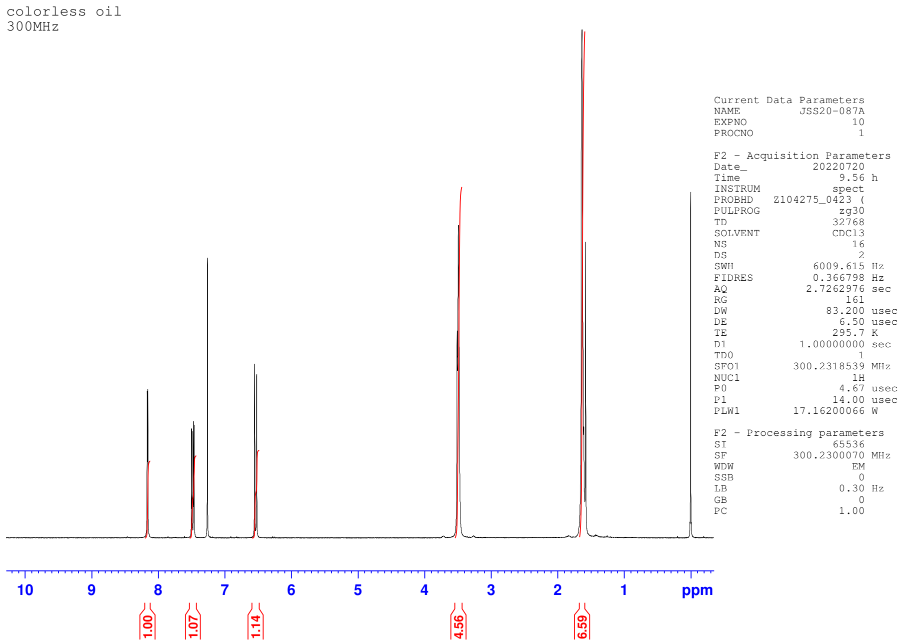

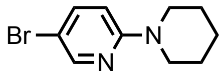

34

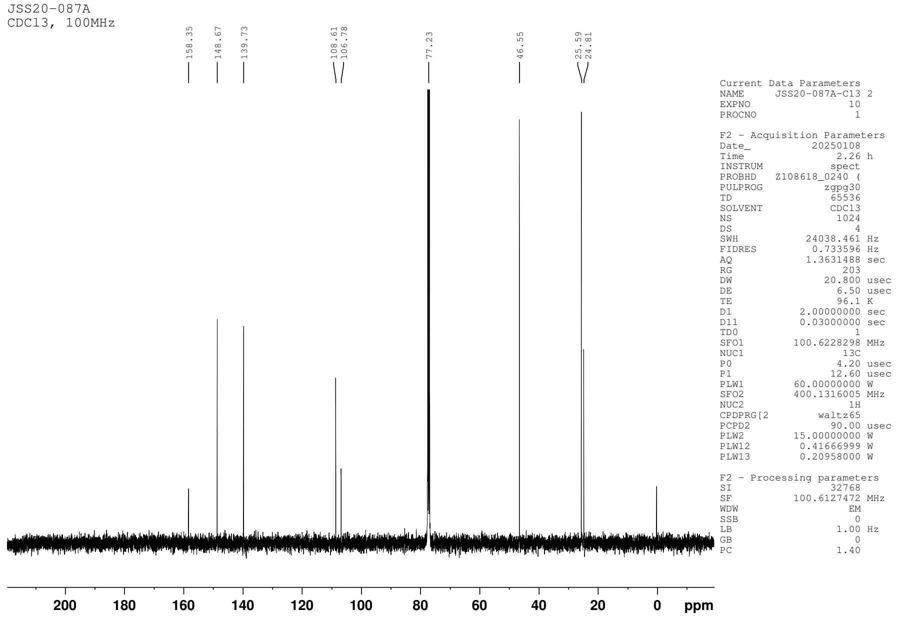

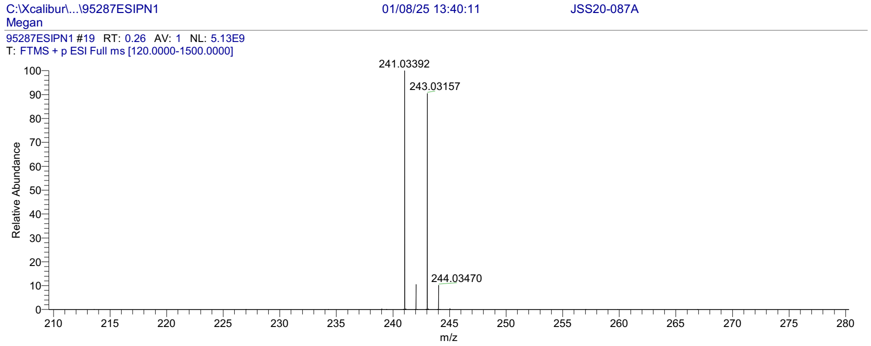

95287ESIPN1#19 RT: 0.26
T: FTMS + p ESI Full ms [120.0000-1500.0000]
m/z= 209.58236-280.29005

m/z	Intensity	Relative	Theo. Mass	Delta (ppm)	Composition
241.03392	5135907328.0	100.00	241.03349	1.80	$C_{10}H_{14}N_2Br$

Figure S43. Compound **34** ^{1}H NMR spectrum (top), ^{13}C NMR spectrum (middle), high-resolution mass spectrum (bottom).

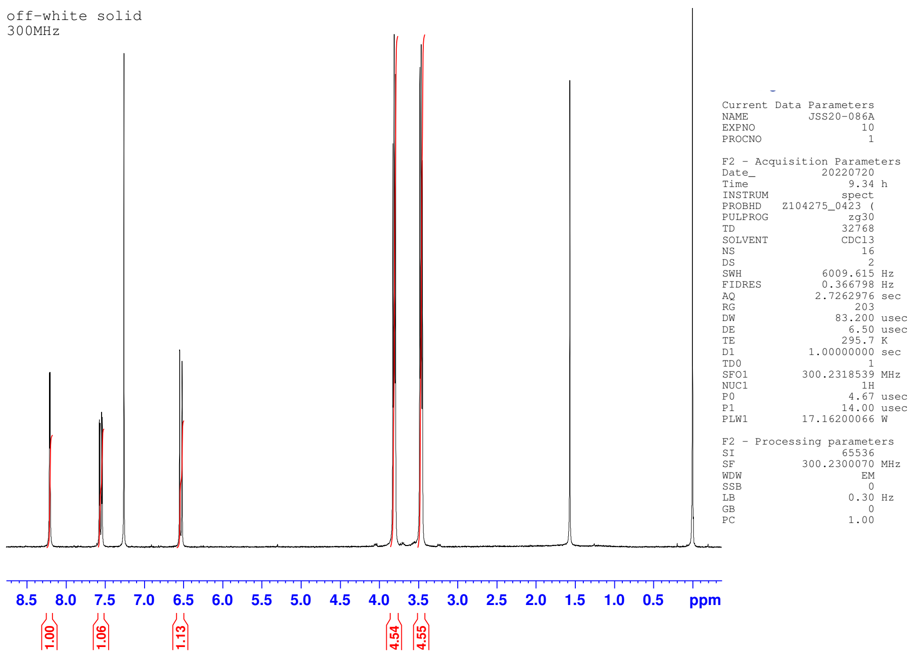

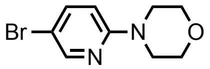

35

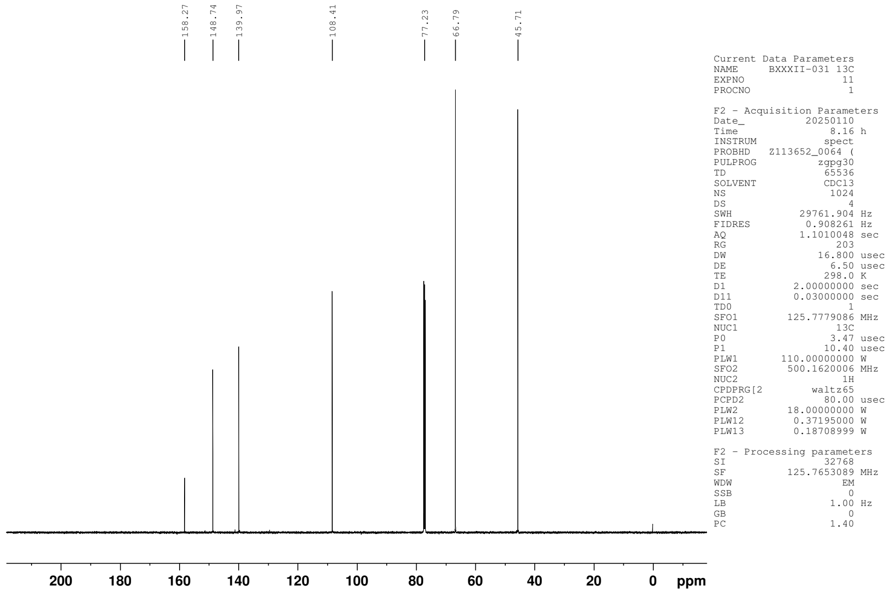

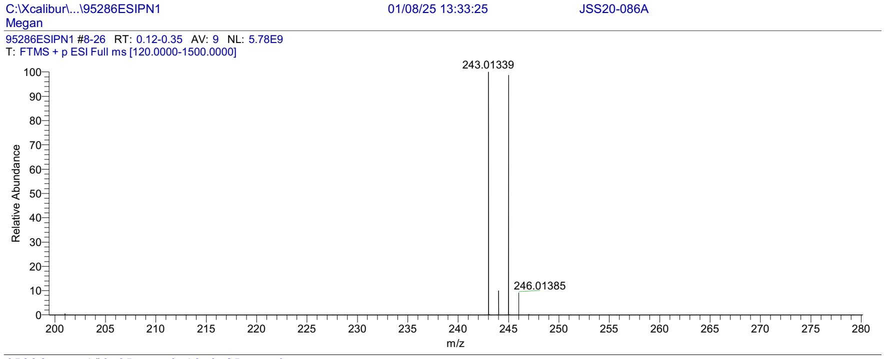

95286ESIPN1#9-25 RT: 0.12-0.35 AV: 9
T: FTMS + p ESI Full ms [120.0000-1500.0000]
m/z= 199.39356-280.11456

m/z	Intensity	Relative	Theo. Mass	Delta (ppm)	Composition
243.01339	5895931904.0	100.00	243.01275	2.64	$C_9H_{12}ON_2Br$

Figure S44. Compound **35** ^{1}H NMR spectrum (top), ^{13}C NMR spectrum (middle), high-resolution mass spectrum (bottom).

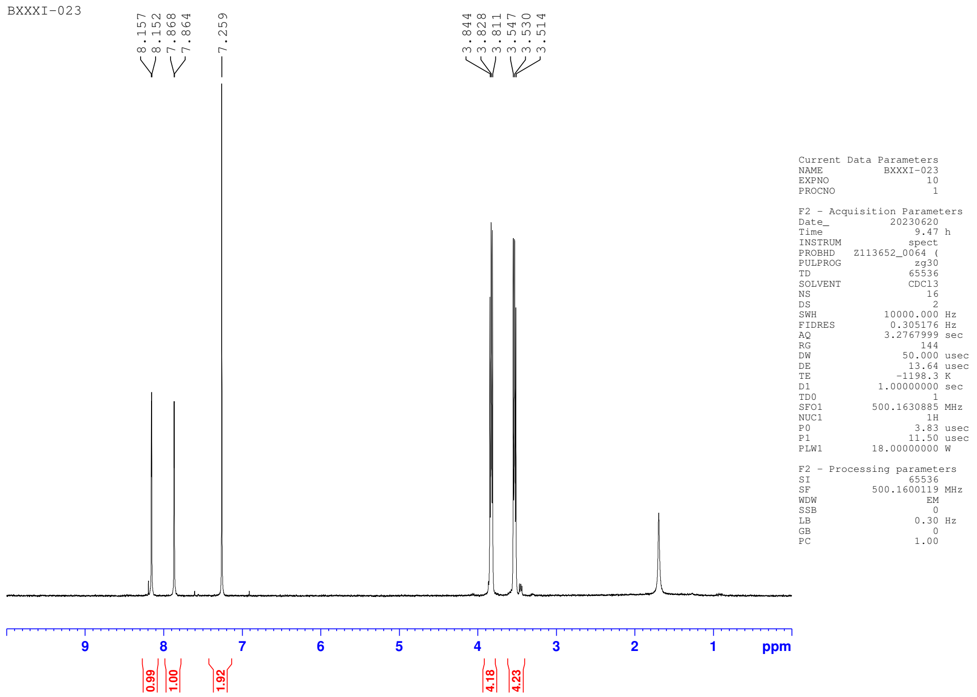

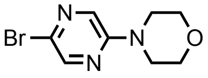

36

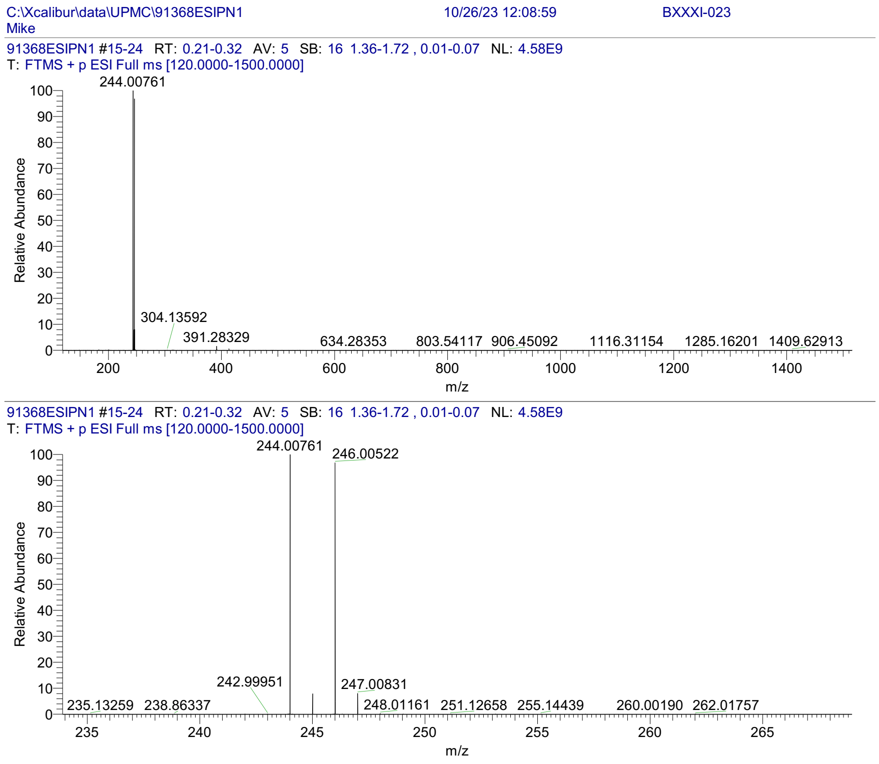

91368ESIPN1#15-23 RT: 0.21-0.32 AV: 5
SB: 16 1.36-1.72 , 0.01-0.07
T: FTMS + p ESI Full ms [120.0000-1500.0000]
m/z= 233.89190-268.96538

m/z	Intensity	Relative	Theo. Mass	Delta (ppm)	Composition
244.00761	4591328256.0	100.00	244.00800	-1.58	$C_8H_{11}ON_3Br$

Figure S45. Compound **36** ^{1}H NMR spectrum (top) and high-resolution mass spectrum (bottom).

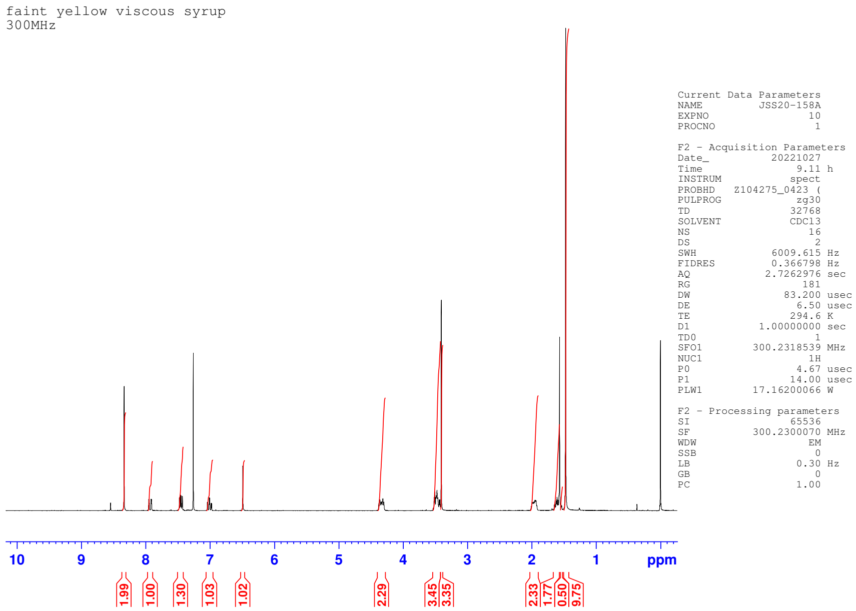

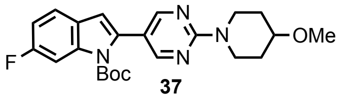

37

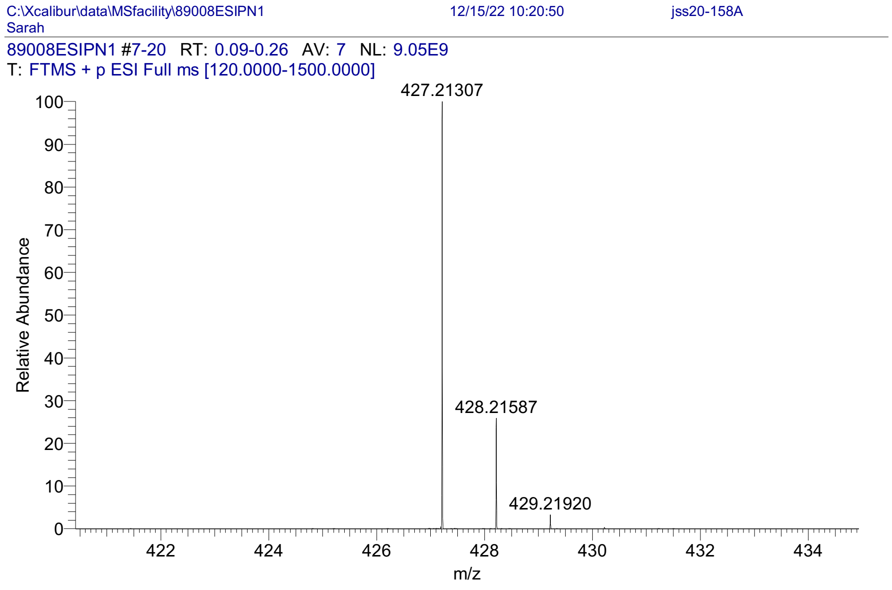

89008ESIPN1#7-19 RT: 0.09-0.26 AV: 7
T: FTMS + p ESI Full ms [120.0000-1500.0000]
m/z= 420.41587-434.93539

m/z	Intensity	Relative	Theo. Mass	Delta (ppm)	Composition
427.21307	9255216128.0	100.00	427.21400	-2.16	$C_{23}H_{28}O_3N_4F$

Figure S46. Compound **37** ^{1}H NMR spectrum (top) and high-resolution mass spectrum (bottom).

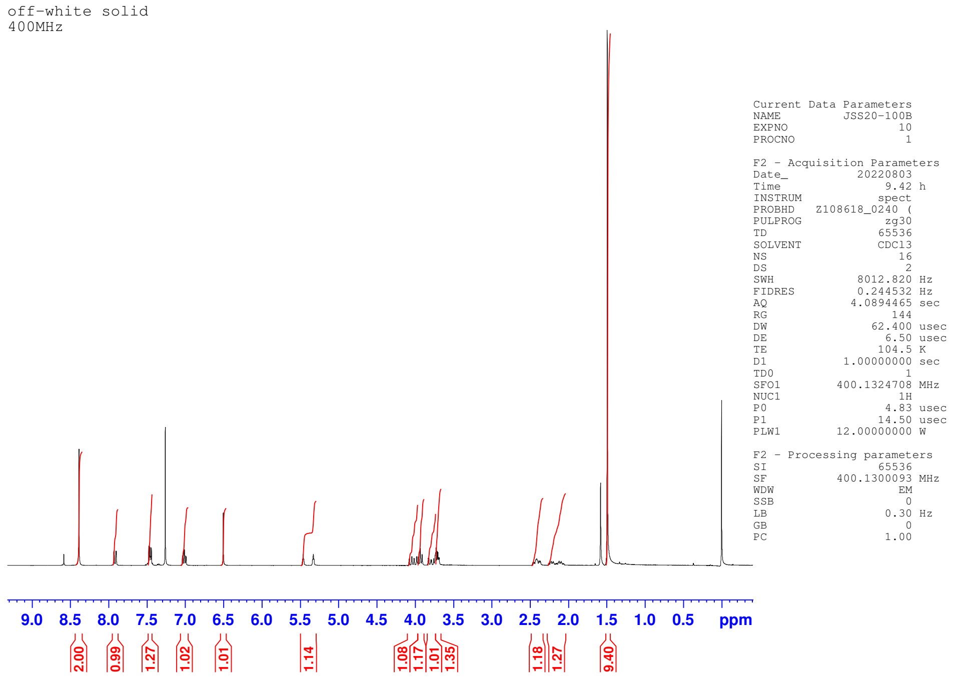

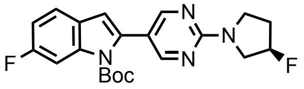

(*R*)-**38**

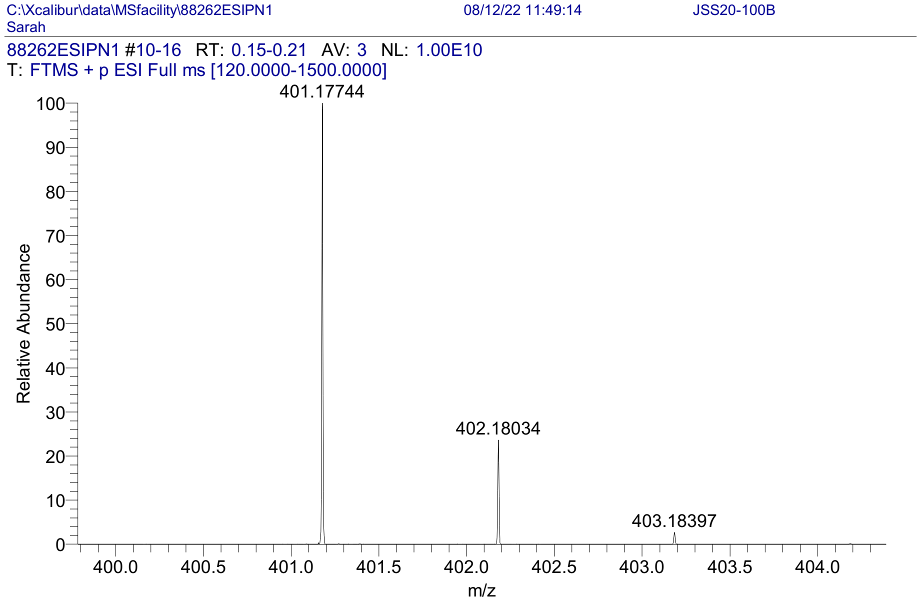

88262ESIPN1#11-15 RT: 0.15-0.21 AV: 3
T: FTMS + p ESI Full ms [120.0000-1500.0000]
m/z= 399.78301-404.38922

m/z	Intensity	Relative	Theo. Mass	Delta (ppm)	Composition
401.17744	10368265216.0	100.00	401.17836	-2.29	$C_{21}H_{23}O_2N_4F_2$

Figure S47. Compound **(*R*)-38** ^{1}H NMR spectrum (top) and high-resolution mass spectrum (bottom).

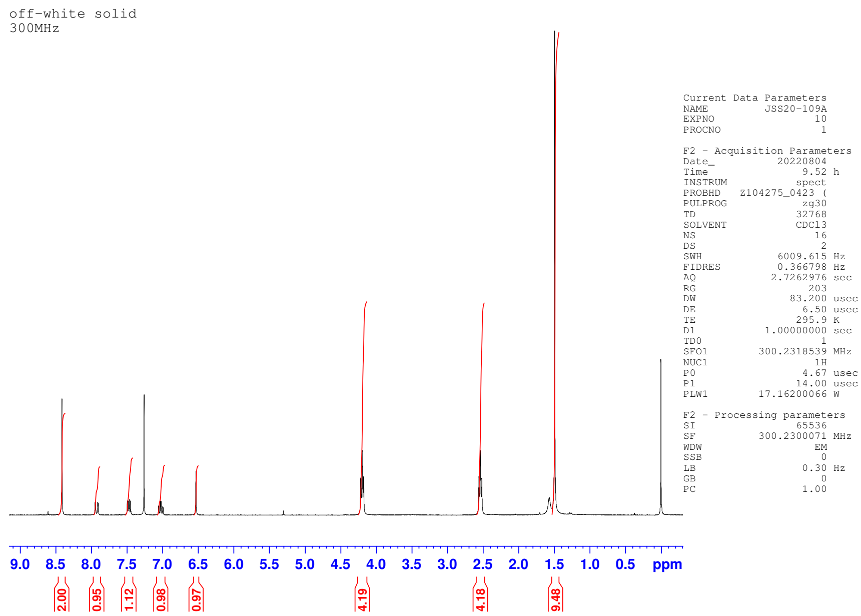

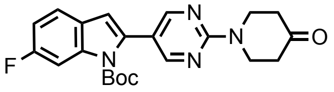

39

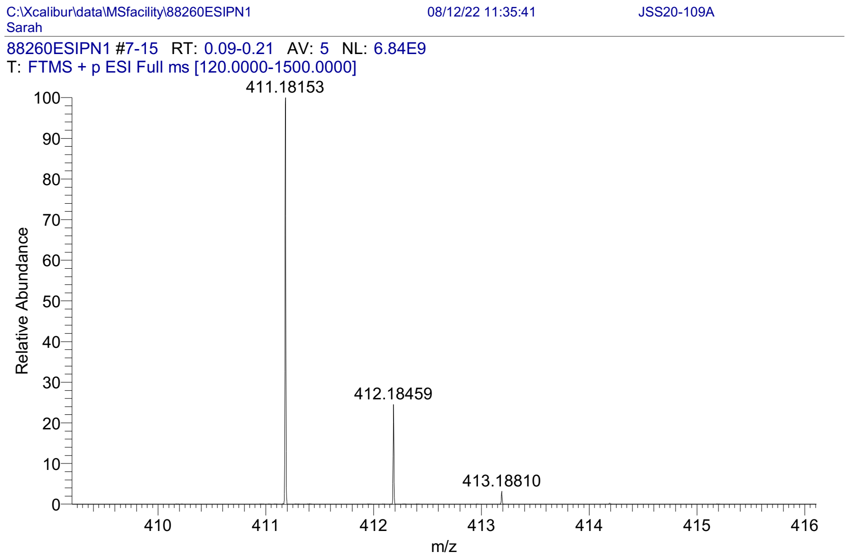

88260ESIPN1#7-15 RT: 0.09-0.21 AV: 5
T: FTMS + p ESI Full ms [120.0000-1500.0000]
m/z= 399.11632-424.53643

m/z	Intensity	Relative	Theo. Mass	Delta (ppm)	Composition
411.18153	7059740160.0	100.00	411.18270	-2.84	$C_{22}H_{24}O_3N_4F$

Figure S48. Compound **39** ^{1}H NMR spectrum (top) and high-resolution mass spectrum (bottom).

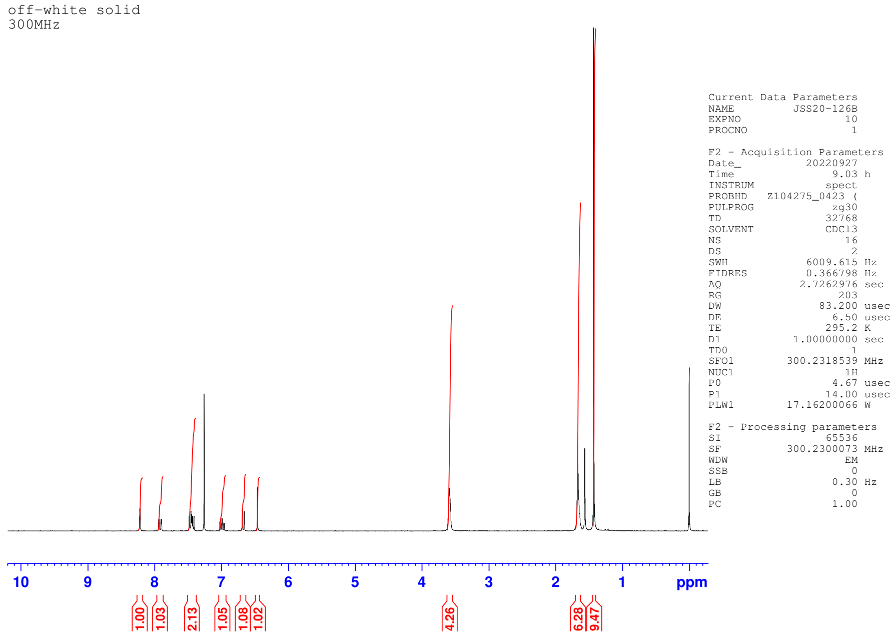

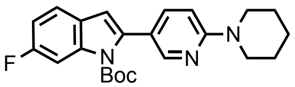

40

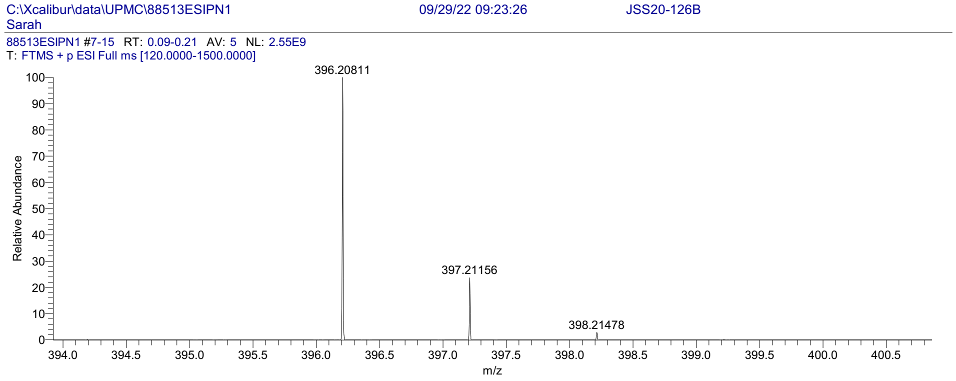

88513ESIPN1#7-15 RT: 0.09-0.21 AV: 5
T: FTMS + p ESI Full ms [120.0000-1500.0000]
m/z= 393.92201-400.85905

m/z	Intensity	Relative	Theo. Mass	Delta (ppm)	Composition
396.20811	2567506688.0	100.00	396.20818	-0.19	$C_{23}H_{27}O_2N_3F$

Figure S49. Compound **40** ^{1}H NMR spectrum (top) and high-resolution mass spectrum (bottom).

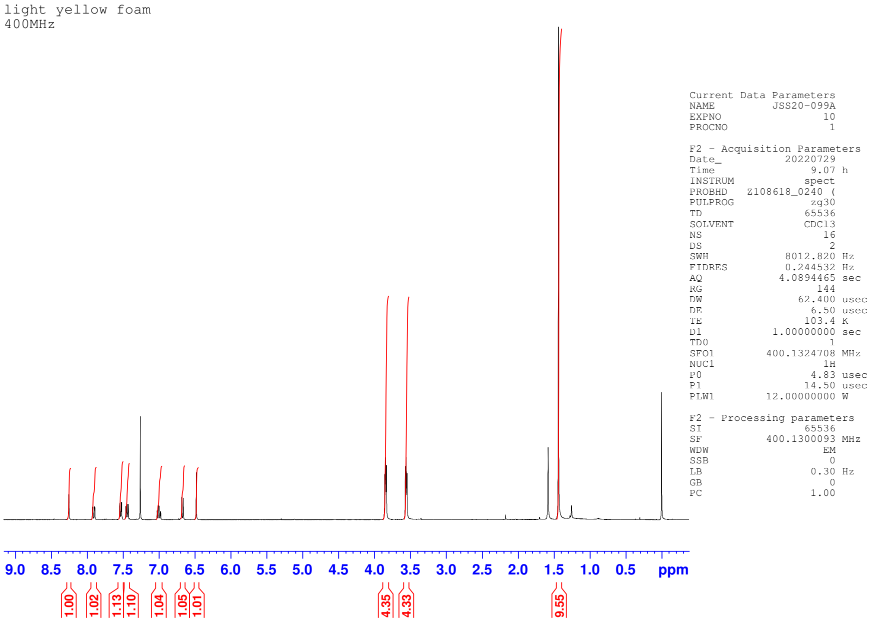

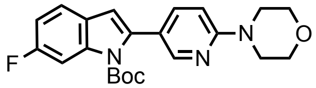

41

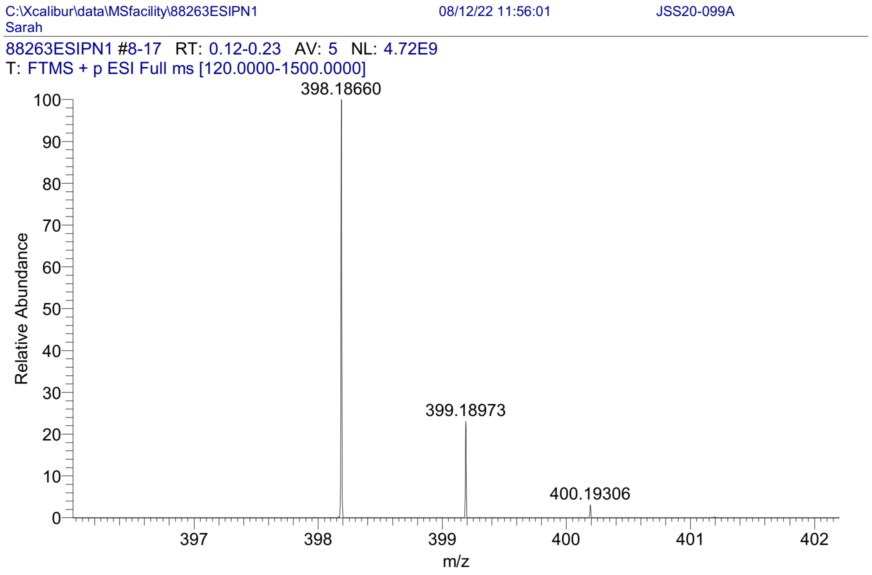

88263ESIPN1#9-17 RT: 0.12-0.23 AV: 5
T: FTMS + p ESI Full ms [120.0000-1500.0000]
m/z= 396.02327-402.19912

m/z	Intensity	Relative	Theo. Mass	Delta (ppm)	Composition
398.18660	4752931840.0	100.00	398.18745	-2.12	$C_{22}H_{25}O_3N_3F$

Figure S50. Compound **41** ^{1}H NMR spectrum (top), ^{13}C NMR spectrum (middle), and high-resolution mass spectrum (bottom).

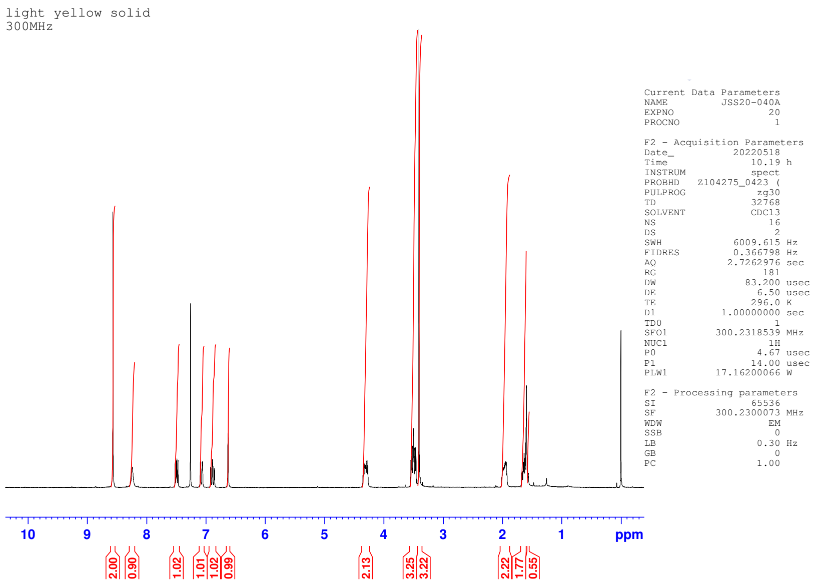

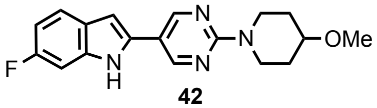

42

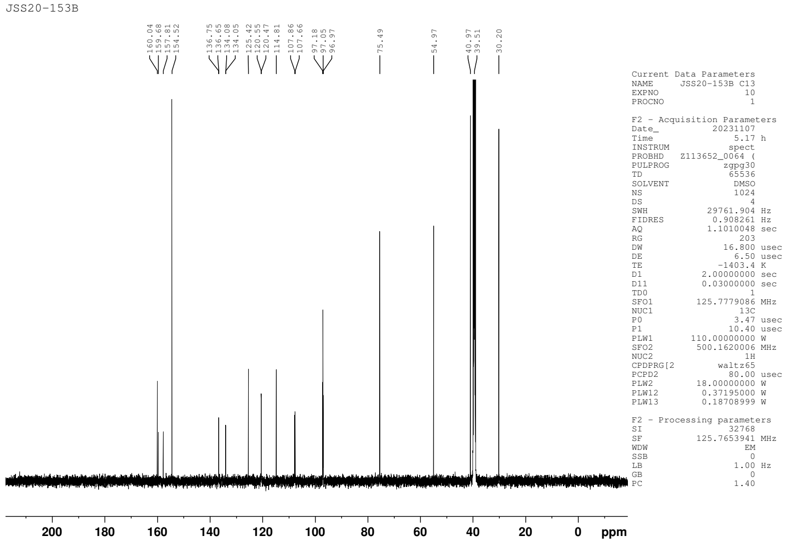

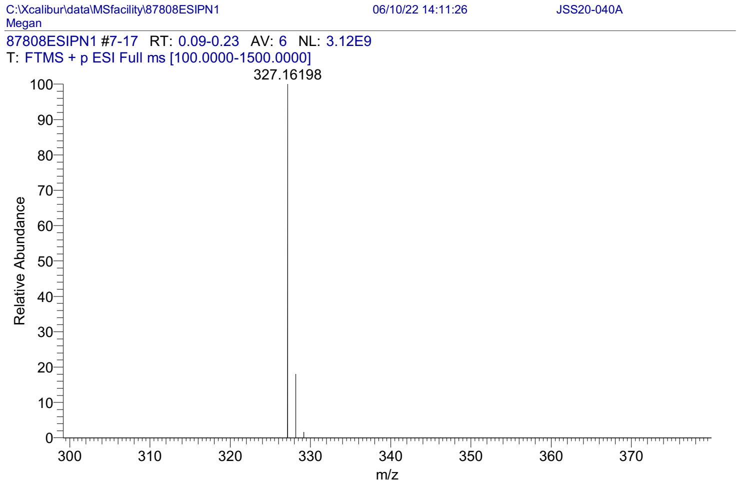

87808ESIPN1#7-17 RT: 0.09-0.23 AV: 6
T: FTMS + p ESI Full ms [100.0000-1500.0000]
m/z= 299.15896-379.95799

m/z	Intensity	Relative	Theo. Mass	Delta (ppm)	Composition
327.16198	3148229120.0	100.00	327.16157	1.26	$C_{18}H_{20}ON_4F$

Figure S51. Compound **42** ^{1}H NMR spectrum (top), ^{13}C NMR spectrum (middle), and high-resolution mass spectrum (bottom).

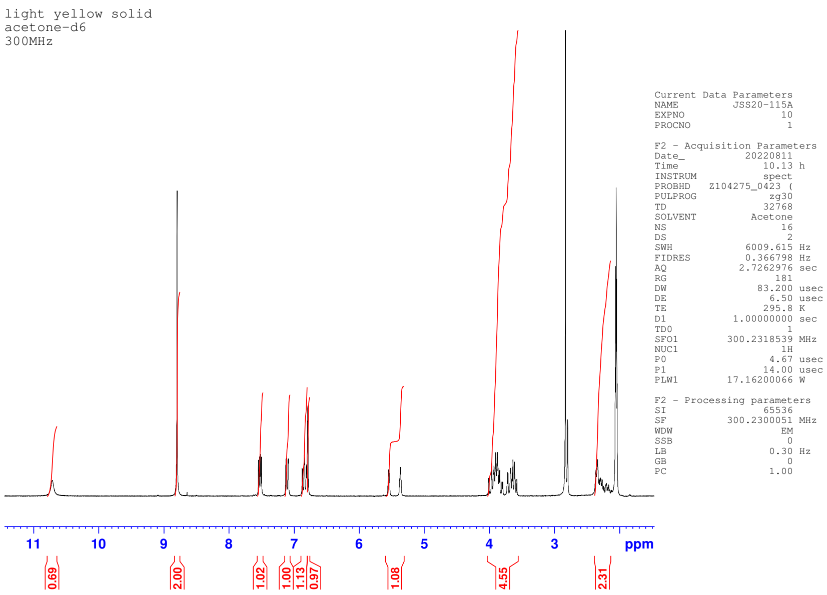

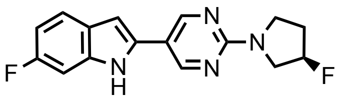

(*R*)-**43**

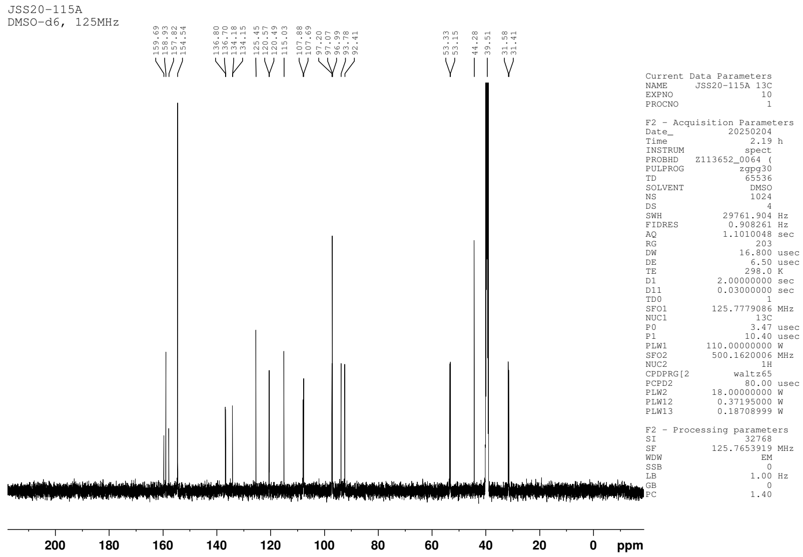

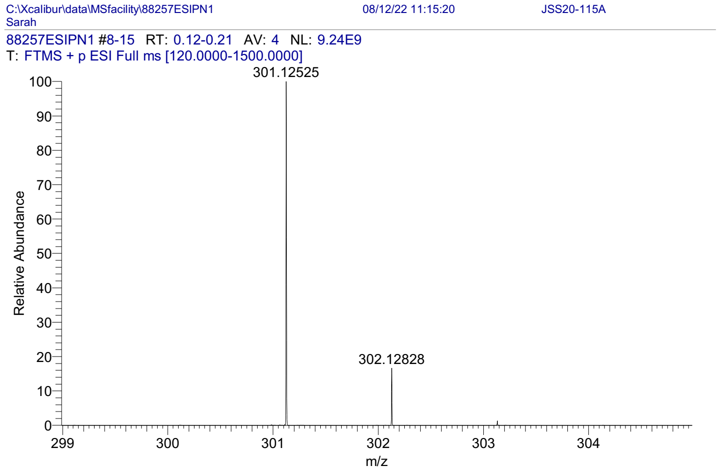

88257ESIPN1#9-15 RT: 0.12-0.21 AV: 4
T: FTMS + p ESI Full ms [120.0000-1500.0000]
m/z= 298.98890-304.98208

m/z	Intensity	Relative	Theo. Mass	Delta (ppm)	Composition
301.12525	9302198272.0	100.00	301.12593	-2.25	$C_{16}H_{15}N_4F_2$

Figure S52. Compound **(*R*)-43** ^{1}H NMR spectrum (top), ^{13}C NMR spectrum (middle), and high-resolution mass spectrum (bottom).

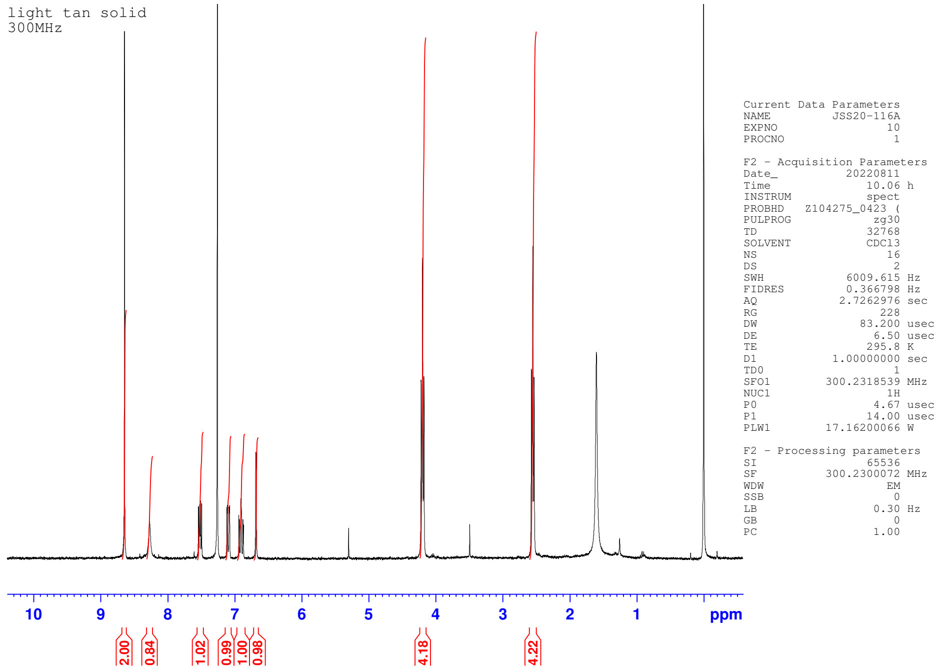

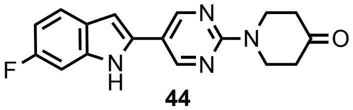

44

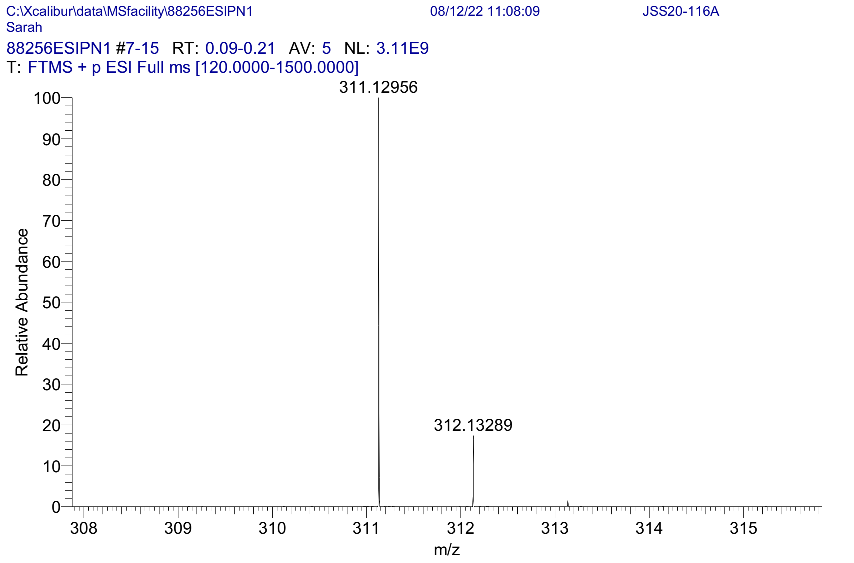

88256ESIPN1#7-15 RT: 0.09-0.21 AV: 5
T: FTMS + p ESI Full ms [120.0000-1500.0000]
m/z= 307.87563-315.83032

m/z	Intensity	Relative	Theo. Mass	Delta (ppm)	Composition
311.12956	3116225280.0	100.00	311.13027	-2.27	$C_{17}H_{16}ON_4F$

Figure S53. Compound **44** ^{1}H NMR spectrum (top) and high-resolution mass spectrum (bottom).

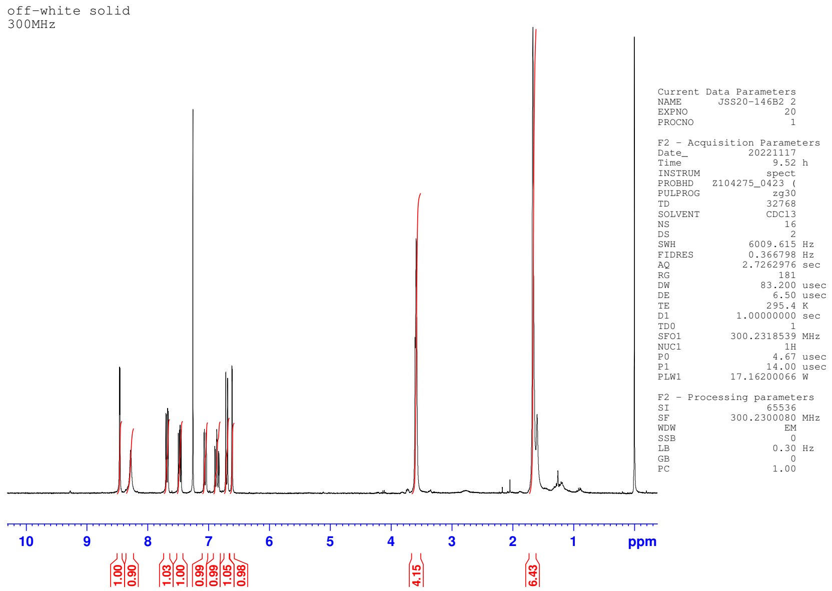

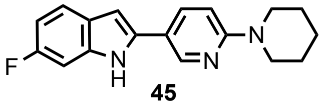

45

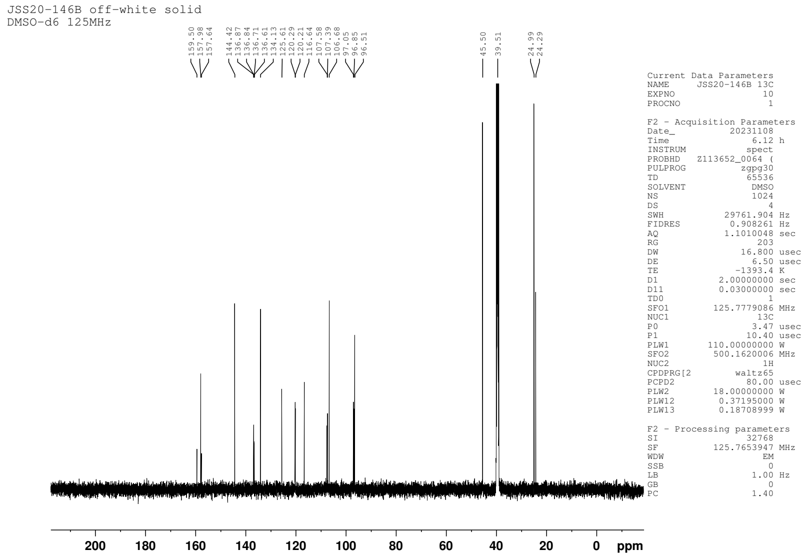

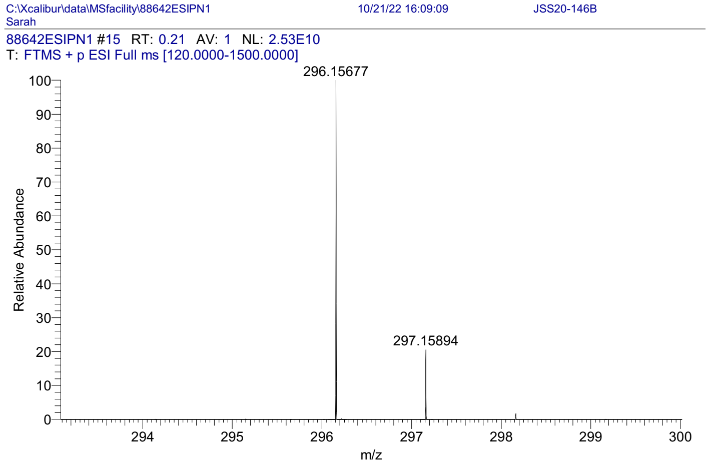

88642ESIPN1#15 RT: 0.21
T: FTMS + p ESI Full ms [120.0000-1500.0000]
m/z= 293.08446-300.01674

m/z	Intensity	Relative	Theo. Mass	Delta (ppm)	Composition
296.15677	26530134016.0	100.00	296.15575	3.43	$C_{18}H_{19}N_3F$

Figure S54. Compound **45** ^{1}H NMR spectrum (top), ^{13}C NMR spectrum (middle), and high-resolution mass spectrum (bottom).

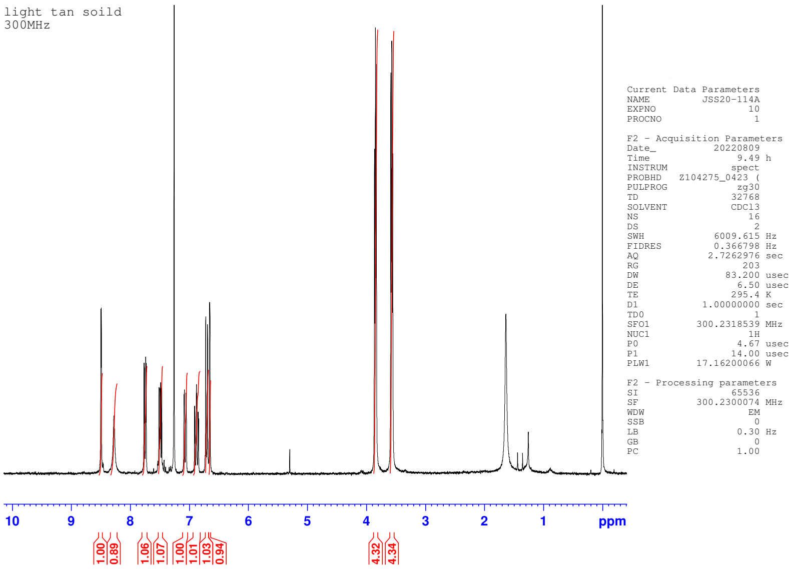

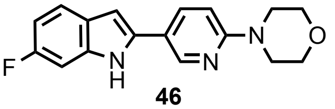

46

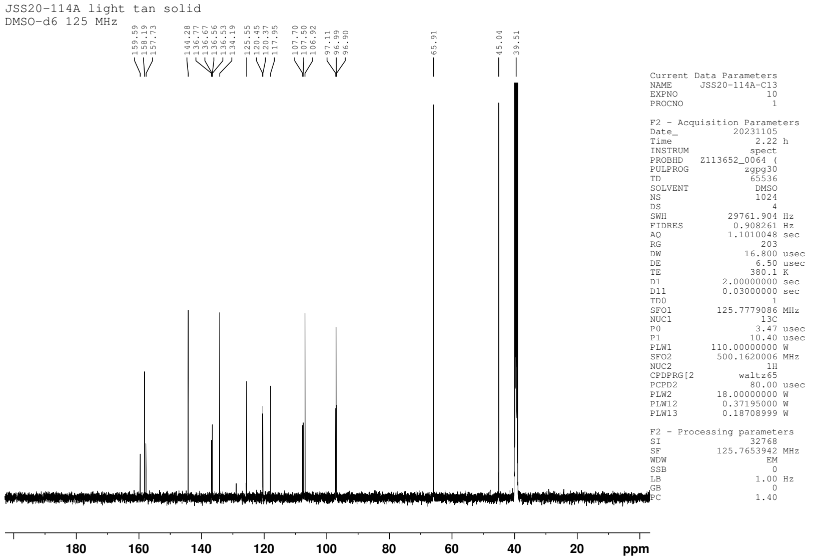

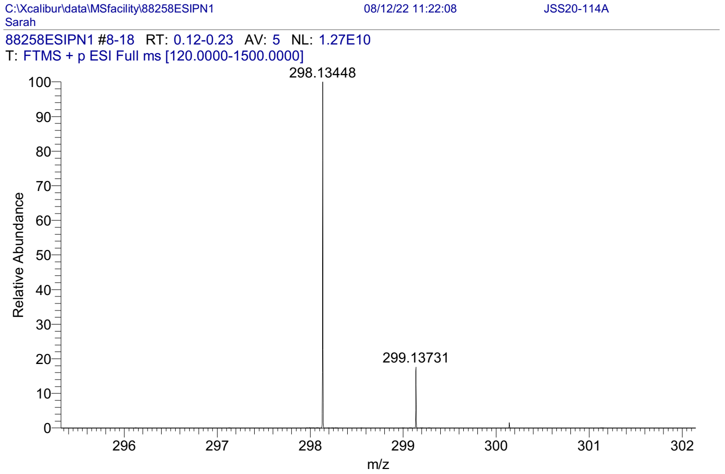

88258ESIPN1#9-17 RT: 0.12-0.23 AV: 5
T: FTMS + p ESI Full ms [120.0000-1500.0000]
m/z= 295.31817-302.14466

m/z	Intensity	Relative	Theo. Mass	Delta (ppm)	Composition
298.13448	12686499840.0	100.00	298.13502	-1.78	$C_{17}H_{17}ON_3F$

Figure S55. Compound **46** ^{1}H NMR spectrum (top), ^{13}C NMR spectrum (middle), and high-resolution mass spectrum (bottom).

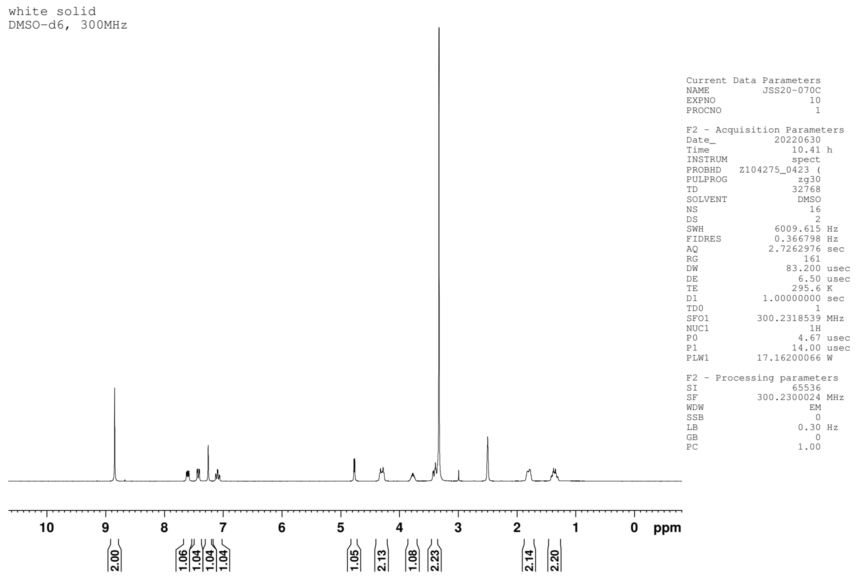

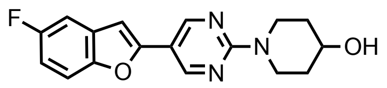

49

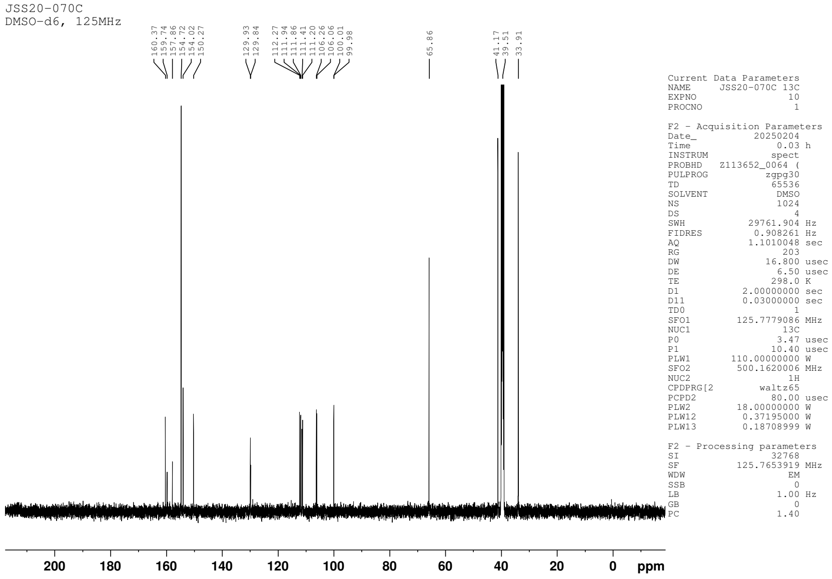

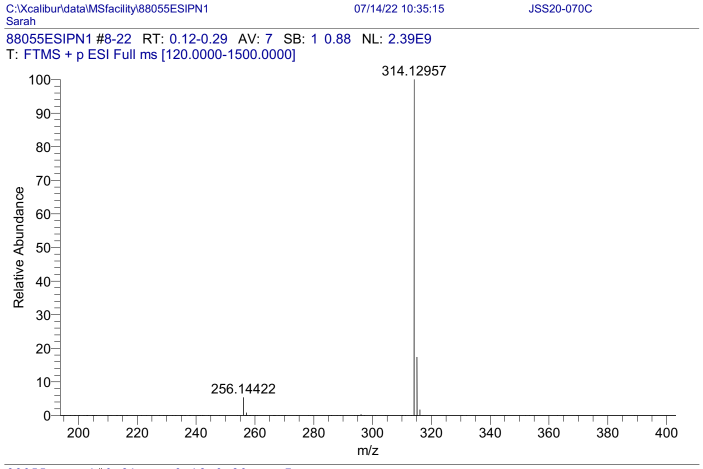

88055ESIPN1#9-21 RT: 0.12-0.29 AV: 7
SB: 1 0.88
T: FTMS + p ESI Full ms [120.0000-1500.0000]
m/z= 196.55885-403.14587

m/z	Intensity	Relative	Theo. Mass	Delta (ppm)	Composition
314.12957	2403647232.0	100.00	314.12993	-1.16	$C_{17}H_{17}O_2N_3F$

Figure S56. Compound **49** ^{1}H NMR spectrum (top), ^{13}C NMR spectrum (middle), and high-resolution mass spectrum (bottom).

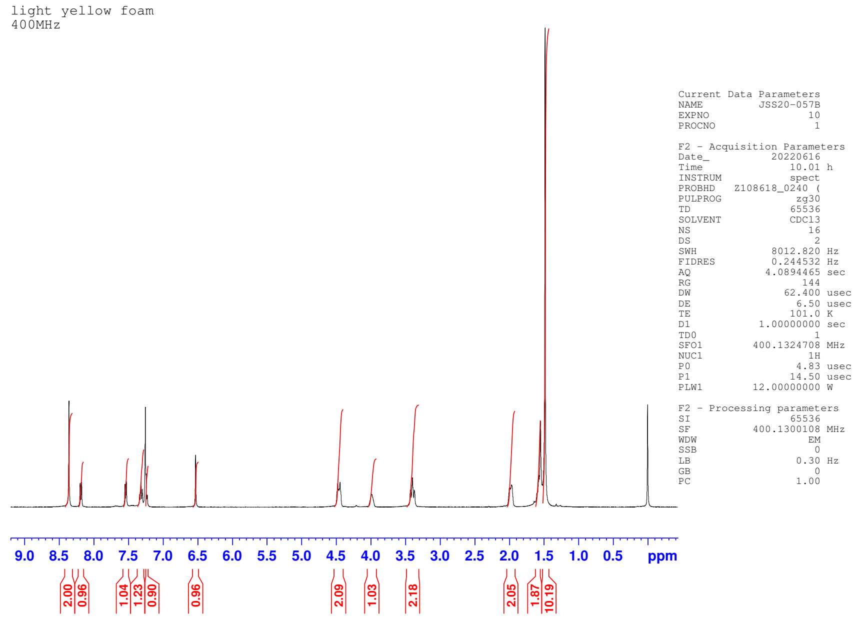

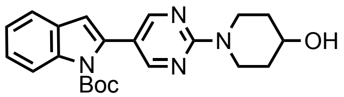

50

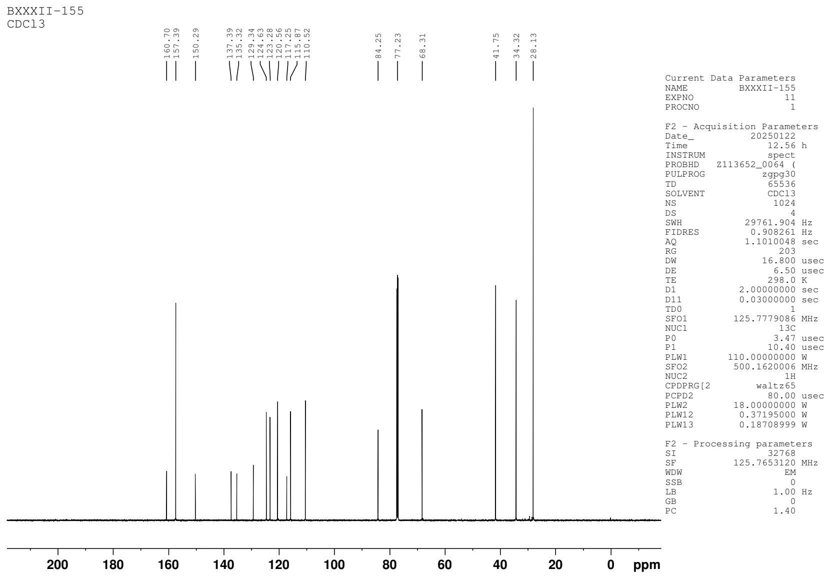

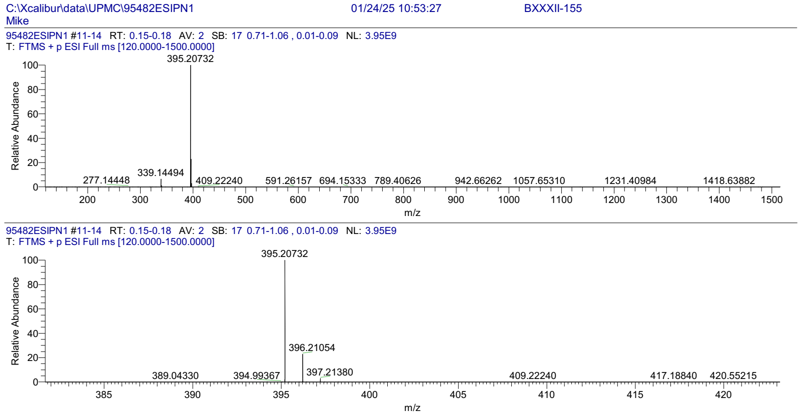

95482ESIPN1#11-13 RT: 0.15-0.18 AV: 2
SB: 17 0.71-1.06 , 0.01-0.09
T: FTMS + p ESI Full ms [120.0000-1500.0000]
m/z= 381.66155-423.16489

m/z	Intensity	Relative	Theo. Mass	Delta (ppm)	Composition
395.20732	3968109056.0	100.00	395.20777	-1.12	$C_{22}H_{27}O_3N_4$

Figure S57. Compound **50** ^{1}H NMR spectrum (top), ^{13}C NMR spectrum (middle), and high-resolution mass spectrum (bottom).

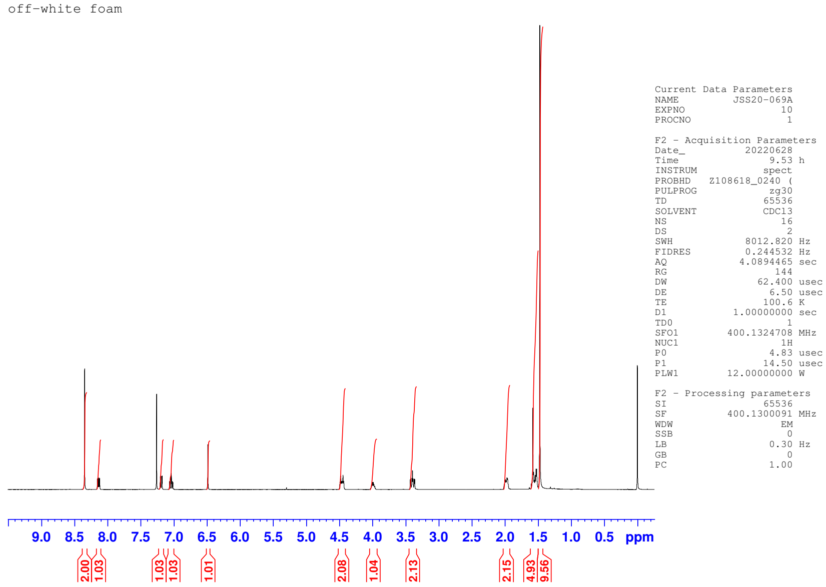

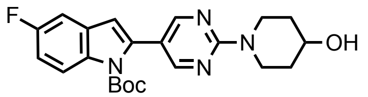

51

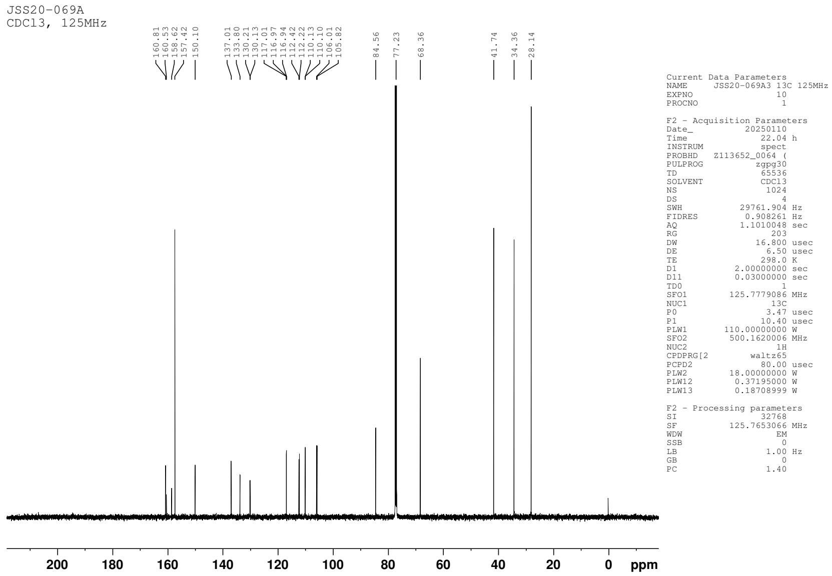

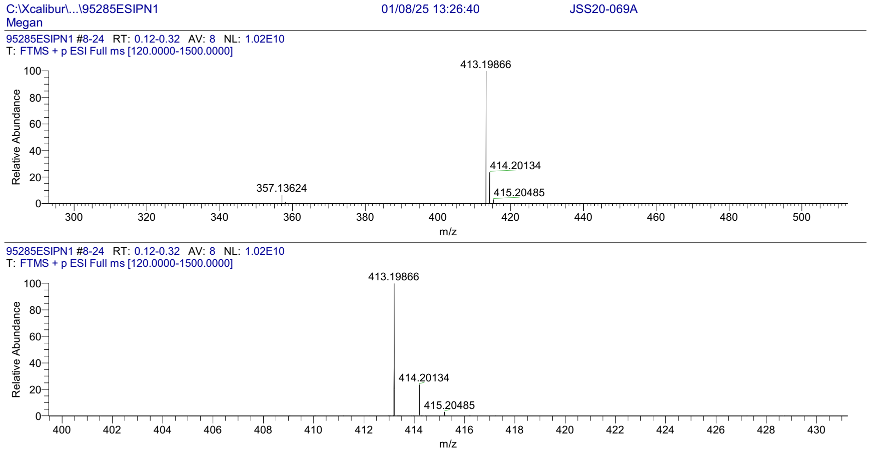

95285ESIPN1#9-23 RT: 0.12-0.32 AV: 8
T: FTMS + p ESI Full ms [120.0000-1500.0000]
m/z= 399.46738-431.23229

m/z	Intensity	Relative	Theo. Mass	Delta (ppm)	Composition
413.19866	10230135808.0	100.00	413.19835	0.76	$C_{22}H_{26}O_3N_4F$

Figure S58. Compound **51** ^{1}H NMR spectrum (top), ^{13}C NMR spectrum (middle), and high-resolution mass spectrum (bottom).

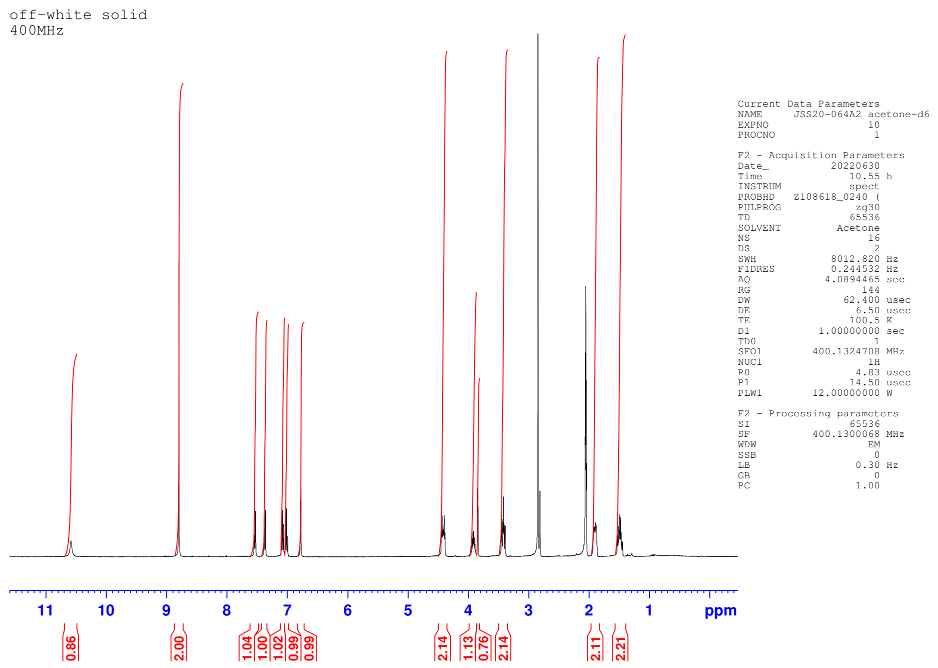

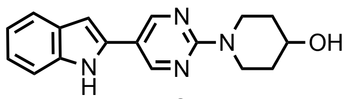

52

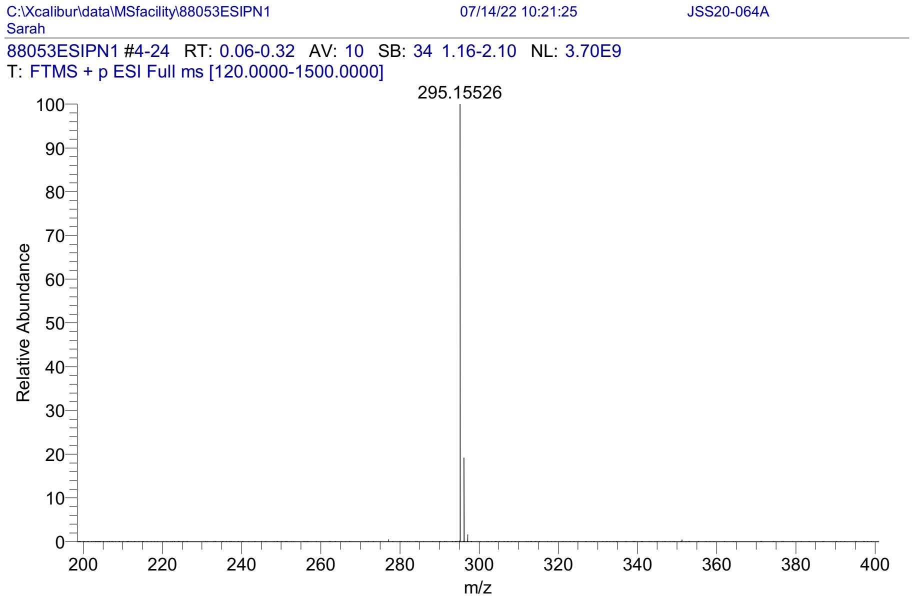

88053ESIPN1#5-23 RT: 0.06-0.32 AV: 10
SB: 34 1.16-2.10
T: FTMS + p ESI Full ms [120.0000-1500.0000]

m/z	Intensity	Relative	Theo. Mass	Delta (ppm)	Composition
295.15526	3837855744.0	100.00	295.15534	-0.26	$C_{17}H_{19}ON_4$

Figure S59. Compound **52** ^{1}H NMR spectrum (top) and high-resolution mass spectrum (bottom).

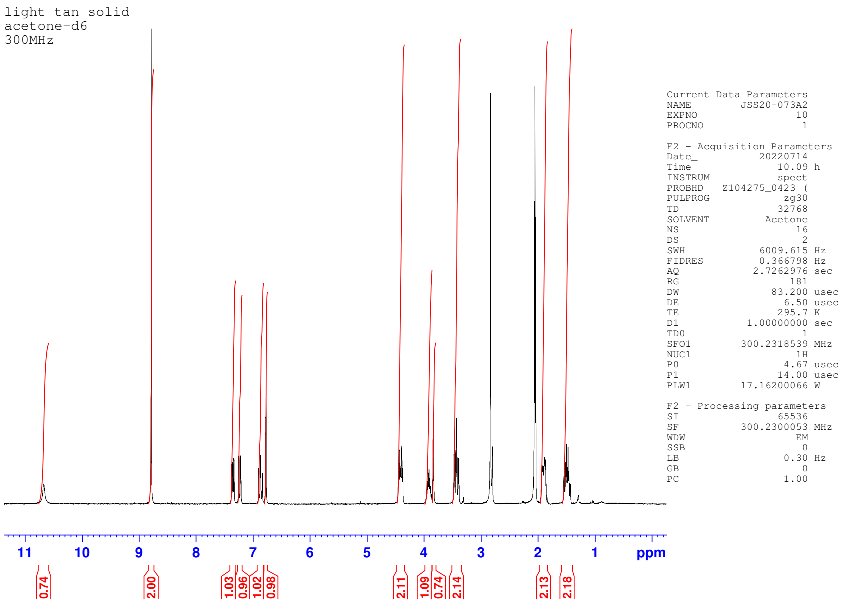

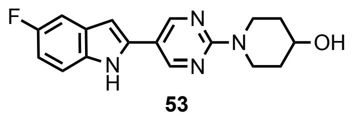

53

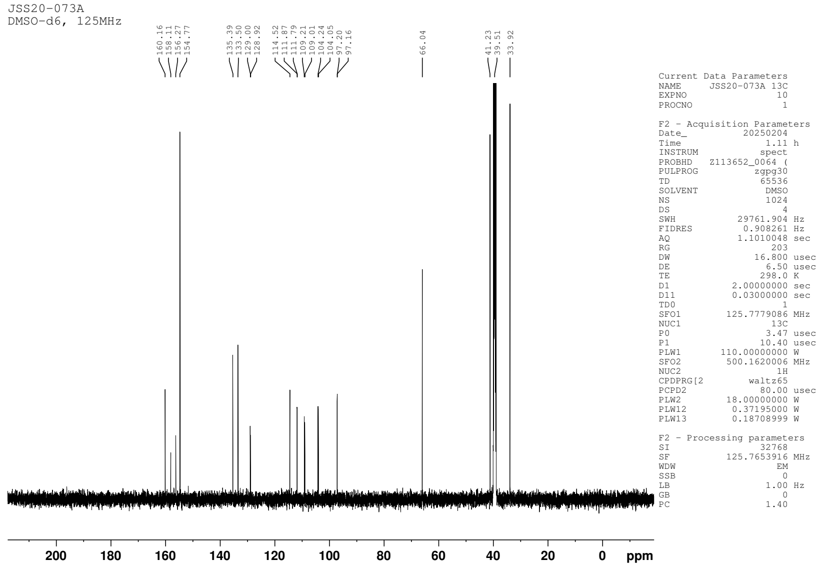

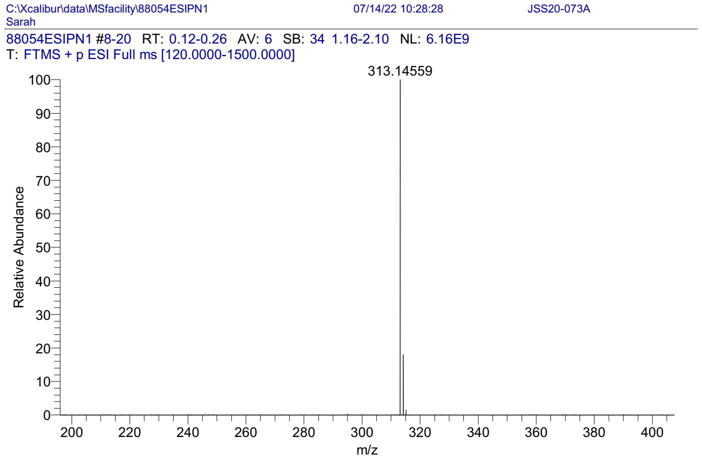

88054ESIPN1#9-19 RT: 0.12-0.26 AV: 6
SB: 34 1.16-2.10
T: FTMS + p ESI Full ms [120.0000-1500.0000]

m/z	Intensity	Relative	Theo. Mass	Delta (ppm)	Composition
313.14559	6182631936.0	100.00	313.14592	-1.03	$C_{17}H_{18}ON_4F$

Figure S60. Compound **53** ^{1}H NMR spectrum (top), ^{13}C NMR spectrum (middle), and high-resolution mass spectrum (bottom).

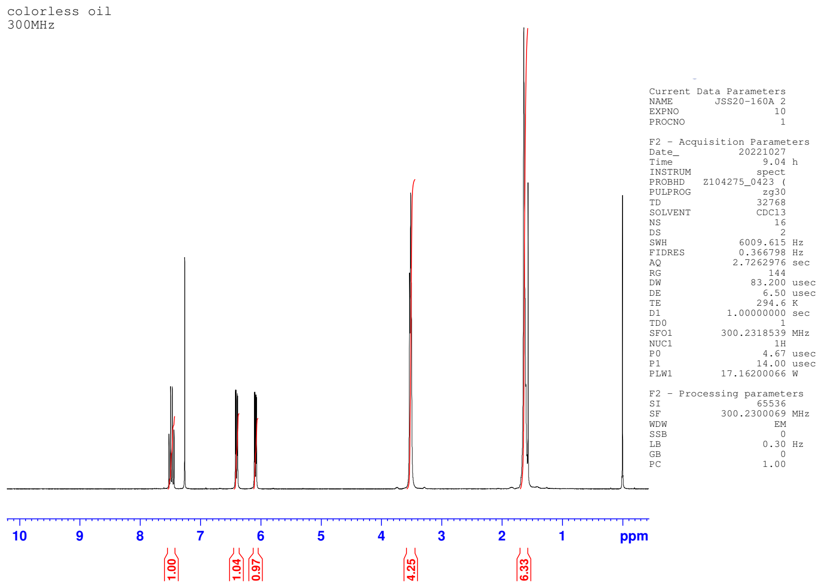

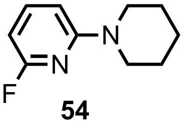

54

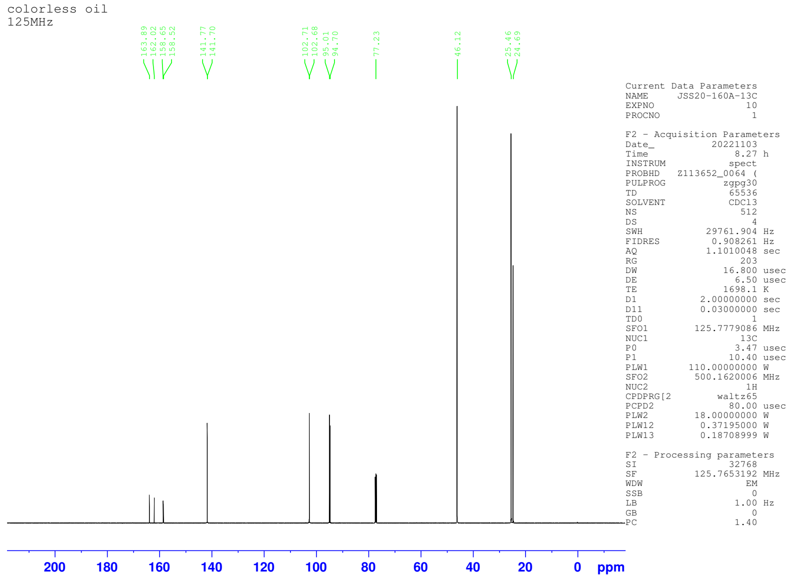

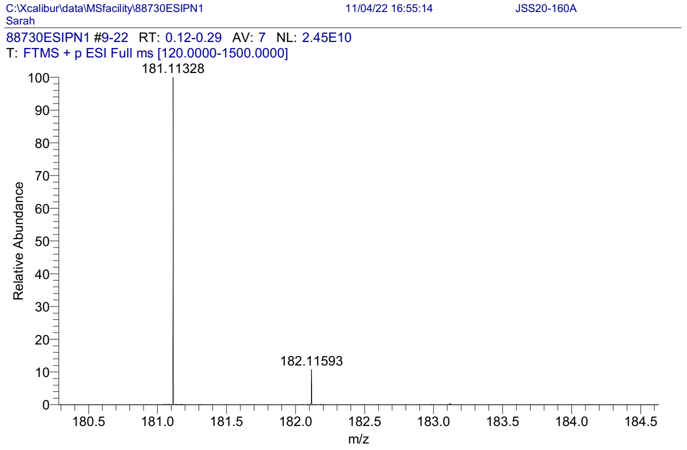

88730ESIPN1#9-21 RT: 0.12-0.29 AV: 7
T: FTMS + p ESI Full ms [120.0000-1500.0000]
m/z= 180.28421-184.63081

m/z	Intensity	Relative	Theo. Mass	Delta (ppm)	Composition
181.11328	24615933952.0	100.00	181.11355	-1.49	$C_{10}H_{14}N_2F$

Figure S61. Compound **54** ^{1}H NMR spectrum (top), ^{13}C NMR spectrum (middle), and high-resolution mass spectrum (bottom).

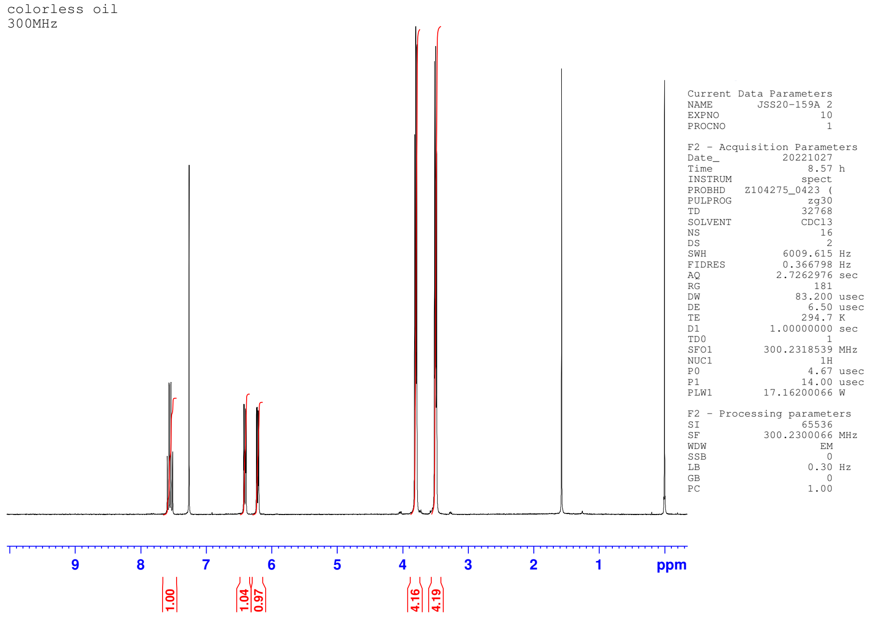

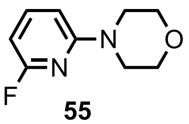

55

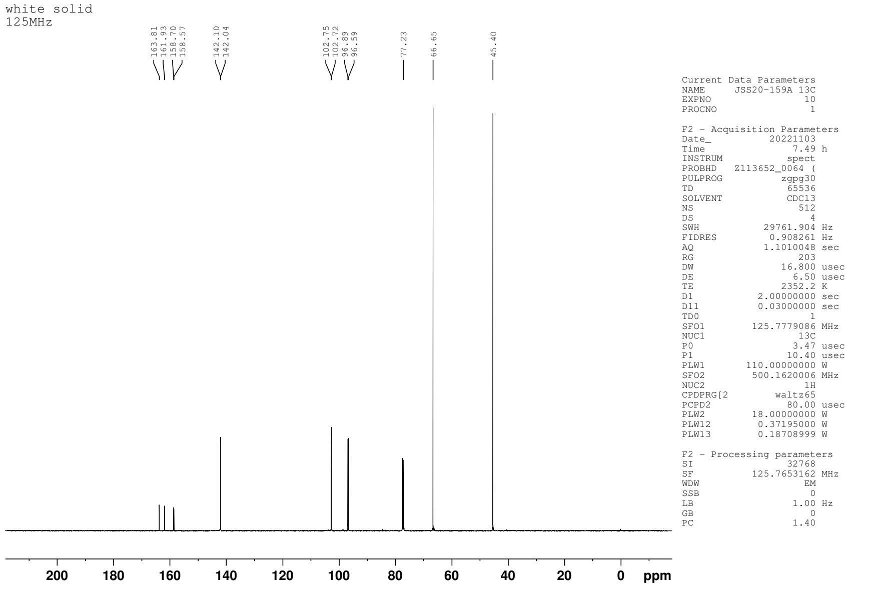

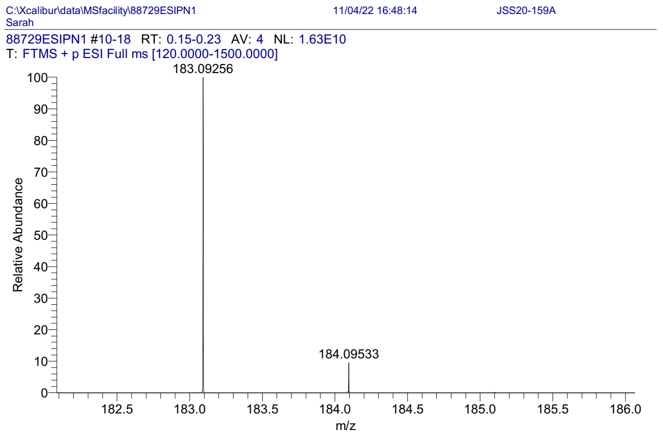

88729ESIPN1#11-17 RT: 0.15-0.23 AV: 4
T: FTMS + p ESI Full ms [120.0000-1500.0000]
m/z= 182.08435-186.06478

m/z	Intensity	Relative	Theo. Mass	Delta (ppm)	Composition
183.09256	16264146944.0	100.00	183.09282	-1.38	$C_9H_{12}ON_2F$

Figure S62. Compound **55** ^{1}H NMR spectrum (top), ^{13}C NMR spectrum (middle), and high-resolution mass spectrum (bottom).

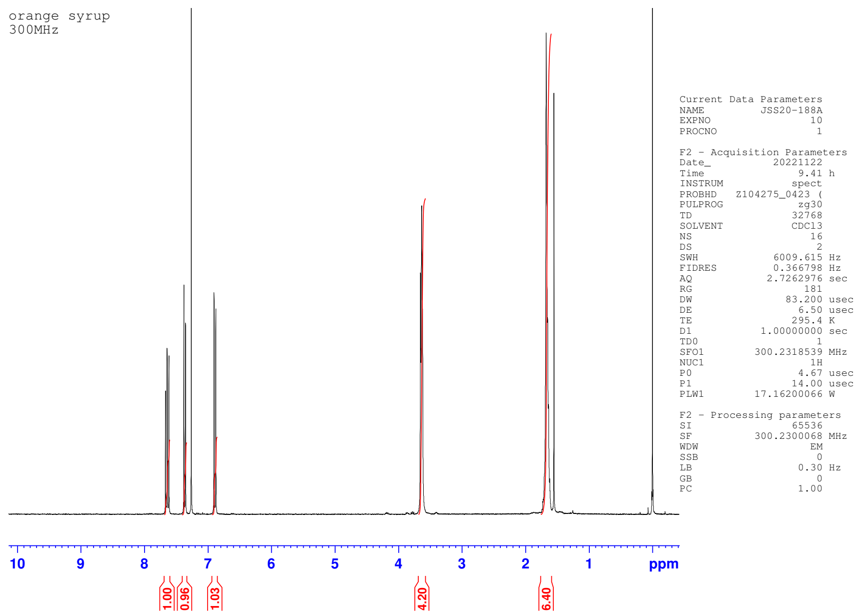

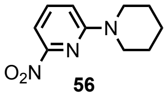

56

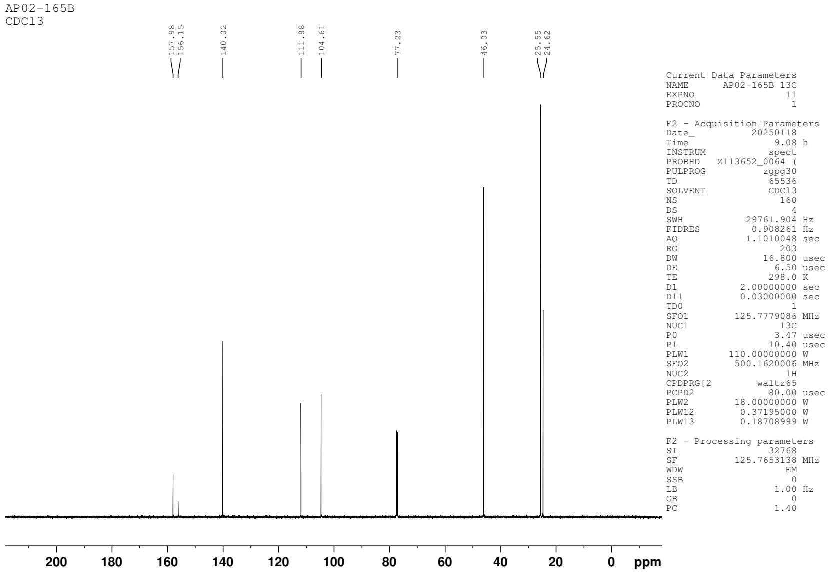

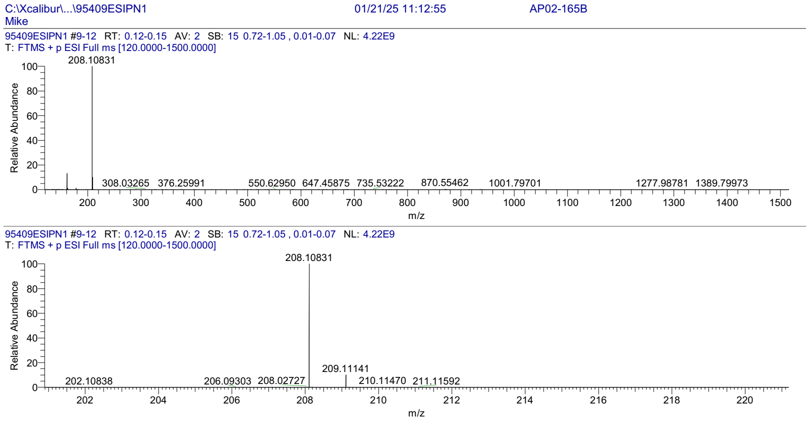

95409ESIPN1#9-11 RT: 0.12-0.15 AV: 2
SB: 15 0.72-1.05 , 0.01-0.07
T: FTMS + p ESI Full ms [120.0000-1500.0000]
m/z= 200.89233-221.18286

m/z	Intensity	Relative	Theo. Mass	Delta (ppm)	Composition
208.10831	4240568320.0	100.00	208.10805	1.23	$C_{10}H_{14}O_2N_3$

Figure S63. Compound **56** ^{1}H NMR spectrum (top), ^{13}C NMR spectrum (middle), and high-resolution mass spectrum (bottom).

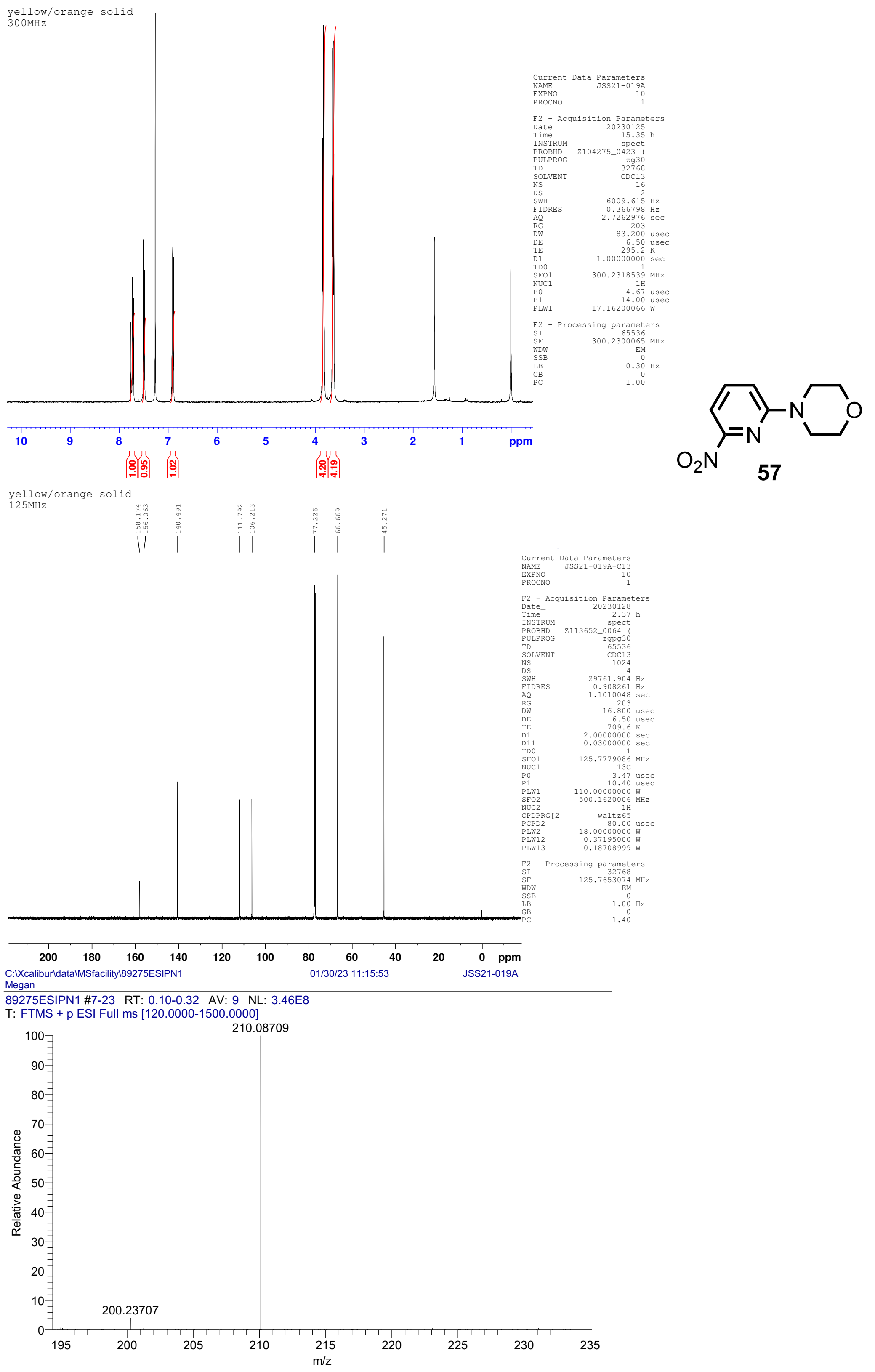

89275ESIPN1#7-23 RT: 0.10-0.32 AV: 9
T: FTMS + p ESI Full ms [120.0000-1500.0000]
m/z= 194.35026-235.11148

m/z	Intensity	Relative	Theo. Mass	Delta (ppm)	Composition
210.08709	351968000.0	100.00	210.08732	-1.10	$C_9H_{12}O_3N_3$

Figure S64. Compound **57** ^{1}H NMR spectrum (top), ^{13}C NMR spectrum (middle), and high-resolution mass spectrum (bottom).

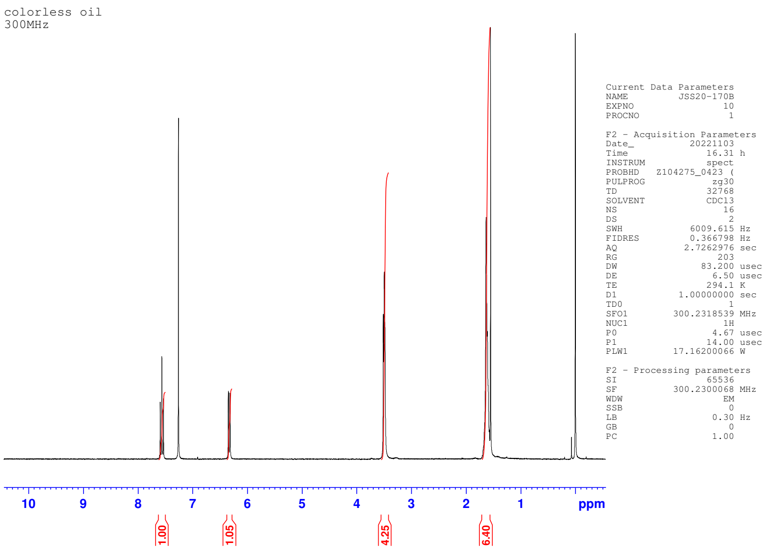

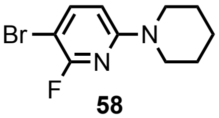

58

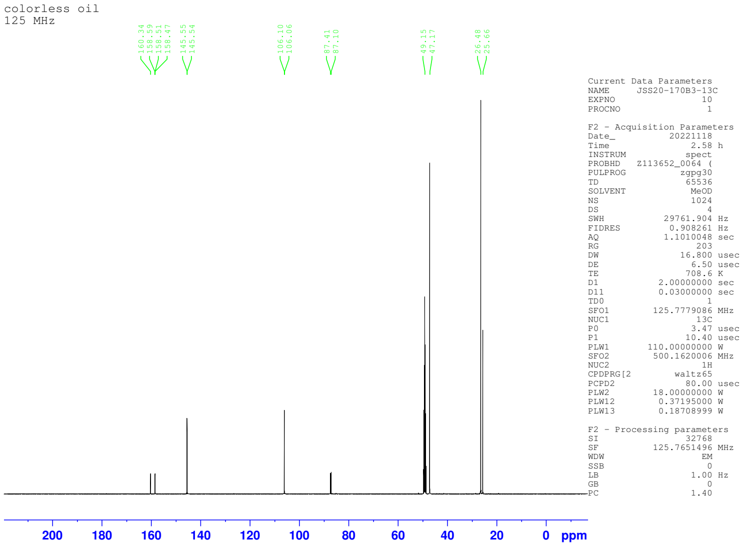

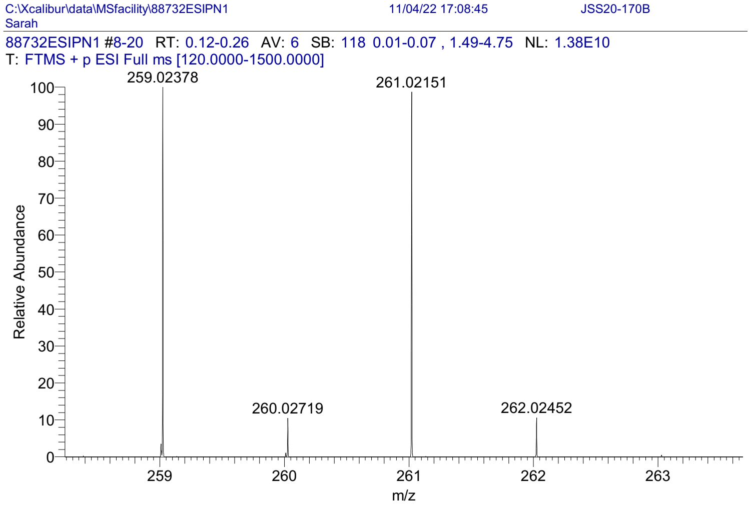

88732ESIPN1#9-19 RT: 0.12-0.26 AV: 6
SB: 118 0.01-0.07 , 1.49-4.75
T: FTMS + p ESI Full ms [120.0000-1500.0000]
m/z= 258.23689-263.68037

m/z	Intensity	Relative	Theo. Mass	Delta (ppm)	Composition
259.02378	13897758720.0	100.00	259.02407	-1.09	$C_{10}H_{13}N_2BrF$

Figure S65. Compound **58** ^{1}H NMR spectrum (top), ^{13}C NMR spectrum (middle), and high-resolution mass spectrum (bottom).

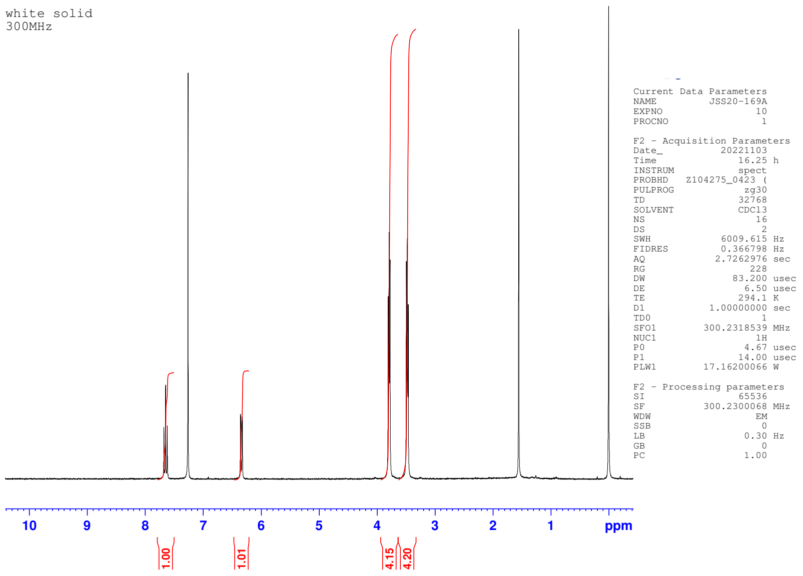

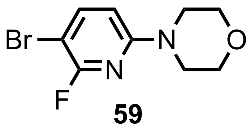

59

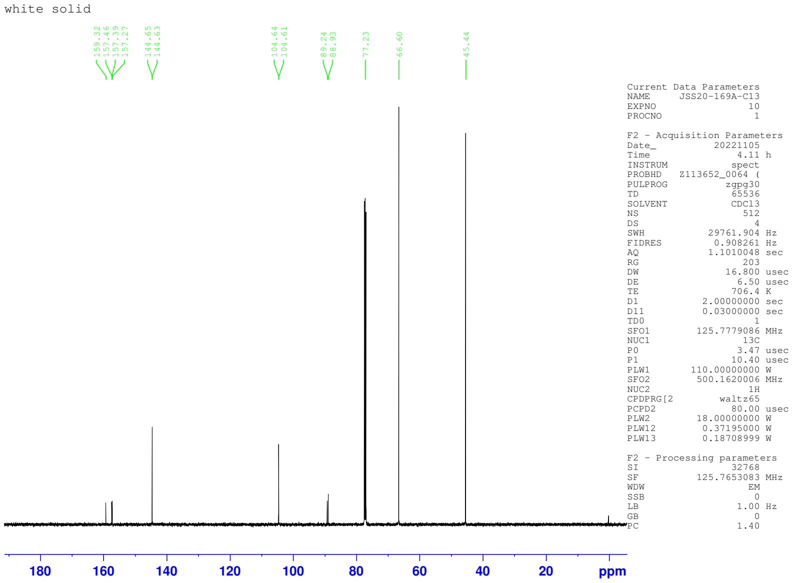

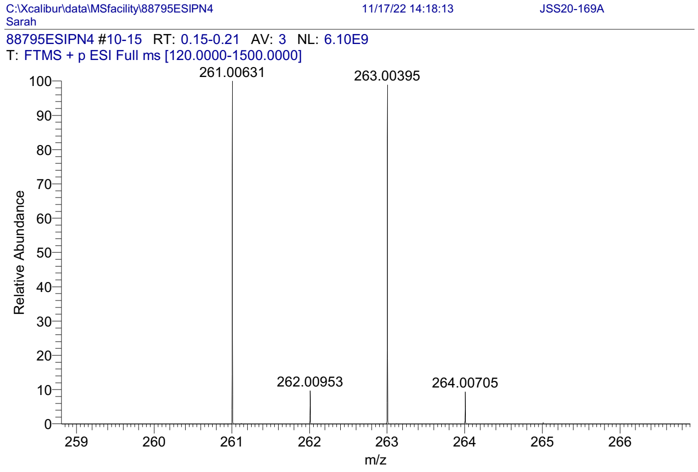

88795ESIPN4#11-15 RT: 0.15-0.21 AV: 3
T: FTMS + p ESI Full ms [120.0000-1500.0000]
m/z= 258.80333-266.90327

m/z	Intensity	Relative	Theo. Mass	Delta (ppm)	Composition
261.00631	6166740480.0	100.00	261.00333	2.98	$C_9H_{11}ON_2BrF$

Figure S66. Compound **59** ^{1}H NMR spectrum (top), ^{13}C NMR spectrum (middle), and high-resolution mass spectrum (bottom).

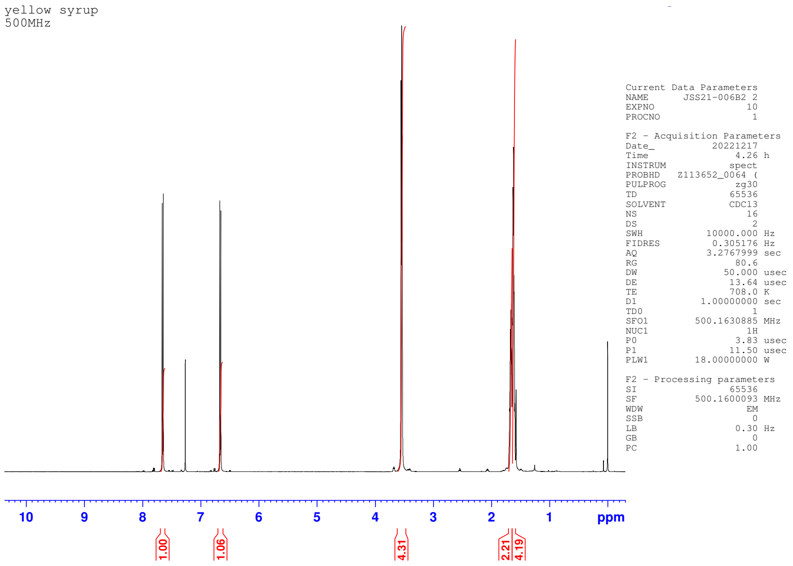

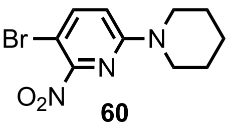

60

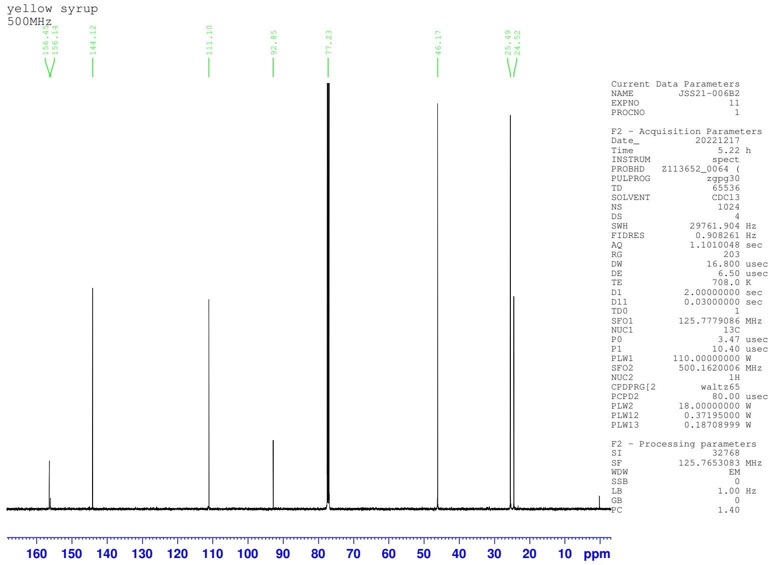

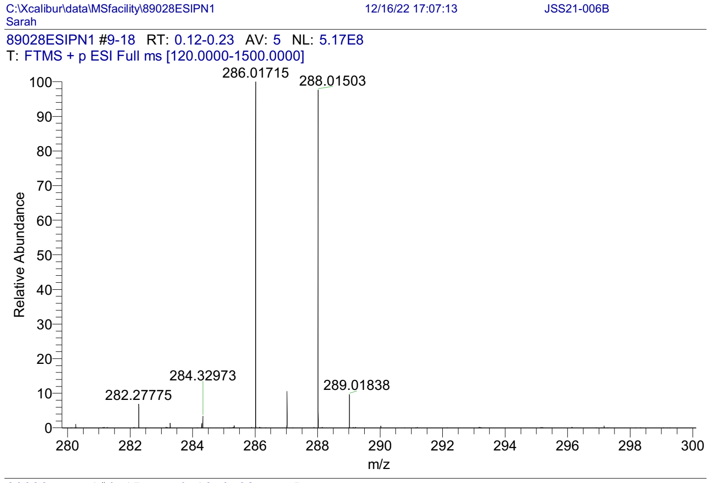

89028ESIPN1#9-17 RT: 0.12-0.23 AV: 5
T: FTMS + p ESI Full ms [120.0000-1500.0000]
m/z= 279.81619-300.10734

m/z	Intensity	Relative	Theo. Mass	Delta (ppm)	Composition
286.01715	517168608.0	100.00	286.01857	-4.93	$C_{10}H_{13}O_2N_3Br$

Figure S67. Compound **60** ^{1}H NMR spectrum (top), ^{13}C NMR spectrum (middle), and high-resolution mass spectrum (bottom).

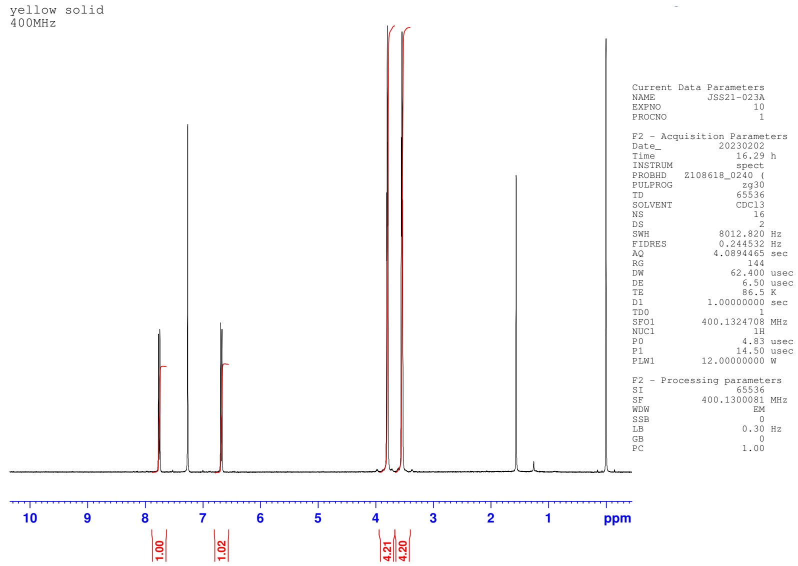

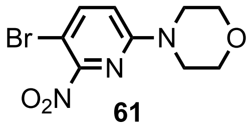

61

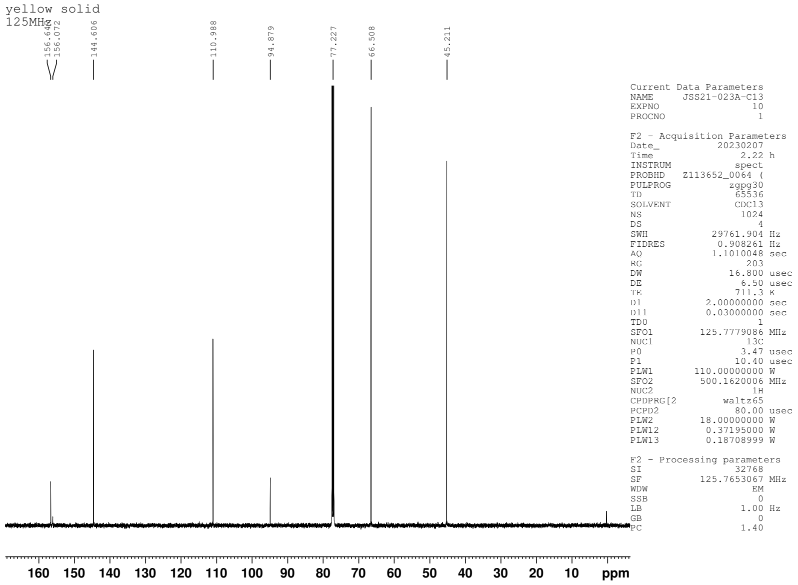

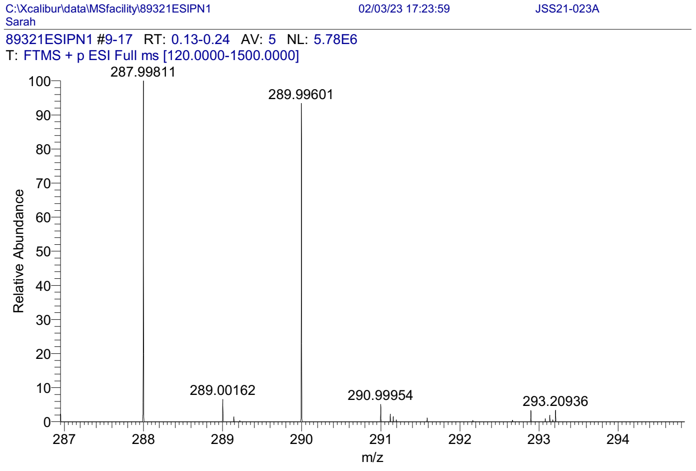

89321ESIPN1#9-17 RT: 0.13-0.24 AV: 5
T: FTMS + p ESI Full ms [120.0000-1500.0000]
m/z= 286.94879-294.84087

m/z	Intensity	Relative	Theo. Mass	Delta (ppm)	Composition
287.99811	5776413.0	100.00	287.99783	0.98	$C_9 H_{11} O_3 N_3 Br$

Figure S68. Compound **61** ^{1}H NMR spectrum (top), ^{13}C NMR spectrum (middle), and high-resolution mass spectrum (bottom).

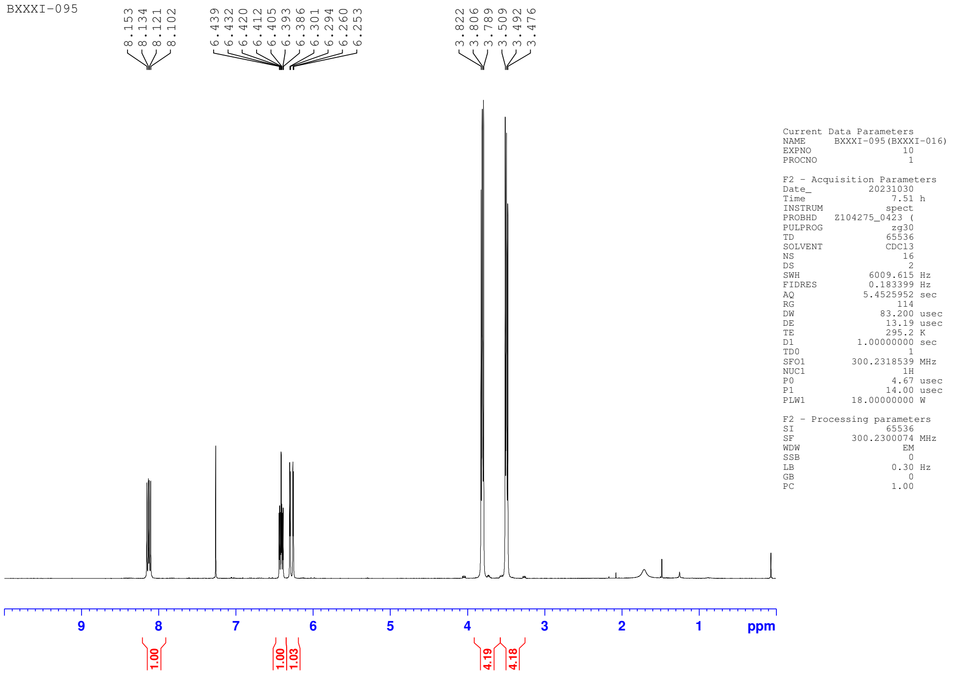

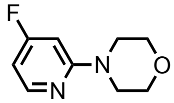

62

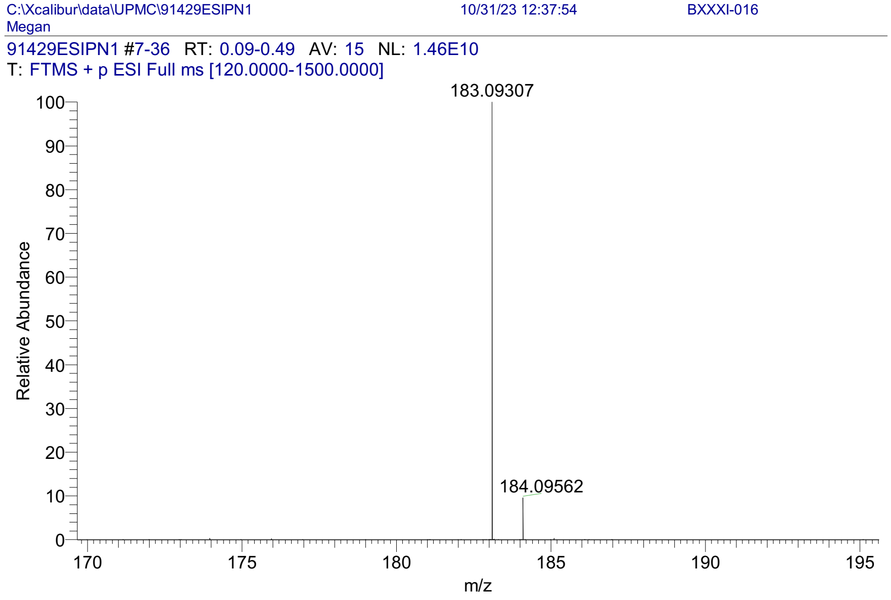

91429ESIPN1#7-35 RT: 0.09-0.49 AV: 15
T: FTMS + p ESI Full ms [120.0000-1500.0000]
m/z= 169.65996-195.61581

m/z	Intensity	Relative	Theo. Mass	Delta (ppm)	Composition
183.09307	15064797184.0	100.00	183.09282	1.35	$C_9H_{12}ON_2F$

Figure S69. Compound **62** ^{1}H NMR spectrum (top) and high-resolution mass spectrum (bottom).

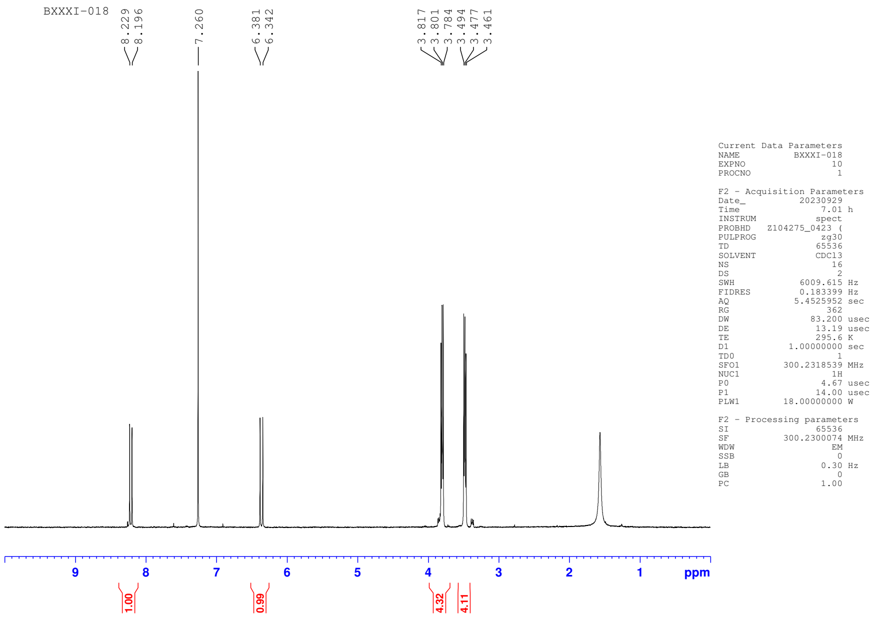

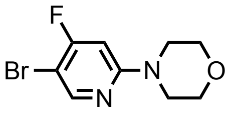

63

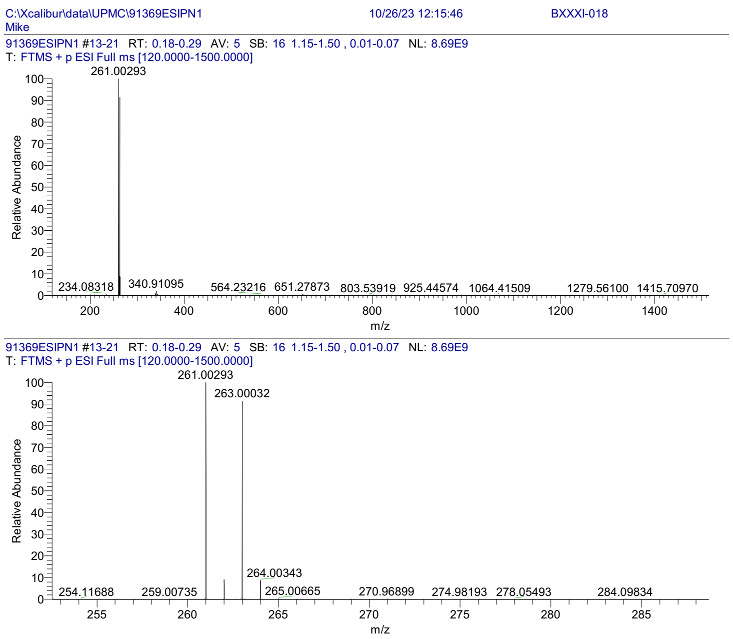

91369ESIPN1#13-21 RT: 0.18-0.29 AV: 5
SB: 16 1.15-1.50 , 0.01-0.07
T: FTMS + p ESI Full ms [120.0000-1500.0000]
m/z= 252.52227-288.69120

m/z	Intensity	Relative	Theo. Mass	Delta (ppm)	Composition
261.00293	8687888384.0	100.00	261.00333	-1.54	$C_9H_{11}ON_2BrF$

Figure S70. Compound **63** ^{1}H NMR spectrum (top) and high-resolution mass spectrum (bottom).

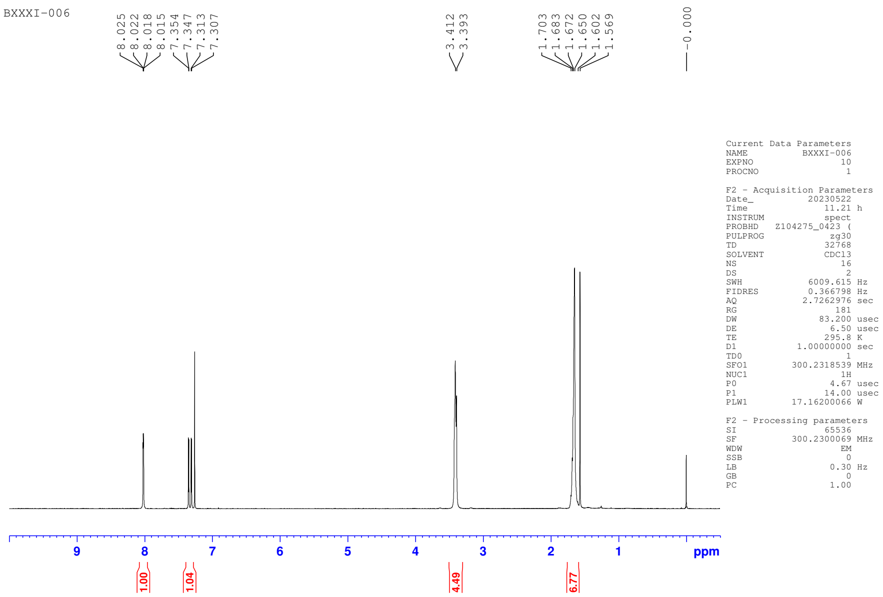

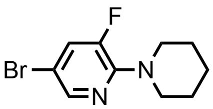

64

C:\Xcalibur\data\UPMC\91370ESIPN1 10/26/23 12:22:32 BXXXI-006
Mike

91370ESIPN1 #10-17 RT: 0.15-0.24 AV: 4 SB: 16 1.19-1.56 , 0.01-0.08 NL: 1.37E10
T: FTMS + p ESI Full ms [120.0000-1500.0000]

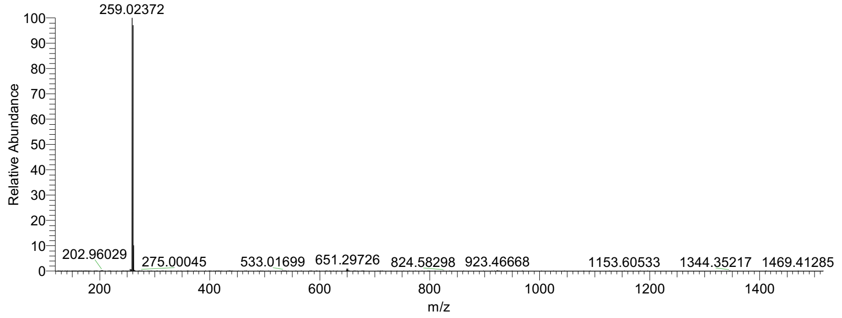

91370ESIPN1 #10-17 RT: 0.15-0.24 AV: 4 SB: 16 1.19-1.56 , 0.01-0.08 NL: 1.37E10
T: FTMS + p ESI Full ms [120.0000-1500.0000]

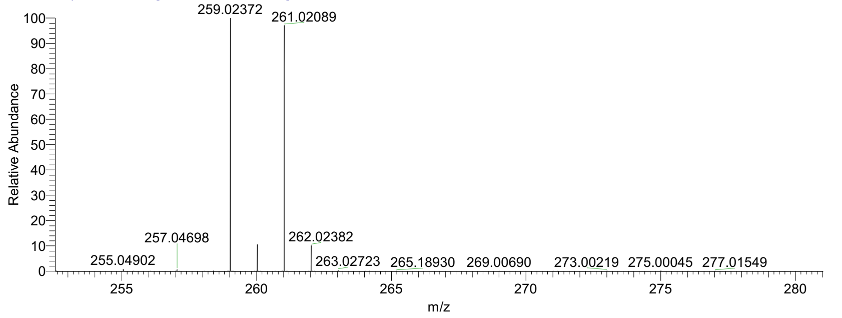

91370ESIPN1#11-17 RT: 0.15-0.24 AV: 4
SB: 16 1.19-1.56 , 0.01-0.08
T: FTMS + p ESI Full ms [120.0000-1500.0000]
m/z= 252.52222-281.01894

m/z	Intensity	Relative	Theo. Mass	Delta (ppm)	Composition
259.02372	13695913984.0	100.00	259.02407	-1.35	$C_{10}H_{13}N_2BrF$

Figure S71. Compound **64** ^{1}H NMR spectrum (top) and high-resolution mass spectrum (bottom).

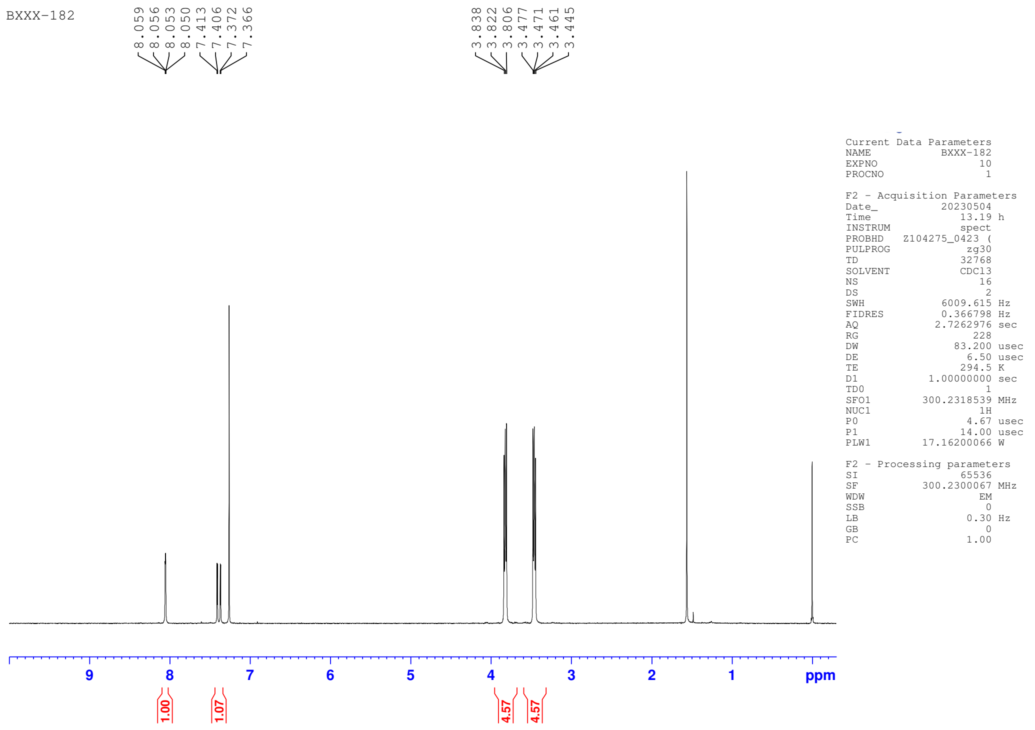

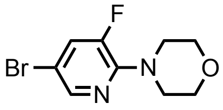

65

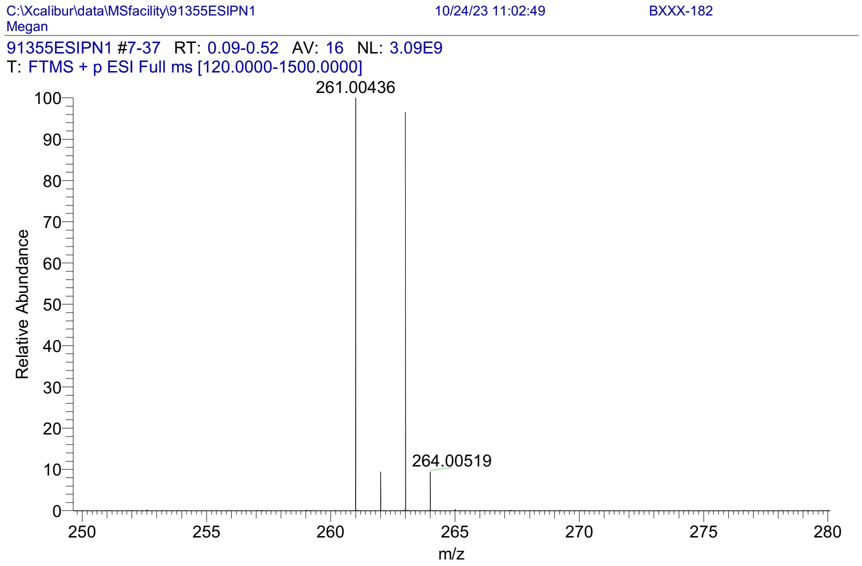

91355ESIPN1#7-37 RT: 0.09-0.52 AV: 16
T: FTMS + p ESI Full ms [120.0000-1500.0000]
m/z= 249.62986-280.08197

m/z	Intensity	Relative	Theo. Mass	Delta (ppm)	Composition
261.00436	3091604224.0	100.00	261.00333	3.96	$C_9H_{11}ON_2BrF$

Figure S72. Compound **65** ^{1}H NMR spectrum (top) and high-resolution mass spectrum (bottom).

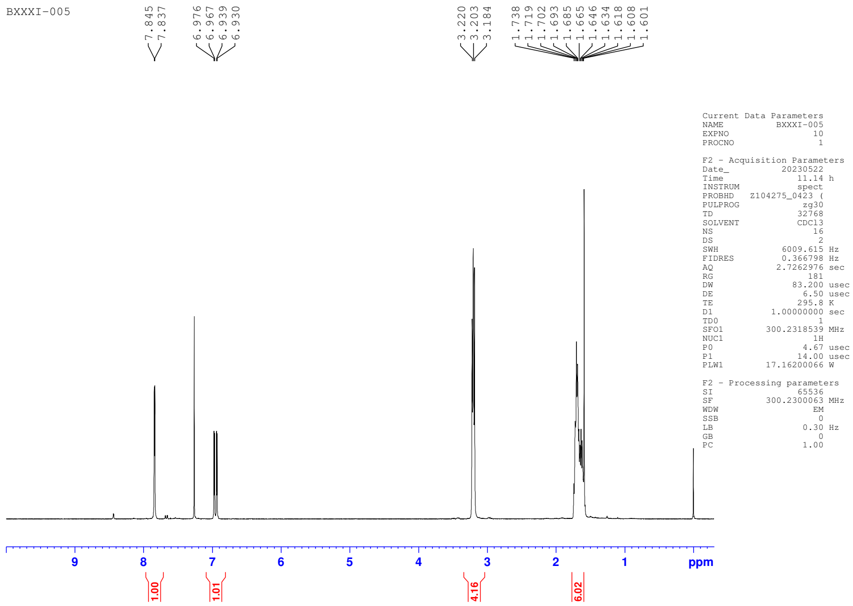

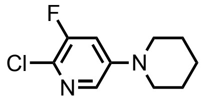

66

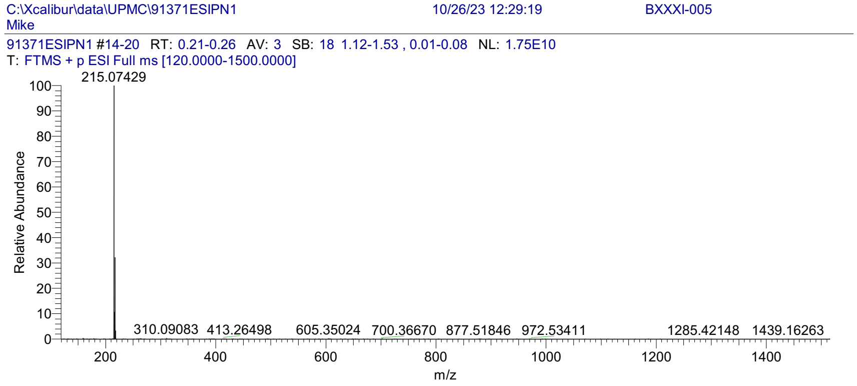

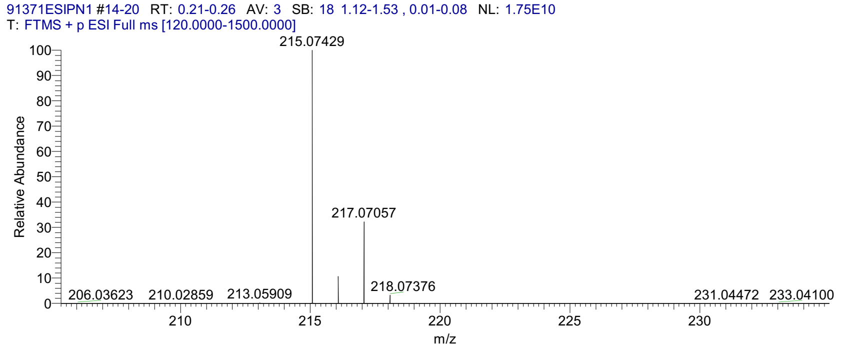

91371ESIPN1#15-19 RT: 0.21-0.26 AV: 3
SB: 18 1.12-1.53 , 0.01-0.08
T: FTMS + p ESI Full ms [120.0000-1500.0000]
m/z= 205.39398-234.98704

m/z	Intensity	Relative	Theo. Mass	Delta (ppm)	Composition
215.07429	17679941632.0	100.00	215.07458	-1.37	$C_{10}H_{13}N_2ClF$

Figure S73. Compound **66** ^{1}H NMR spectrum (top) and high-resolution mass spectrum (bottom).

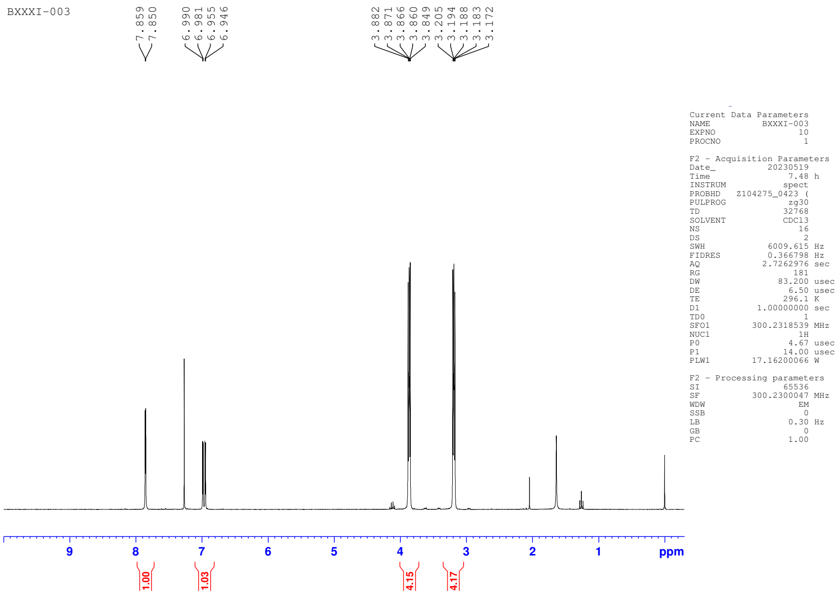

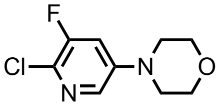

67

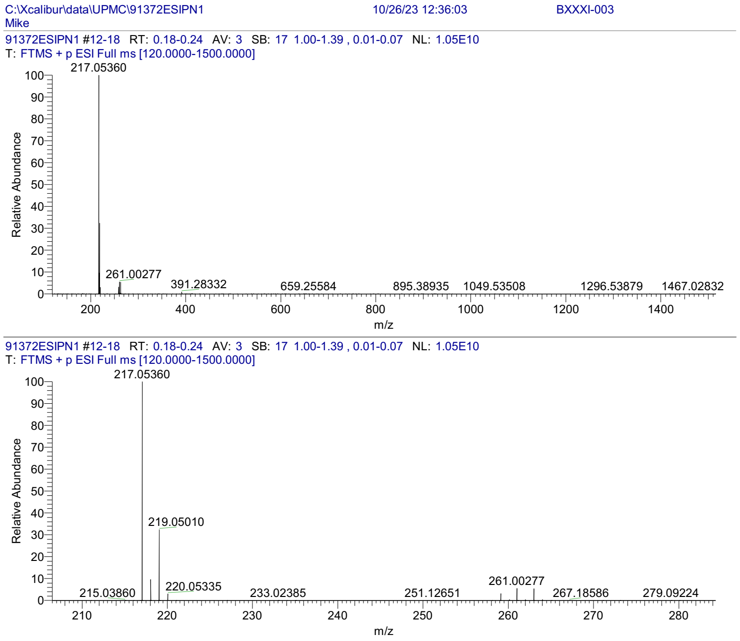

91372ESIPN1#13-17 RT: 0.18-0.24 AV: 3
SB: 17 1.00-1.39 , 0.01-0.07
T: FTMS + p ESI Full ms [120.0000-1500.0000]
m/z= 206.48999-284.30877

m/z	Intensity	Relative	Theo. Mass	Delta (ppm)	Composition
217.05360	10581902336.0	100.00	217.05385	-1.14	$C_9H_{11}ON_2ClF$

Figure S74. Compound **67** ^{1}H NMR spectrum (top), ^{13}C NMR spectrum (middle), high-resolution mass spectrum (bottom).

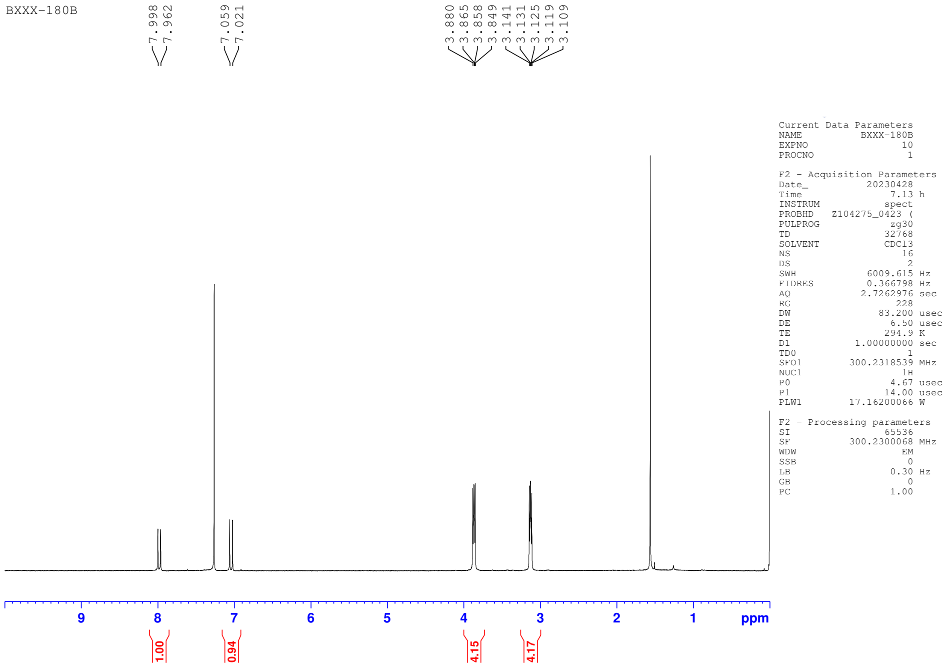

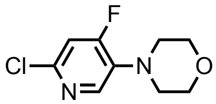

68

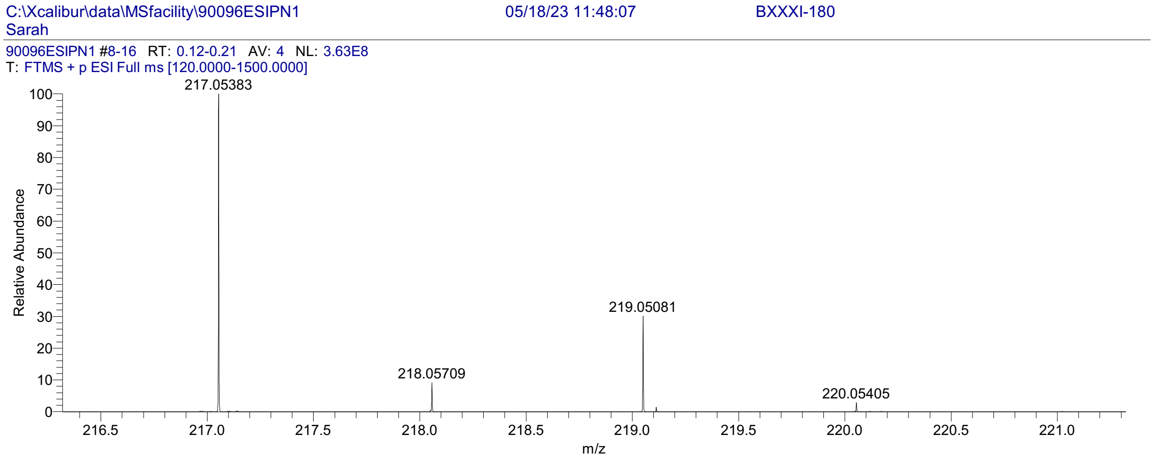

90096ESIPN1#9-15 RT: 0.12-0.21 AV: 4
T: FTMS + p ESI Full ms [120.0000-1500.0000]
m/z= 216.31866-221.32113

m/z	Intensity	Relative	Theo. Mass	Delta (ppm)	Composition
217.05383	370609952.0	100.00	217.05385	-0.07	$C_9H_{11}ON_2ClF$

Figure S75. Compound **68** ^{1}H NMR spectrum (top) and high-resolution mass spectrum (bottom).

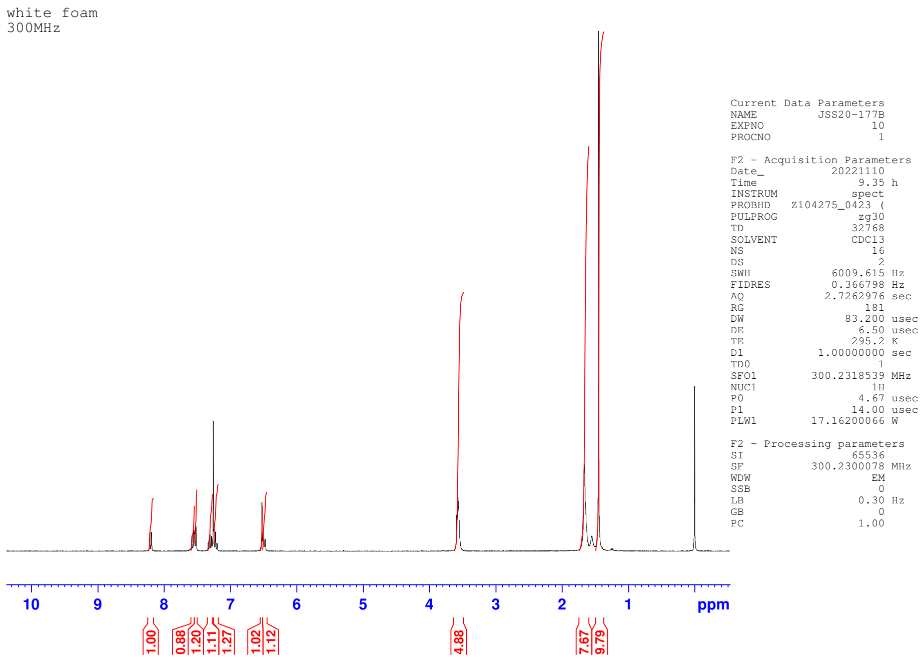

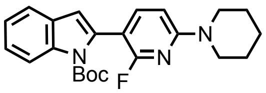

69

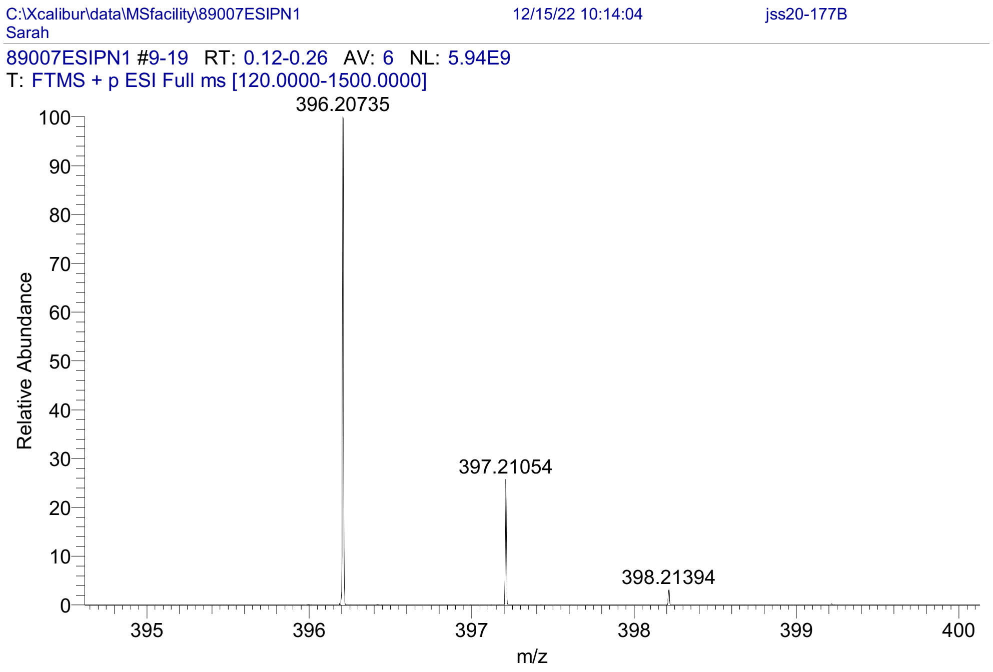

89007ESIPN1#9-19 RT: 0.12-0.26 AV: 6
T: FTMS + p ESI Full ms [120.0000-1500.0000]
m/z= 394.61629-400.12767

m/z	Intensity	Relative	Theo. Mass	Delta (ppm)	Composition
396.20735	6182650880.0	100.00	396.20818	-2.09	$C_{23}H_{27}O_2N_3F$

Figure S76. Compound **69** ^{1}H NMR spectrum (top) and high-resolution mass spectrum (bottom).

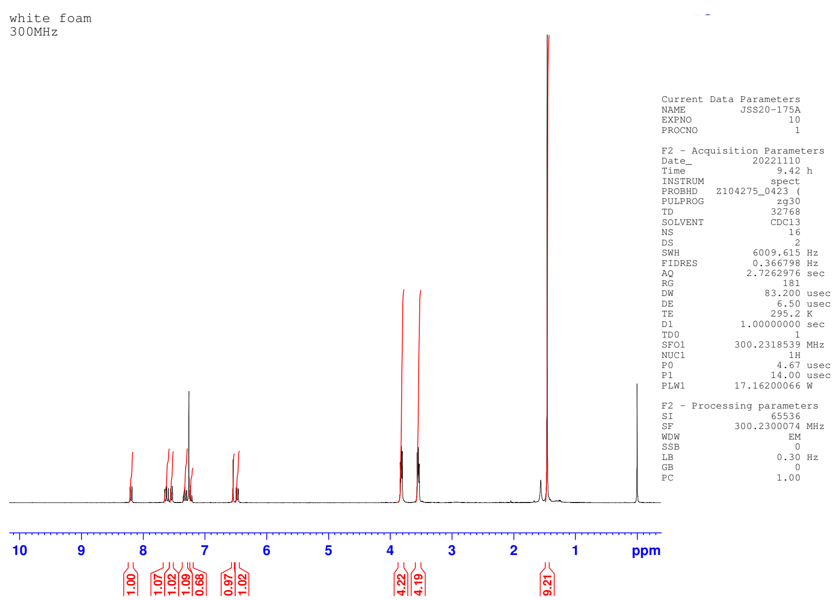

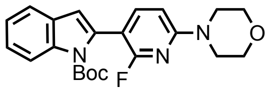

70

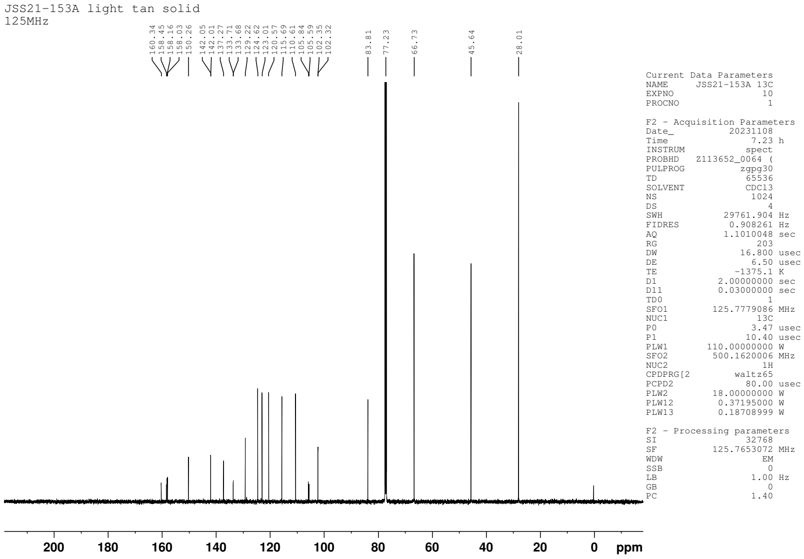

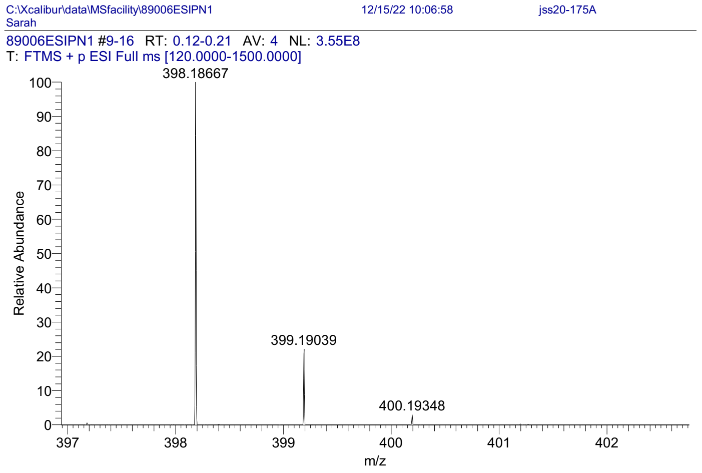

89006ESIPN1#9-15 RT: 0.12-0.21 AV: 4
T: FTMS + p ESI Full ms [120.0000-1500.0000]
m/z= 396.94007-402.76113

m/z	Intensity	Relative	Theo. Mass	Delta (ppm)	Composition
398.18667	355398112.0	100.00	398.18745	-1.94	$C_{22}H_{25}O_3N_3F$

Figure S77. Compound **70** ^{1}H NMR spectrum (top), ^{13}C NMR spectrum (middle), and high-resolution mass spectrum (bottom).

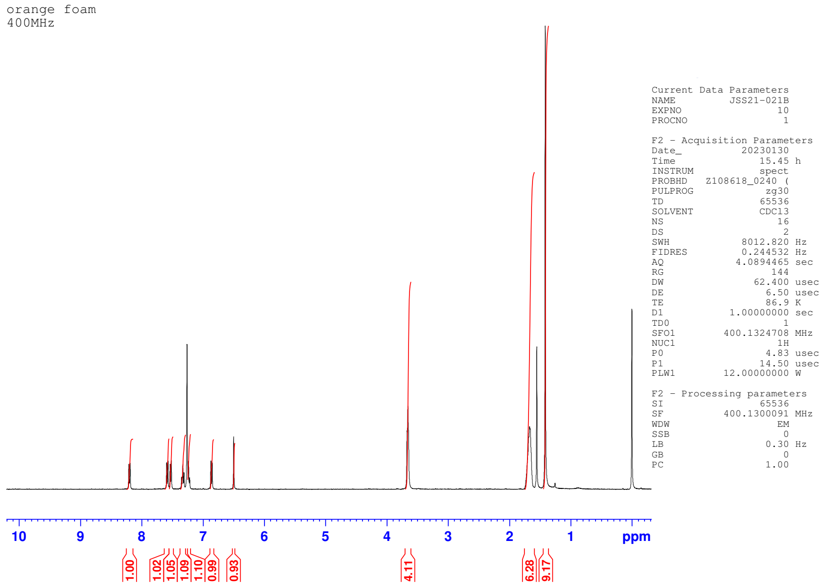

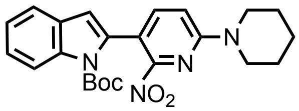

71

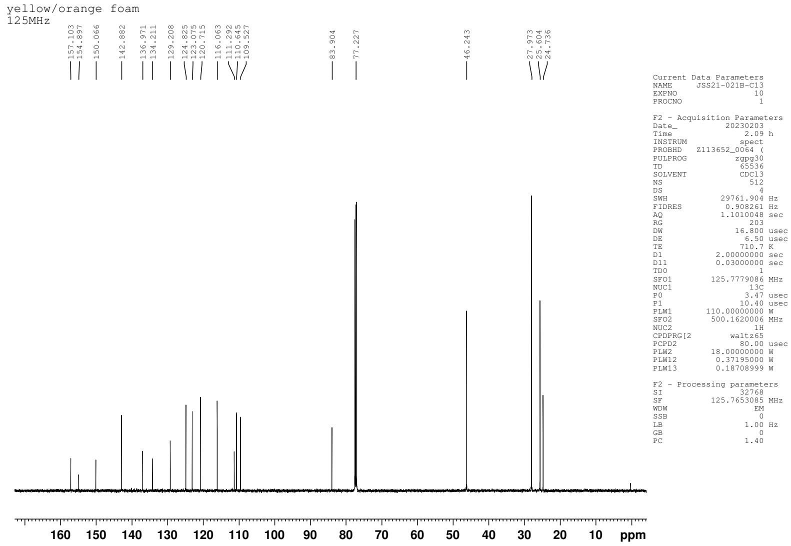

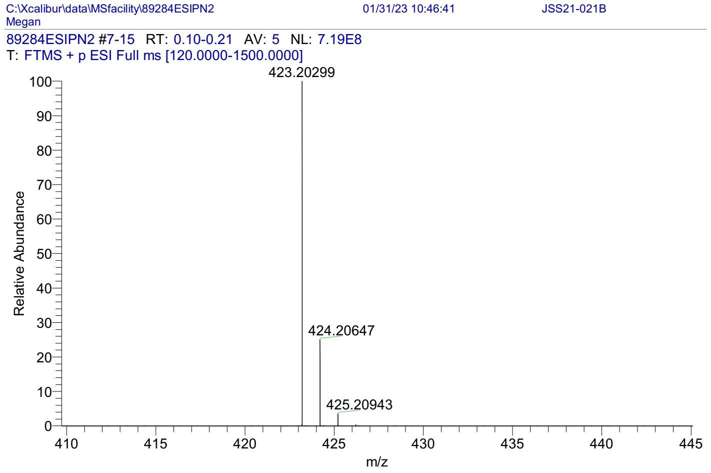

89284ESIPN2#7-15 RT: 0.10-0.21 AV: 5
T: FTMS + p ESI Full ms [120.0000-1500.0000]
m/z= 409.71417-445.09268

m/z	Intensity	Relative	Theo. Mass	Delta (ppm)	Composition
423.20299	742180096.0	100.00	423.20268	0.73	$C_{23}H_{27}O_4N_4$

Figure S78. Compound **71** ^{1}H NMR spectrum (top), ^{13}C NMR spectrum (middle), and high-resolution mass spectrum (bottom).

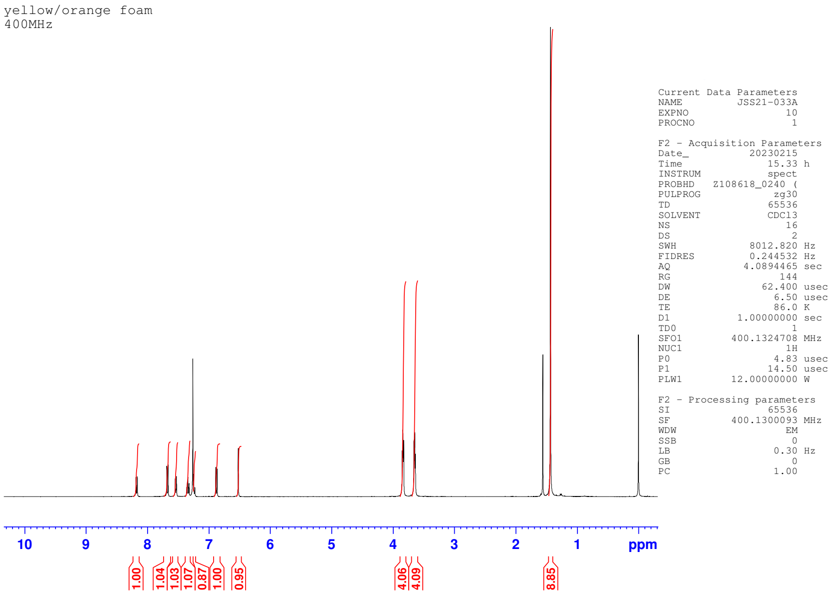

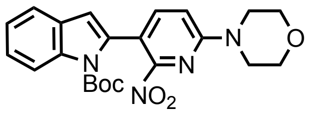

72

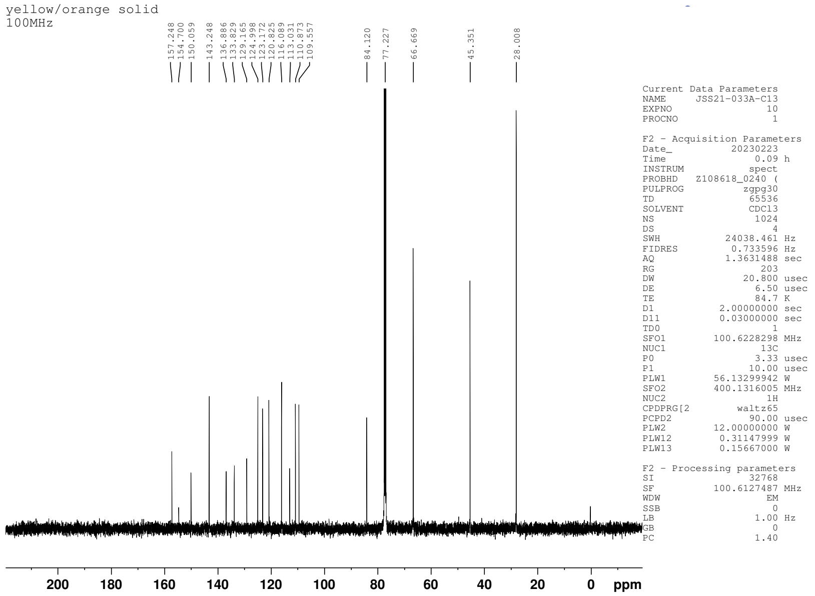

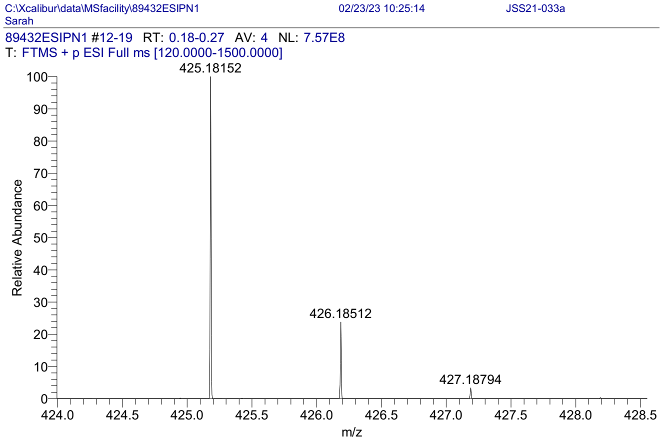

89432ESIPN1#13-19 RT: 0.18-0.27 AV: 4
T: FTMS + p ESI Full ms [120.0000-1500.0000]
m/z= 423.99300-428.55014

m/z	Intensity	Relative	Theo. Mass	Delta (ppm)	Composition
425.18152	758258752.0	100.00	425.18195	-1.00	$C_{22}H_{25}O_5N_4$

Figure S79. Compound **72** ^{1}H NMR spectrum (top), ^{13}C NMR spectrum (middle), and high-resolution mass spectrum (bottom).

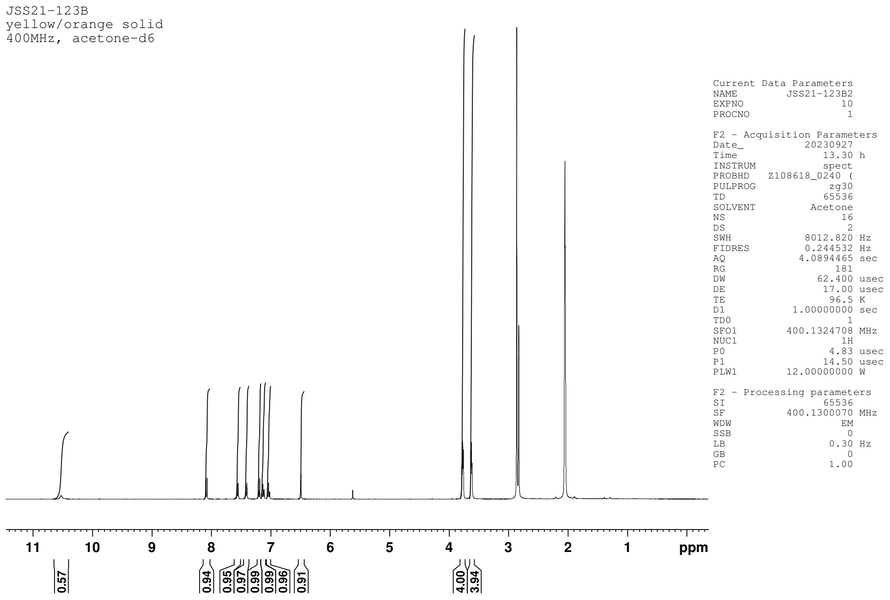

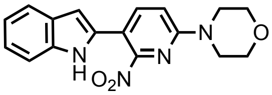

73

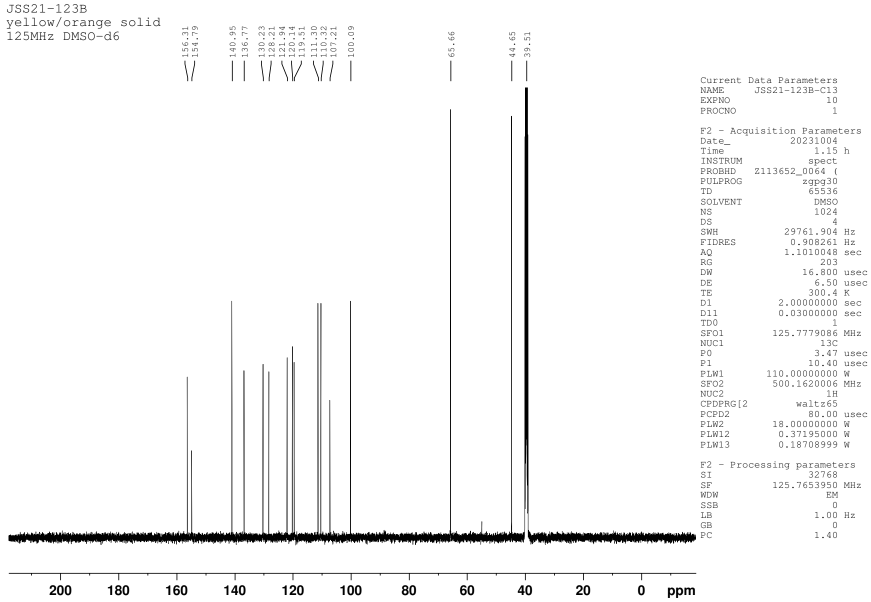

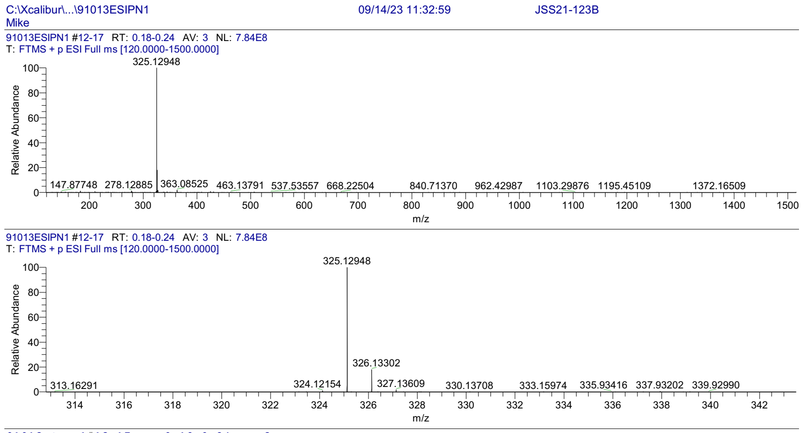

91013ESIPN1#13-17 RT: 0.18-0.24 AV: 3
T: FTMS + p ESI Full ms [120.0000-1500.0000]
m/z= 312.80561-343.49456

m/z	Intensity	Relative	Theo. Mass	Delta (ppm)	Composition
325.12948	784773568.0	100.00	325.12952	-0.11	$C_{17}H_{17}O_3N_4$

Figure S80. Compound **73** ^{1}H NMR spectrum (top), ^{13}C NMR spectrum (middle), and high-resolution mass spectrum (bottom).

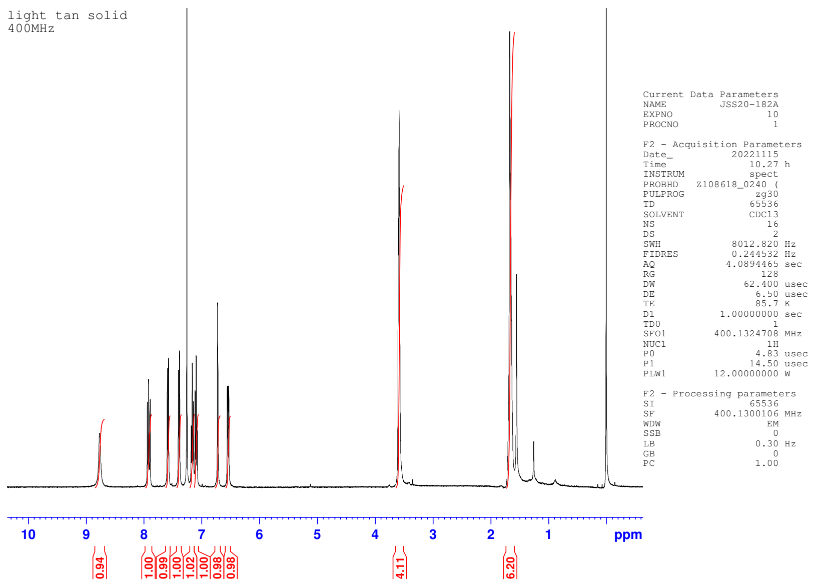

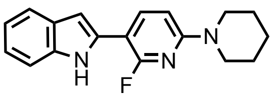

74

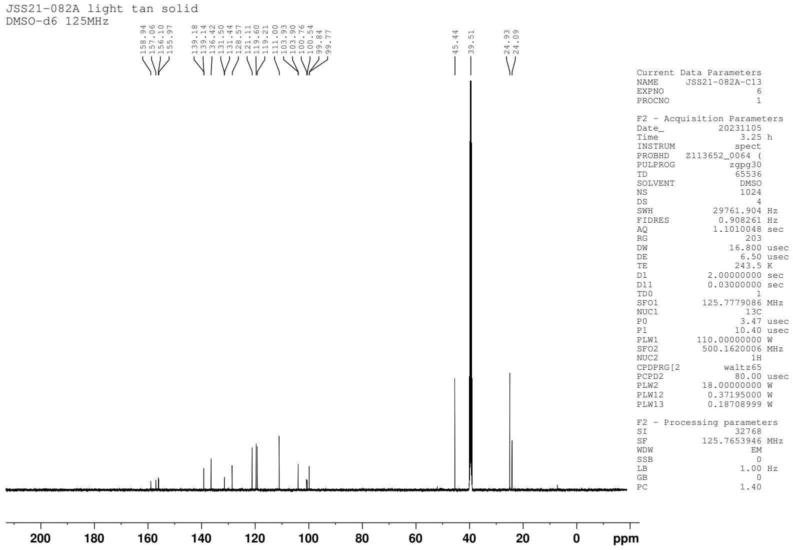

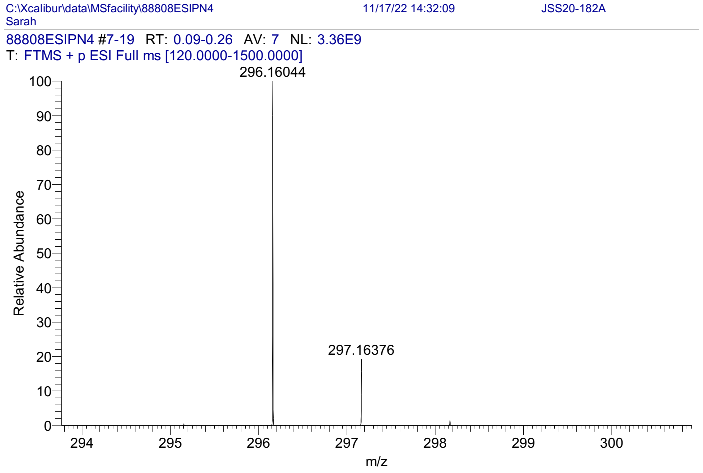

88808ESIPN4#7-19 RT: 0.09-0.26 AV: 7
T: FTMS + p ESI Full ms [120.0000-1500.0000]
m/z= 293.76436-300.90926

m/z	Intensity	Relative	Theo. Mass	Delta (ppm)	Composition
296.16044	3389452032.0	100.00	296.15575	4.68	$C_{18}H_{19}N_3F$

Figure S81. Compound **74** ^{1}H NMR spectrum (top), ^{13}C NMR spectrum (middle), and high-resolution mass spectrum (bottom).

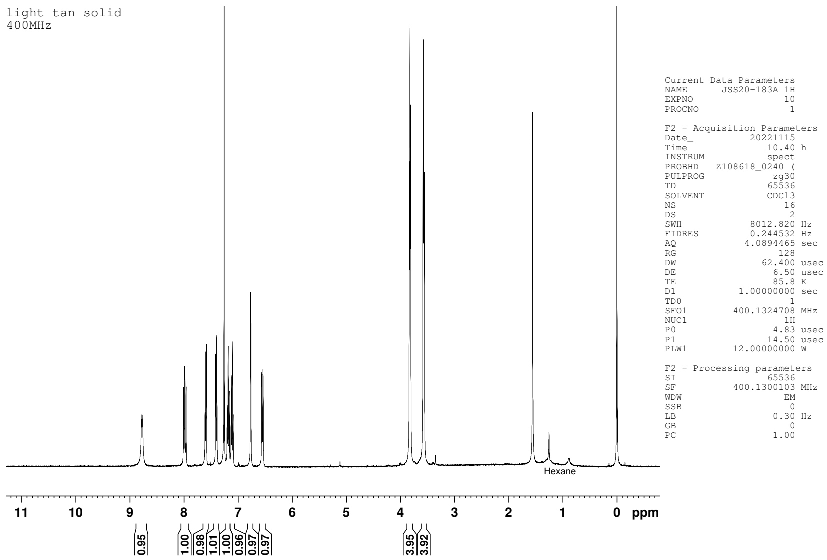

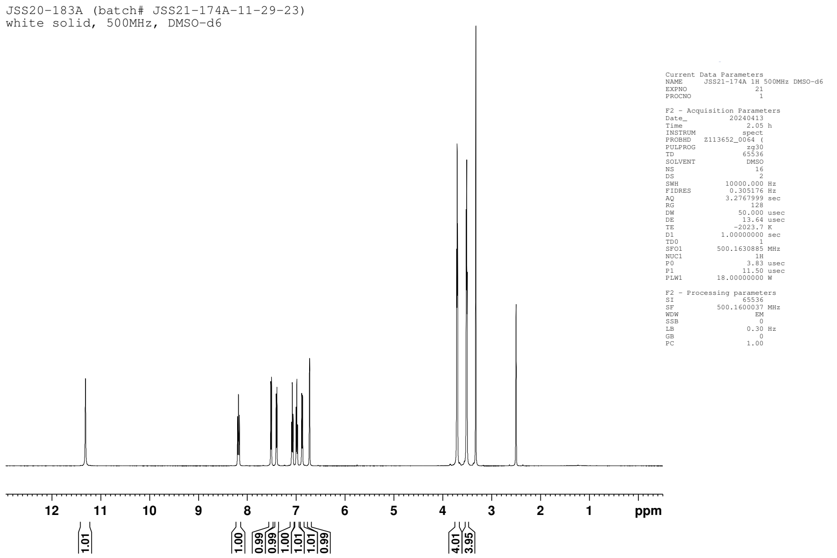

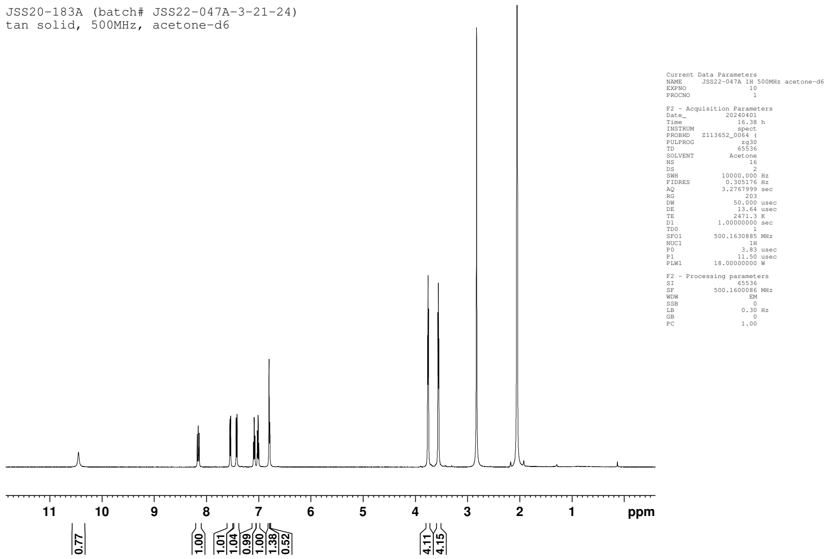

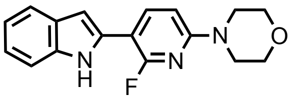

75

Figure S82. Compound **75** ^{1}H NMR spectra in $CDCl_3$ (top), DMSO-d_6 (middle), and acetone-d_6 (bottom).

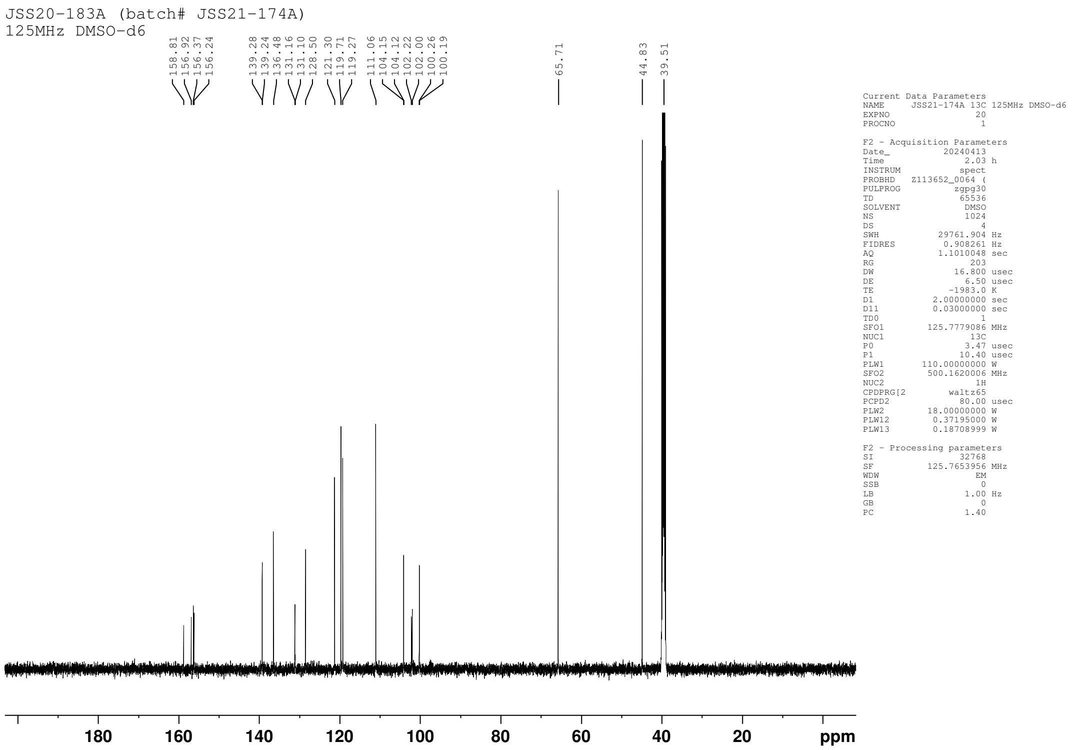

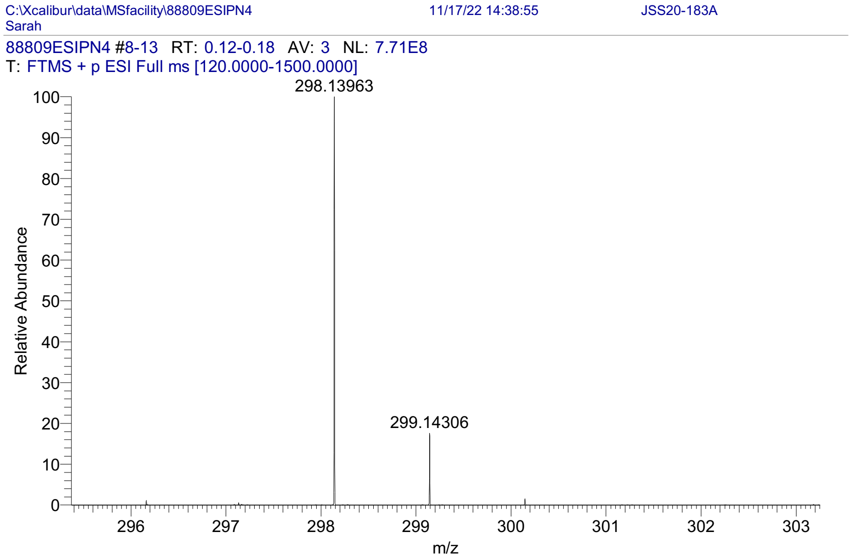

88809ESIPN4#9-13 RT: 0.12-0.18 AV: 3
T: FTMS + p ESI Full ms [120.0000-1500.0000]
m/z= 295.37040-303.25121

m/z	Intensity	Relative	Theo. Mass	Delta (ppm)	Composition
298.13963	784189376.0	100.00	298.13502	4.61	$C_{17}H_{17}ON_3F$

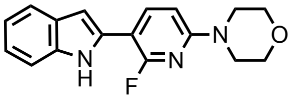

75

Figure S83. Compound **75** ^{13}C NMR spectrum (top) and high-resolution mass spectrum (bottom).

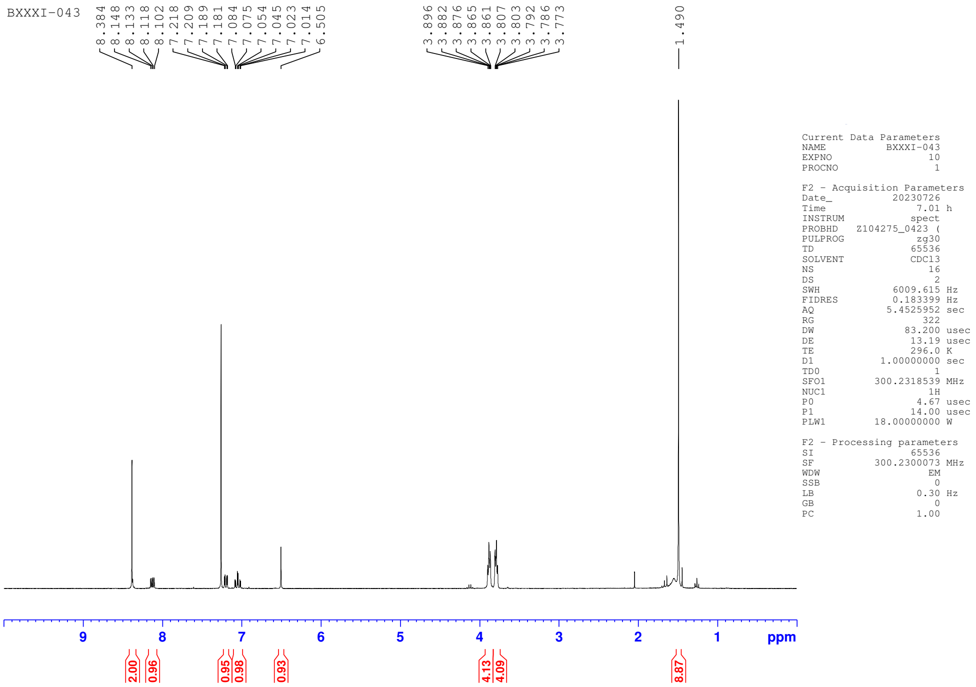

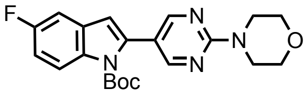

76

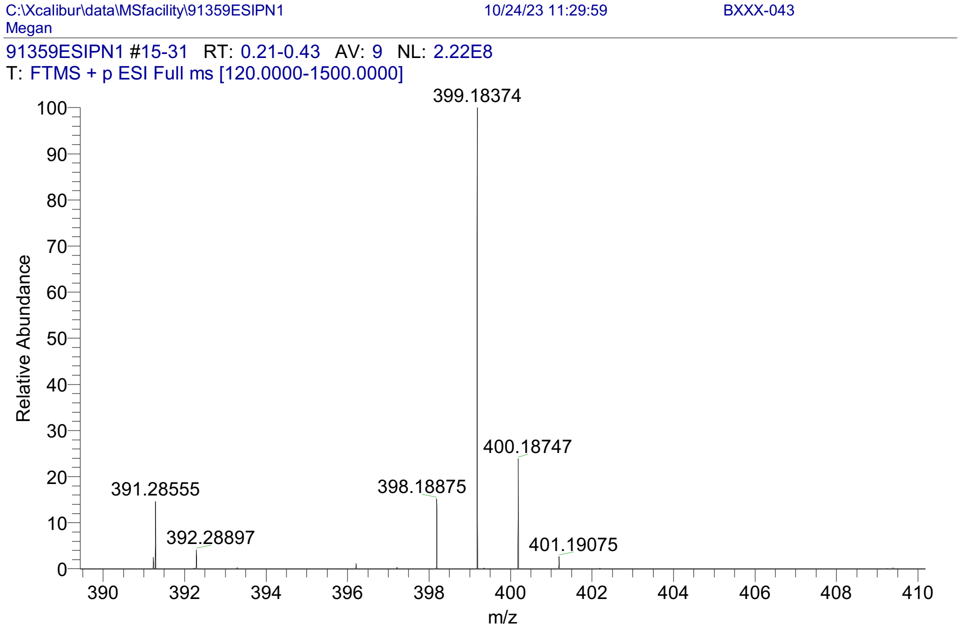

91359ESIPN1#15-31 RT: 0.21-0.43 AV: 9
T: FTMS + p ESI Full ms [120.0000-1500.0000]
m/z= 389.43346-410.17931

m/z	Intensity	Relative	Theo. Mass	Delta (ppm)	Composition
399.18374	225761696.0	100.00	399.18270	2.63	$C_{21}H_{24}O_3N_4F$

Figure S84. Compound **76** ^{1}H NMR spectrum (top) and high-resolution mass spectrum (bottom).

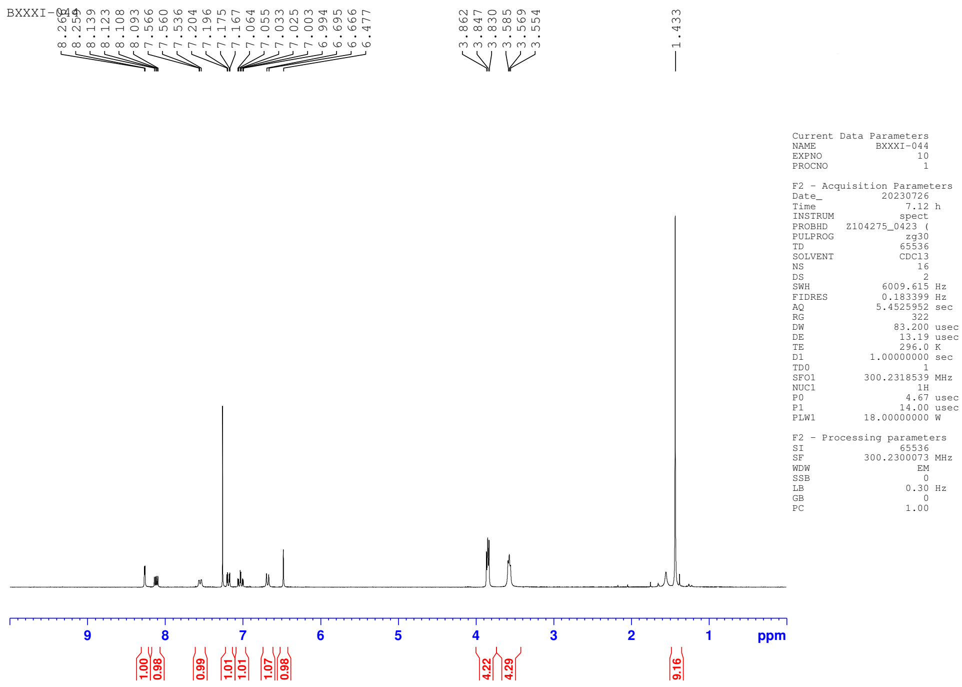

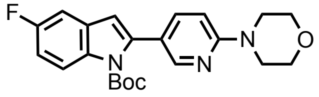

77

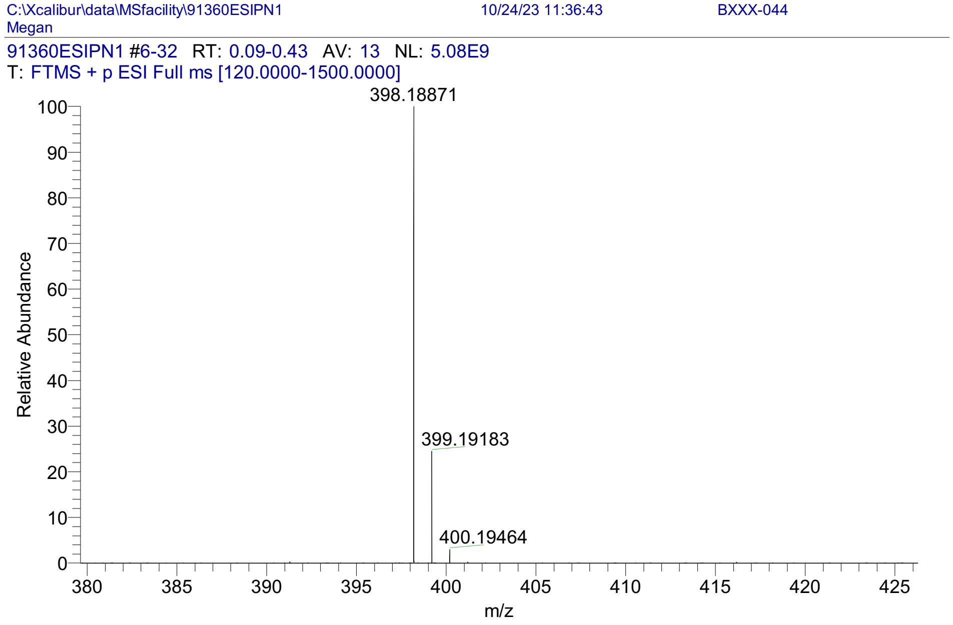

91360ESIPN1#7-31 RT: 0.09-0.43 AV: 13
T: FTMS + p ESI Full ms [120.0000-1500.0000]
m/z= 379.61385-426.26451

m/z	Intensity	Relative	Theo. Mass	Delta (ppm)	Composition
398.18871	5209212416.0	100.00	398.18745	3.18	$C_{22}H_{25}O_3N_3F$

Figure S85. Compound **77** ¹H NMR spectrum (top) and high-resolution mass spectrum (bottom).

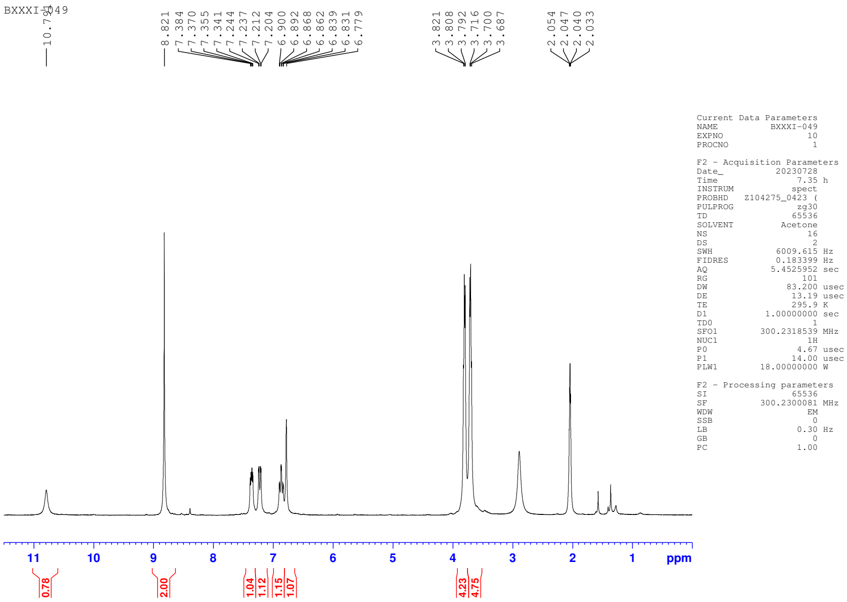

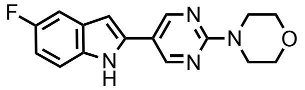

78

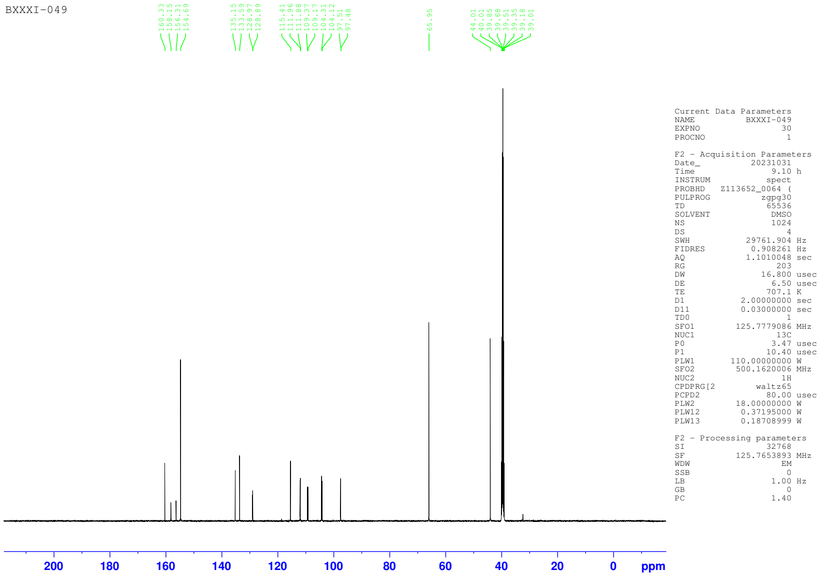

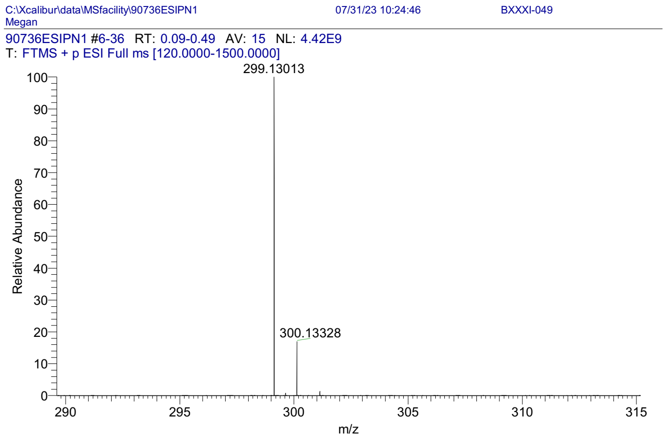

90736ESIPN1#7-35 RT: 0.09-0.49 AV: 15
T: FTMS + p ESI Full ms [120.0000-1500.0000]
m/z= 289.61055-315.17356

m/z	Intensity	Relative	Theo. Mass	Delta (ppm)	Composition
299.13013	4565434368.0	100.00	299.13027	-0.45	$C_{16}H_{16}ON_4F$

Figure S86. Compound **78** ^{1}H NMR spectrum (top), ^{13}C NMR spectrum (middle), high-resolution mass spectrum (bottom).

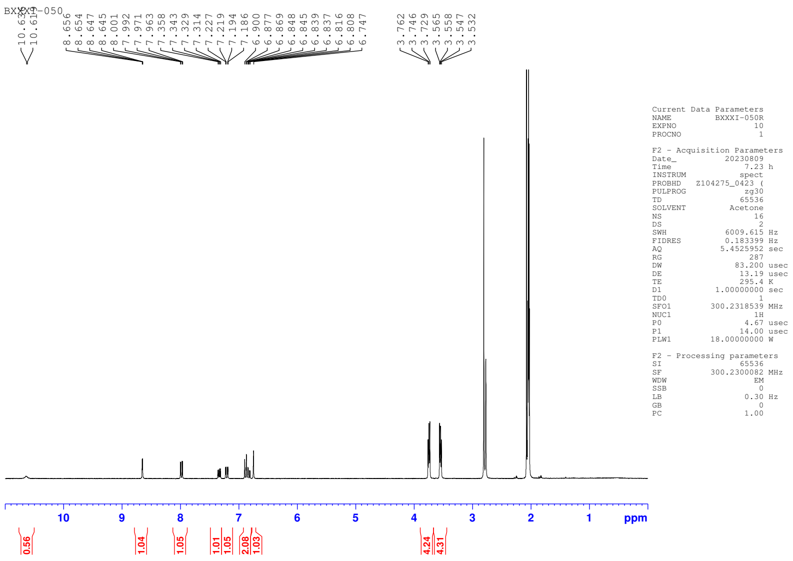

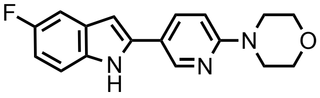

79

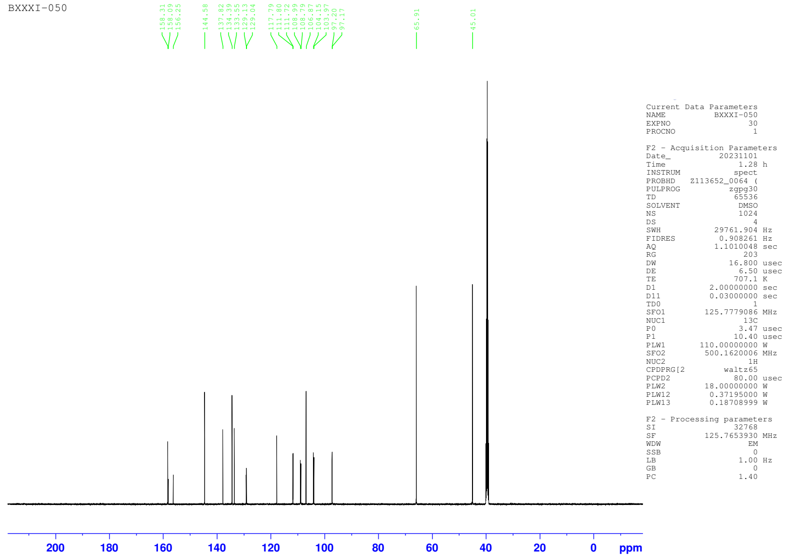

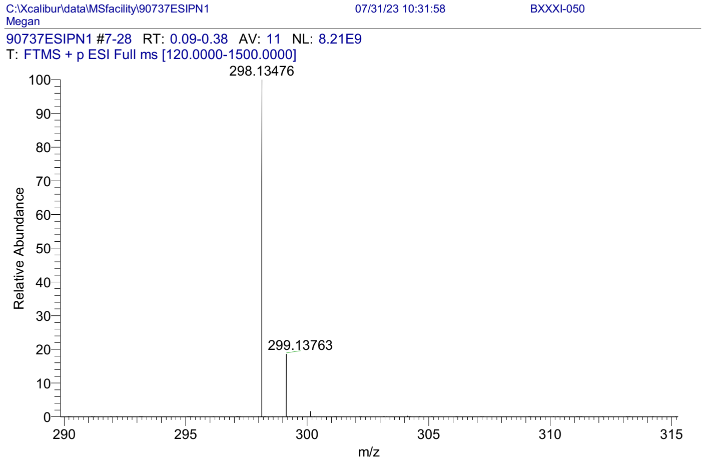

90737ESIPN1#7-27 RT: 0.09-0.38 AV: 11
T: FTMS + p ESI Full ms [120.0000-1500.0000]
m/z= 289.84109-315.24786

m/z	Intensity	Relative	Theo. Mass	Delta (ppm)	Composition
298.13476	8419191808.0	100.00	298.13502	-0.88	$C_{17}H_{17}ON_3F$

Figure S87. Compound **79** ^{1}H NMR spectrum (top), ^{13}C NMR spectrum (middle), high-resolution mass spectrum (bottom).

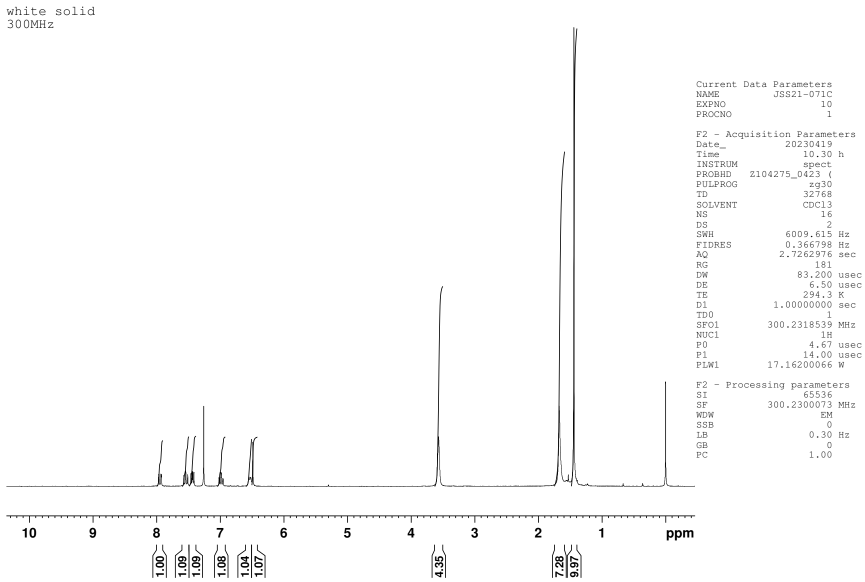

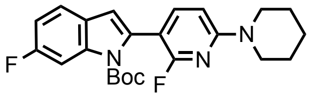

80

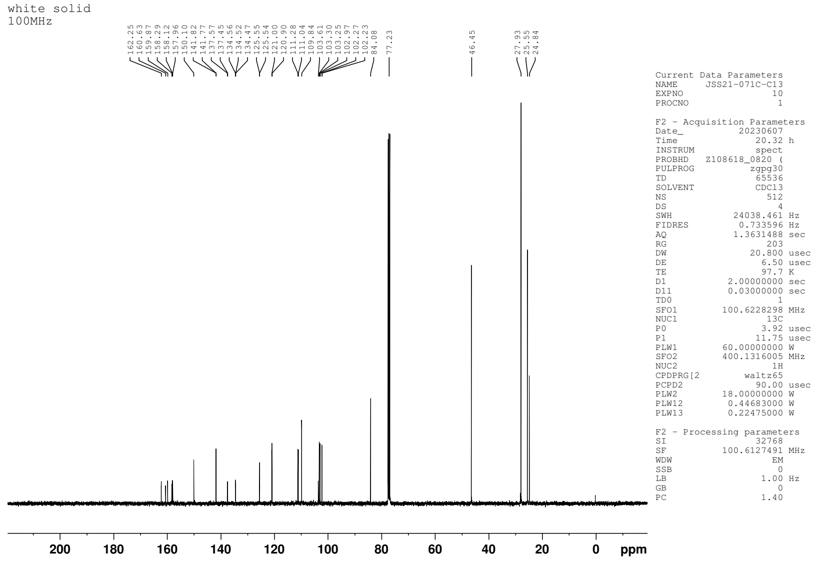

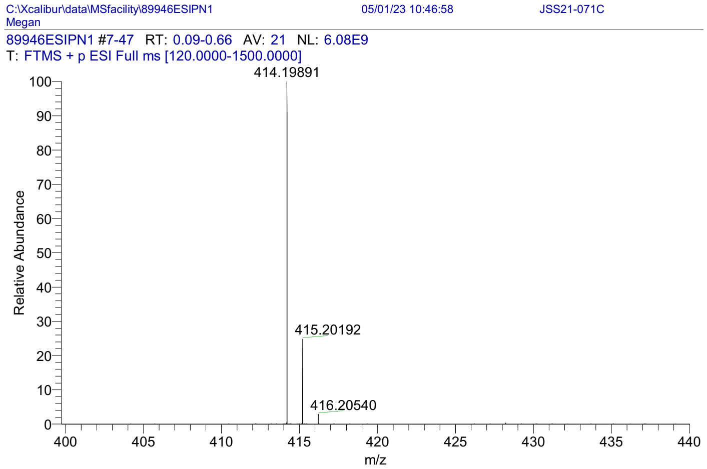

89946ESIPN1#7-47 RT: 0.09-0.66 AV: 21
T: FTMS + p ESI Full ms [120.0000-1500.0000]
m/z= 399.72558-440.02136

m/z	Intensity	Relative	Theo. Mass	Delta (ppm)	Composition
414.19891	6151417344.0	100.00	414.19876	0.37	$C_{23}H_{26}O_2N_3F_2$

Figure S88. Compound **80** ^{1}H NMR spectrum (top), ^{13}C NMR spectrum (middle), and high-resolution mass spectrum (bottom).

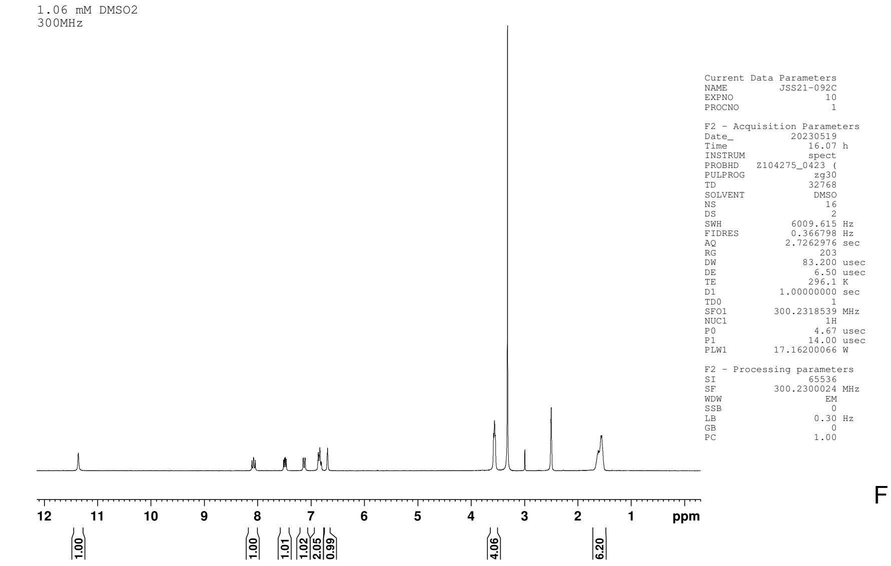

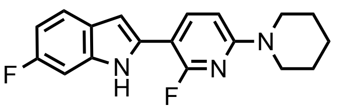

81

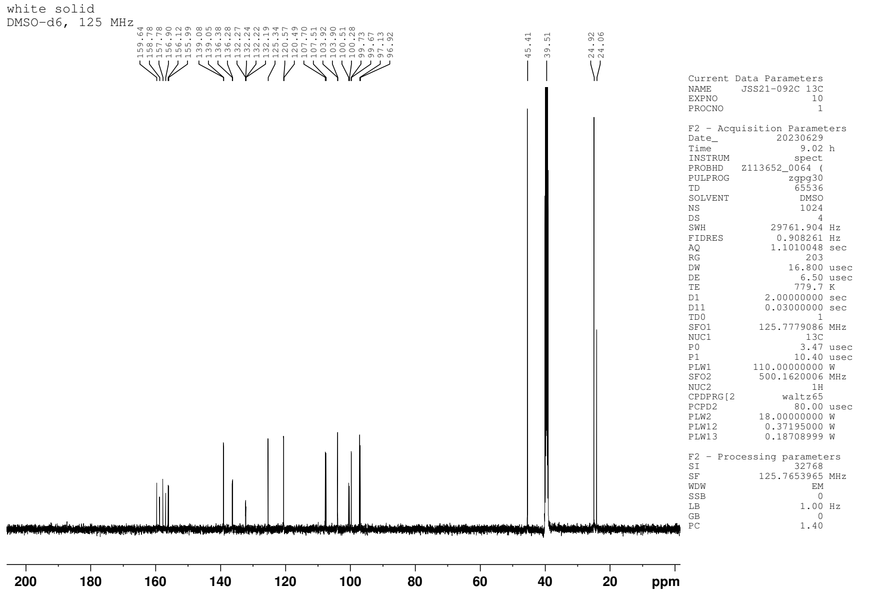

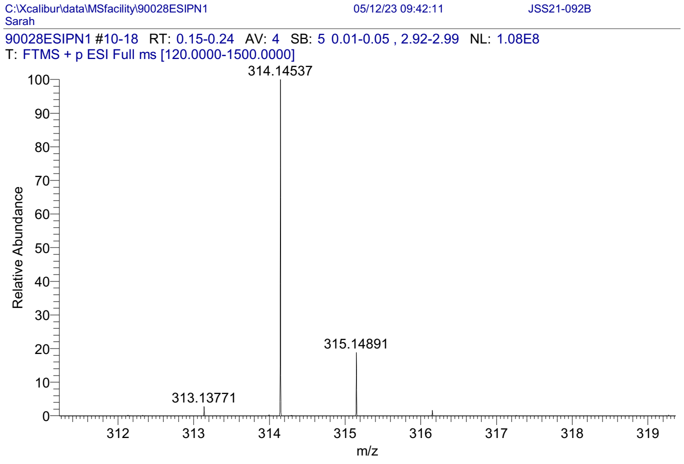

90028ESIPN1#11-17 RT: 0.15-0.24 AV: 4
SB: 5 0.01-0.05 , 2.92-2.99
T: FTMS + p ESI Full ms [120.0000-1500.0000]

m/z	Intensity	Relative	Theo. Mass	Delta (ppm)	Composition
314.14537	109846352.0	100.00	314.14633	-3.06	$C_{18}H_{18}N_3F_2$

Figure S89. Compound **81** ^{1}H NMR spectrum (top), ^{13}C NMR spectrum (middle), and high-resolution mass spectrum (bottom).

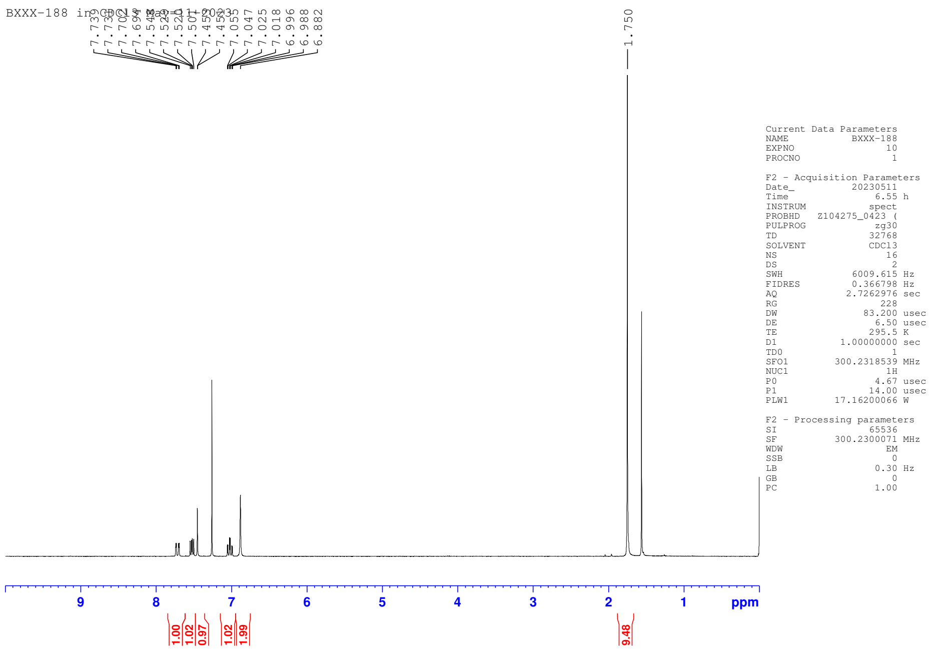

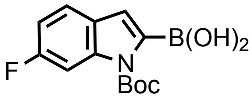

82

Figure S90. Compound **82** ^{1}H NMR spectrum.
Note: compound **82** decomposes in the mass spectrometer under both positive ion mode and negative ion mode.

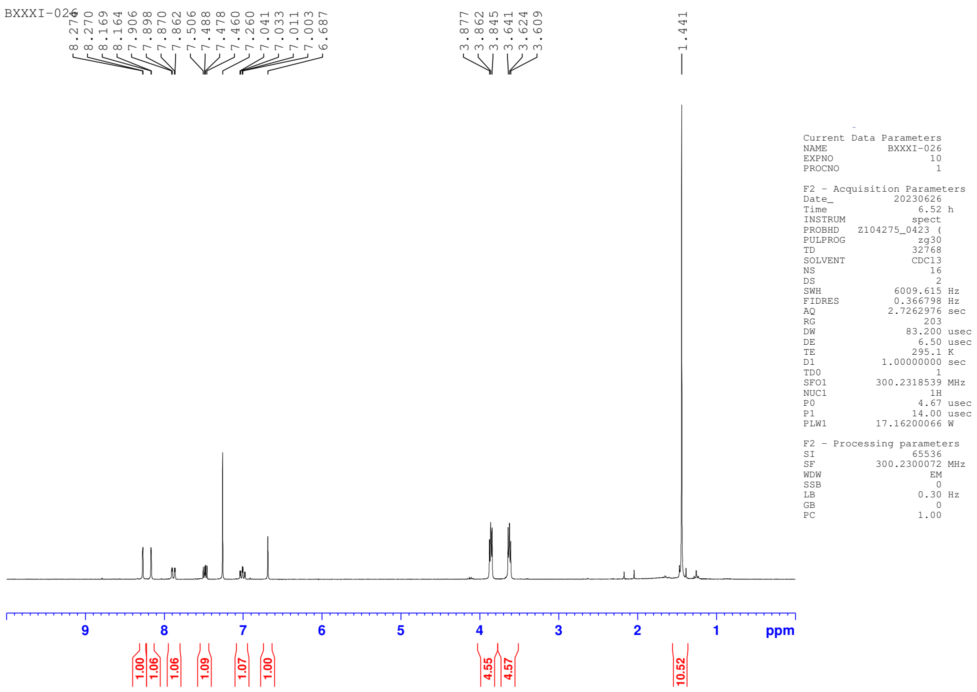

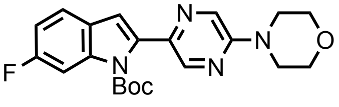

83

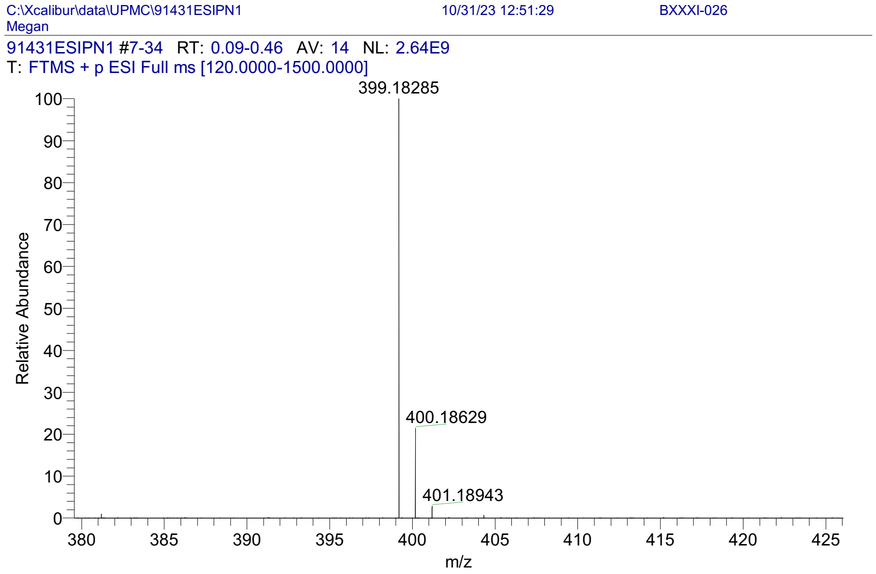

91431ESIPN1#7-33 RT: 0.09-0.46 AV: 14
T: FTMS + p ESI Full ms [120.0000-1500.0000]
m/z= 379.54296-426.04479

m/z	Intensity	Relative	Theo. Mass	Delta (ppm)	Composition
399.18285	2647502592.0	100.00	399.18270	0.38	$C_{21}H_{24}O_3N_4F$

Figure S91. Compound **83** ¹H NMR spectrum (top) and high-resolution mass spectrum (bottom).

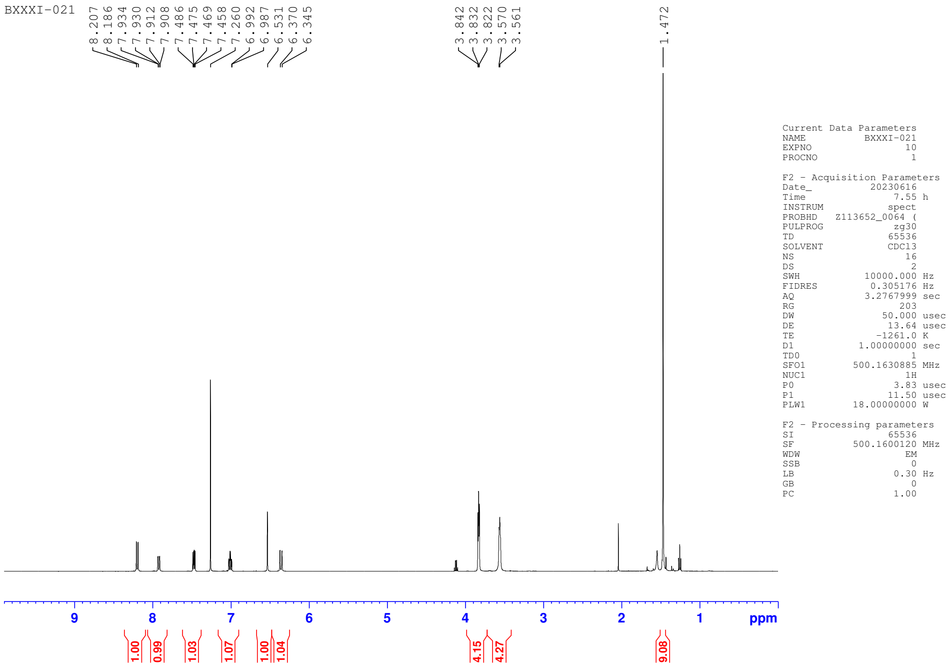

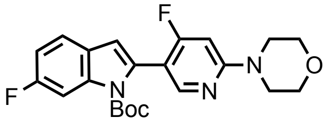

84

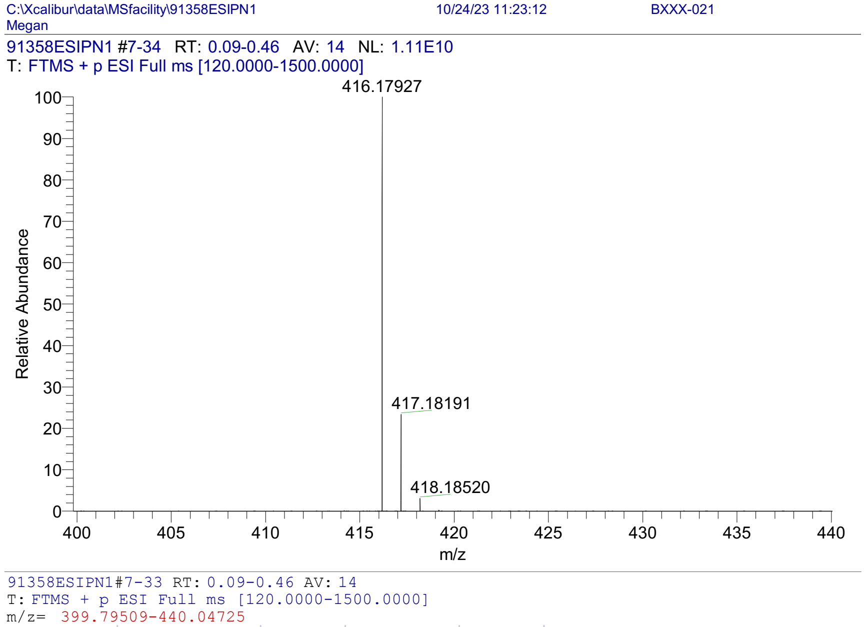

m/z	Intensity	Relative	Theo. Mass	Delta (ppm)	Composition
416.17927	11222353920.0	100.00	416.17802	2.98	$C_{22}H_{24}O_3N_3F_2$

Figure S92. Compound **84** ^{1}H NMR spectrum (top) and high-resolution mass spectrum (bottom).

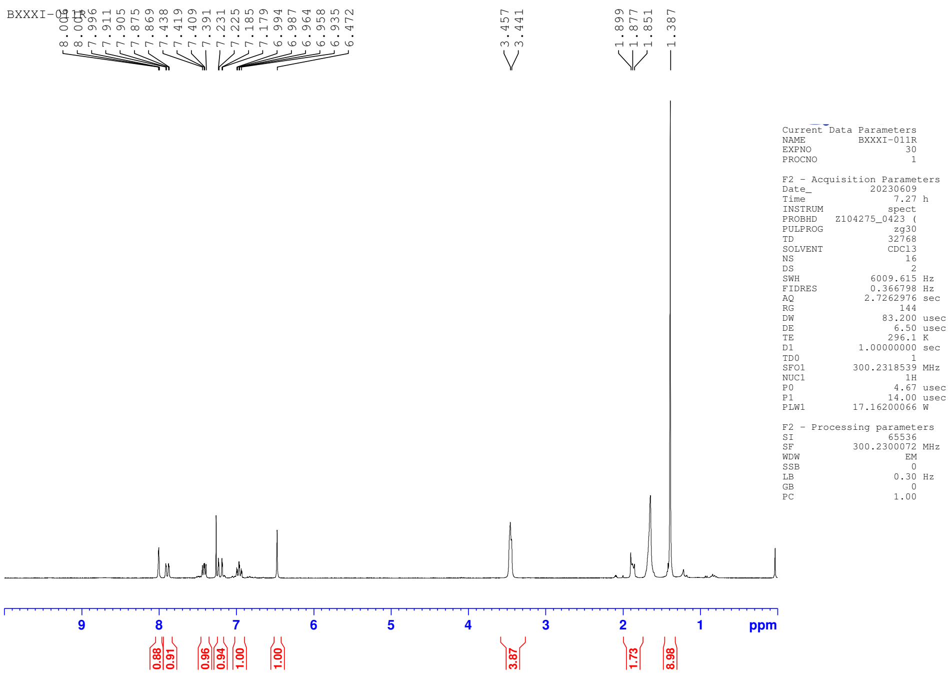

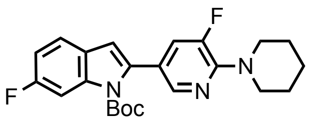

85

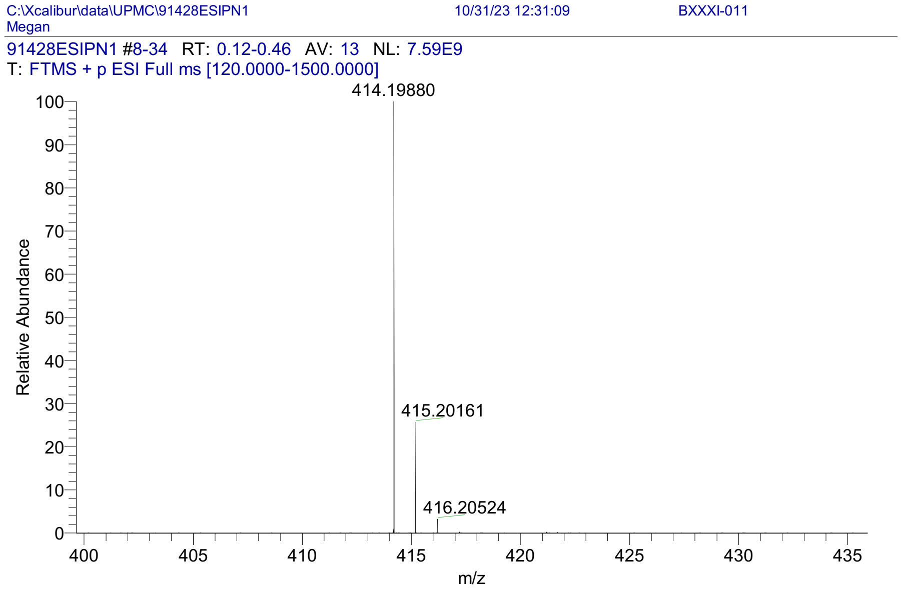

91428ESIPN1#9-33 RT: 0.12-0.46 AV: 13
T: FTMS + p ESI Full ms [120.0000-1500.0000]
m/z= 399.63229-435.90400

m/z	Intensity	Relative	Theo. Mass	Delta (ppm)	Composition
414.19880	7860680192.0	100.00	414.19876	0.10	$C_{23}H_{26}O_2N_3F_2$

Figure S93. Compound **85** ^{1}H NMR spectrum (top) and high-resolution mass spectrum (bottom).

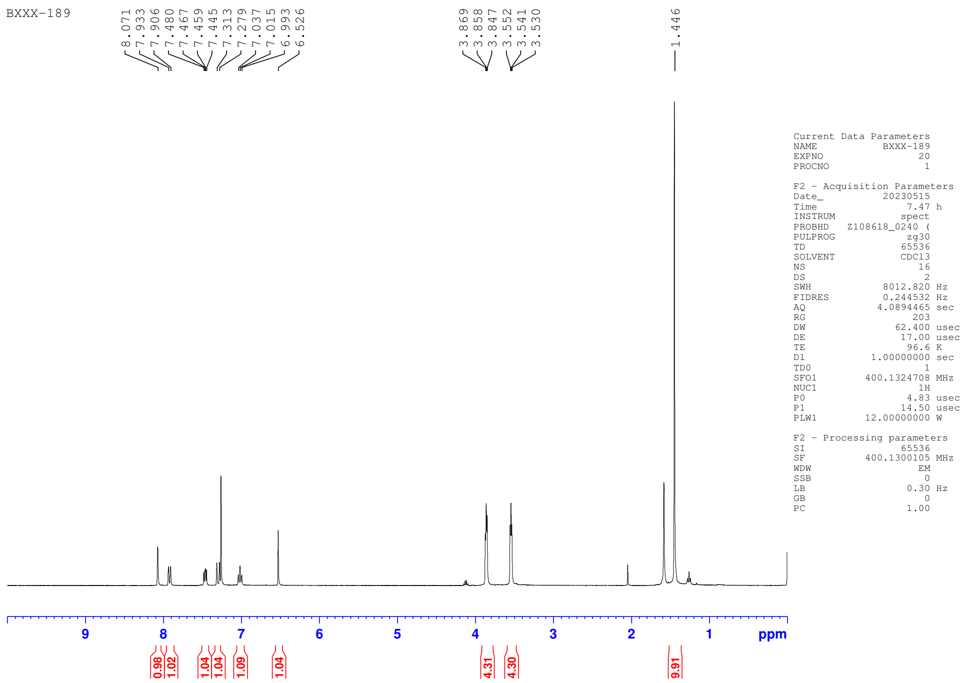

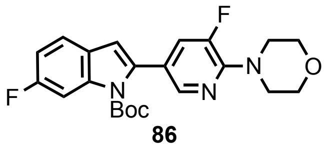

86

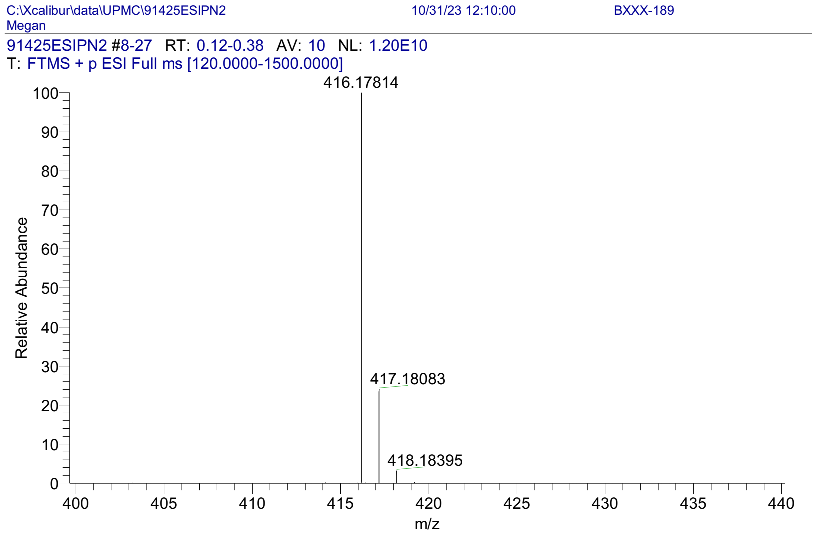

91425ESIPN2#9-27 RT: 0.12-0.38 AV: 10
T: FTMS + p ESI Full ms [120.0000-1500.0000]
m/z= 399.63702-440.18214

m/z	Intensity	Relative	Theo. Mass	Delta (ppm)	Composition
416.17814	12348234752.0	100.00	416.17802	0.28	$C_{22}H_{24}O_3N_3F_2$

Figure S94. Compound **86** ^{1}H NMR spectrum (top) and high-resolution mass spectrum (bottom).

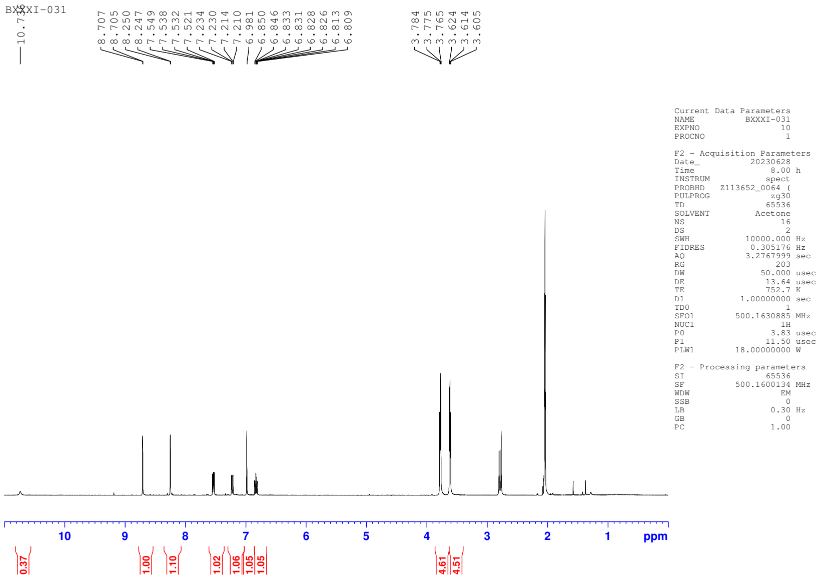

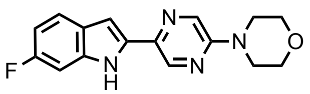

87

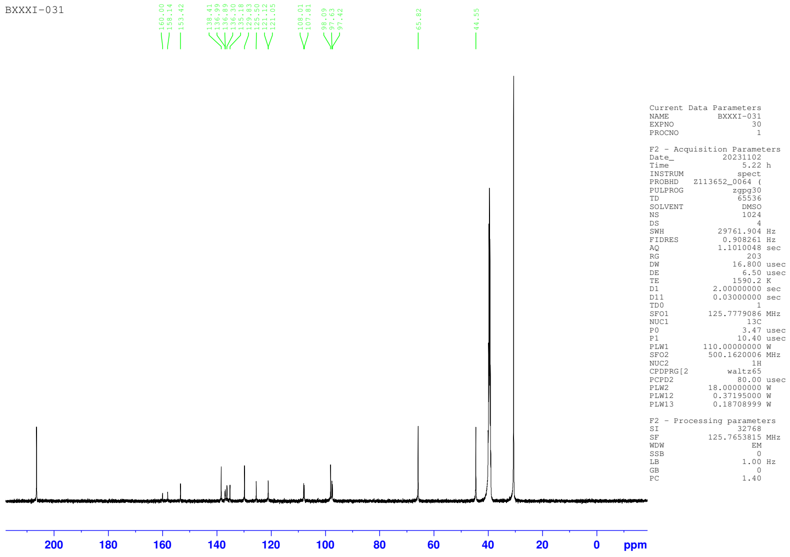

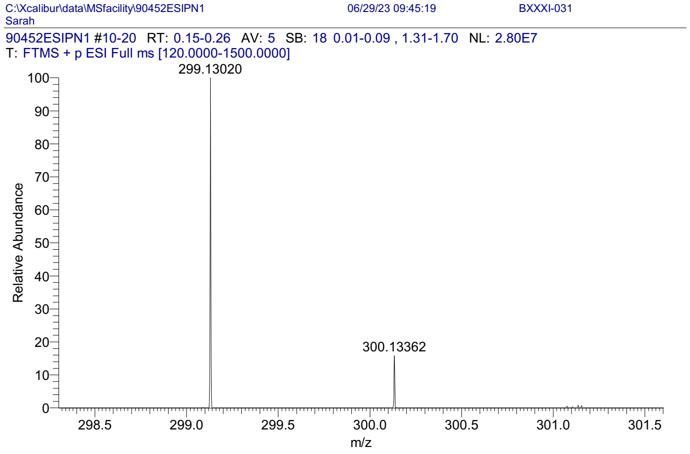

90452ESIPN1#11-19 RT: 0.15-0.26 AV: 5
SB: 18 0.01-0.09 , 1.31-1.70
T: FTMS + p ESI Full ms [120.0000-1500.0000]
m/z= 298.30190-301.60165

m/z	Intensity	Relative	Theo. Mass	Delta (ppm)	Composition
299.13020	28102602.0	100.00	299.13027	-0.22	$C_{16}H_{16}ON_4F$

Figure S95. Compound **87** ^{1}H NMR spectrum (top), ^{13}C NMR spectrum (middle), high-resolution mass spectrum (bottom).

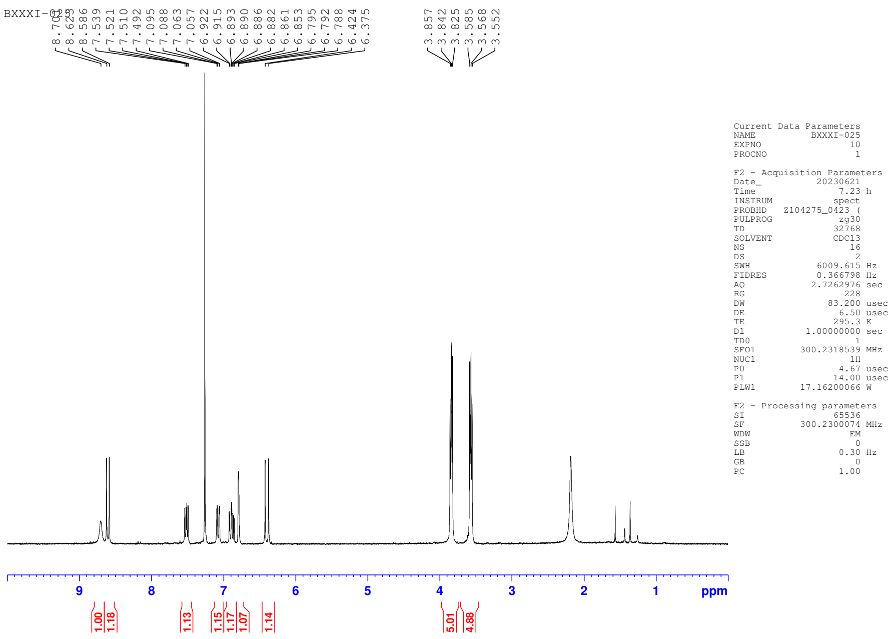

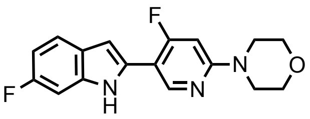

88

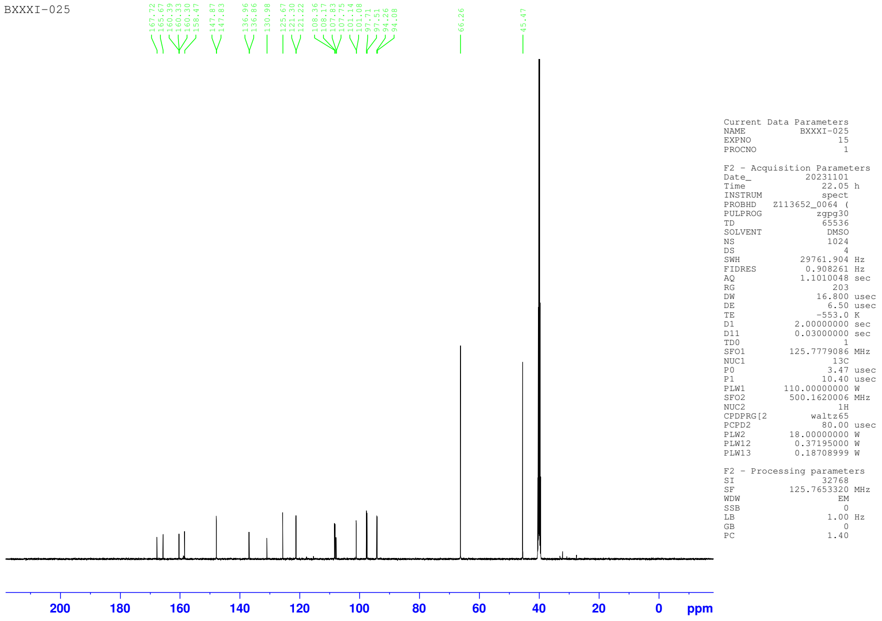

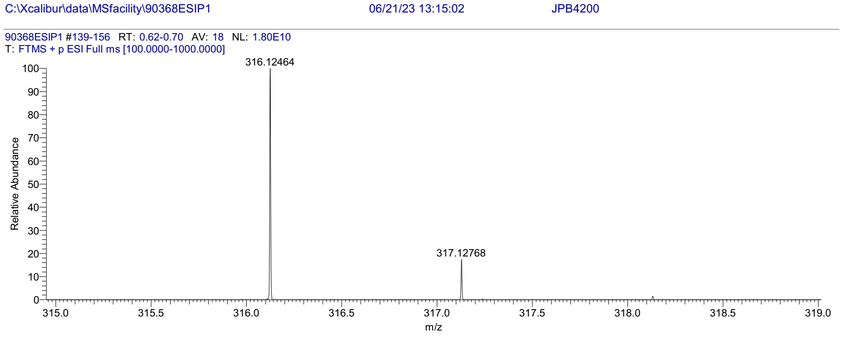

90368ESIP1#139-156 RT: 0.62-0.70 AV: 18
T: FTMS + p ESI Full ms [100.0000-1000.0000]
m/z= 314.94965-319.01508

m/z	Intensity	Relative	Theo. Mass	Delta (ppm)	Composition
316.12464	18333968384.0	100.00	316.12560	-3.01	$C_{17}H_{16}ON_3F_2$

Figure S96. Compound **88** ^{1}H NMR spectrum (top), ^{13}C NMR spectrum (middle), high-resolution mass spectrum (bottom).

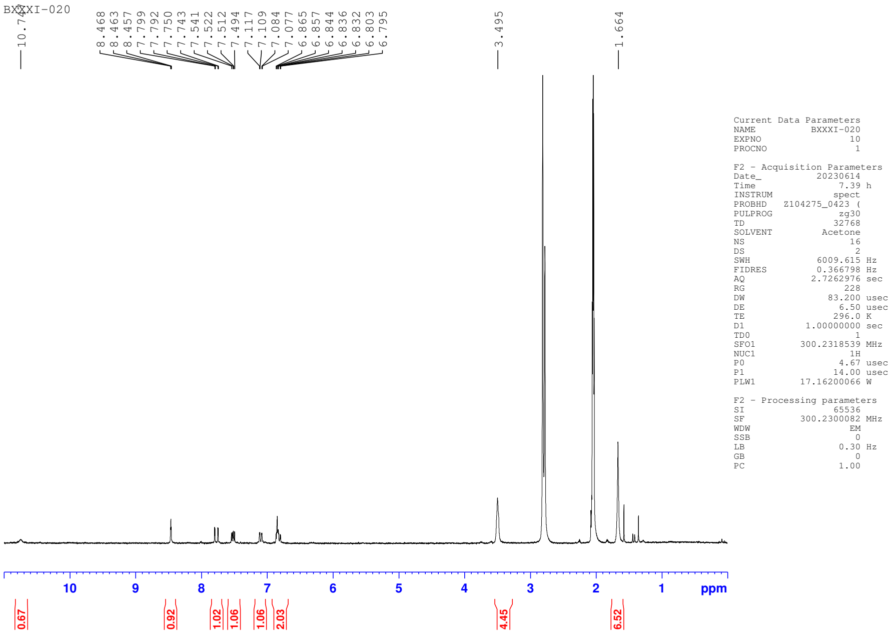

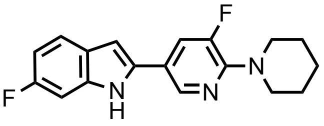

89

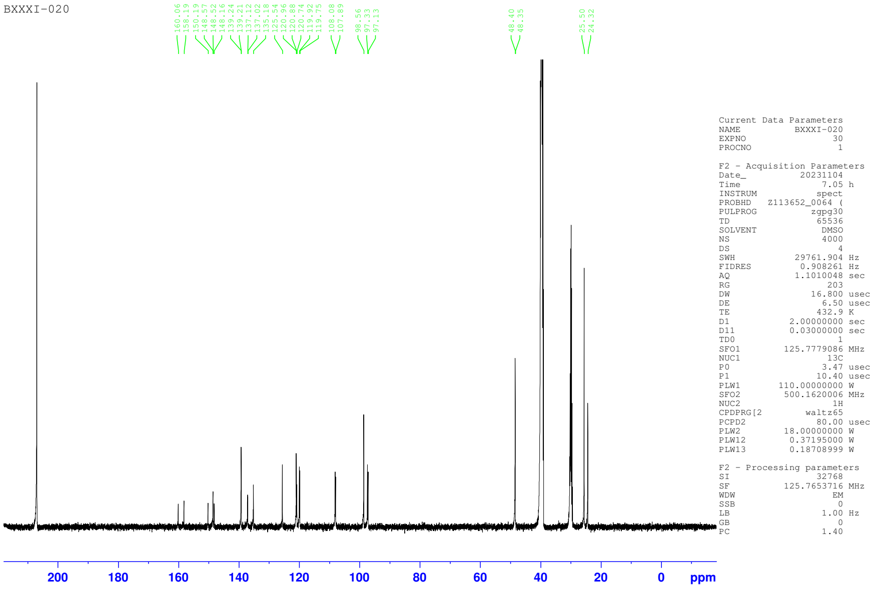

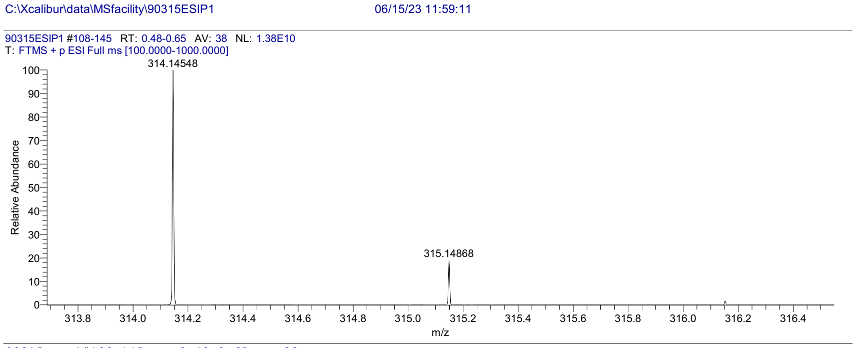

90315ESIP1#108-145 RT: 0.48-0.65 AV: 38
T: FTMS + p ESI Full ms [100.0000-1000.0000]
m/z= 313.68730-316.54781

m/z	Intensity	Relative	Theo. Mass	Delta (ppm)	Composition
314.14548	13788840960.0	100.00	314.14633	-2.71	$C_{18}H_{18}N_3F_2$

Figure S97. Compound **89** ^{1}H NMR spectrum (top), ^{13}C NMR spectrum (middle), high-resolution mass spectrum (bottom).

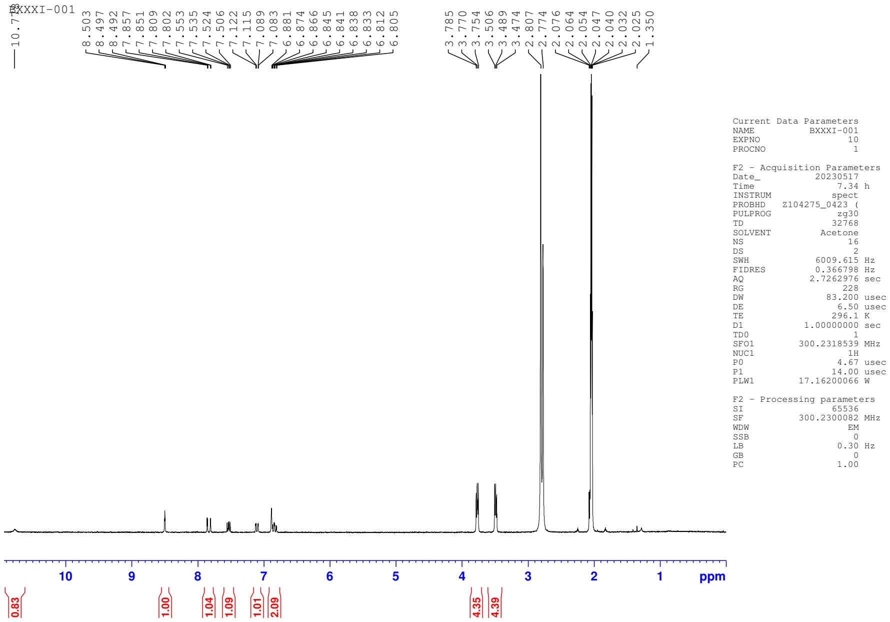

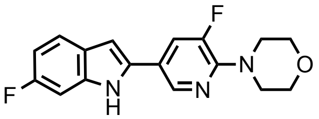

90

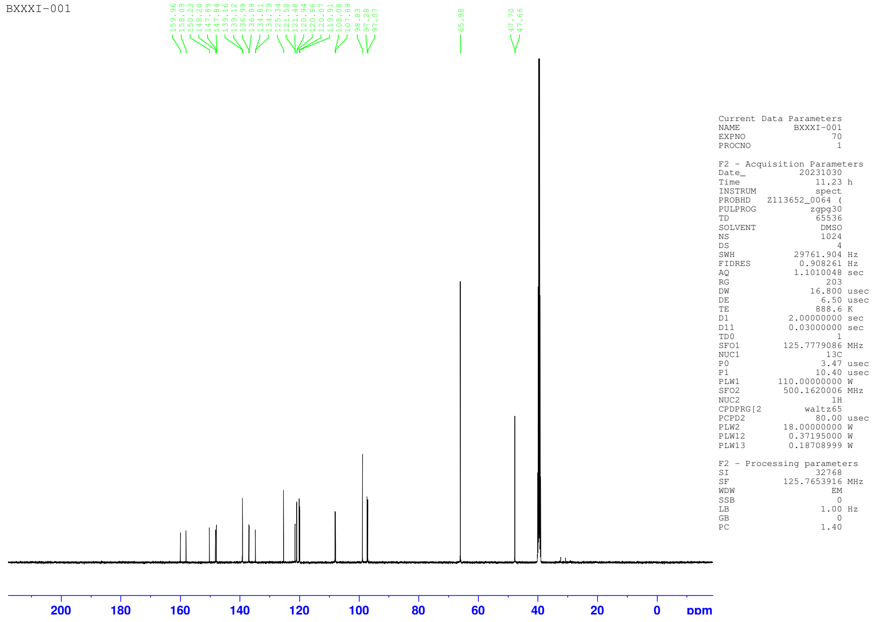

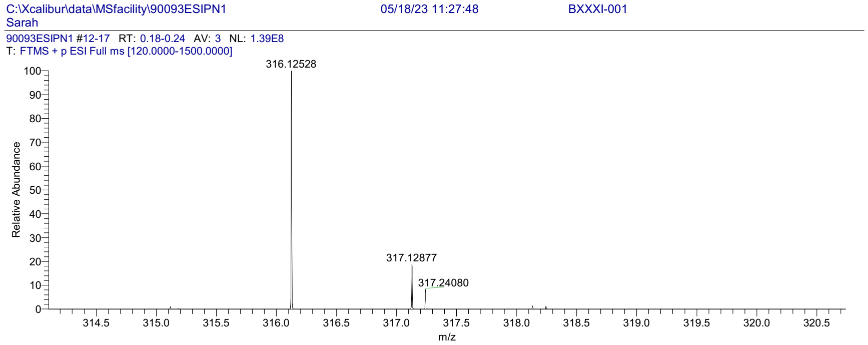

90093ESIPN1#13-17 RT: 0.18-0.24 AV: 3
T: FTMS + p ESI Full ms [120.0000-1500.0000]
m/z= 314.10253-320.73941

m/z	Intensity	Relative	Theo. Mass	Delta (ppm)	Composition
316.12528	139615936.0	100.00	316.12560	-0.98	$C_{17}H_{16}ON_3F_2$

Figure S98. Compound **90** ^{1}H NMR spectrum (top), ^{13}C NMR spectrum (middle), high-resolution mass spectrum (bottom).

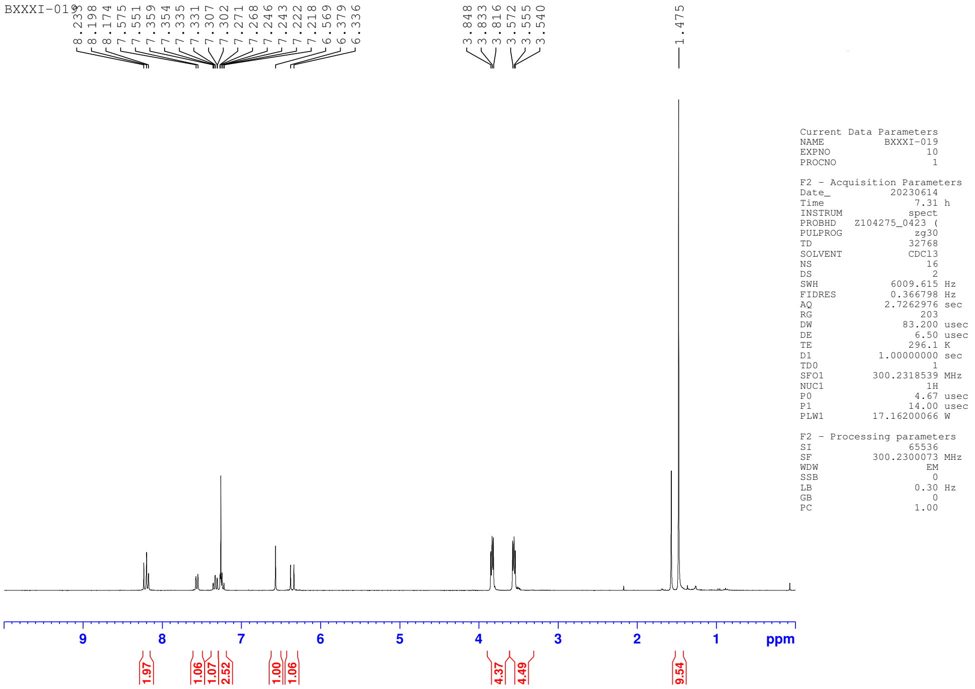

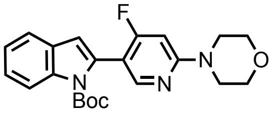

91

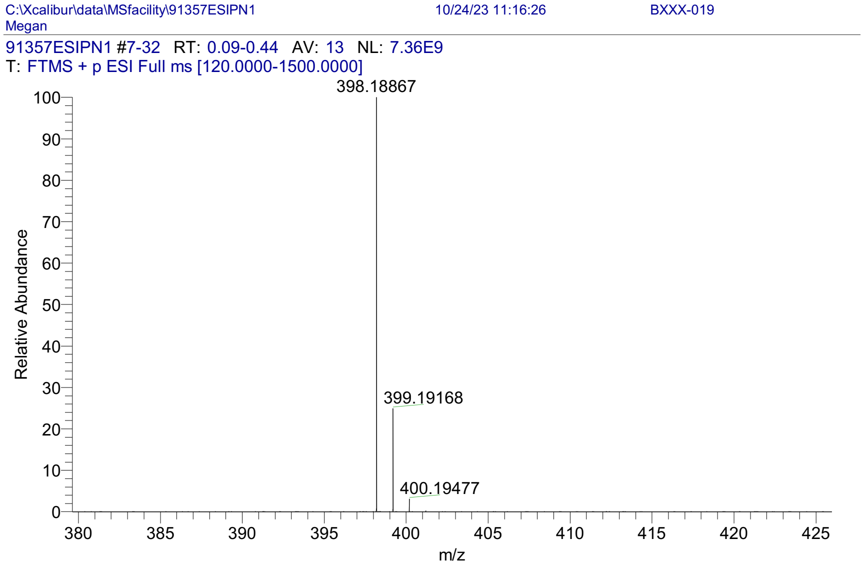

91357ESIPN1#7-31 RT: 0.09-0.44 AV: 13
T: FTMS + p ESI Full ms [120.0000-1500.0000]
m/z= 379.62237-425.96293

m/z	Intensity	Relative	Theo. Mass	Delta (ppm)	Composition
398.18867	7587728384.0	100.00	398.18745	3.06	$C_{22}H_{25}O_3N_3F$

Figure S99. Compound **91** ^{1}H NMR spectrum (top) and high-resolution mass spectrum (bottom).

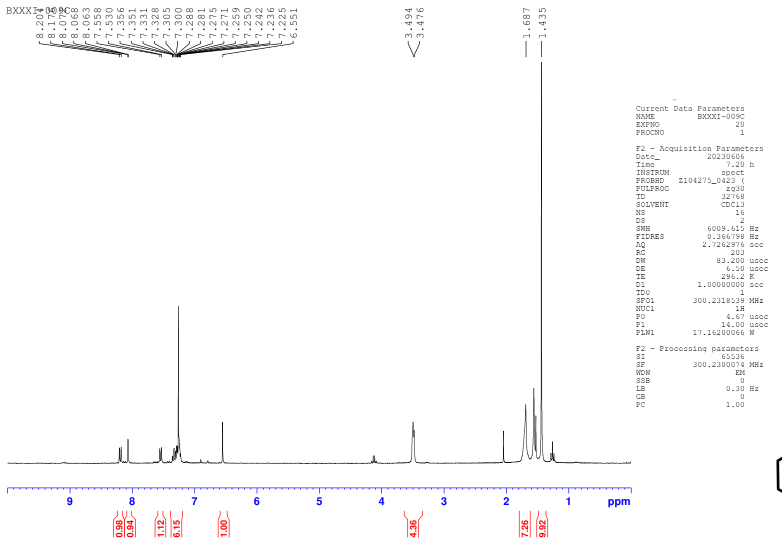

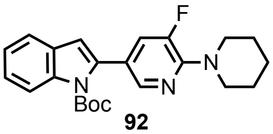

92

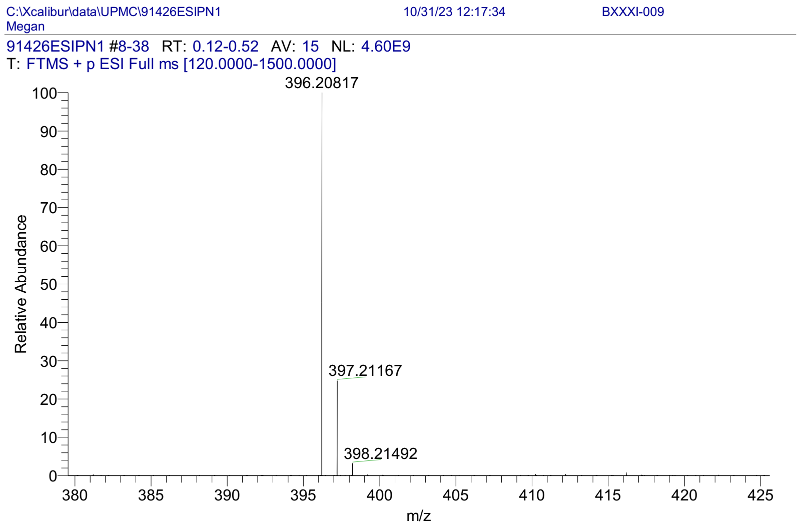

91426ESIPN1#9-37 RT: 0.12-0.52 AV: 15
T: FTMS + p ESI Full ms [120.0000-1500.0000]
m/z= 379.54863-425.58415

m/z	Intensity	Relative	Theo. Mass	Delta (ppm)	Composition
396.20817	4607591424.0	100.00	396.20818	-0.02	$C_{23}H_{27}O_2N_3F$

Figure S100. Compound **92** ^{1}H NMR spectrum (top) and high-resolution mass spectrum (bottom).

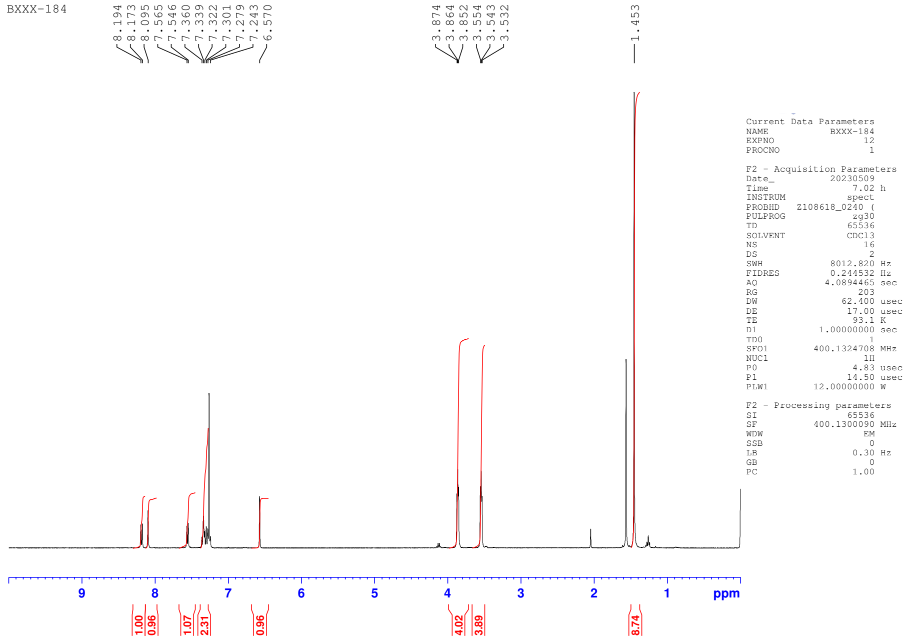

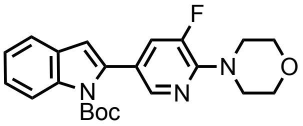

93

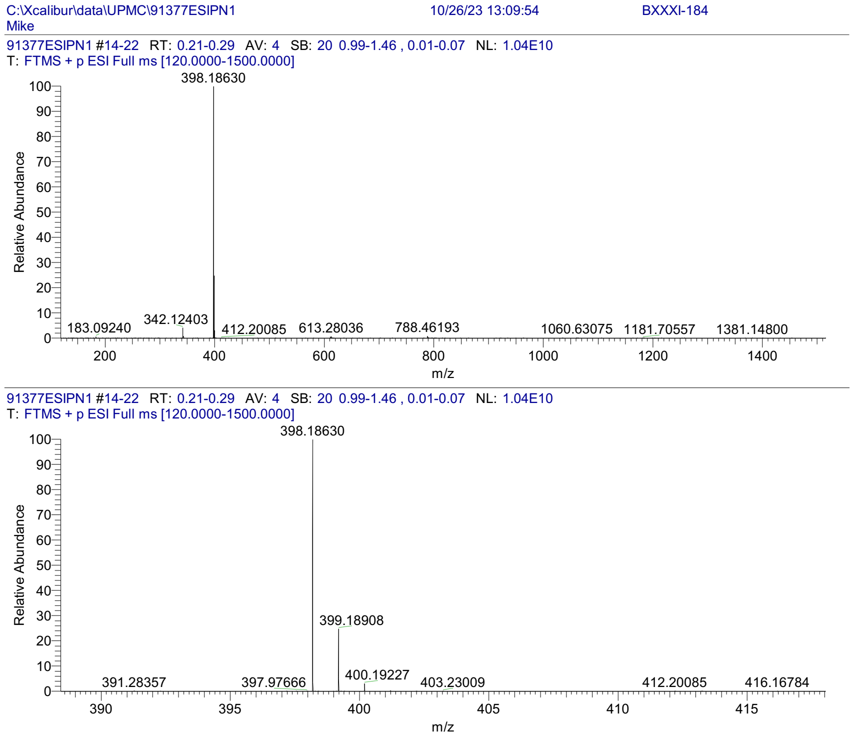

91377ESIPN1#15-21 RT: 0.21-0.29 AV: 4
SB: 20 0.99-1.46 , 0.01-0.07
T: FTMS + p ESI Full ms [120.0000-1500.0000]
m/z= 388.43424-418.02747

m/z	Intensity	Relative	Theo. Mass	Delta (ppm)	Composition
398.18630	10530295808.0	100.00	398.18745	-2.89	$C_{22}H_{25}O_3N_3F$

Figure S101. Compound **93** ^{1}H NMR spectrum (top) and high-resolution mass spectrum (bottom).

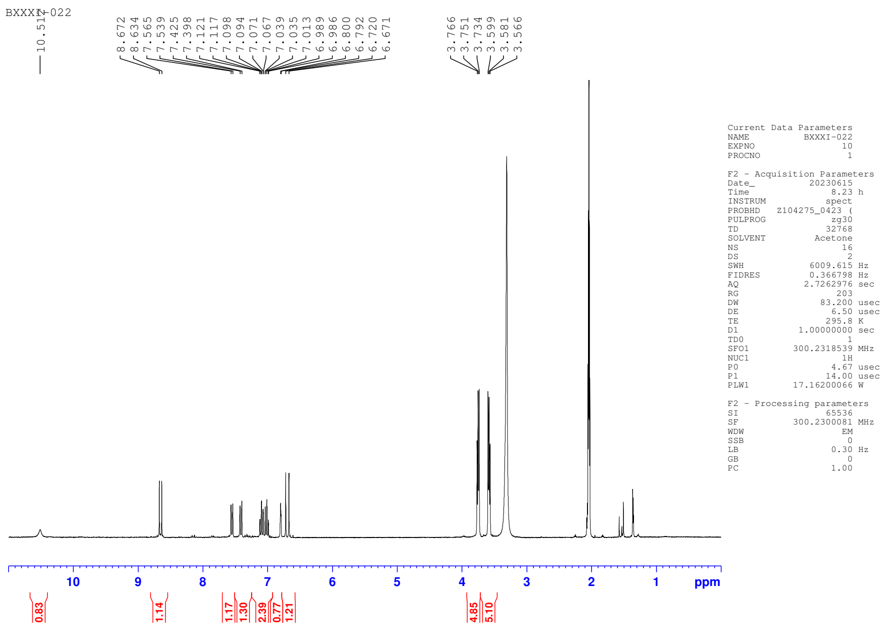

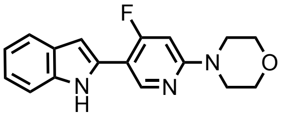

94

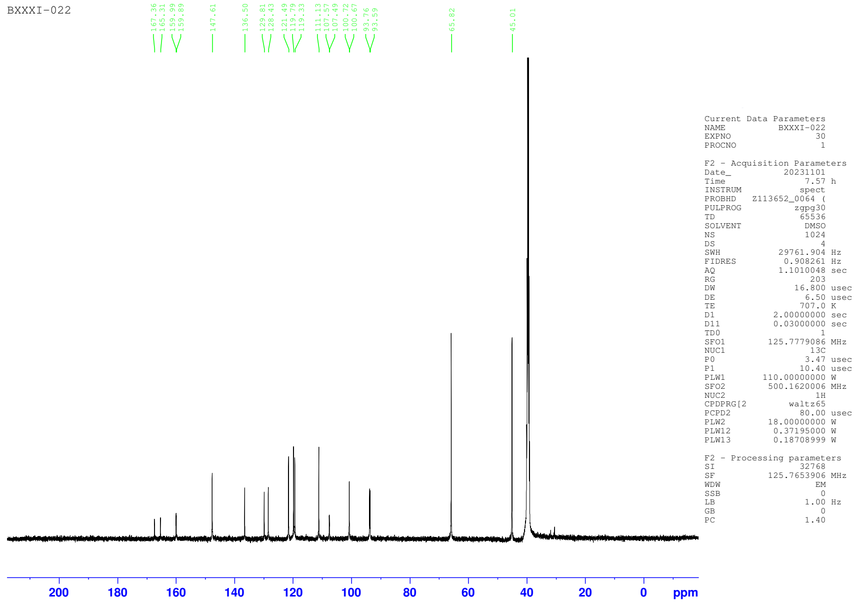

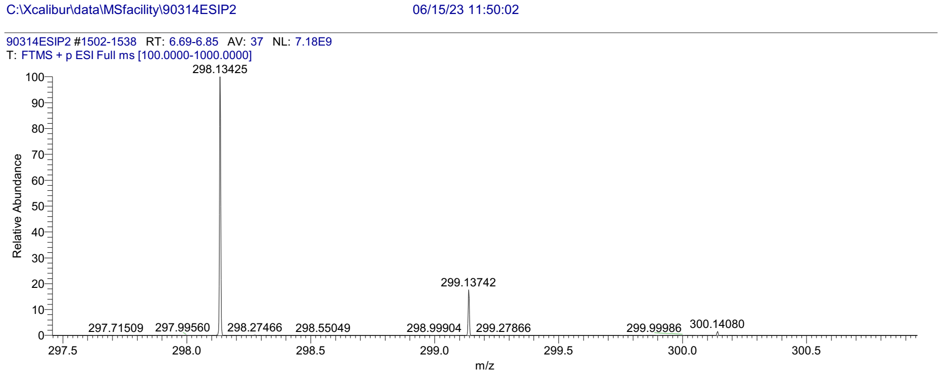

90314ESIP2#1502-1538 RT: 6.69-6.85 AV: 37
T: FTMS + p ESI Full ms [100.0000-1000.0000]

m/z	Intensity	Relative	Theo. Mass	Delta (ppm)	Composition
298.13425	7186618880.0	100.00	298.13502	-2.57	$C_{17}H_{17}ON_3F$

Figure S102. Compound **94** ^{1}H NMR spectrum (top), ^{13}C NMR spectrum (middle), high-resolution mass spectrum (bottom).

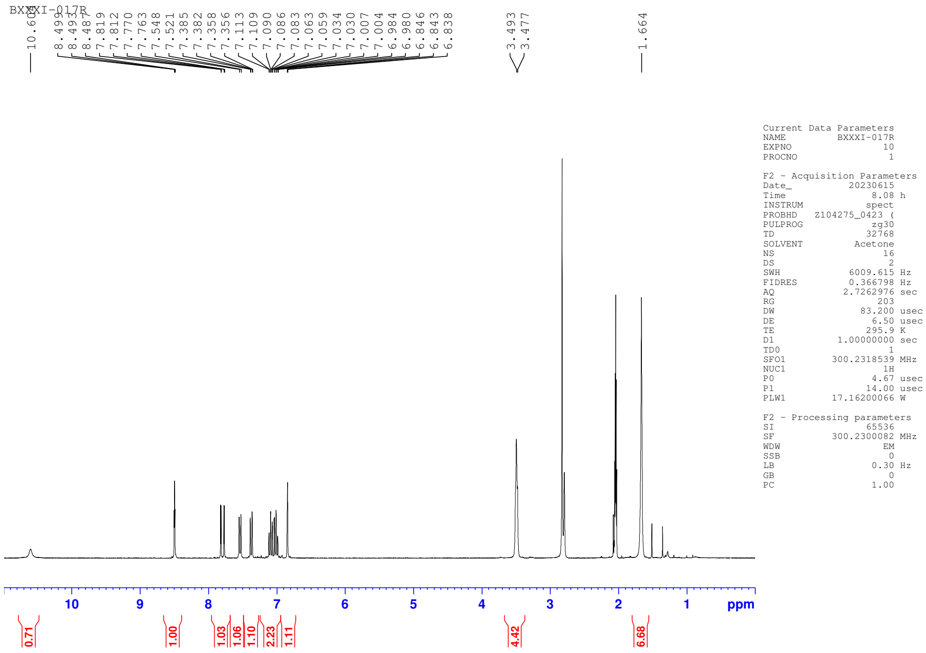

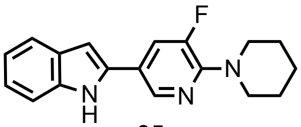

95

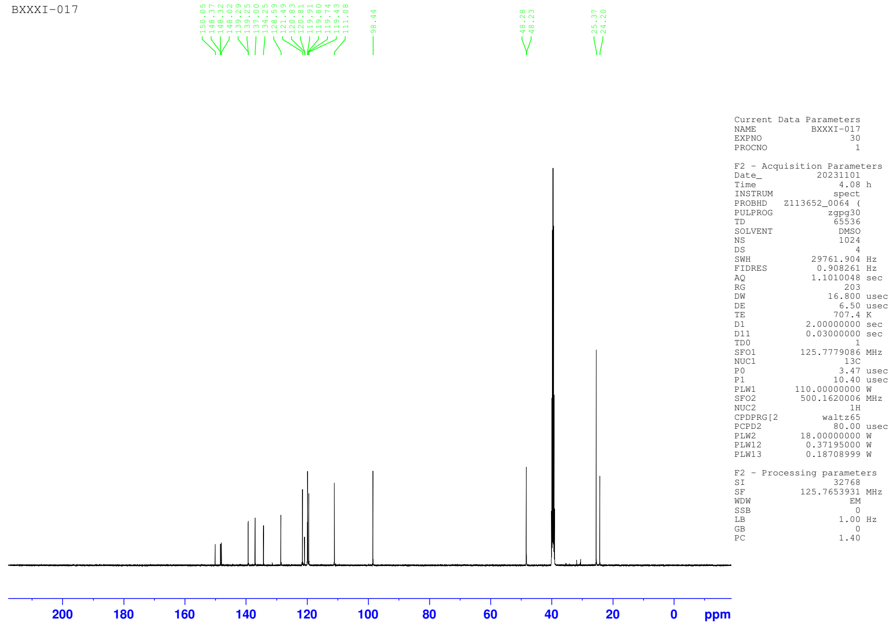

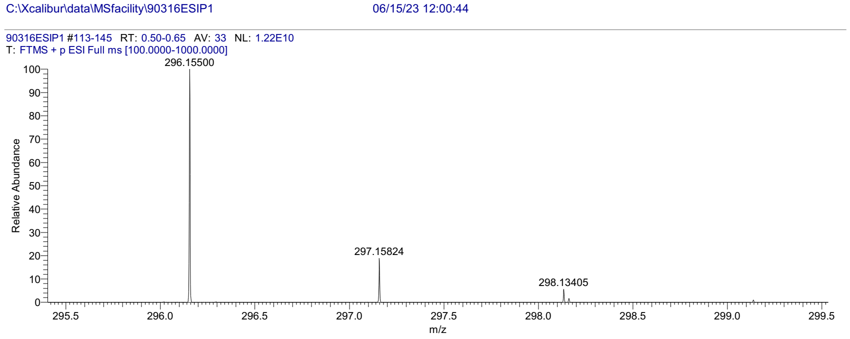

90316ESIP1#113-145 RT: 0.50-0.65 AV: 33
T: FTMS + p ESI Full ms [100.0000-1000.0000]
m/z= 295.40348-299.53248

m/z	Intensity	Relative	Theo. Mass	Delta (ppm)	Composition
296.15500	12217956352.0	100.00	296.15575	-2.54	$C_{18} H_{19} N_3 F$

Figure S103. Compound **95** ^{1}H NMR spectrum (top), ^{13}C NMR spectrum (middle), high-resolution mass spectrum (bottom).

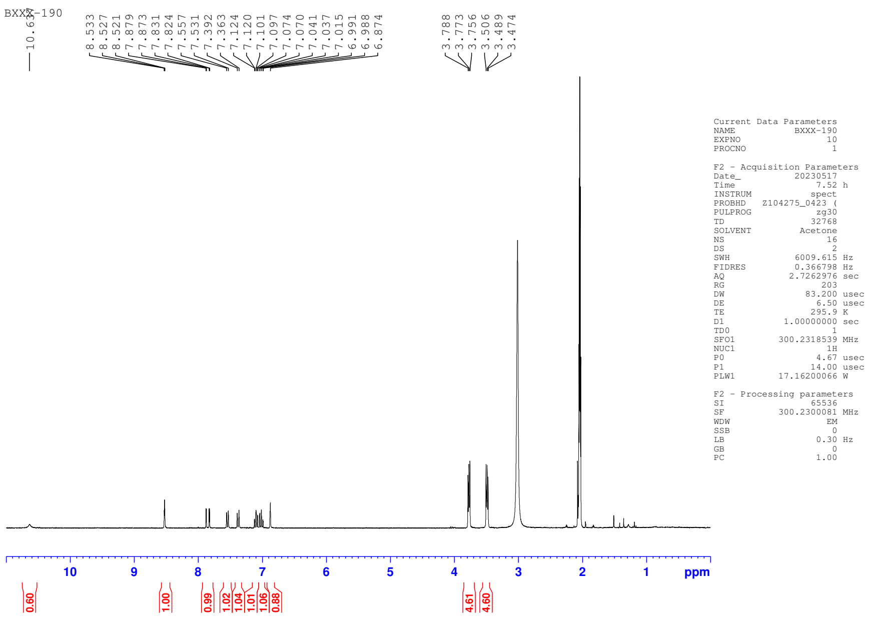

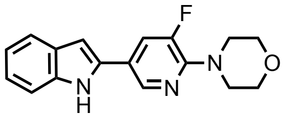

96

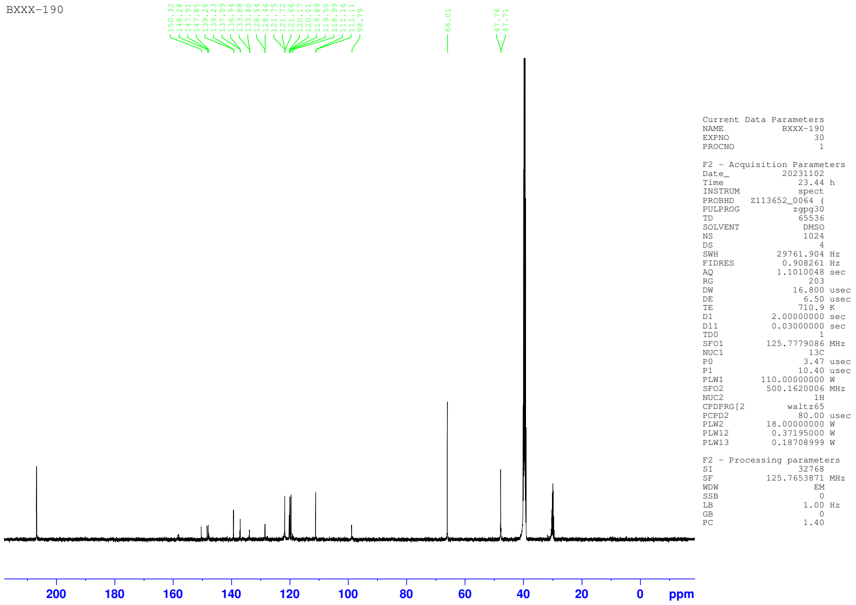

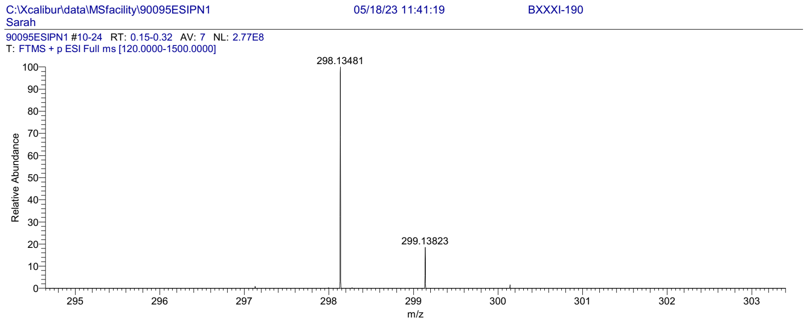

90095ESIPN1#11-23 RT: 0.15-0.32 AV: 7
T: FTMS + p ESI Full ms [120.0000-1500.0000]
m/z= 294.64675-303.40083

m/z	Intensity	Relative	Theo. Mass	Delta (ppm)	Composition
298.13481	285786912.0	100.00	298.13502	-0.68	$C_{17}H_{17}ON_3F$

Figure S104. Compound **96** ^{1}H NMR spectrum (top), ^{13}C NMR spectrum (middle), high-resolution mass spectrum (bottom).

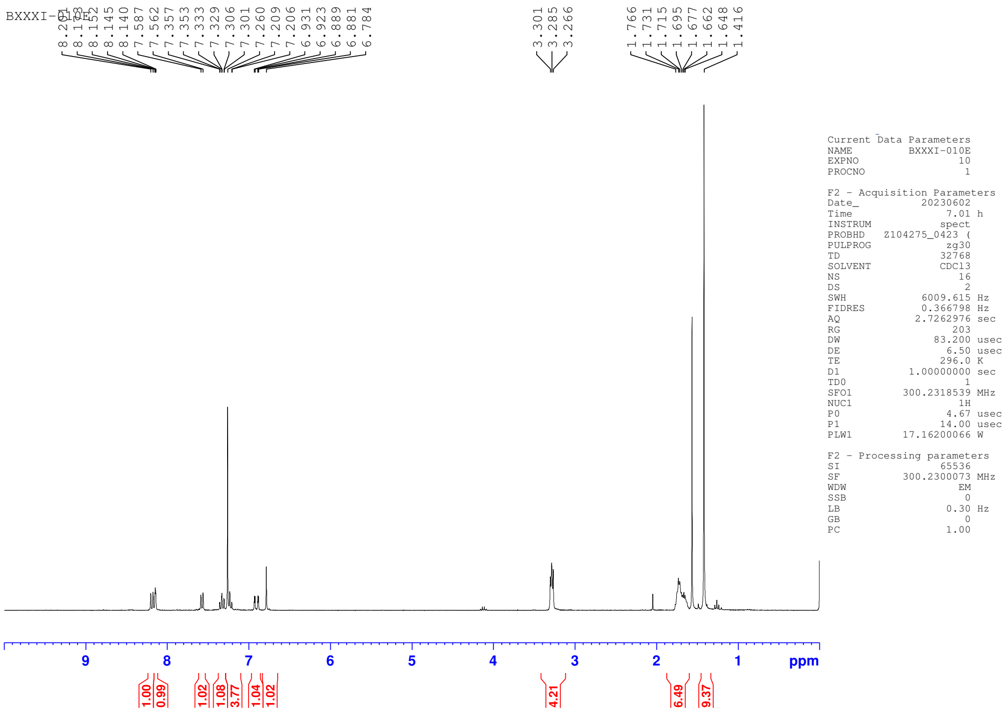

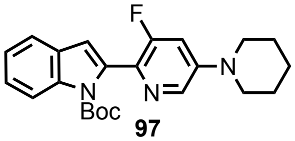

97

C:\Xcalibur\data\UPMC\91427ESIPN1 10/31/23 12:24:21 BXXXI-010
Megan

91427ESIPN1 #7-35 RT: 0.09-0.49 AV: 15 NL: 6.23E9
T: FTMS + p ESI Full ms [120.0000-1500.0000]

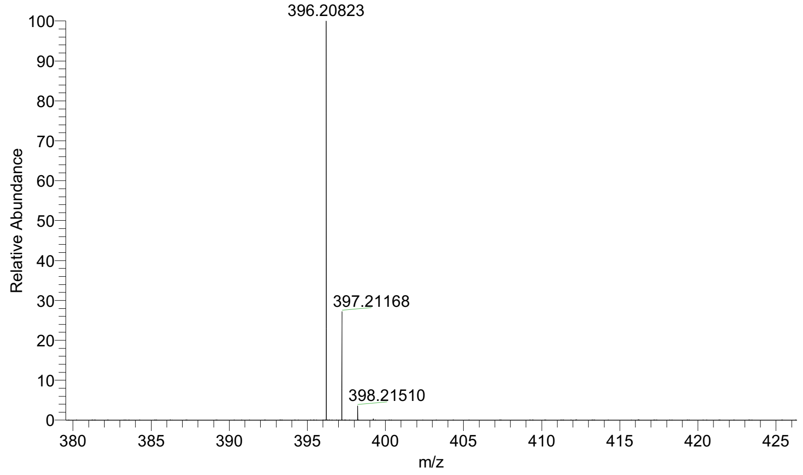

91427ESIPN1#7-35 RT: 0.09-0.49 AV: 15
T: FTMS + p ESI Full ms [120.0000-1500.0000]
m/z= 379.52615-426.33063

m/z	Intensity	Relative	Theo. Mass	Delta (ppm)	Composition
396.20823	6236114432.0	100.00	396.20818	0.11	$C_{23}H_{27}O_2N_3F$

Figure S105. Compound **97** ^1H NMR spectrum (top) and high-resolution mass spectrum (bottom).

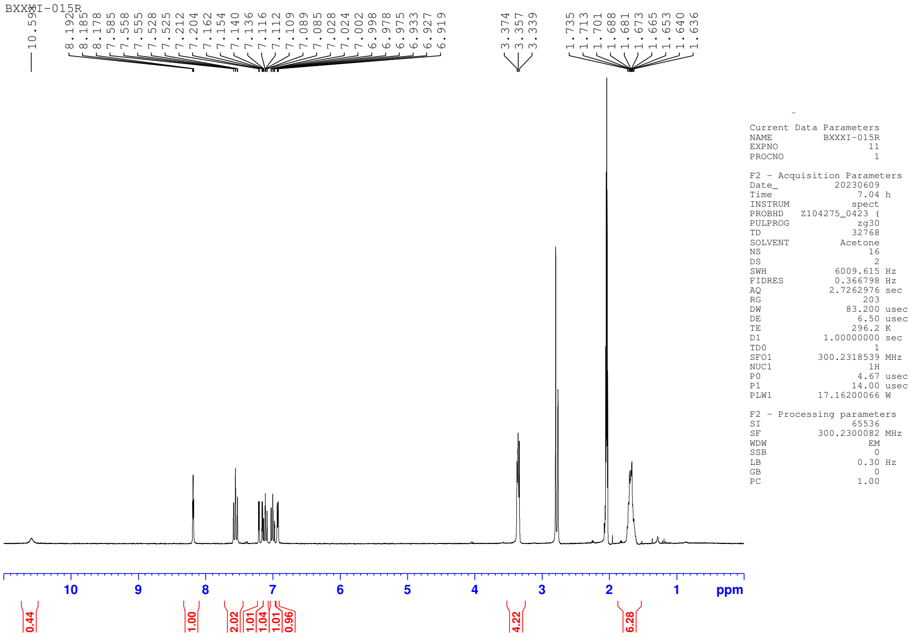

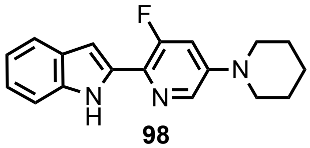

98

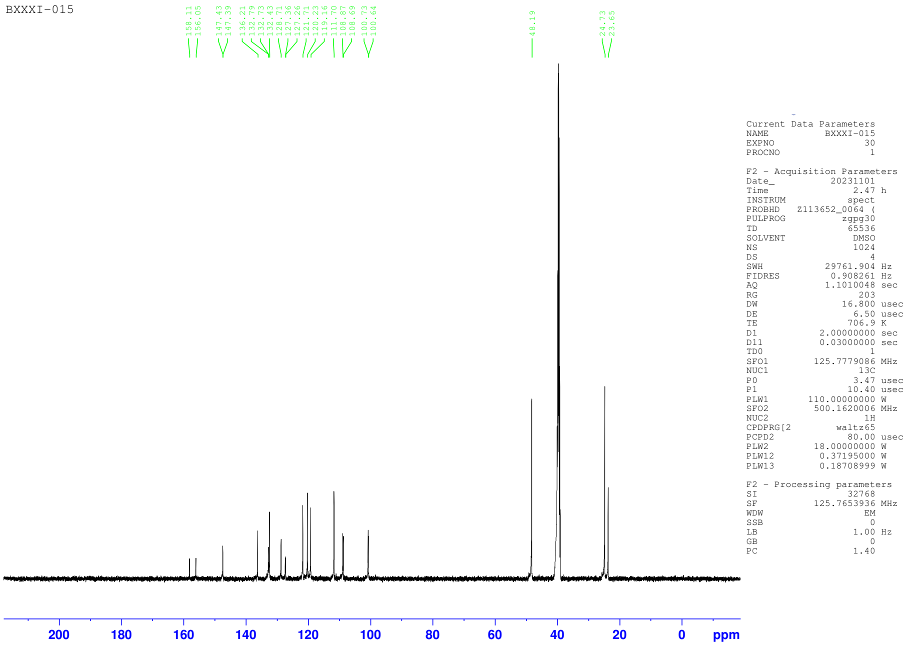

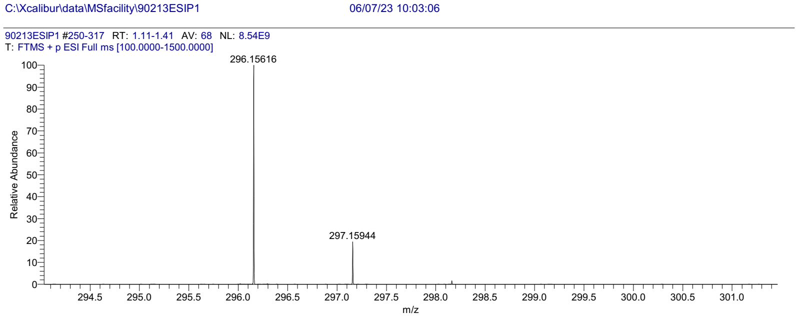

90213ESIP1#250-317 RT: 1.11-1.41 AV: 68
T: FTMS + p ESI Full ms [100.0000-1500.0000]
m/z= 294.03329-301.45948

m/z	Intensity	Relative	Theo. Mass	Delta (ppm)	Composition
296.15616	8649415680.0	100.00	296.15575	1.37	$C_{18}H_{19}N_3F$

Figure S106. Compound **98** ^{1}H NMR spectrum (top), ^{13}C NMR spectrum (middle), high-resolution mass spectrum (bottom).

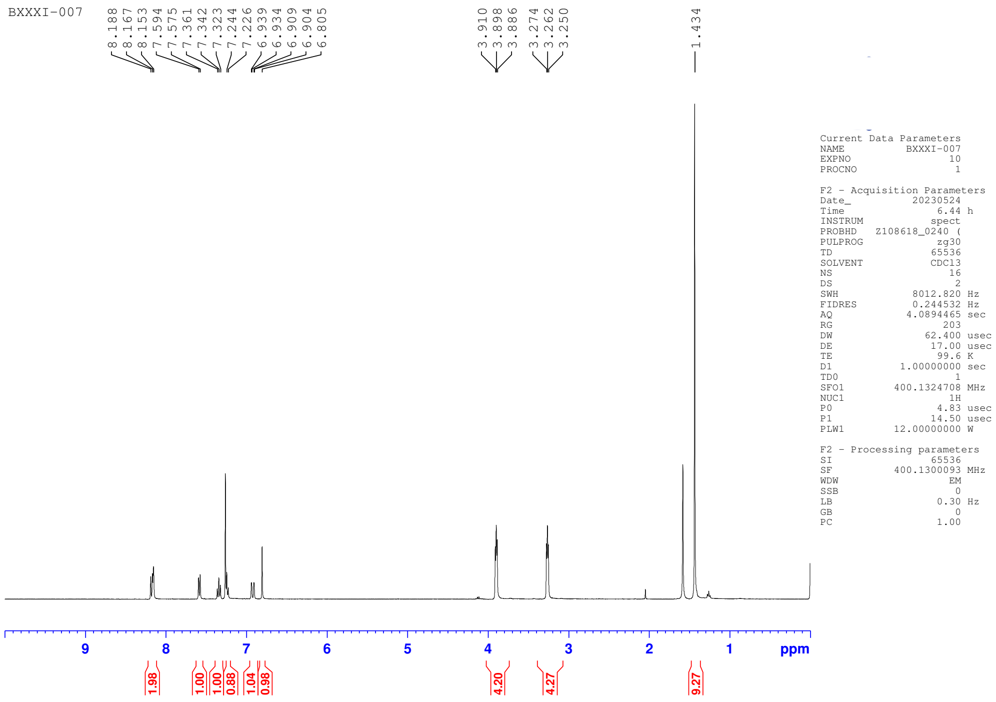

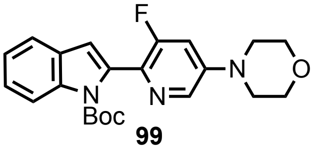

99

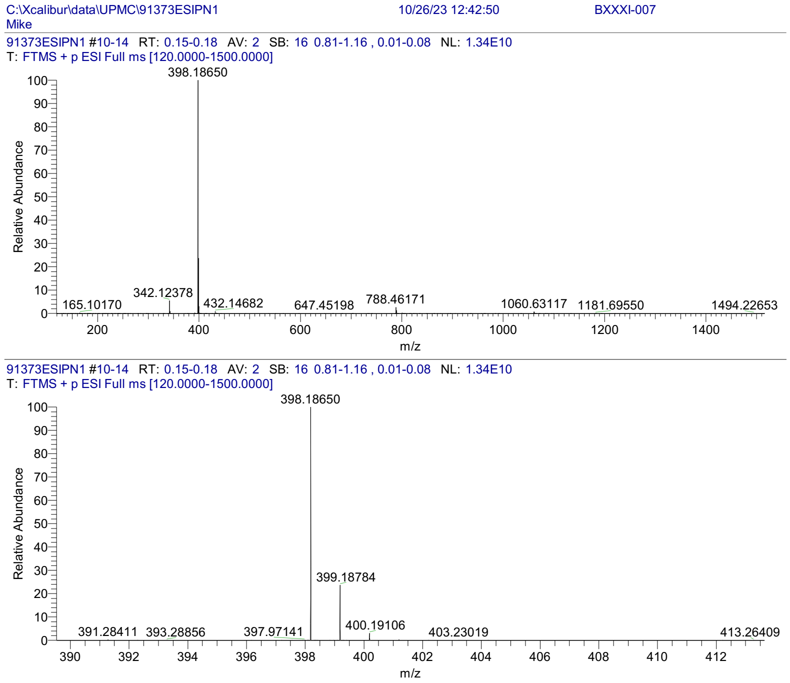

91373ESIPN1#11-13 RT: 0.15-0.18 AV: 2
SB: 16 0.81-1.16 , 0.01-0.08
T: FTMS + p ESI Full ms [120.0000-1500.0000]
m/z= 389.52597-413.63861

m/z	Intensity	Relative	Theo. Mass	Delta (ppm)	Composition
398.18650	13625583616.0	100.00	398.18745	-2.38	$C_{22}H_{25}O_3N_3F$

Figure S107. Compound **99** ^{1}H NMR spectrum (top) and high-resolution mass spectrum (bottom).

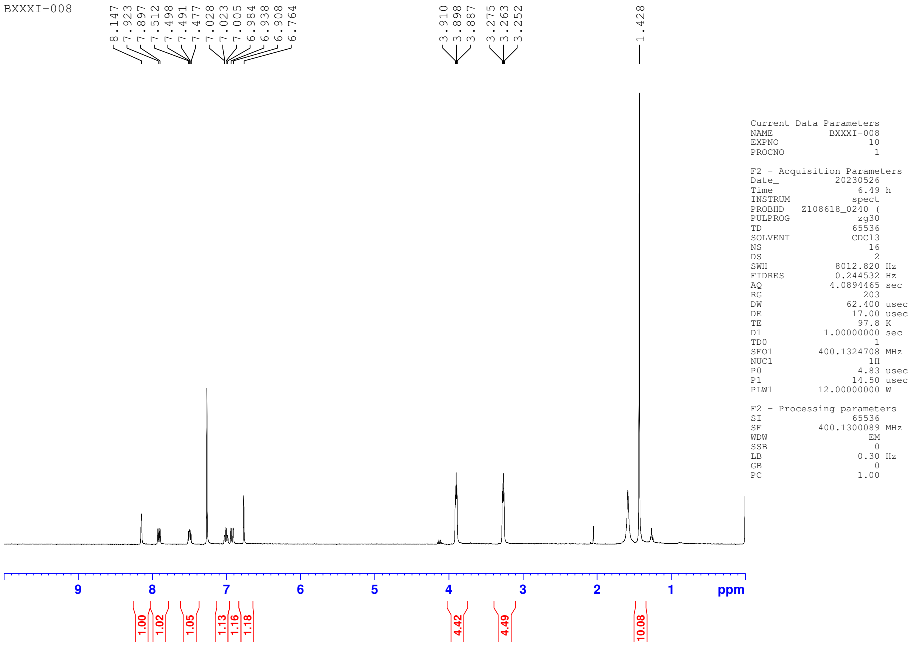

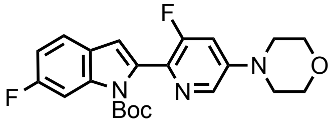

100

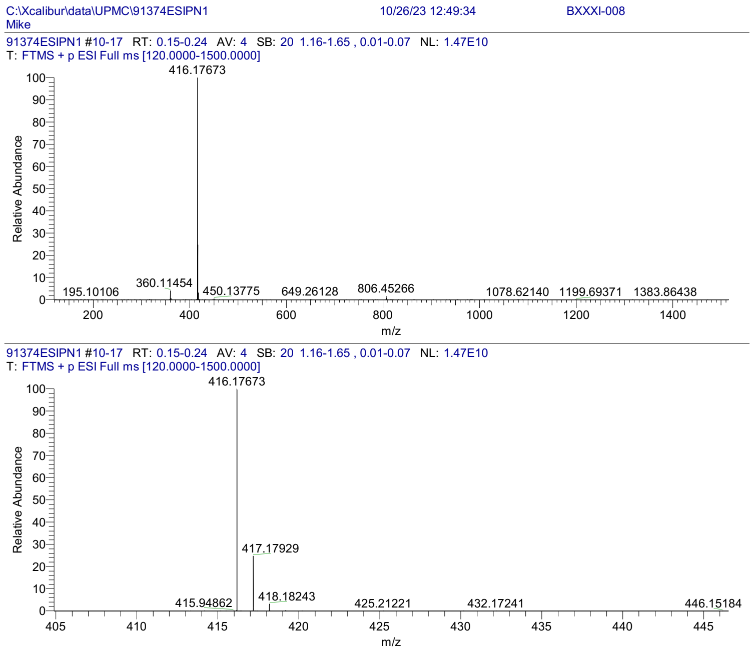

91374ESIPN1#11-17 RT: 0.15-0.24 AV: 4
SB: 20 1.16-1.65 , 0.01-0.07
T: FTMS + p ESI Full ms [120.0000-1500.0000]
m/z= 404.87475-446.52445

m/z	Intensity	Relative	Theo. Mass	Delta (ppm)	Composition
416.17673	15254100992.0	100.00	416.17802	-3.10	$C_{22}H_{24}O_3N_3F_2$

Figure S108. Compound **100** ^{1}H NMR spectrum (top) and high-resolution mass spectrum (bottom).

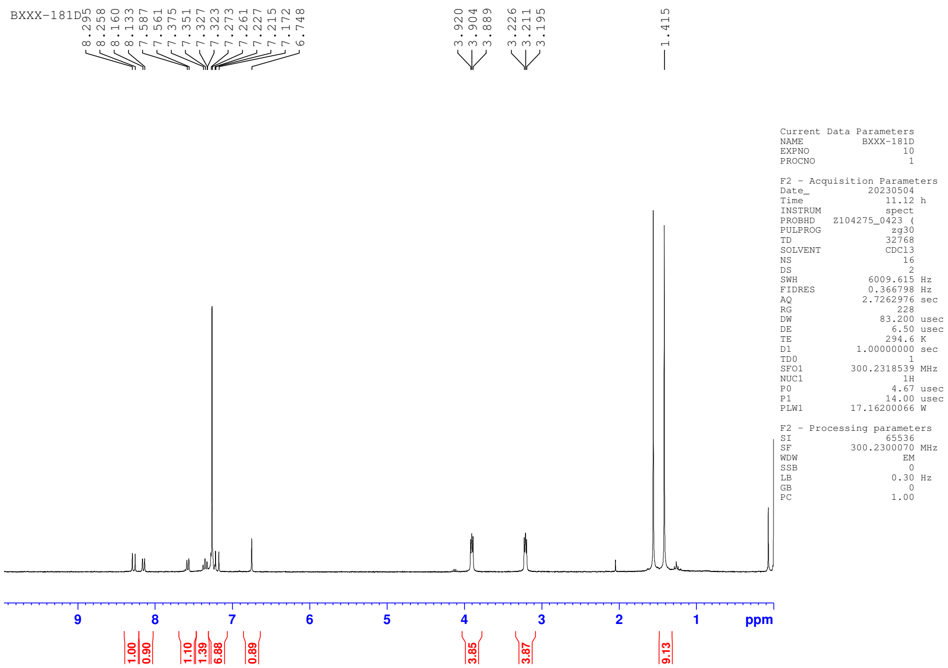

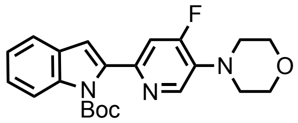

101

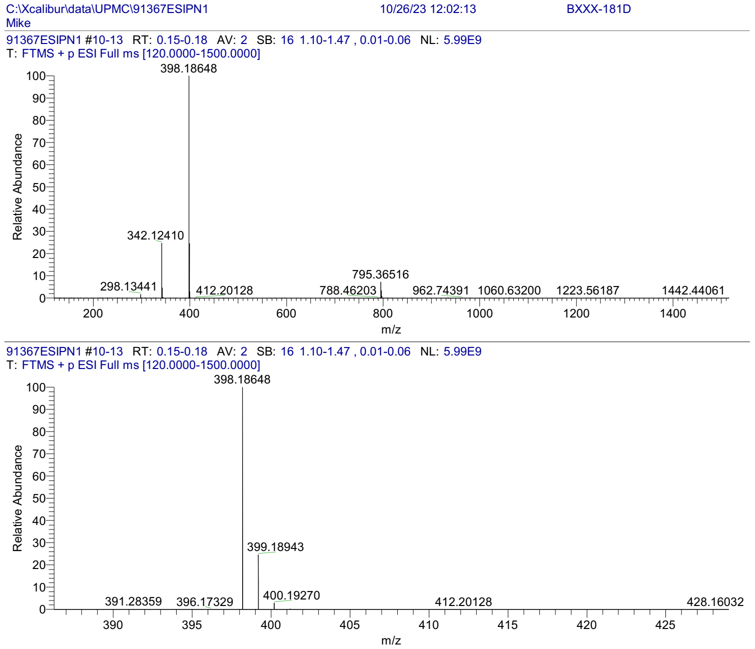

91367ESIPN1#11-13 RT: 0.15-0.18 AV: 2
SB: 16 1.10-1.47 , 0.01-0.06
T: FTMS + p ESI Full ms [120.0000-1500.0000]
m/z= 386.23993-428.98535

m/z	Intensity	Relative	Theo. Mass	Delta (ppm)	Composition
398.18648	6098129920.0	100.00	398.18745	-2.43	$C_{22}H_{25}O_3N_3F$

Figure S109. Compound **101** ^{1}H NMR spectrum (top) and high-resolution mass spectrum (bottom).

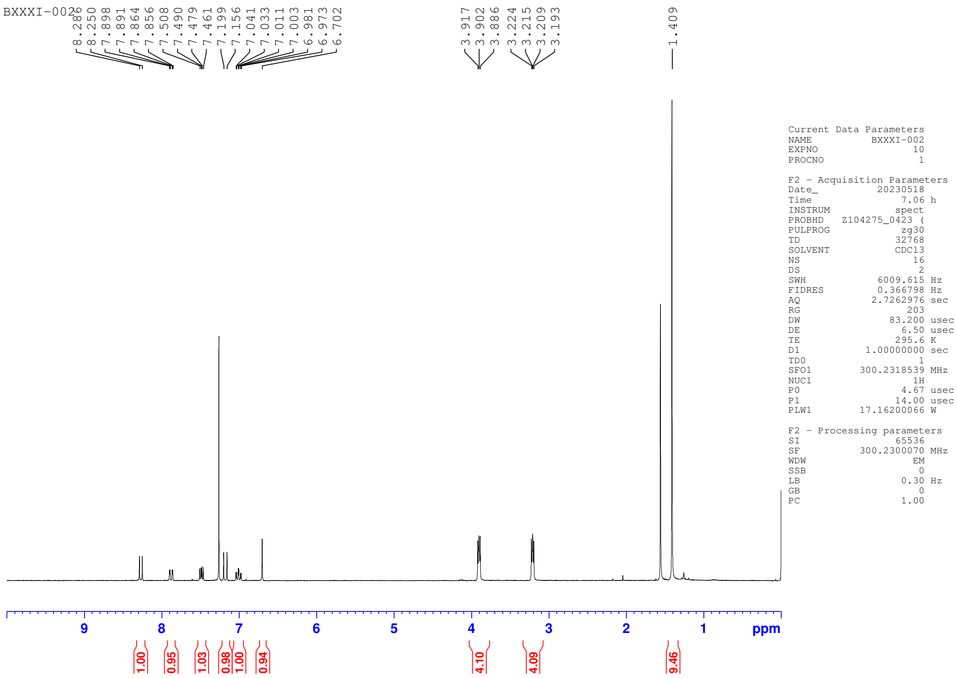

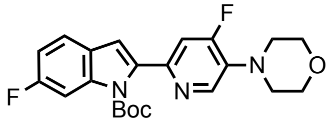

102

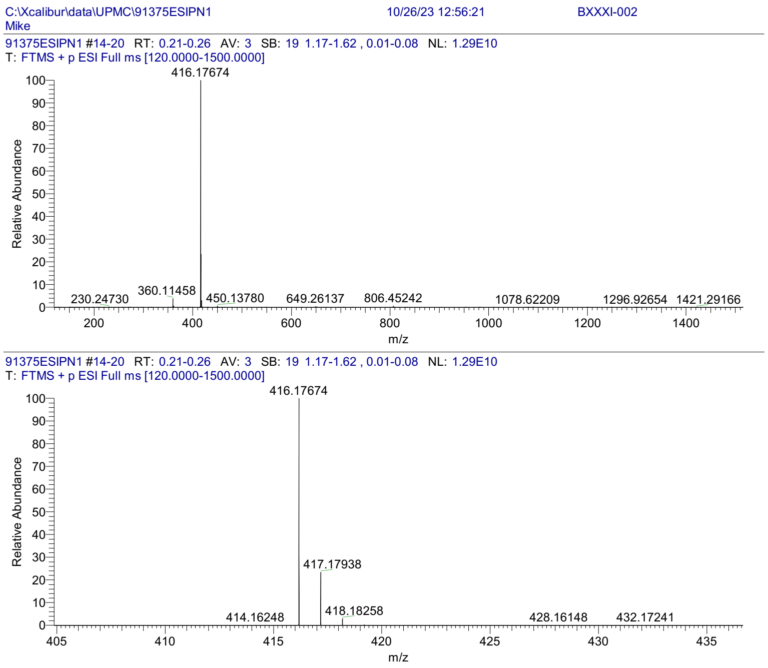

91375ESIPN1#15-19 RT: 0.21-0.26 AV: 3
SB: 19 1.17-1.62 , 0.01-0.08
T: FTMS + p ESI Full ms [120.0000-1500.0000]
m/z= 404.87234-436.65737

m/z	Intensity	Relative	Theo. Mass	Delta (ppm)	Composition
416.17674	13182138368.0	100.00	416.17802	-3.09	$C_{22}H_{24}O_3N_3F_2$

Figure S110. Compound **102** ^{1}H NMR spectrum (top) and high-resolution mass spectrum (bottom).

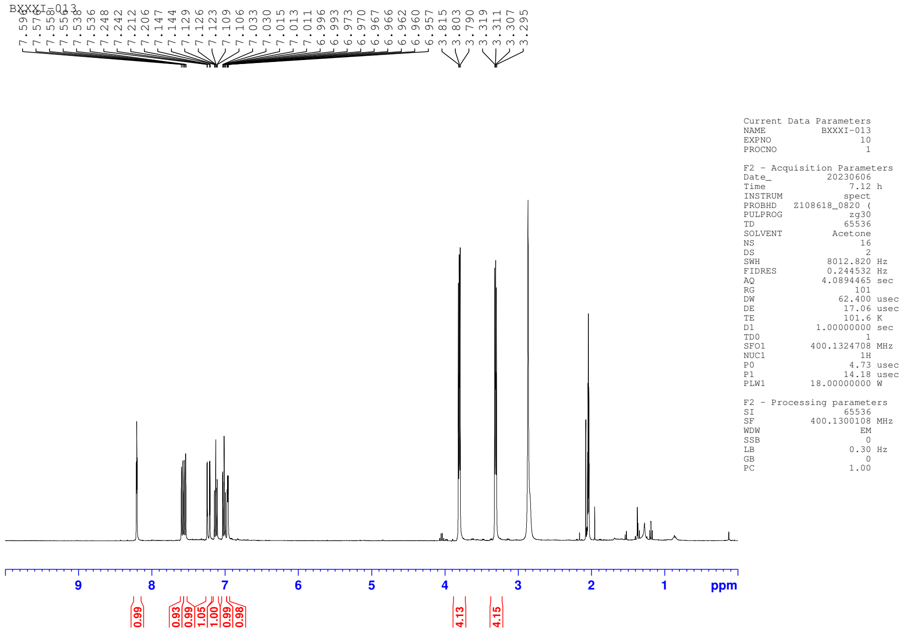

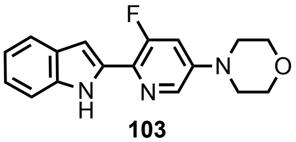

103

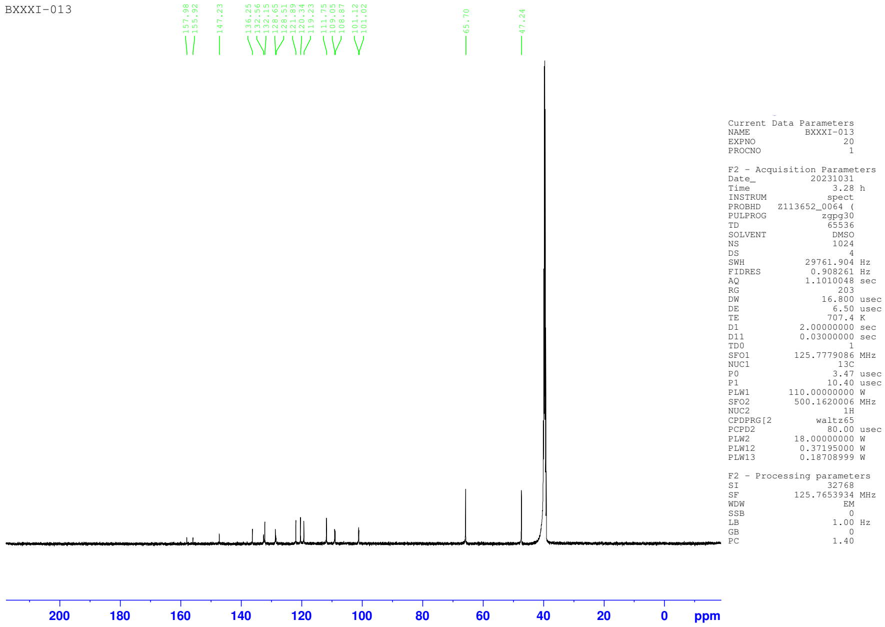

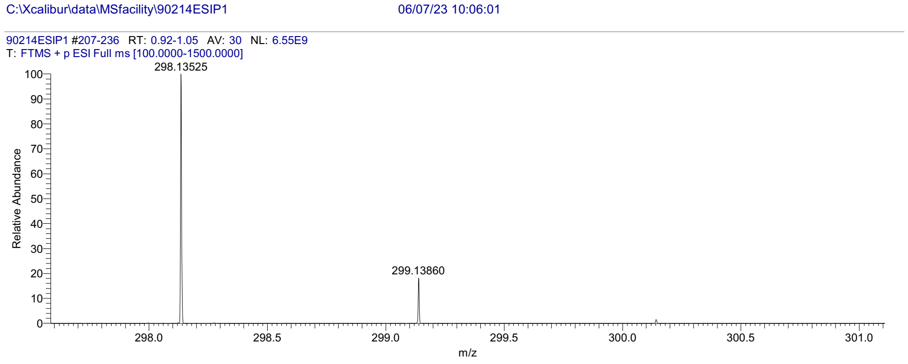

90214ESIP1#207-236 RT: 0.92-1.05 AV: 30
T: FTMS + p ESI Full ms [100.0000-1500.0000]
m/z= 297.58412-301.10757

m/z	Intensity	Relative	Theo. Mass	Delta (ppm)	Composition
298.13525	6702235136.0	100.00	298.13502	0.78	$C_{17}H_{17}ON_3F$

Figure S111. Compound **103** ^{1}H NMR spectrum (top), ^{13}C NMR spectrum (middle), high-resolution mass spectrum (bottom).

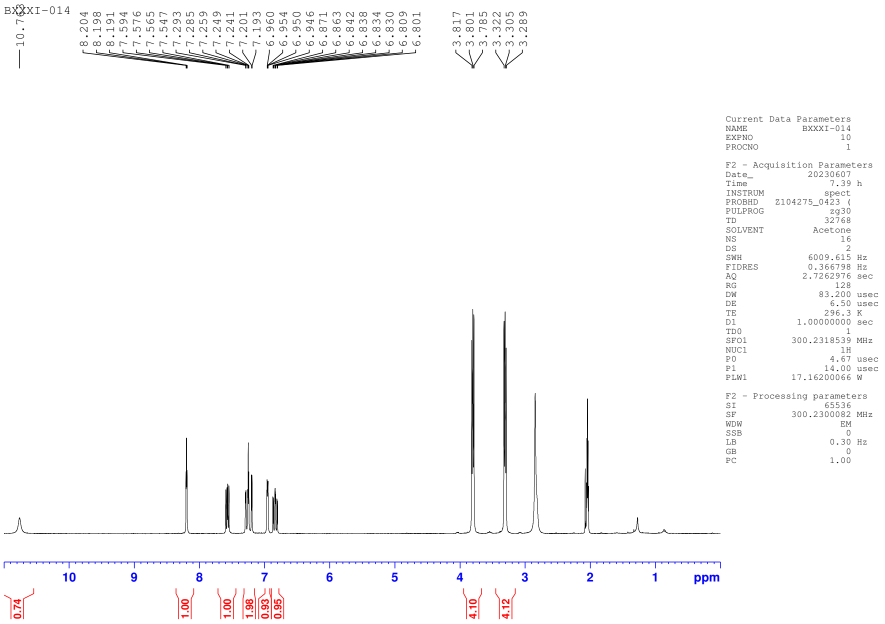

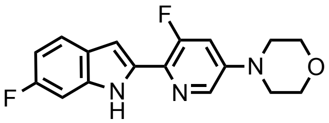

104

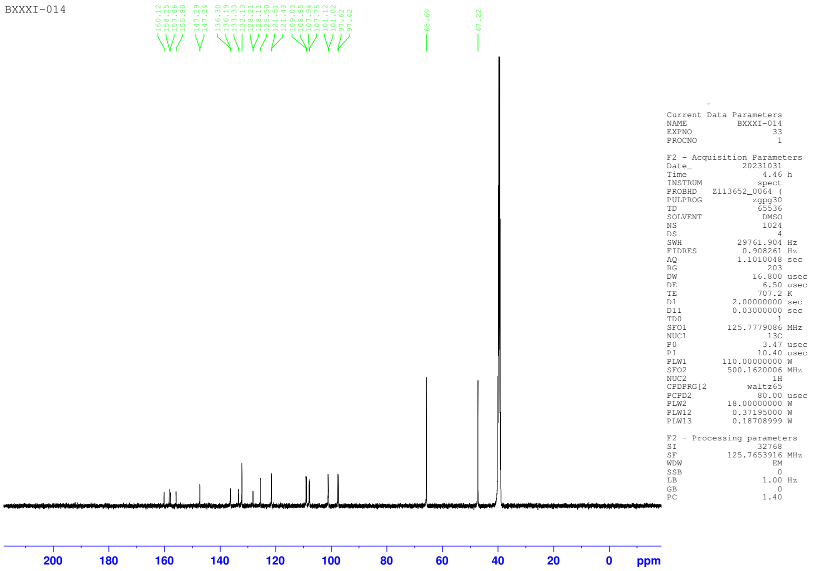

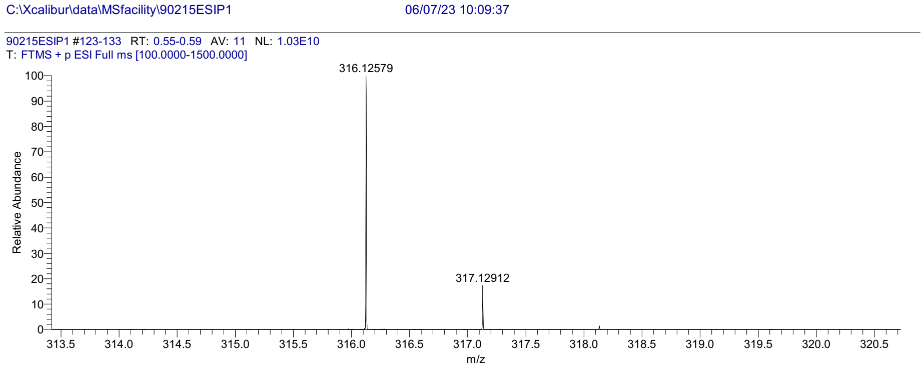

90215ESIP1#123-133 RT: 0.55-0.59 AV: 11
T: FTMS + p ESI Full ms [100.0000-1500.0000]
m/z= 313.41787-320.72321

m/z	Intensity	Relative	Theo. Mass	Delta (ppm)	Composition
316.12579	10461192192.0	100.00	316.12560	0.63	$C_{17}H_{16}ON_3F_2$

Figure S112. Compound **104** ^{1}H NMR spectrum (top), ^{13}C NMR spectrum (middle), high-resolution mass spectrum (bottom).

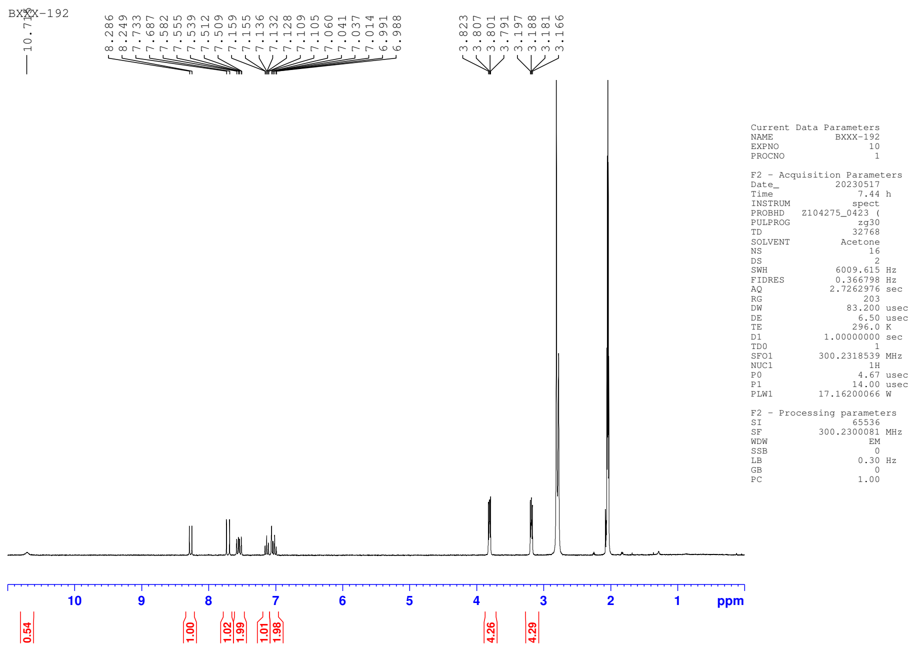

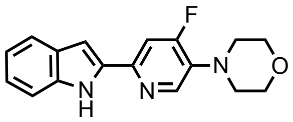

105

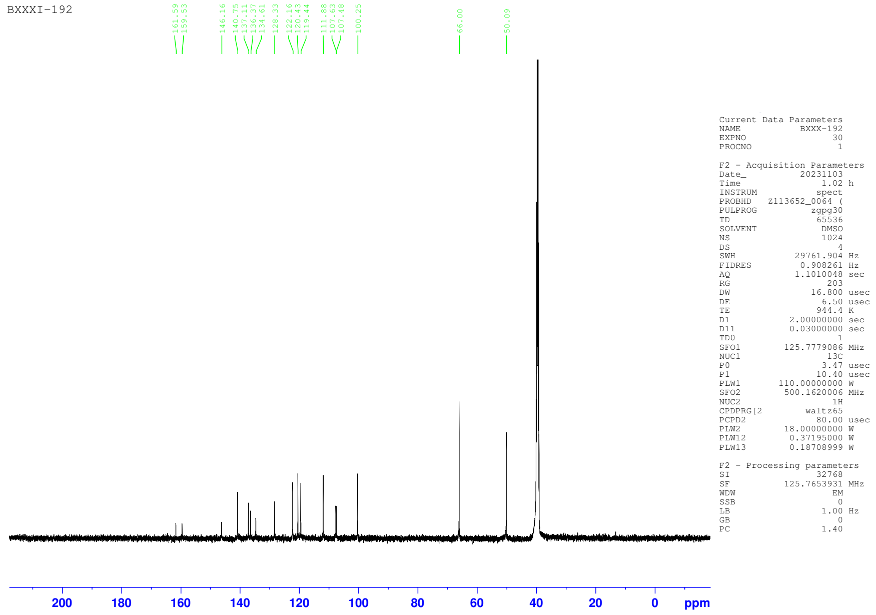

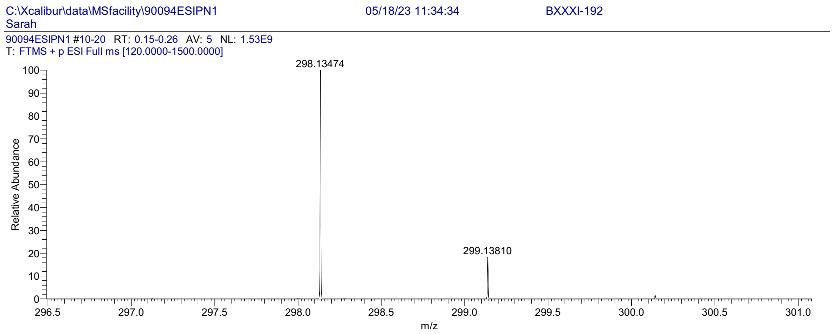

90094ESIPN1#11-19 RT: 0.15-0.26 AV: 5
T: FTMS + p ESI Full ms [120.0000-1500.0000]
m/z= 296.49209-301.08312

m/z	Intensity	Relative	Theo. Mass	Delta (ppm)	Composition
298.13474	1573469184.0	100.00	298.13502	-0.94	$C_{17}H_{17}ON_3F$

Figure S113. Compound **105** ^{1}H NMR spectrum (top), ^{13}C NMR spectrum (middle), high-resolution mass spectrum (bottom).

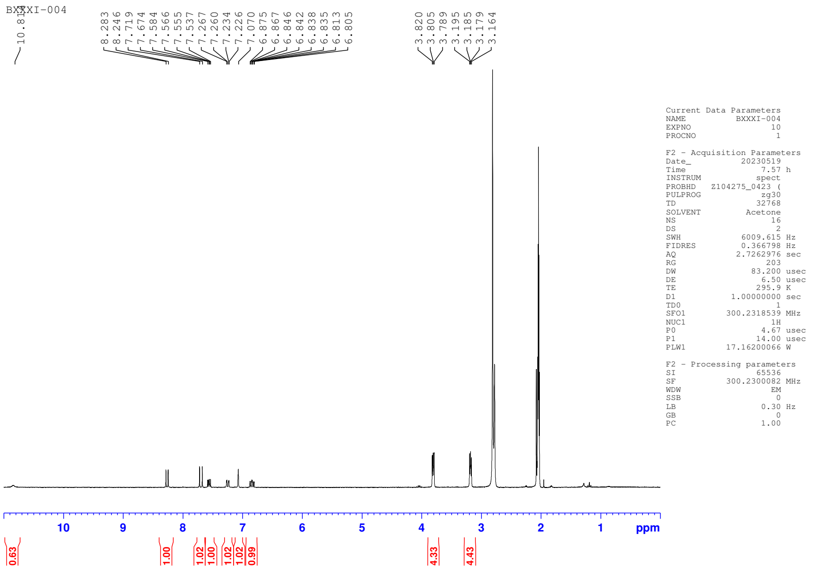

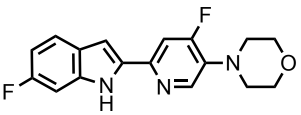

106

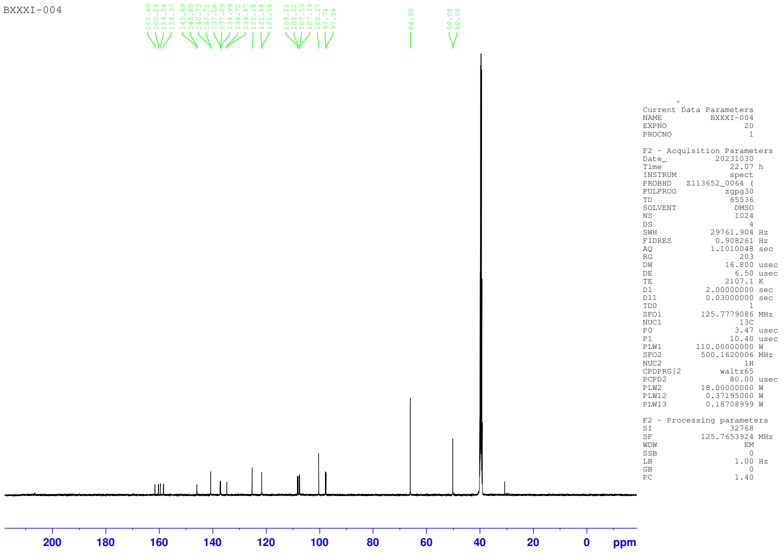

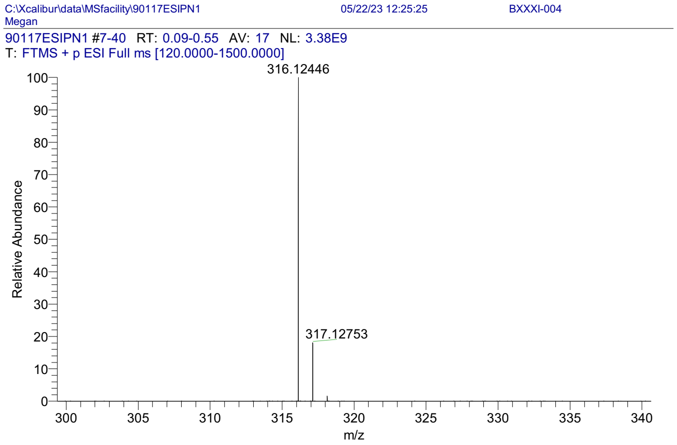

90117ESIPN1#7-39 RT: 0.09-0.55 AV: 17
T: FTMS + p ESI Full ms [120.0000-1500.0000]
m/z= 299.36949-340.62218

m/z	Intensity	Relative	Theo. Mass	Delta (ppm)	Composition
316.12446	3516484352.0	100.00	316.12560	-3.57	$C_{17}H_{16}ON_3F_2$

Figure S114. Compound **106** ^{1}H NMR spectrum (top), ^{13}C NMR spectrum (middle), high-resolution mass spectrum (bottom).

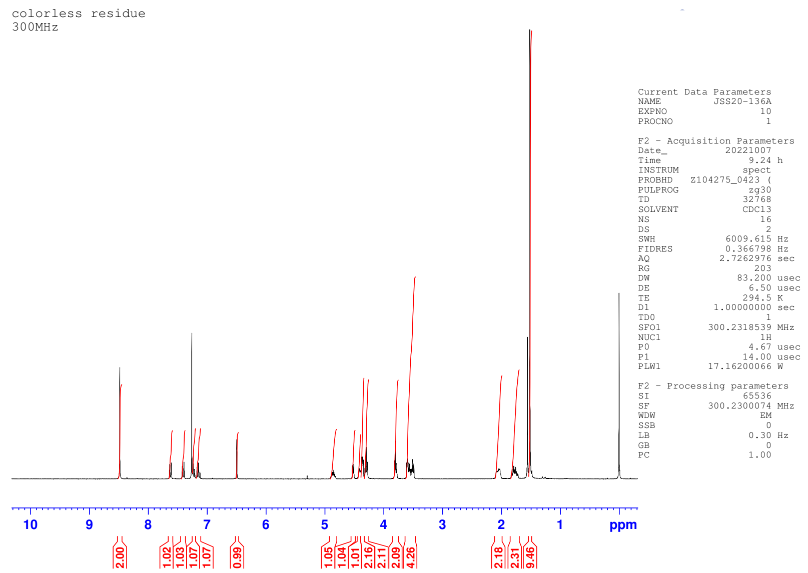

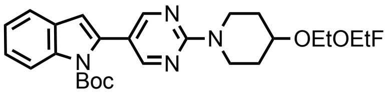

107

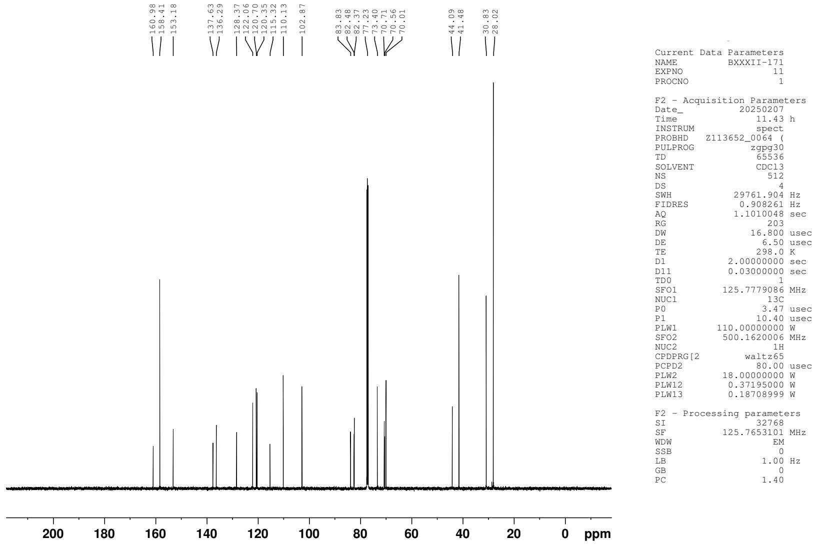

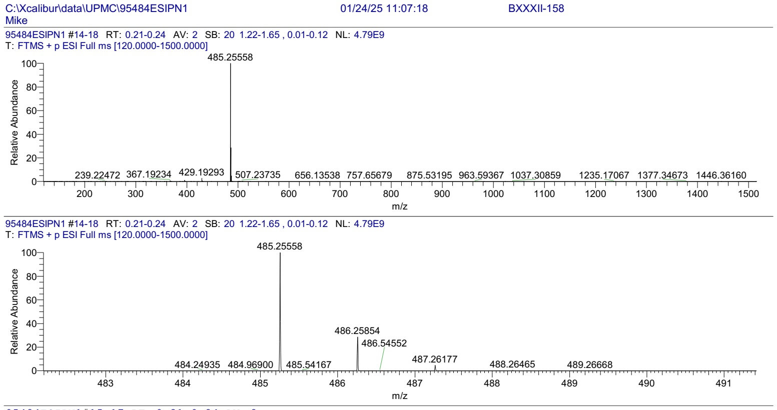

95484ESIPN1#15-17 RT: 0.21-0.24 AV: 2
SB: 20 1.22-1.65 , 0.01-0.12
T: FTMS + p ESI Full ms [120.0000-1500.0000]
m/z= 482.19338-491.41638

m/z	Intensity	Relative	Theo. Mass	Delta (ppm)	Composition
485.25558	4809057280.0	100.00	485.25586	-0.58	$C_{26}H_{34}O_4N_4F$

Figure S115. Compound **107** ^{1}H NMR spectrum (top), ^{13}C NMR spectrum (middle), and high-resolution mass spectrum (bottom).

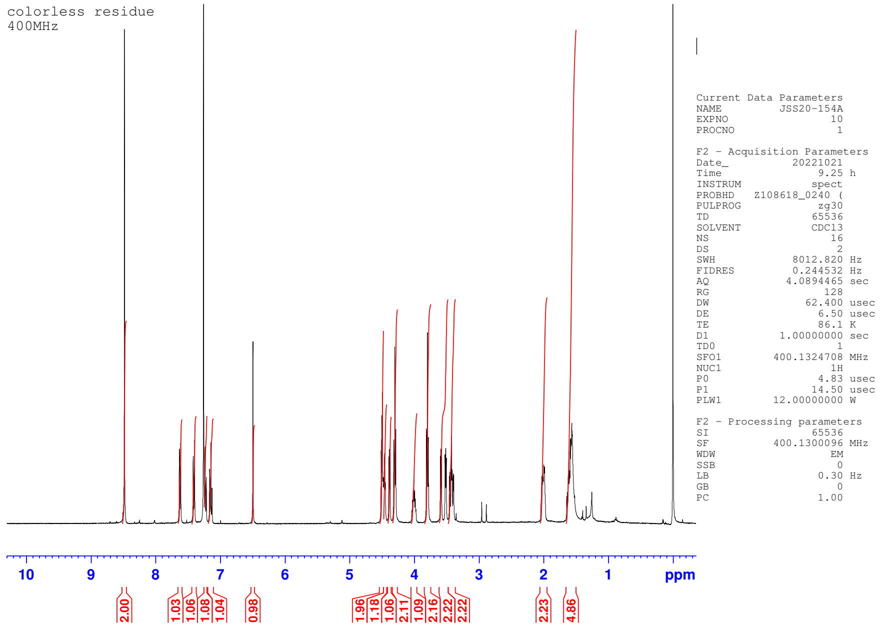

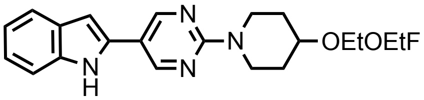

108

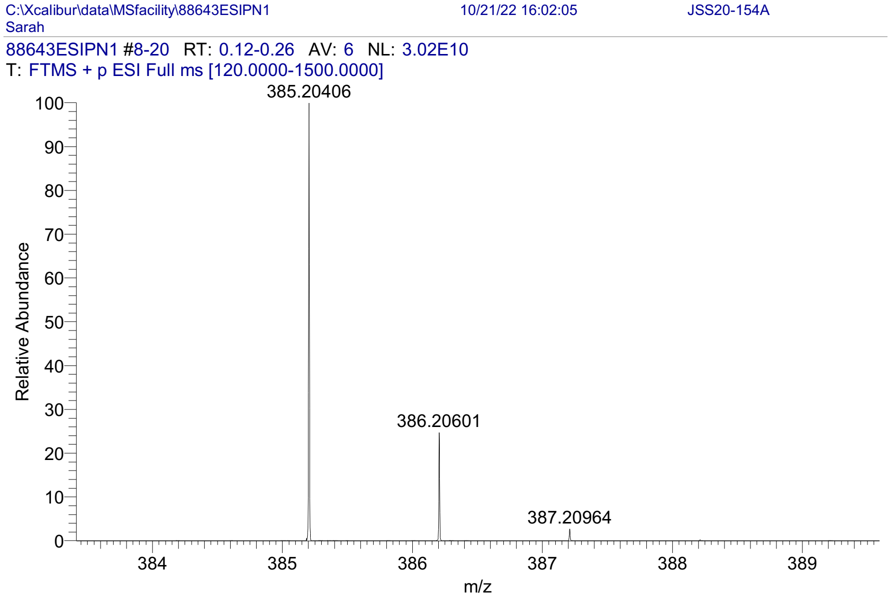

88643ESIPN1#9-19 RT: 0.12-0.26 AV: 6
T: FTMS + p ESI Full ms [120.0000-1500.0000]
m/z= 383.41298-389.59237

m/z	Intensity	Relative	Theo. Mass	Delta (ppm)	Composition
385.20406	31148318720.0	100.00	385.20343	1.64	$C_{21}H_{26}O_2N_4F$

Figure S116. Compound **108** ^{1}H NMR spectrum (top) and high-resolution mass spectrum (bottom).

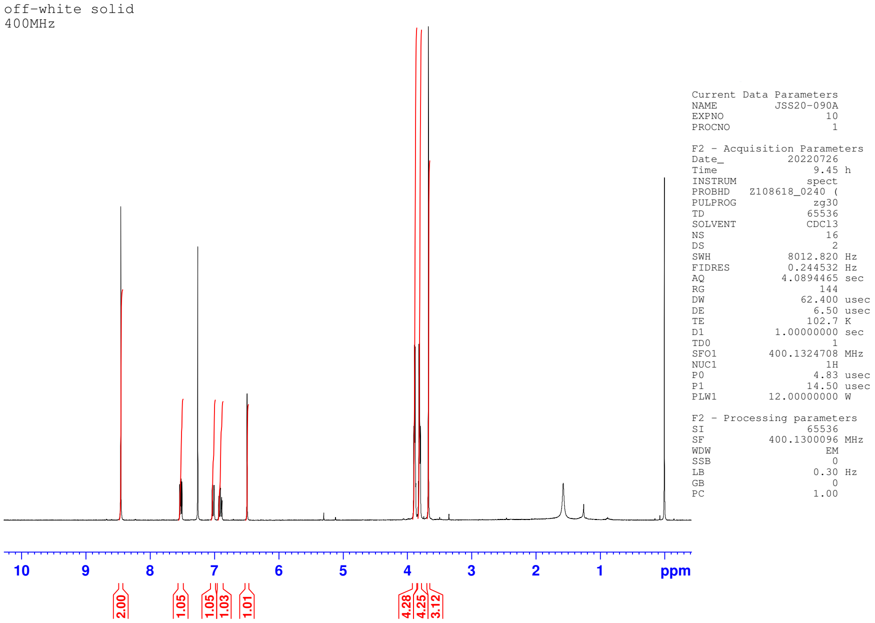

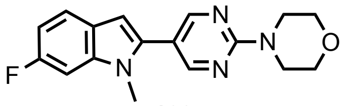

109

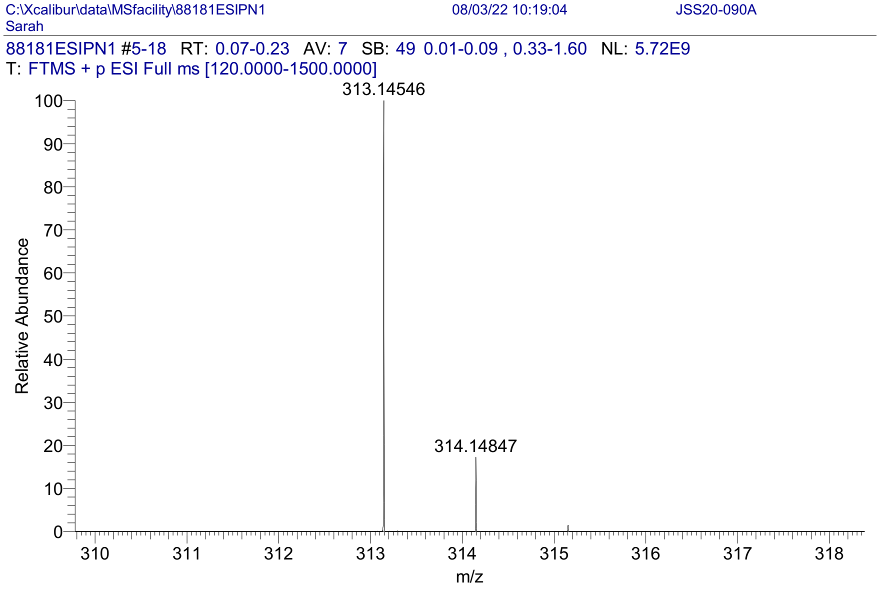

88181ESIPN1#5-17 RT: 0.07-0.23 AV: 7
SB: 49 0.01-0.09 , 0.33-1.60
T: FTMS + p ESI Full ms [120.0000-1500.0000]
m/z= 309.78090-318.38191

m/z	Intensity	Relative	Theo. Mass	Delta (ppm)	Composition
313.14546	5724209152.0	100.00	313.14592	-1.47	$C_{17}H_{18}ON_4F$

Figure S117. Compound **109** ^{1}H NMR spectrum (top) and high-resolution mass spectrum (bottom).

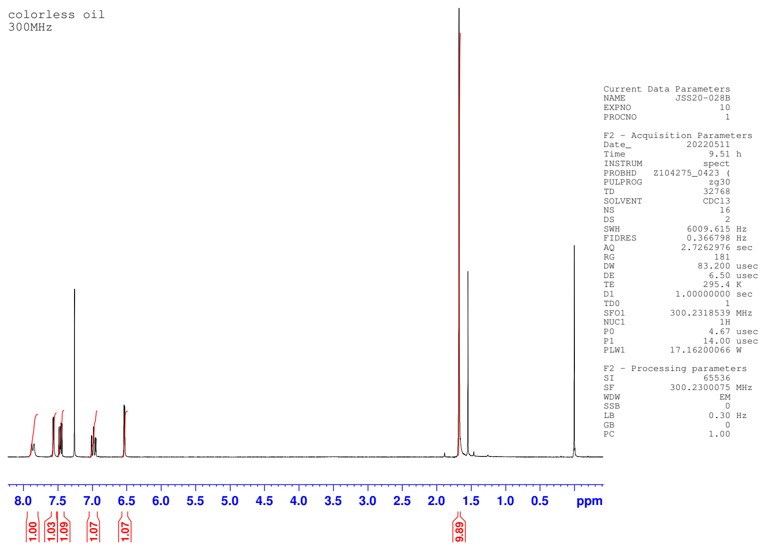

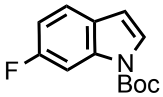

110

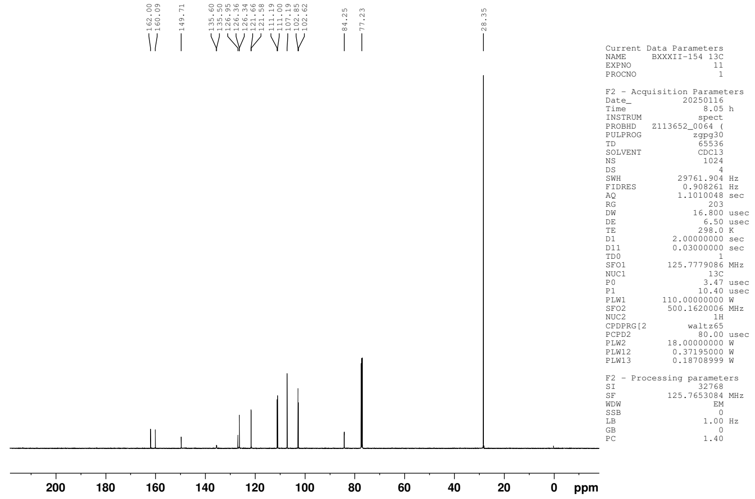

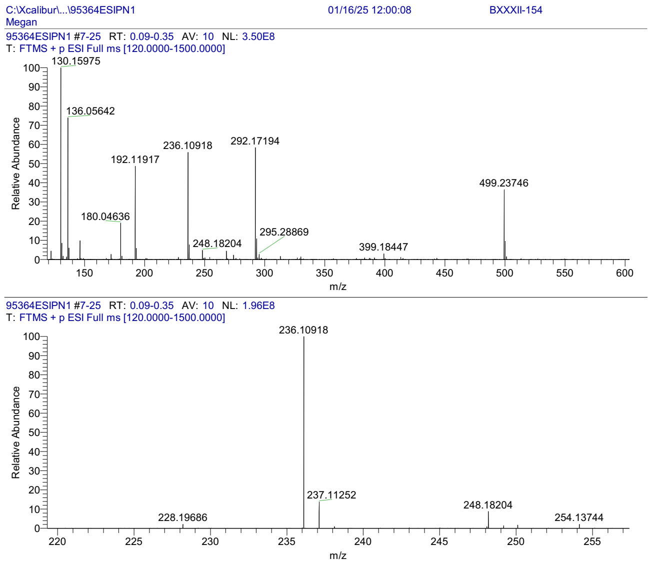

95364ESIPN1#7-25 RT: 0.09-0.35 AV: 10
T: FTMS + p ESI Full ms [120.0000-1500.0000]
m/z= 219.31316-257.40271

m/z	Intensity	Relative	Theo. Mass	Delta (ppm)	Composition
236.10918	196301616.0	100.00	236.10813	4.43	$C_{13}H_{15}O_2NF$

Figure S118. Compound **110** ^{1}H NMR spectrum (top), ^{13}C NMR spectrum (middle), and high-resolution mass spectrum (bottom).
